# Positive Electrochemical Potentials Induce Enhanced Chemoselectivity and Activity in the Thermocatalytic Hydrogenation of 3‐Nitrostyrene

**DOI:** 10.1002/anie.5569475

**Published:** 2026-06-08

**Authors:** Yunfei Jiao, Martina A. Pogany, Murielle F. Delley

**Affiliations:** ^1^ Department of Chemistry University of Basel Basel Switzerland

**Keywords:** chemoselectivity, electrochemical promotion of catalysis, hydrogenation, polarization, surface‐enhanced infrared absorption spectroscopy

## Abstract

Electrochemical promotion of catalysis is emerging as a powerful approach to control chemical reactions externally without complex catalyst modification, but this has mostly been used for substrates with a single reactive group so far. Herein, we demonstrate that this approach can induce full chemoselectivity in catalytic transformations of substrates with multiple functional groups. Specifically, we show that application of a positive electrochemical potential during the thermocatalytic hydrogenation (TCH) of 3‐nitrostyrene exclusively delivered one single product, 1‐ethyl‐3‐nitrobenzene, with six‐fold enhanced yield, while a complex product mixture was obtained at open circuit voltage. These effects resulted from the combined effects of a positive electrode polarization and acidification of the bulk solution through a concurrent hydrogen oxidation reaction (HOR). Surface‐enhanced infrared absorption spectroscopy and kinetic experiments showed that the positive polarization optimized the surface concentrations of reactants and the adsorption properties of 3‐nitrostyrene and hydrogenation products. Validation with additional reactants and catalyst implies a possible broader relevance of our findings beyond the initial system of 3‐nitrostyrene hydrogenation. Overall, our work provides fundamental insight into the effects of electrode polarization on thermal hydrogenation reactions and opens new opportunities to use electrochemical promotion to control chemoselectivity and activity.

## Graphical abstract

Induced by positive electrochemical potentials, thermocatalytic hydrogenation (TCH) of 3‐nitrostyrene with H_2_ delivers one single product with six‐fold enhanced yield. The enhanced selectivity and activity originate from changes in the adsorption geometry of 3‐nitrostyrene and in the surface concentration of 3‐nitrostyrene and hydrogen via a concurrent hydrogen oxidation reaction (HOR).

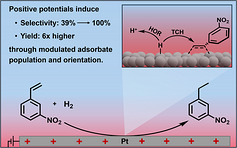

## 1 Introduction

Precise control of catalytic performance under actual reaction conditions remains a key goal in heterogeneous catalysis [1]. Adjusting catalytic properties on demand by an external control element would be of particular interest as this would circumvent complex catalyst modification strategies and could offer flexible use of one catalyst for different desired reaction outcomes. However, this continues to be exceptionally challenging. Electrochemical promotion of catalysis (EPOC), also termed non‐Faradaic electrochemical modification of catalytic activity (NEMCA), refers to a phenomenon in which the catalytic activity or selectivity of a thermal reaction can be enhanced in situ in a reversible fashion by applying an external electrochemical potential [2, 3]. On a fundamental level, EPOC can be caused by the interaction of the applied electric field with electric dipole moments of substrates and transition states having different magnitude or orientation [4]. However, due to the complexity of solid‐fluid interfacial chemistry in an external electric field [5], EPOC can also result from several other phenomena including interfacial ion migration [2, 3, 6], varied surface concentrations [7, 8], promoted interfacial charge transfer [9], change in local pH [10, 11], and competing electrochemical pathways [11, 12]. EPOC using heterogeneous catalysts has been demonstrated in a few but spectacular cases, including thermal oxidations [13, 14, 15], Brønsted acid‐catalyzed dehydration [9, 16], epoxide rearrangements [17], anion–π catalysis [18, 19], hydrogenations [7, 8, 11, 20], carbene reaction [21], and methane steam reforming [22]. These precedents highlight EPOC as a powerful approach to control thermal reactions externally. Nevertheless, the benefit of EPOC has mostly been shown for reactions involving the transformation of a single functional group so far. We propose that EPOC could also provide a potent way to achieve chemoselective catalysis for substrates with multiple functional groups.

Chemoselective hydrogenation is a prominent challenge in the production of chemicals [23, 24]. Highly chemoselective hydrogenation has been achieved using chemically or structurally modified catalysts, but this often requires complex adaptation of the catalyst preparation method [25, 26]. In addition, this often comes at the cost of catalytic activity [23]. EPOC could overcome these limitations. Indeed, prior work has shown that enhanced hydrogenation of substrates with one reducible group, that is ethylene [7, 8], acetylene [7], and CO_2_ [11, 20], can be obtained in an external bias. We hypothesized that an external bias could also induce chemoselectivity for substrates with multiple functional groups. This seems plausible in view of prior literature on chemoselective hydrogenations achieved through electronic effects imposed by a modified catalyst [23, 27]. For instance, chemoselective hydrogenation of nitroarenes or α,β‐unsaturated aldehydes has been obtained with bimetallic catalysts, catalysts doped with more electropositive elements, or electrostatic effects in confined spaces [26, 28, 29, 30].

In this work, we are testing whether chemoselective thermal hydrogenation can be achieved by employing electrochemical potentials. We probe the effect of a positive applied potential on the thermocatalytic hydrogenation of 3‐nitrostyrene (3‐NS) using a Pt catalyst. We demonstrate a substantial enhancement of not only chemoselectivity but also activity for the thermal hydrogenation of the vinyl‐group in 3‐NS that does neither come from an electrochemical reduction of 3‐NS nor a change in catalyst structure. Combined control experiments, kinetic studies and surface‐enhanced infrared absorption spectro‐electrochemistry (EC‐SEIRAS) suggest that the promotion of catalytic activity and selectivity at a positive applied potential is due to changes in surface concentration of the substrates induced by the positive polarization and a concurrent hydrogen oxidation reaction as well as an enhanced affinity and activation of electron‐rich over electron‐deficient functional groups at the surface, respectively.

## 2 Results and Discussion

### 2.1 Thermal 3‐NS Hydrogenation as a Function of Applied Potential

**FIGURE 1:**
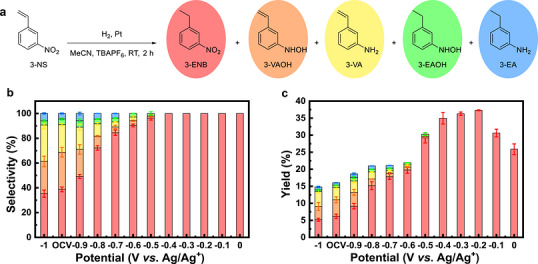
(a) Thermal 3‐NS hydrogenation and possible products: 1‐ethyl‐3‐nitrobenzene (3‐ENB, red), 3‐vinyl‐*N*‐hydroxyaniline (3‐VAOH, orange), 3‐vinylaniline (3‐VA, yellow), 3‐ethyl‐*N*‐hydroxyaniline (3‐EAOH, green), and 3‐ethylaniline (3‐EA, blue). (b) Selectivity and (c) yield of the thermal 3‐NS hydrogenation as a function of applied potential measured after 2 h reaction time at each applied potential. Mean and standard deviation shown for each set of conditions in (b) and (c) were derived from three independent replicate experiments.

To test whether chemoselectivity in thermal hydrogenation reactions can be controlled by external polarization, we probed the room‐temperature thermal hydrogenation [31] of 3‐NS as a function of applied potential in an H‐cell (Figures 1a and S1). For the 3‐NS hydrogenation, a Pt catalyst deposited on a Ni foam was used in a solution of 100 mM tetrabutylammonium hexafluorophosphate (TBAPF_6_) in acetonitrile (MeCN) that was sparged with a constant flow of H_2(g)_ at 1 bar. Reaction products were identified by taking aliquots of the mixture at different time intervals and analysis by NMR. The reaction was tested at open circuit voltage (OCV) as well as at different applied potentials between −1 V and 0 V versus Ag/Ag^+^ applied to the Pt catalyst. Aprotic solvents and other reaction conditions were chosen such to ensure stability within the potential window examined and to reduce the probability for electrochemical hydrogenation (Figure S15) [7, 32]. The Ni foam has no catalytic activity (Figure S18).

Both selectivity and activity of the Pt catalyst for thermal 3‐NS hydrogenation was strongly promoted by the positive applied potentials (Figure 1b,c). At OCV, we observed five products from full and partial hydrogenation of the nitro‐ or vinyl‐groups in 3‐NS (Figure 1a). At positive applied potentials, 1‐ethyl‐3‐nitrobenzene (3‐ENB) was the only product observed. Selectivity for 3‐ENB increased from 39% at OCV to 100% above −0.4 V. The yield for 3‐ENB was also substantially enhanced at positive applied potentials from 6% at OCV to 37% at −0.2 V after 2 h reaction time. At more positive applied potentials, the yield decreased again perhaps due to limiting hydrogen (see below). Herein, we use an applied potential of −0.2 V that gave the highest selectivity and yield as a standard potential to compare to the reaction at OCV. At −0.2 V, full conversion could be reached in a quarter of the time needed at OCV and 3‐ENB was produced as the only product even at full conversion (Figure S17).

To minimize possible effects from electrocatalytic hydrogenation of 3‐NS by residual water in MeCN, we examined 3‐NS hydrogenation at positive applied potential versus *E*_OCV_, ca. −0.9 to 0 V versus Ag/Ag^+^ where no electroreduction of 3‐NS should occur (Figure S15) [33, 34]. In this potential window, we measured positive currents suggesting that oxidative processes dominate (Figure S19 and Table S4). No formation of products was observed in absence of H_2_ showing that H_2_ is necessary for catalysis (Figure S20). Combined, this suggests that there is no significant electrochemical hydrogenation under our conditions and observed products derive from thermal hydrogenation.

Running the 3‐NS hydrogenation iteratively at OCV, at −0.2 V, and again at OCV with the same Pt‐catalyst showed comparable catalytic performance in the second OCV reaction compared to the first (Figure S21). This shows that the applied potential does not permanently change the catalyst. This is supported by our analysis of the spent catalysts at OCV and −0.2 V by scanning electron microscopy, x‐ray photoelectron spectroscopy, and powder x‐ray diffraction, which showed similar features (Figures S22–S24 and Table S6). Additional control experiments showed that any possible catalyst leaching for example by dissolution or exfoliation of Pt to solution, did not significantly contribute to our observations at OCV or −0.2 V (Figure S25).

In sum, we observed a substantial enhancement of selectivity and activity in the thermal hydrogenation of 3‐NS through an applied electrochemical potential that is attributable to an external tuning of the thermal reaction in situ.

### 2.2 The Effect of the Concurrent Hydrogen Oxidation Reaction

At the catalyst/electrolyte interface H_2_ can dissociate on Pt surface sites (*). The thereby formed surface hydrogen (H*) may be oxidized to H^+^ via $1/2\;H_{2}+*⇌H^{*}⇌H_{\text{aq}}^{+}+e_{\text{metal}}^{-}+*$. Consequently, sparging the reaction mixture with H_2_ decreased the *E*_OCV_ from ca. −0.3 V to ca. −1.1 V (Figure S2) and the solution ^MeCN^pH in MeCN from 6.3 to 5.9 after 20 min (see SI: Section S2 for a more detailed discussion of ^MeCN^pH measurements). A cyclic voltammogram under reaction conditions showed that applied potentials above −1 V drive the hydrogen oxidation reaction (HOR) (Figure S15). During 3‐NS hydrogenation at −0.9 to 0 V, we observed a positive current and the ^MeCN^pH in the working chamber was substantially lowered (Figure S19 and Table S4). The corresponding reductive current at the counter electrode likely mainly corresponds to the hydrogen evolution reaction based on the increased ^MeCN^pH values in the counter chamber and an observed bubble formation (see SI: Section S4.4 for a more detailed discussion). Our data suggest that HOR occurred concurrently with the thermal hydrogenation of 3‐NS in the working chamber at positive applied potentials (but not at OCV), which could influence the thermal catalysis [11, 12, 35].

Two general possibilities can be considered, in which the thermal catalysis is influenced by the concurrent HOR. First, a coupled electrochemical‐thermal process could in principle occur, in which HOR provides H^+^ in solution that add to 3‐NS in a Faradaic reaction (i.e., for 3‐ENB: (step 1) H_2_ → 2 H^+^ + 2 *e^–^*, (step 2) 3‐NS + 2 H^+^ + 2 *e^–^* → 3‐ENB). Alternatively, an EPOC process is possible, in which the hydrogenation occurs as a non‐Faradaic reaction (i.e., for 3‐ENB: 3‐NS + H_2_ → 3‐ENB) that is indirectly influenced by the concurrent HOR. The coupled electrochemical‐thermal process is not typically observed with H_2_ as substrate [8]. In addition, the lack of product formation in the absence of H_2_ despite the availability of protons in solution suggests that a coupled electrochemical‐thermal process does not occur to a significant extent herein, though, the positive potential limits availability of electrons (see below, and Figure S20, Table S13). To further differentiate between the two possibilities, we probed the hydrogenation of 3‐NS with H_2_ at OCV and at −0.2 V in presence of D_2_O (SI: Section S8.2). The observed similar fractions of deuterated 3‐ENB at OCV and −0.2 V can likely be attributed to H/D exchange between surface hydrogen and D_2_O and strongly suggests that H^+^ formed from HOR are not to a significant extent incorporated in 3‐ENB (Tables S9–S11). If this was the case, fast exchange between D_2_O and H^+^ in solution should have resulted in a significantly higher fraction of deuterated 3‐ENB at −0.2 V than at OCV. The data hence suggest that the coupled electrochemical‐thermal process does not occur and that the observed catalytic enhancement at −0.2 V should be attributed to EPOC.

**FIGURE 2:**
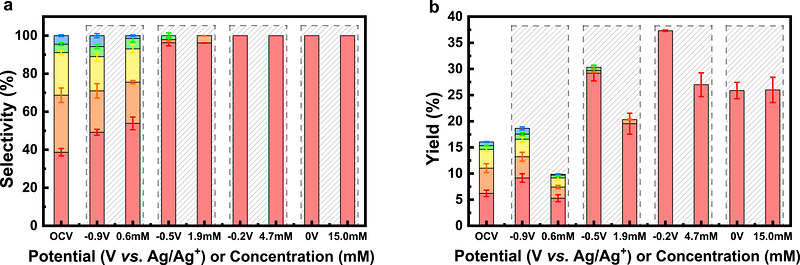
(a) Selectivity and (b) yield of the 3‐NS hydrogenation at OCV with addition of different amounts of HPF_6_ and comparison to data obtained at different applied potentials (reproduced from Figure 1 and shown here for comparison). Colored bars correspond to the observed products: 3‐NS (red), 3‐VAOH (orange), 3‐VA (yellow), 3‐EAOH (green), and 3‐EA (blue). Mean and standard deviation shown for each set of conditions were derived from three independent replicate experiments.

However, proton formation at −0.2 V may still contribute to the observed enhanced 3‐ENB selectivity [36]. To test this hypothesis, we added HPF_6_ as an external proton source to the hydrogenation reaction at OCV. HPF_6_ concentration was chosen such to match the proton concentration expected at four different applied potentials if all observed oxidation currents at these potentials were due to HOR. The *E*_OCV_ increased upon addition of HPF_6_ as expected from the Nernst equation (Figure S26). Consistent with the above hypothesis, the addition of HPF_6_ influenced 3‐NS hydrogenation in a similar manner as positive applied potentials: it increased the selectivity for 3‐ENB up to 100% and the yield by up to a factor of 4 (Figure 2). Control experiments showed that the observed effects are attributable to the added H^+^ and not to water content or PF_6_^–^ (Table S8 and Figure S28). However, even when large amounts of HPF_6_ were added the high yield enhancement obtained at −0.2 V was not reached. This implies that additional aspects apart from HOR contribute to the effects observed at positive applied potential.

We tested whether the lower yield increase obtained with HPF_6_ versus that obtained at −0.2 V (Figure 2b) is due to an additional proton source in the system. The ^MeCN^pH in the working chamber after reactions at positive applied potential was indeed much lower than what would be expected based on the observed HOR currents (Table S12). Control experiments showed that the ^MeCN^pH in the working chamber is lowered even in absence of H_2_ at positive applied potentials but not at OCV (Table S13). We tentatively attribute this to the release of protons at positive applied potentials from the Nafion N‐117 membrane used to separate working and counter chambers. However, comparison of 15 versus 4.7 mM HPF_6_ added to the reaction at OCV suggests that the yield cannot be further enhanced with larger amounts of H^+^ (Figure 2b). This suggests that protons released from the Nafion membrane cannot account for the observed higher catalytic activity enhancement with applied potentials and that the acidification of the working chamber solution is not the only mechanism of catalytic enhancement at positive applied potentials. This is consistent with our experiments in a single compartment cell (Figure S38), which still showed a substantial enhancement of selectivity and yield at −0.2 V compared to OCV even though the bulk ^MeCN^pH values were similar (perhaps due to H^+^ reduction at the counter electrode, Table S14).

Overall, the data show that the addition of H^+^ to the bulk solution can enhance selectivity and activity in a similar manner as positive applied potentials. This seems logical in view of the connection between the electrochemical potentials of surface hydrogen, H^+^ in solution and electrons in Pt that suggests that the Pt catalyst in contact with H_2_ will spontaneously positively polarize when decreasing the solution pH [37]. Prior work suggests that such a “wireless” polarization of a catalytic material by redox species in solution can enhance thermal catalysis in a similar manner as applied potentials [9]. In our case, H^+^ produced by HOR and other sources could contribute to the positive polarization of Pt that is obtained with a positive applied potential, which would explain the higher enhancement of selectivity and activity observed in an H‐cell setup compared to the single compartment cell (Figure S38). However, the same effects as obtained with a positive applied potential cannot be fully achieved by just changing the pH of the solution and some effects can even be obtained without pH change of the bulk solution. This highlights that the approach of applying a potential to enhance catalytic performance is more nuanced and powerful than simple pH changes of the bulk solution. Furthermore, while the addition of H^+^ can phenomenologically reproduce the catalytic enhancement to some extent, the underlying mechanism of this enhancement is not clear and will be further examined below.

### 2.3 The Origin of the Enhanced Catalytic Performance at Positive Applied Potentials

**FIGURE 3:**
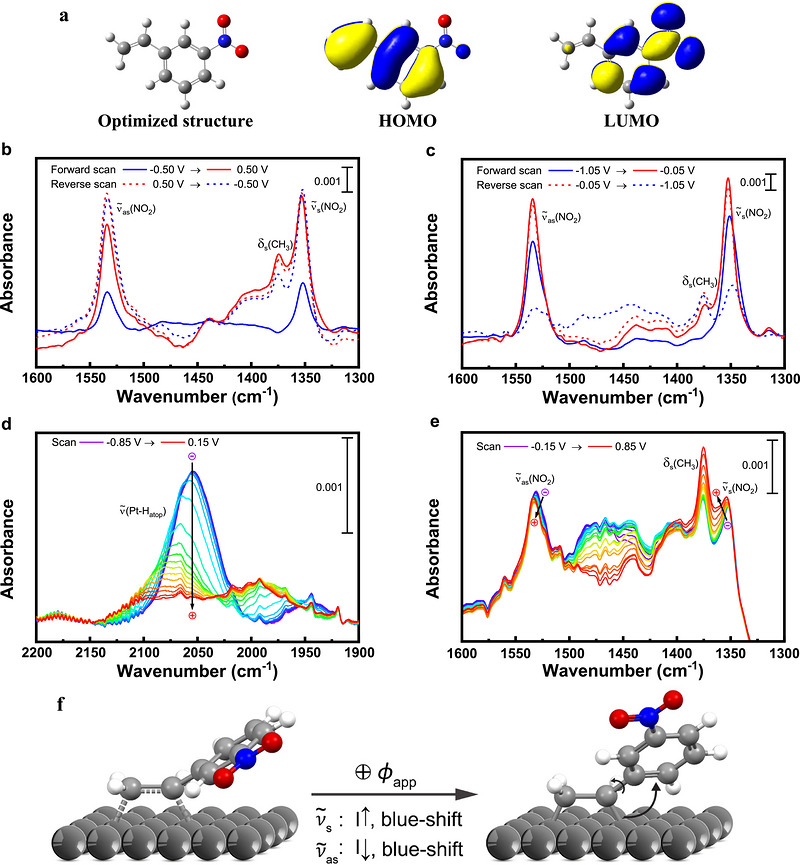
(a) Optimized structure of 3‐NS, and its HOMO (highest occupied molecular orbital) and LUMO (lowest unoccupied molecular orbital) as calculated from DFT. EC‐SEIRA spectra of a solution of 100 mM 3‐NS and 100 mM TBAPF_6_ in MeCN on a Pt electrode in (b) absence or (c) presence of H_2_ at different applied potentials going from OCV (blue lines) to positive applied potentials (red lines) during a forward (solid lines) and reverse scans (dashed lines) showing increased $\tilde{\nu }$_s_ (NO_2_) and $\tilde{\nu }$_as_ (NO_2_) band intensities at positive applied potentials. (d) EC‐SEIRA spectra of 100 mM 3‐NS and 100 mM TBAPF_6_ in MeCN on Pt in the presence of H_2_ while applying increasingly positive applied potentials showing decreased Pt–H band intensities at more positive applied potentials. (e) EC‐SEIRA spectra recorded of strongly surface‐bound 3‐NS_ads_ on a Pt electrode immersed in a solution of 100 mM TBAPF_6_ in MeCN in the absence of H_2_ at different applied potentials going from OCV (purple) to positive applied potentials (red). SEIRA spectra have been measured with respect to a background SEIRA spectrum of 100 mM TBAPF_6_ in MeCN on Pt in absence (b, d, and e) or presence (c) of H_2_ without (d) or with (b, c, and e) first applying +0.80 V versus OCV (also see SI: Section S9.4). Potential values indicated in (b–e) were measured versus an Ag/AgCl reference electrode. (f) Illustration of the effect on the orientation geometry of surface‐bound 3‐NS when applying a positive applied potential in the range from −0.15 V to +0.85 V versus Ag/AgCl that involves a tilting up of the –PhNO_2_ group of 3‐NS away from the surface and a rotation around the C_vinyl_─C_phenyl_ bond enabled through increased activation of the vinyl‐group.

We hypothesized that positive applied potentials and the produced H^+^ could both contribute to positively polarize the catalyst and induce a promoted adsorption of the 3‐NS substrate in a specific orientation to enhance yield and C═C hydrogenation selectivity. Density functional theory (DFT) calculations suggest that the HOMO is largely localized on the vinyl‐group making this the more electron‐rich functional group compared to the nitro‐group (Figure 3a). A more electron‐rich group could adsorb preferentially at a positively polarized electrode [23]. Prior work using structurally modified catalysts suggests that selectivity for hydrogenation of nitro‐ versus vinyl‐groups in nitrostyrene is mainly a consequence of which group preferentially adsorbs at the surface [23, 38].

We used EC‐SEIRAS to probe the interfacial interaction of 3‐NS with Pt as a function of applied potential under similar reaction conditions as used in the H‐cell setup. The symmetric and antisymmetric stretching vibrations of the NO_2_‐group in 3‐NS ($\tilde{\nu }$_s_ (NO_2_) and $\tilde{\nu }$_as_ (NO_2_)) increased in intensity with more positive applied potentials, both in the absence and presence of H_2_ (Figures 3b,c, S50, and S57). Increased SEIRAS intensities could result from increased 3‐NS concentration near the surface or from changes in catalyst morphology or 3‐NS orientation. However, enhanced $\tilde{\nu }$_s_ (NO_2_) and $\tilde{\nu }$_as_ (NO_2_) band intensities were only obtained in presence of excess 3‐NS in solution (SI: Sections S9.7–S9.9). Furthermore, post‐catalytic characterization and re‐use experiments of the Pt catalyst suggest no significant morphology changes (SI: Sections S7.1–S7.2). This suggests that the intensity increase at positive applied potentials can be predominantly attributed to a higher concentration of 3‐NS near the Pt surface. That this was also observed in absence of H_2_ suggests that HOR is not a prerequisite for this effect. Decreased Pt–H band [39, 40] intensities at ∼2050–2065 cm^−1^ suggest lowered H‐coverage at more positive applied potentials (Figure 3d and SI: Section S9.9). Combined, the changes in surface coverages could contribute to the observed higher activity at positive applied potentials.

The mechanism of enhanced 3‐ENB selectivity at more positive applied potentials was studied by probing exclusively 3‐NS that are strongly surface‐bound in the absence of H_2_ by EC‐SEIRAS (SI: Sections S9.4.2 and S9.8). Under these conditions, the $\tilde{\nu }$_s_ (NO_2_) intensity increased while the $\tilde{\nu }$_as_ (NO_2_) intensity decreased and both bands blue‐shifted with more positive applied potentials (Figures 3e and S54). The observation of both $\tilde{\nu }$_s_ (NO_2_) and $\tilde{\nu }$_as_ (NO_2_) combined with surface selection rules [41, 42] and prior work [43, 44] suggest that 3‐NS adsorbs in a tilted‐parallel configuration on Pt, in which the vinyl‐group of 3‐NS predominantly interacts with the surface (Figure 3f and SI: Section S9.6). The observed changes in $\tilde{\nu }$_s_ (NO_2_) and $\tilde{\nu }$_as_ (NO_2_) band intensities and frequency shifts with applied potential imply that the orientation of the ensemble of strongly‐bound 3‐NS at the surface change with applied potential. Specifically, the tilted‐parallel adsorbed 3‐NS likely tilts up further from the surface through enhanced activation of the vinyl‐group toward a di‐sigma‐type binding (SI: Section S9.8.3). This enables rotation around the C_vinyl_─C_phenyl_ bond in 3‐NS and thereby reduces the interaction of the NO_2_‐ group with the surface. The observed orientation change reflects a stronger C═C bond activation, which also implies a larger affinity for the electron‐rich vinyl‐ over electron‐deficient NO_2_‐ groups at positive applied potential. The stronger activation of the C═C bond of 3‐NS at positive applied potential is consistent with the observed higher 3‐ENB over 3‐VA formation. Interestingly, these orientation changes and our observed chemoselective hydrogenation to 3‐ENB in a positive external field are different to a recent report that attributed a selective 3‐VA production on curved interior catalyst surfaces to electric field effects [29]. We speculate that these differences are due to confinement effects in the prior report [45, 46].

A stronger affinity of Pt for the electron‐rich C═C over electron‐deficient NO_2_‐ groups in 3‐NS at positive applied potentials can explain 3‐ENB over 3‐VA formation as well as the lack of fully hydrogenated 3‐EA under these conditions. Product formation as a function of time measured at OCV suggested that 3‐ENB is both a product and an intermediate and can be further hydrogenated to 3‐EAOH and 3‐EA (Figures S12 and S17c). At positive applied potential, the affinity of Pt for 3‐ENB that contains the electron‐deficient NO_2_‐group could be decreased to such an extent that desorption is favored over further hydrogenation. This is consistent with our observations when using 3‐ENB as substrate for hydrogenation, where 3‐EAOH and 3‐EA were formed at OCV but not at −0.2 V (Table S16).

**FIGURE 4:**
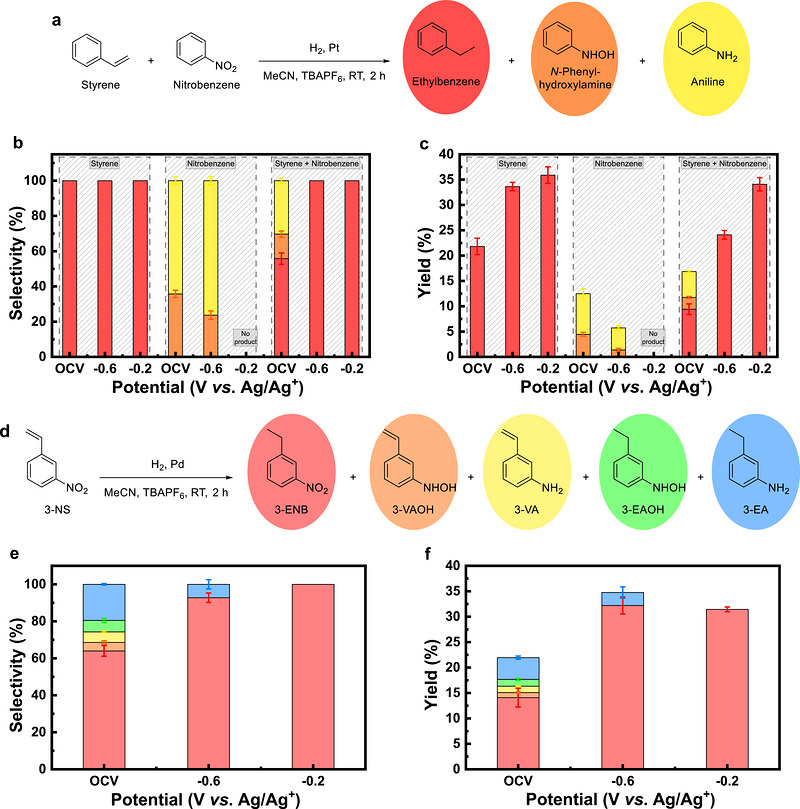
(a) Catalytic hydrogenation of styrene, nitrobenzene, or a competitive hydrogenation of styrene and nitrobenzene using Pt as catalyst and associated (b) selectivity and (c) yield as a function of applied potential. Colored bars correspond to observed products: Ethylbenzene (red), *N*‐phenylhydroxylamine (orange), and aniline (yellow). (d) 3‐NS hydrogenation using Pd as catalyst and associated (e) selectivity and (f) yield as a function of applied potential. Colored bars correspond to the observed products: 3‐ENB (red), 3‐VAOH (orange), 3‐VA (yellow), 3‐EAOH (green), and 3‐EA (blue). Mean and standard deviation shown for each set of conditions in (b), (c), (e), and (f) were derived from three independent replicate experiments.

The promotional effects on selectivity and activity from a stronger activation of electron‐rich groups over electron‐deficient and an increased surface concentration of the substrate, respectively, should not be limited to 3‐NS and Pt. We hence tested the generality of the observed catalytic effects with other substrates and catalytic materials. Indeed, styrene and nitrobenzene showed higher and lower yields at more positive applied potentials, respectively (Figure 4a–c and Table S17). This is also consistent with our results for 3‐ENB hydrogenation discussed above (Table S16). Higher yields for C═C reduction at positive applied potentials were also observed for 3‐vinylanisole, 3‐bromostyrene, 4‐vinylbenzoic acid, 2‐allylbenzaldehyde, and 1‐allyl‐4‐methoxybenzene, while the methoxy‐, bromo‐, carboxylic acid‐, and aldehyde‐groups were not reduced (Table S18). Consistent with the trend observed with 3‐NS or 3‐ENB, NO_2_‐ hydrogenation was only observed at OCV in the hydrogenation of 5‐nitroindole but not at positive applied potential (Table S18). Similarly, yield and selectivity to hydrogenate the more electron‐rich C═C bond versus C═O in *trans*‐cinnamaldehyde [26] as well as in 4‐pentenal increased at more positive applied potentials (Figures S70 and S72). Similarly to when using Pt as catalyst, Pd showed enhanced yield and selectivity for 3‐ENB at more positive applied potentials (Figure 4d–f). We also tested Cu, cobalt phosphide, and cobalt sulfide as alternative catalysts, but could not observe hydrogenation activity under our conditions at either OCV or positive applied potential (SI: Section S10.2).

Overall, our results from EC‐SEIRAS and DFT calculations suggest that the higher activity and selectivity for 3‐ENB production at positive applied potential are associated with a higher surface concentration of 3‐NS and an enhanced affinity of Pt for the electron‐rich C═C group over the electron‐deficient NO_2_‐group. These effects extend beyond the primary system of 3‐NS hydrogenation considered herein and also apply to other substrates and catalysts.

### 2.4 Effects of Electrode Polarization on Surface Species Concentration

Hydrogenation reactions over metal catalysts are commonly thought to occur via the Horiuti–Polanyi mechanism [47, 48, 49]. H_2_ dissociates on the catalyst and the resulting surface H* species add to the adsorbed substrate in a stepwise fashion [23, 25]. To test possible changes in mechanism with applied potential, we carried out kinetic experiments. We focus our analysis on possible changes in mechanism for the formation of 3‐ENB, as 3‐ENB is the sole product observed at −0.2 V. Accordingly, we compared the amount of 3‐ENB produced at varied reactant concentrations and temperatures below. While reaction orders cannot be accurately determined with the data in hand since H_2_ was controlled by flow rate rather than partial pressure, important trends with applied potential can be identified.

**FIGURE 5:**
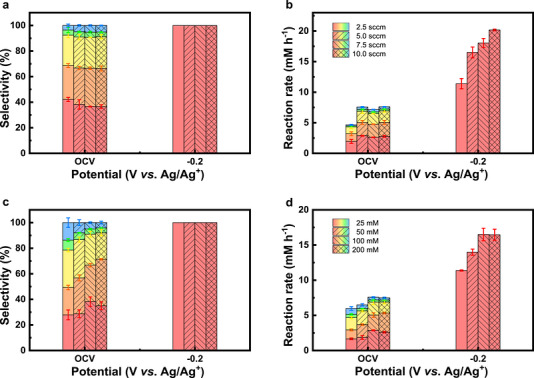
(a) Selectivity and (b) reaction rate of the 3‐NS hydrogenation with variation of the hydrogen flow rate. (c) Selectivity and (d) reaction rate of the 3‐NS hydrogenation with variation of the 3‐NS concentration in solution. Colored bars correspond to the observed products: 3‐NS (red), 3‐VAOH (orange), 3‐VA (yellow), 3‐EAOH (green), and 3‐EA (blue). Mean and standard deviation shown for each set of conditions were derived from three independent replicate experiments. Sccm: standard cubic centimeters per minute.

Variation of H_2_ flow rate had a larger effect on the production rate of 3‐ENB at −0.2 V than at OCV (Figure 5a,b). While at OCV there was no further enhancement for 3‐ENB production above 5 sccm, the 3‐ENB production continued to increase at higher flow rates at −0.2 V. This suggests that the concentration of H* is more rate‐limiting at −0.2 V, consistent with the idea that HOR provides a competing pathway to hydrogenation for H* at −0.2 V but not at OCV (Table S20). The competing HOR pathway combined with possibly reduced binding energies of H* at higher pH [50, 51, 52] (though, there are conflicting reports on this [40]) could reduce the H* surface concentration, consistent with EC‐SEIRAS data (Figure 3d). This could optimize the H* and 3‐NS surface concentrations and thereby enhance the hydrogenation reaction rate at positive applied potential. The lower yield observed at very positive applied potentials above −0.2 V (Figure 1c) could be associated with a H* coverage that is too low. Only a small increase in the portion of overhydrogenated 3‐EA was observed at higher H_2_ flow rates at OCV, likely due to the low conversion conditions under which these kinetic experiments needed to be run and surface saturation with hydrogen at higher flow rates (Figure 5a,b). At −0.2 V, no 3‐EA was observed even at high H_2_ flow rate consistent with the idea that the electron‐deficient 3‐ENB desorbs rather than being further hydrogenated at the positively polarized Pt.

Variation of the 3‐NS concentration had a larger effect on 3‐ENB production rate at OCV than at −0.2 V (Figure 5c,d). The 3‐ENB production increased with higher 3‐NS concentration up to 100 mM by a factor of 1.73 or a factor of 1.45 at OCV or −0.2 V, respectively, and then stayed constant. This suggests that the 3‐NS surface concentration is more rate‐limiting at OCV than at −0.2 V. This seems consistent with our EC‐SEIRAS results suggesting higher 3‐NS surface concentration at positive applied potentials, which could lead to lower sensitivity to 3‐NS concentration changes in solution. The observation of a smaller electric charge passed at higher 3‐NS concentration (Table S21) suggests that HOR is dependent on 3‐NS surface concentration with less HOR occurring at higher 3‐NS concentration. This implies that 3‐NS and H* adsorb competitively on Pt, consistent with prior reports [53].

**FIGURE 6:**
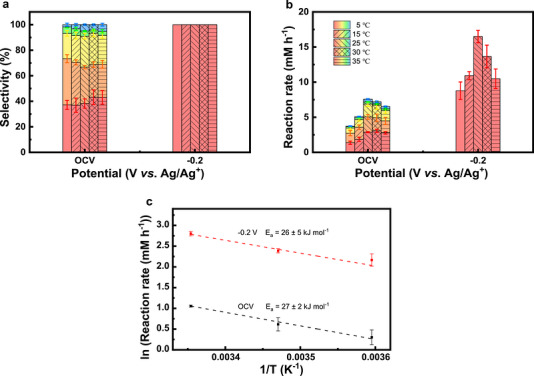
(a) Selectivity and (b) reaction rate of the 3‐NS hydrogenation at varied temperature, and the (c) apparent activation energy of the 3‐NS hydrogenation to 3‐ENB estimated from these data. Colored bars in (a) and (b) correspond to the observed products: 3‐NS (red), 3‐VAOH (orange), 3‐VA (yellow), 3‐EAOH (green), and 3‐EA (blue). Mean and standard deviation shown for each set of conditions were derived from three independent replicate experiments.

The reaction rate to 3‐ENB showed a non‐linear relationship with varied temperature at both OCV and at −0.2 V (Figure 6). At low temperatures of 5°C–25°C, the reaction rate to 3‐ENB increased both at OCV and at −0.2 V. At higher temperatures of 25°C–35°C, the rate was nearly constant at OCV and decreased at −0.2 V. We approximated apparent activation energies *E*_app_ at low temperatures using the 3‐ENB production rates and the Arrhenius equation as 27 ± 2 and 26 ± 5 kJ mol^−1^ at OCV and −0.2 V, respectively (SI: Section S11.4). This implies no change in *E*_app_ at lower temperatures with applied potential within error. This is perhaps consistent with a relatively small rate decrease observed with increasing TBAPF_6_ electrolyte strength at both OCV and −0.2 V (SI: Section S11.5). This observation suggests sensitivity to the interfacial potential gradient but not a pronounced rate increase. The latter would be expected for a rate‐determining interfacial charge transfer step that is influenced by the electric field gradient, which would then translate into different activation barriers at OCV and at −0.2 V [9]. The constant or decreasing rates at high temperatures could be explained with a change of mechanism or adsorption/desorption equilibria of 3‐NS and/or H*. At −0.2 V, trends observed with the positive charge passed and solution ^MeCN^pH with temperature (Table S22) suggest that the HOR reaction is temperature‐sensitive and possibly limited by a lower H_2_ solubility or H^*^ desorption at higher temperatures. Based on this and trends observed with varied H_2_‐flow rate or 3‐NS concentration, the reaction at OCV is likely limited by 3‐NS surface concentration and the reaction at −0.2 V by H* and possibly also 3‐NS surface concentration at high temperature. Overall, our data imply that the applied potential influences the 3‐NS hydrogenation activity primarily via changes in the surface coverage of reactants rather than the activation energy.

**FIGURE 7:**
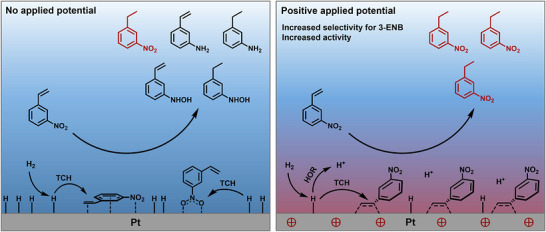
Schematic illustration of the effects of a positive applied potential on the thermocatalytic hydrogenation (TCH) of 3‐NS with H_2_. At OCV (no applied potential), the Pt surface has higher coverage with hydrogen and lower coverage with 3‐NS. Multiple different orientations of 3‐NS are possible leading to multiple products. With positive applied potential, the orientation of 3‐NS changes and the vinyl‐group is more activated and preferentially hydrogenated. An enhanced affinity for the electron‐rich vinyl‐group and a reduced affinity for the electron‐deficient NO_2_‐group also result in a lower barrier for desorption of 3‐ENB over further hydrogenation at positive applied potential. Furthermore, surface coverage of hydrogen is lower and that of 3‐NS is higher, in part, due to a competing pathway for surface hydrogen: hydrogen oxidation reaction (HOR). While the apparent activation energy for 3‐ENB formation is similar at OCV and positive applied potential, the surface concentration, adsorption geometry, and adsorption properties of adsorbates are changed. These effects lead to enhanced activity and chemoselectivity for the thermal 3‐NS hydrogenation to 3‐ENB.

Figure 7 illustrates the solid/solution interface of the Pt catalyst for 3‐NS hydrogenation in absence and presence of a positive applied potential. At OCV, the Pt surface is largely covered with H* that competes for adsorption sites with 3‐NS and thereby limits 3‐ENB reaction rates. H* addition to vinyl‐ or nitro‐groups results in five different hydrogenation products. If the Pt catalyst is positively polarized, HOR is a competing pathway for H* to thermal hydrogenation. The positive polarization of the Pt catalyst and the lower H* surface concentration resulting from HOR allow larger 3‐NS surface concentrations. This increases activity. In addition, the positive polarization of the electrode induced by the applied potential and the H^+^ in solution influences the binding and orientation of 3‐NS at the surface, which leads to a selective C═C over NO_2_ hydrogenation. The enhanced affinity of Pt for C═C groups and the reduced affinity for NO_2_‐groups at positive applied potentials also results in a preferred 3‐ENB desorption over overhydrogenation to 3‐EA. Enhanced performance can be obtained reversibly by the applied potential as shown by consecutive OCV‐applied potential‐OCV experiments and under mild conditions without overhydrogenation, which highlights the advantages of the EPOC method over conventional approaches using structurally modified catalysts (Table S3).

## 3 Conclusion

This work demonstrates a powerful promotional effect of an applied electrochemical potential on not only selectivity but also activity of thermocatalytic hydrogenation. At positive applied potentials full chemoselectivity and six‐times enhanced activity for 3‐ENB formation from the hydrogenation of 3‐NS was reached, while only <40% selectivity for 3‐ENB was obtained at OCV. Our data show that this phenomenon is due to an electrochemically promoted thermal reaction rather than an electrocatalytic hydrogenation or a permanent catalyst change. The concurrent hydrogen oxidation reaction resulted in an acidic environment that can cause the observed effects, but only in part, which highlights the privileged role of the applied potential. EC‐SEIRAS combined with detailed mechanistic studies and DFT suggested that the obtained promotional effect on selectivity and activity originates from a change in surface concentrations of 3‐NS and hydrogen as well as a change in adsorption geometry of 3‐NS and an enhanced affinity of Pt for electron‐rich over electron‐deficient groups at positive potentials. This strategy overcomes a key limitation in conventional thermal catalysis by enabling tunable reaction outcomes without catalyst modification or activity loss. On a conceptual level, we showed that concentration and orientation of surface adsorbates can be influenced by an applied potential. Given the fundamental importance of these parameters to heterogeneous catalysis, the approach presented herein may provide exquisite control over a range of chemical transformations beyond hydrogenations. Furthermore, the obtained chemoselectivity minimizes purification steps and thereby highlights the advantages of the approach for more sustainable chemical processes.

## Author Contributions

**Yunfei Jiao**: investigation, validation, formal analysis, visualization, writing – original draft, writing – review and editing. **Martina A. Pogany**: investigation, validation, formal analysis, visualization, writing – original draft, writing – review and editing. **Murielle F. Delley**: conceptualization, funding acquisition, project administration, supervision, formal analysis, writing – original draft, writing – review and editing.

## Conflicts of Interest

The authors declare no conflicts of interest.

## Supporting information



Detailed experimental procedures, replicate catalytic experiments data, systematic control experiments, pH value analysis, electrochemical experiments, isotopic labelling experiments, SEM, EDX, XPS data, detailed EC‐SEIRAS experiments and analysis, and supplementary discussions.
**Supporting File 1**: anie73093‐sup‐0001‐SuppMat.pdf.

## Data Availability

The data that supports the findings of this study are available in the Supporting Information of this article.

## References

[1] E. Roduner , “Understanding Catalysis,” Chemical Society Reviews 43 (2014): 8226–8239, 10.1039/C4CS00210E. 25311156

[2] C. G. Vayenas , S. Bebelis , and S. Ladas , “Dependence of Catalytic Rates on Catalyst Work Function,” Nature 343 (1990): 625–627, 10.1038/343625a0.

[3] P. Vernoux , L. Lizarraga , M. N. Tsampas , et al., “Ionically Conducting Ceramics as Active Catalyst Supports,” Chemical Reviews 113 (2013): 8192–8260, 10.1021/cr4000336. 23826949

[4] S. Shaik , R. Ramanan , D. Danovich , and D. Mandal , “Structure and Reactivity/Selectivity Control by Oriented‐External Electric Fields,” Chemical Society Reviews 47 (2018): 5125–5145, 10.1039/C8CS00354H. 29979456

[5] T.‐C. Chang Chien and M. F. Delley , “Disentangling Chemical Interaction and Electric Fields at Electrochemical Interfaces,” Journal of Physical Chemistry C 129 (2025): 999–1012, 10.1021/acs.jpcc.4c07553.

[6] J. Nicole , D. Tsiplakides , C. Pliangos , X. E. Verykios , C. Comninellis , and C. G. Vayenas , “Electrochemical Promotion and Metal–Support Interactions,” Journal of Catalysis 204 (2001): 23–34, 10.1006/jcat.2001.3360.

[7] Q. Shen , H. Yang , K. Zhao , et al., “Unraveling Intrinsic Electronic Factors in Thermocatalytic (Hemi‐)Hydrogenation of Ethylene and Acetylene With Electric Polarization,” ACS Catalysis 13 (2023): 14570–14579, 10.1021/acscatal.3c04258.

[8] C. W. Lim , M. J. Hulsey , and N. Yan , “Non‐Faradaic Promotion of Ethylene Hydrogenation Under Oscillating Potentials,” JACS Au 1 (2021): 536–542, 10.1021/jacsau.1c00044. 34467316 PMC8395646

[9] K. S. Westendorff , M. J. Hülsey , T. S. Wesley , Y. Román‐Leshkov , and Y. Surendranath , “Electrically Driven Proton Transfer Promotes Brønsted Acid Catalysis by Orders of Magnitude,” Science 383 (2024): 757–763, 10.1126/science.adk4902. 38359117

[10] J. Ryu and Y. Surendranath , “Tracking Electrical Fields at the Pt/H_2_O Interface During Hydrogen Catalysis,” Journal of the American Chemical Society 141 (2019): 15524–15531, 10.1021/jacs.9b05148. 31433173 PMC6777043

[11] J. Ryu and Y. Surendranath , “Polarization‐Induced Local pH Swing Promotes Pd‐Catalyzed CO_2_ Hydrogenation,” Journal of the American Chemical Society 142 (2020): 13384–13390, 10.1021/jacs.0c01123. 32628840

[12] J. Sanabria‐Chinchilla , K. Asazawa , T. Sakamoto , K. Yamada , H. Tanaka , and P. Strasser , “Noble Metal‐Free Hydrazine Fuel Cell Catalysts: EPOC Effect in Competing Chemical and Electrochemical Reaction Pathways,” Journal of the American Chemical Society 133 (2011): 5425–5431, 10.1021/ja111160r. 21425793

[13] S. G. Neophytides , D. Tsiplakides , P. Stonehart , M. Jaksic , and C. G. Vayenas , “Non‐Faradaic Electrochemical Modification of the Catalytic Activity of Pt for H_2_ Oxidation in Aqueous Alkaline media,” Journal of Physical Chemistry 100 (1996): 14803–14814, 10.1021/jp960971u.

[14] M. Athanasiou , B. Hasa , J. Vakros , L. Sygellou , and A. Katsaounis , “Electrochemical Promotion of Carbon Supported Pt, Rh and Pd Catalysts for H_2_ Oxidation in Aqueous Alkaline Media,” Journal of Chemical Technology & Biotechnology 93 (2018): 1542–1548, 10.1002/jctb.5564.

[15] X. Qi , T. Shinagawa , X. Lu , Y. Yui , M. Ibe , and K. Takanabe , “Surface Coverage Control for Dramatic Enhancement of Thermal CO Oxidation by Precise Potential Tuning of Metal Supported Catalysts,” Chemical Science 13 (2022): 9774–9783, 10.1039/D2SC03145K. 36091892 PMC9400665

[16] A. A. Khechfe , M. M. Sullivan , D. Zagoraios , A. Katsaounis , C. G. Vayenas , and Y. Román‐Leshkov , “Non‐Faradaic Electrochemical Promotion of Brønsted Acid‐Catalyzed Dehydration Reactions Over Molybdenum Oxide,” ACS Catalysis 12 (2022): 906–912, 10.1021/acscatal.1c04885.

[17] C. F. Gorin , E. S. Beh , and M. W. Kanan , “An Electric Field‐Induced Change in the Selectivity of a Metal Oxide‐Catalyzed Epoxide Rearrangement,” Journal of the American Chemical Society 134 (2012): 186–189, 10.1021/ja210365j. 22191979

[18] M. Akamatsu , N. Sakai , and S. Matile , “Electric‐Field‐Assisted Anion–π Catalysis,” Journal of the American Chemical Society 139 (2017): 6558–6561, 10.1021/jacs.7b02421. 28481534

[19] M. Á. Gutiérrez López , R. Ali , M.‐L. Tan , N. Sakai , T. Wirth , and S. Matile , “Electric Field‐Assisted Anion‐π Catalysis on Carbon Nanotubes in Electrochemical Microfluidic Devices,” Science Advances 9 (2023): eadj5502. 37824606 10.1126/sciadv.adj5502 PMC10569703

[20] F. Cai , D. Gao , H. Zhou , et al., “Electrochemical Promotion of Catalysis Over Pd Nanoparticles for CO_2_ Reduction,” Chemical Science 8 (2017): 2569–2573, 10.1039/C6SC04966D. 28553489 PMC5431665

[21] C. F. Gorin , E. S. Beh , Q. M. Bui , G. R. Dick , and M. W. Kanan , “Interfacial Electric Field Effects on a Carbene Reaction Catalyzed by Rh Porphyrins,” Journal of the American Chemical Society 135 (2013): 11257–11265, 10.1021/ja404394z. 23837635

[22] F. Che , J. T. Gray , S. Ha , and J.‐S. McEwen , “Improving Ni Catalysts Using Electric Fields: A DFT and Experimental Study of the Methane Steam Reforming Reaction,” ACS Catalysis 7 (2016): 551–562, 10.1021/acscatal.6b02318.

[23] L. Zhang , M. Zhou , A. Wang , and T. Zhang , “Selective Hydrogenation Over Supported Metal Catalysts: From Nanoparticles to Single Atoms,” Chemical Reviews 120 (2020): 683–733, 10.1021/acs.chemrev.9b00230. 31549814

[24] J. Song , Z.‐F. Huang , L. Pan , et al., “Review on Selective Hydrogenation of Nitroarene by Catalytic, Photocatalytic and Electrocatalytic Reactions,” Applied Catalysis B: Environmental 227 (2018): 386–408, 10.1016/j.apcatb.2018.01.052.

[25] F. Zaera , “The Surface Chemistry of Metal‐Based Hydrogenation Catalysis,” ACS Catalysis 7 (2017): 4947–4967, 10.1021/acscatal.7b01368.

[26] X. Lan and T. Wang , “Highly Selective Catalysts for the Hydrogenation of Unsaturated Aldehydes: A Review,” ACS Catalysis 10 (2020): 2764–2790, 10.1021/acscatal.9b04331.

[27] C. Pei , S. Chen , D. Fu , Z. J. Zhao , and J. Gong , “Structured Catalysts and Catalytic Processes: Transport and Reaction Perspectives,” Chemical Reviews 124 (2024): 2955–3012, 10.1021/acs.chemrev.3c00081. 38478971

[28] X. Wang , X. Liang , P. Geng , and Q. Li , “Recent Advances in Selective Hydrogenation of Cinnamaldehyde Over Supported Metal‐Based Catalysts,” ACS Catalysis 10 (2020): 2395–2412, 10.1021/acscatal.9b05031.

[29] Y. Zhang , Q. Cheng , S. Xiong , et al., “Nanotubular Curvature‐Induced Internal Electrostatic Field Realizing Chemoselective Hydrogenation,” ACS Catalysis 15 (2025): 13085–13096, 10.1021/acscatal.5c01993.

[30] M. Luneau , J. S. Lim , D. A. Patel , E. C. H. Sykes , C. M. Friend , and P. Sautet , “Guidelines to Achieving High Selectivity for the Hydrogenation of α,β‐Unsaturated Aldehydes With Bimetallic and Dilute Alloy Catalysts: A Review,” Chemical Reviews 120 (2020): 12834–12872, 10.1021/acs.chemrev.0c00582. 33006894

[31] E. J. Garcia‐Suarez and A. Riisager , in Sustainable Catalysis (Wiley, 2018), 1–34.

[32] N. Elgrishi , K. J. Rountree , B. D. McCarthy , E. S. Rountree , T. T. Eisenhart , and J. L. Dempsey , “A Practical Beginner's Guide to Cyclic Voltammetry,” Journal of Chemical Education 95 (2017): 197–206, 10.1021/acs.jchemed.7b00361.

[33] W. Zhang , W. Zhang , J. Tan , D. Pan , Y. Tang , and Q. Gao , “Alloying Promotion of Pd‐Based Metallenes in Electrocatalytic Hydrogenation of Functionalized Nitroarenes,” Journal of Materials Chemistry A 11 (2023): 7505–7512, 10.1039/D2TA10021E.

[34] J. Tan , J. Shao , Y. Shi , W. Zhang , and Q. Gao , “Selective Electrocatalytic Hydrogenation of Nitroarenes on Interlayer‐Expanded MoS_2_ ,” ACS Sustainable Chemistry & Engineering 10 (2022): 13525–13533, 10.1021/acssuschemeng.2c04667.

[35] J. Ryu , A. Wuttig , and Y. Surendranath , “Quantification of Interfacial pH Variation at Molecular Length Scales Using a Concurrent Non‐Faradaic Reaction,” Angewandte Chemie International Edition 57 (2018): 9300–9304, 10.1002/anie.201802756. 29766624

[36] M. J. Beier , J.‐M. Andanson , and A. Baiker , “Tuning the Chemoselective Hydrogenation of Nitrostyrenes Catalyzed by Ionic Liquid‐Supported Platinum Nanoparticles,” ACS Catalysis 2 (2012): 2587–2595, 10.1021/cs300529y.

[37] T. S. Wesley , Y. Roman‐Leshkov , and Y. Surendranath , “Spontaneous Electric Fields Play a Key Role in Thermochemical Catalysis at Metal–Liquid Interfaces,” ACS Central Science 7 (2021): 1045–1055, 10.1021/acscentsci.1c00293. 34235265 PMC8228591

[38] Y. Ren , Y. Yang , and M. Wei , “Recent Advances on Heterogeneous Non‐Noble Metal Catalysts Toward Selective Hydrogenation Reactions,” ACS Catalysis 13 (2023): 8902–8924, 10.1021/acscatal.3c01442.

[39] K. Kunimatsu , H. Uchida , M. Osawa , and M. Watanabe , “In Situ Infrared Spectroscopic and Electrochemical Study of Hydrogen Electro‐Oxidation on Pt Electrode in Sulfuric Acid,” Journal of Electroanalytical Chemistry 587 (2006): 299–307, 10.1016/j.jelechem.2005.11.026.

[40] S. Zhu , X. Qin , Y. Yao , and M. Shao , “pH‐Dependent Hydrogen and Water Binding Energies on Platinum Surfaces as Directly Probed Through Surface‐Enhanced Infrared Absorption Spectroscopy,” Journal of the American Chemical Society 142 (2020): 8748–8754, 10.1021/jacs.0c01104. 32306730

[41] M. Osawa , “Dynamic Processes in Electrochemical Reactions Studied by Surface‐Enhanced Infrared Absorption Spectroscopy (SEIRAS),” Bulletin of the Chemical Society of Japan 70 (1997): 2861–2880, 10.1246/bcsj.70.2861.

[42] R. Kas , O. Ayemoba , N. J. Firet , J. Middelkoop , W. A. Smith , and A. Cuesta , “In‐Situ Infrared Spectroscopy Applied to the Study of the Electrocatalytic Reduction of CO_2_: Theory, Practice and Challenges,” ChemPhysChem 20 (2019): 2904–2925, 10.1002/cphc.201900533. 31441195

[43] L. Zhang , X.‐M. Cao , and P. Hu , “Insight into Chemoselectivity of Nitroarene Hydrogenation: A DFT‐D3 Study of Nitroarene Adsorption on Metal Surfaces Under the Realistic Reaction Conditions,” Applied Surface Science 392 (2017): 456–471, 10.1016/j.apsusc.2016.09.031.

[44] L. Zhang , Z.‐J. Shao , X.‐M. Cao , and P. Hu , “Insights into Different Products of Nitrosobenzene and Nitrobenzene Hydrogenation on Pd(111) Under Realistic Reaction Conditions,” Journal of Physical Chemistry C 122 (2018): 20337–20350, 10.1021/acs.jpcc.8b05364.

[45] F. Ziegler , H. Kraus , M. J. Benedikter , et al., “Confinement Effects for Efficient Macrocyclization Reactions With Supported Cationic Molybdenum Imido Alkylidene *N*‐Heterocyclic Carbene Complexes,” ACS Catalysis 11 (2021): 11570–11578, 10.1021/acscatal.1c03057.

[46] P. Probst , M. Lindemann , J. R. Bruckner , et al., “Ring‐Expansion Metathesis Polymerization Under Confinement,” Journal of the American Chemical Society 147 (2025): 8741–8750, 10.1021/jacs.4c18171. 40009038

[47] I. Horiuti and M. Polanyi , “Exchange Reactions of Hydrogen on Metallic Catalysts,” Transactions of the Faraday Society 30 (1934): 1164, 10.1039/tf9343001164.

[48] J. Horiuti and M. Polanyi , “Direct Introduction of Deuterium into Benzene,” Nature 134 (1934): 847–847, 10.1038/134847a0.

[49] B. Mattson , W. Foster , J. Greimann , et al., “Heterogeneous Catalysis: The Horiuti–Polanyi Mechanism and Alkene Hydrogenation,” Journal of Chemical Education 90 (2013): 613–619.

[50] W. Sheng , Z. Zhuang , M. Gao , J. Zheng , J. G. Chen , and Y. Yan , “Correlating Hydrogen Oxidation and Evolution Activity on Platinum at Different pH With Measured Hydrogen Binding Energy,” Nature Communications 6 (2015): 5848, 10.1038/ncomms6848. 25569511

[51] T. Cheng , L. Wang , B. V. Merinov , and W. A. Goddard 3rd , “Explanation of Dramatic pH‐Dependence of Hydrogen Binding on Noble Metal Electrode: Greatly Weakened Water Adsorption at High pH,” Journal of the American Chemical Society 140 (2018): 7787–7790, 10.1021/jacs.8b04006. 29792321

[52] N. Singh , M.‐S. Lee , S. A. Akhade , et al., “Impact of pH on Aqueous‐Phase Phenol Hydrogenation Catalyzed by Carbon‐Supported Pt and Rh,” ACS Catalysis 9 (2019): 1120–1128, 10.1021/acscatal.8b04039.

[53] U. Sanyal , S. F. Yuk , K. Koh , et al., “Hydrogen Bonding Enhances the Electrochemical Hydrogenation of Benzaldehyde in the Aqueous Phase,” Angewandte Chemie International Edition 60 (2021): 290–296, 10.1002/anie.202008178. 32770641 PMC7821193

