# Supplementary material for: Positive Electrochemical Potentials Induce Enhanced Chemoselectivity and Activity in the Thermocatalytic Hydrogenation of 3‐Nitrostyrene
Source: Angew Chem Int Ed Engl. 2026 Jun 8;65(33):e5569475. doi: 10.1002/anie.5569475 (PMC13452539; doi:10.1002/anie.5569475)
Supplement: Supplementary file 1 [file ANIE-65-e5569475-s001.pdf]

Supporting Information for

Positive Electrochemical Potentials Induce Enhanced Chemoselectivity and Activity in the Thermocatalytic Hydrogenation of 3-Nitrostyrene

Yunfei Jiao [a], Martina A. Pogany [a], Murielle F. Delley [a, *]

[a] Department of Chemistry, University of Basel, Mattenstrasse 22, 4058 Basel, Switzerland

[*] E-mail: murielle.delley@unibas.ch

Table of Contents

1. Chemicals and materials S4

2. General methods S5

3. Experimental methods S6

3.1 Preparation of the Pt electrode S6

3.2 General reaction setup and reaction conditions S6

3.3 Thermal 3-NS hydrogenation at open circuit voltage S6

3.4 Thermal 3-NS hydrogenation at different applied potentials S7

3.5 EC-SEIRAS measurements S7

4. Product identification and reaction pathway analysis S8

4.1 Analysis of products observed during 3-NS hydrogenation catalysis: 3-ENB, 3-VA, and 3-EA S8

4.2 Analysis of additional products observed during 3-NS hydrogenation catalysis: 3-VAOH and 3-EAOH S12

4.3 Reaction products and reaction pathway for 3-NS hydrogenation catalysis S17

4.4 Analysis of products or reactions at the counter electrode S18

5. Thermal 3-NS hydrogenation as a function of applied potential S21

6. Testing the possibility of electrocatalytic 3-NS hydrogenation under the conditions studied herein S25

7. Testing catalyst stability when a positive potential is applied S27

7.1 Testing if original catalytic performance at OCV is recovered after an applied potential S27

7.2 Characterization of the as-prepared catalyst and the catalyst after reaction S28

7.3 Testing the contribution to catalysis from possible leached components of the catalyst S31

8. The effect of the concurrent hydrogen oxidation reaction S32

8.1 Supporting data for experiments involving the addition of HPF_6 .... S32

8.2 Isotopic labelling experiments with D_2O S35
8.3 The effect of H^+ from the Nafion-117 membrane S43
9. Mechanistic investigation using EC-SEIRAS S45
9.1 General considerations S45
9.2 Preparation of the gold film and the Pt overlayer film S46
9.3 Characterization of the prepared Pt film on Au S47
9.3.1 Characterization by CV S47
9.3.2 Determination of the electrochemically active surface area, roughness, and thickness S48
9.3.3 Characterization by CO adsorption S48
9.4 Experimental procedures for EC-SEIRAS measurements involving 3-NS S49
9.4.1 EC-SEIRAS experiments of 3-NS on Pt in the absence of H_2 as a function of applied potential S49
9.4.2 EC-SEIRAS experiments of 3-NS on Pt in the absence of H_2 as a function of applied potential, involving strongly surface-bound 3-NS only S49
9.4.3 EC-SEIRAS experiments of 3-NS on Pt in the presence of H_2 as a function of applied potential S51
9.4.4 EC-SEIRAS experiments of 3-NS on Pt in the presence of H_2 as a function of time, involving strongly surface-bound 3-NS only S53
9.5 Band assignments S54
9.6 Implications from observed SEIRAS bands associated with 3-NS from surface selection rules S58
9.7 EC-SEIRAS data of 3-NS on Pt in the absence of H_2 as a function of applied potential S60
9.7.1 Forward scan from OCV to more positive applied potentials S60
9.7.2 Reverse scan from more positive applied potentials to OCV S62
9.7.3 Discussion S64
9.8 EC-SEIRAS data of 3-NS on Pt in the absence of H_2 as a function of applied potential, involving strongly surface-bound 3-NS only S66
9.8.1 Forward scan from OCV to more positive applied potentials S66
9.8.2 Reverse scan from more positive applied potentials to OCV S68
9.8.3 Discussion S69
9.9 EC-SEIRAS data of 3-NS on Pt in the presence of H_2 as a function of applied potential S72
9.9.1 Forward scan from OCV to more positive applied potentials S72
9.9.2 Reverse scan from more positive applied potentials to OCV S76
9.9.3 Discussion S80

9.10 EC-SEIRAS data of 3-NS on Pt in presence of H_2 at OCV monitored as a function of time, involving strongly surface-bound 3-NS only S82

9.11 Conclusions from EC-SEIRAS studies and relevance of these studies in the context of standard H-cell experiments of catalytic 3-NS hydrogenation S84

10. Supplementary data on the catalytic hydrogenation of other substrates and using other catalysts S85

10.1 Testing catalytic hydrogenation of other substrates as a function of applied potential S85

10.2 Testing catalytic hydrogenation of 3-NS as a function of applied potential using other catalysts S90

11. Kinetic experiments S94

11.1 Variation of the hydrogen flow rate S94

11.2 Variation of the 3-NS concentration S97

11.3 Variation of the reaction temperature S99

11.4 Estimation of activation energy S102

11.5 Variation of the $TBAPF_6$ concentration S103

References S106

1. Chemicals and materials

Tetrabutylammonium hexafluorophosphate ($TBAPF_6$, ≥ 99.0%), potassium chloride (KCl, ≥ 99.0%), silver nitrate ($AgNO_3$, ≥ 99.999%), tetrabutylammonium perchlorate (TBAP, ≥ 99.0%), 3-ethylaniline (3-EA, 98%), styrene (≥ 99.0%), nitrobenzene (≥ 99.0%), *N*-phenylhydroxylamine (≥ 95.0%), hydrocinnamyl alcohol (HCOL, 98%), tetrabutylammonium fluoride hydrate ($TBAF \cdot xH_2O$, 98%), potassium hexacyanoferrate(III) ($K_3[Fe(CN)_6]$, ≥ 99.0%), sodium acetate (CH_3COONa, ≥ 99.0%), thiourea (≥ 99.0%), sodium acetate (CH_3COONa, ≥ 99.0%), 3-vinylanisole (97%) and 5-nitroindole (98%) were purchased from Sigma-Aldrich. 3-Nitrostyrene (3-NS, 97%), 1,3,5-trimethoxybenzene (TMB, 99%), ethylbenzene (99.8%), aniline (99.8%), *trans*-cinnamaldehyde (CAL, 99%), hydrocinnamaldehyde (HCAL, 95%), cinnamyl alcohol (COL, 98% *trans*-), hexafluorophosphoric acid, (HPF_6, 60 wt% solution in water), palladium(II) chloride ($PdCl_2$, 99.999%), perchloric acid ($HClO_4$, 70 wt% solution in water), hydrogen peroxide (H_2O_2, 30 wt% solution in water), lithium chloride (LiCl, 99%), sodium phosphate dibasic (Na_2HPO_4, 99+%), sodium hypophosphite monohydrate ($NaH_2PO_2 \cdot H_2O$, for analysis), cobalt(II) chloride hexahydrate ($CoCl_2 \cdot 6H_2O$, 98%), cobalt(II) sulfate heptahydrate ($CoSO_4 \cdot 7H_2O$, 98%), 4-vinylbenzoic acid (96%) and 4-pentenal (96%) were purchased from Thermo Scientific Chemicals. Hexachloroplatinic(IV) acid hexahydrate ($H_2PtCl_6 \cdot 6H_2O$, 38.0% Pt content) was purchased from TCI. 1-Ethyl-3-nitrobenzene (3-ENB, 95%) and 3-bromostyrene (97%) were purchased from BLD Pharm. 3-Vinylaniline (3-VA, 95%) and deuterium oxide (D_2O > 99.9 atom %) were purchased from Apollo Scientific. Sulfuric acid (H_2SO_4, 95%), hydrochloric acid (HCl, 37%), acetonitrile (MeCN, HPLC grade), ethanol (EtOH, ACS reagent grade), and acetic acid glacial (CH_3COOH, ≥ 98%) were purchased from VWR Chemicals. 2-Allylbenzaldehyde (97%) was purchased from Combi-Blocks. 1-Allyl-4-methoxybenzene was purchased from Fluka Chemicals. Acetone (chemically pure grade) and diethyl ether (chemically pure grade) were purchased from Biosolve. Deuterated chloroform ($CDCl_3$, 99.80% D) was purchased from Eurisotop. Dry MeCN was obtained from Inert solvent purification system. All chemicals and solvents were used as received without further purification.

Hydrogen gas (H_2, ≥ 99.999%) and nitrogen gas (N_2, ≥ 99.999%) were purchased from Linde Gas.

Ultrapure water (18.2 mω cm) was obtained from a Milli-Q Reference system and is referred to as MQ water in this manuscript.

Nickel foam (Ni foam) (thickness: 1 mm, PPI: 110 ppi, pore size: 0.23 mm, porosity: 98%) and copper foam (Cu foam) (thickness: 1 mm, PPI: 110 ppi, pore size: 0.23 mm, porosity: 98%) were purchased from Xiamen Zopin New Material Limited.

Ag/AgCl electrode (saturated KCl in MQ water), Ag/Ag^+ electrode (10 mM $AgNO_3$ and 100 mM TBAP in MeCN), Pt coil, graphite rod electrode, and sample holder with a Tantalum contact were purchased from Redoxme AB. Single compartment electrochemical cell, H-type electrochemical cell, and Nafion-117 proton exchange membrane were purchased from DekResearch. Reversible hydrogen electrode (RHE) was purchased from Gaskatel.

2. General methods

All electrochemical experiments were driven by or recorded with a BioLogic SP-50e potentiostat. Hydrogen gas flow was controlled by a high-precision thermal mass flow controller, red-y smart controller GSC, from Vögtlin Instruments GmbH. ^{1}H nuclear magnetic resonance (NMR) spectra were collected by a Bruker Avance Neo 500 MHz. Chemical shifts of ^{1}H-NMR are given in ppm. $CDCl_3$ (7.26 ppm) was used as deuterated solvent. Gas chromatography-mass spectrometry (GC-MS) was recorded on a Shimadzu GCMS-QP2020 NX, and liquid chromatography-mass spectrometry (LC-MS) was recorded on a Shimadzu LCMS-2020. Water content in reaction mixtures was measured by a Mettler Toledo C10SD Karl Fischer titrator. Surface-enhanced infrared absorption spectro-electrochemistry (EC-SEIRAS) was measured on a Bruker VERTEX 80 fourier transform infrared spectrometer equipped with a photovoltaic liquid N_2-cooled MCT (HgCdTe) detector under continuous purified air purge supplied by a purge gas generator (Peak Scientific Instruments PG14L) and using a BioLogic SP-50e potentiostat. Further details on EC-SEIRAS measurements can be found in the corresponding SI section. Scanning electron microscopy (SEM) and energy dispersive X-ray spectrometer (EDX) were performed by a Zeiss GeminiSEM 450 equipped with an Ametek EDAX Octane Elite EDS/EDX system at the Nano Imaging Lab, Swiss Nanoscience Institute, University of Basel. Powder X-ray diffraction (pXRD) was measured on a STOE StadiP powder diffractometer with a Dectris Mythen 1K-detector and a micro-focused Cu-Kα-source (λ = 1.542 Å). X-ray photoelectron spectroscopy (XPS) was measured in an ultrahigh vacuum (UHV) chamber equipped with a monochromatic Al-Kα X-ray source and a photoelectron spectroscopy analyzer (VG ESCALAB 210). XPS data was fitted using the Unifit software, and calibrated by shifting the spectra such that the carbon signal appeared at the reported value at 284.8 eV.[1] The geometry optimizations and frontier molecular orbital calculations were performed using density functional theory (DFT) with the Gaussian 09 program under Becke's 3-parameters employing the Lee-Yang-Perdew function (B3LYP) with the 6-31G(d) basis set.[2-7]

pH values of different reaction solutions in MeCN (^{MeCN}pH) were measured by a Mettler Toledo FiveEasy pH meter F20 with InLab® Science sensor. The outer reference electrolyte was changed to a 1 M LiCl solution in EtOH.[8] The electrode was then calibrated with aqueous pH standards.[9] We note that the ^{MeCN}pH values measured by this method should be interpreted with care and in the context of a MeCN- or MeCN/H_2O-mixture-specific pH scale that is distinct from the familiar pH scale applicable to aqueous solutions.[10,11] The HPLC-grade MeCN used herein contained ~0.01 v/v-% water and different reaction mixtures contained ~0.04–0.14 v/v-% water based on our water content measurements using a Karl Fischer titrator (Table S5). Furthermore, based on our replicate experiments the herein measured ^{MeCN}pH values can have a large uncertainty of ca. 1 pH value, which is a known issue with MeCN/H_2O-mixtures with low water content.[12] However, general trends observed with measured ^{MeCN}pH values should be more accurate and were generally consistent and clear within a given series of experiments (see e.g. Table S4). We therefore propose that the trends rather than absolute values of the measured ^{MeCN}pH values should be interpreted. For instance, a lower ^{MeCN}pH value indicates a more acidic bulk solution compared to a bulk solution having a higher ^{MeCN}pH value.

3. Experimental methods

3.1 Preparation of the Pt electrode

Pt electrode was prepared by electrodeposition with a three-electrodes system in a one-compartment electrochemical cell. Pt was deposited on Ni foam (2 × 1 cm^2) from a solution of 20 mM $H_2PtCl_6 \cdot 6H_2O$ and 20 mM HCl in MQ water by applying −0.6 V *vs*. Ag/AgCl for 1 h at 100 rpm stirring. The Ni foam, an Ag/AgCl electrode and a Pt coil were used as working, reference and counter electrode, respectively. Prior to the Pt deposition, the Ni foam was cleaned by an ultrasonic bath using 1 M H_2SO_4, acetone and MQ water for five minutes each. The Ag/AgCl reference electrode was calibrated regularly using an RHE electrode in a 50 mM H_2SO_4 solution.

3.2 General reaction setup and reaction conditions

Thermal hydrogenation catalysis was tested in an H-type electrochemical cell separated by a Nafion-117 membrane (perfluorosulfonic acid-PTFE copolymer) with a three-electrodes system. Figure S1 shows the setup of the reaction cell. A Pt electrode, an Ag/Ag^+ electrode and a graphite rod were used as working, reference, and counter electrode, respectively. The Ag/Ag^+ electrode was calibrated regularly using an RHE electrode in a 50 mM H_2SO_4 solution. Hydrogen gas (1 bar) was continuously sparged into both compartments and controlled at a certain flow rate by a mass flow controller with a typical flow rate of 5 standard cubic centimeters per minute (sccm). Standard experiments used 20 mL solution of 100 mM 3-NS and 100 mM $TBAPF_6$ in MeCN in each compartment. Reactions were typically run at room temperature (25 ± 1 °C) for 2 h and at 600 rpm stirring in the working compartment.

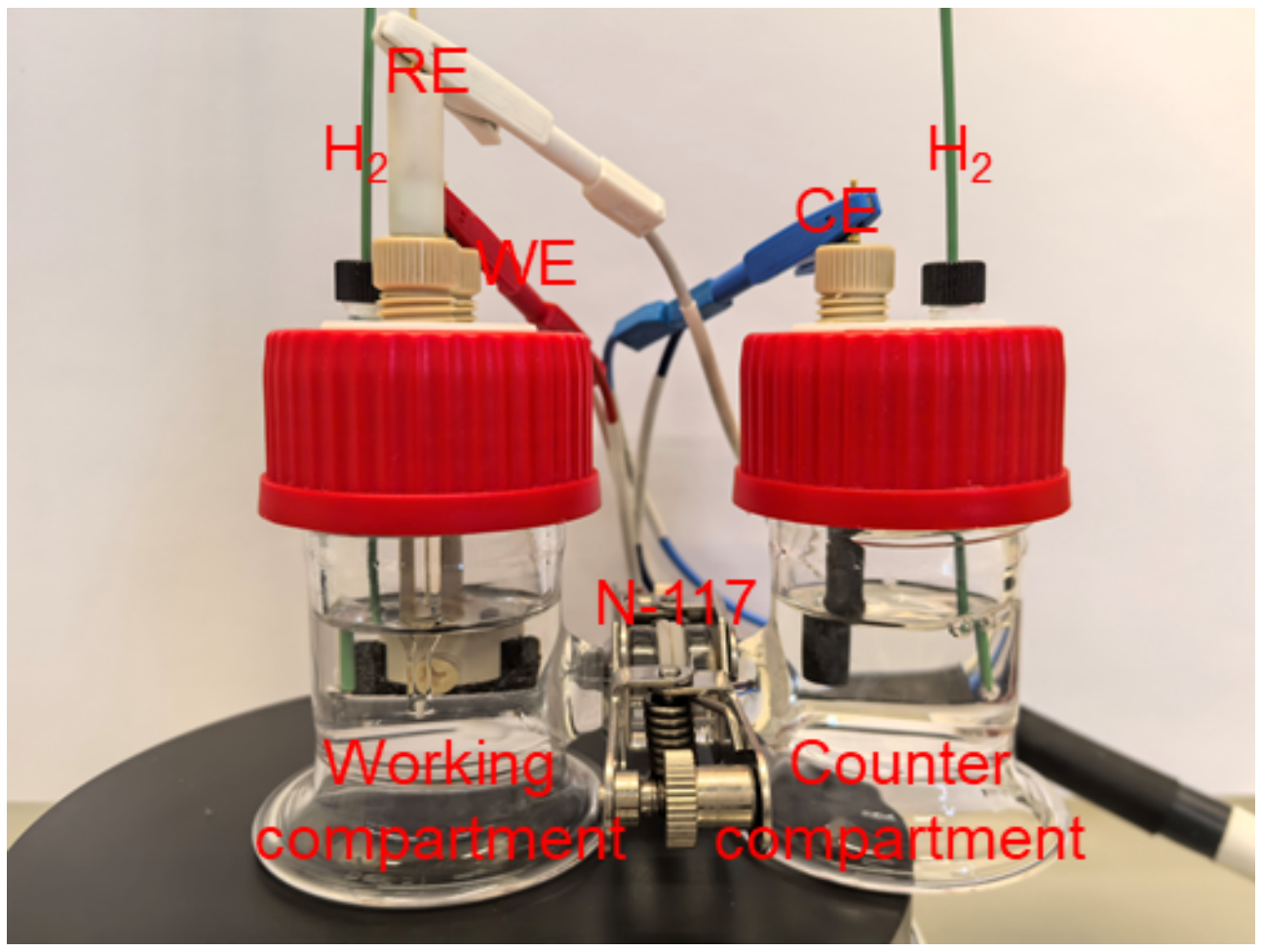

Figure S1. Photograph of the H-type electrochemical cell used to test thermal hydrogenation catalysis as a function of applied potential.

3.3 Thermal 3-NS hydrogenation at open circuit voltage

Thermal 3-NS hydrogenation reactions at OCV were run using the setup described above. E_{OCV} was recorded over time by a potentiostat after introducing 19.72 mL solution of a 101.4 mM $TBAPF_6$ in MeCN

into the setup (Figure S2). After the E_{OCV} had stabilized after 1 min, sparging of hydrogen gas into both cell compartments was started. After 20 min of hydrogen sparging, the E_{OCV} typically equilibrated to ca. −1.1 V *vs*. Ag/Ag^+. Then, upon addition of 0.28 mL of 3-NS to both compartments (which in total gives a solution that is 100 mM $TBAPF_6$ and 100 mM 3-NS in MeCN) shifted the E_{OCV} to ca. −1.0 V *vs*. Ag/Ag^+ immediately. E_{OCV} was then continuously monitored during the catalytic hydrogenation at OCV, typically run for 2 h. The catalytic performance was evaluated by taking 100 µL aliquots of the reaction mixture at the start of the reaction once 3-NS was added and after 2 h reaction time and analysis of these aliquots by NMR using 5 mg of 1,3,5-trimethoxybenzene as internal standard in 500 µL $CDCl_3$. This procedure was replicated three times to evaluate experimental reproducibility. An additional experiment was performed to evaluate the whole reaction time course and, for this, aliquots were taken every hour until full conversion of the reaction was reached (Figure S17).

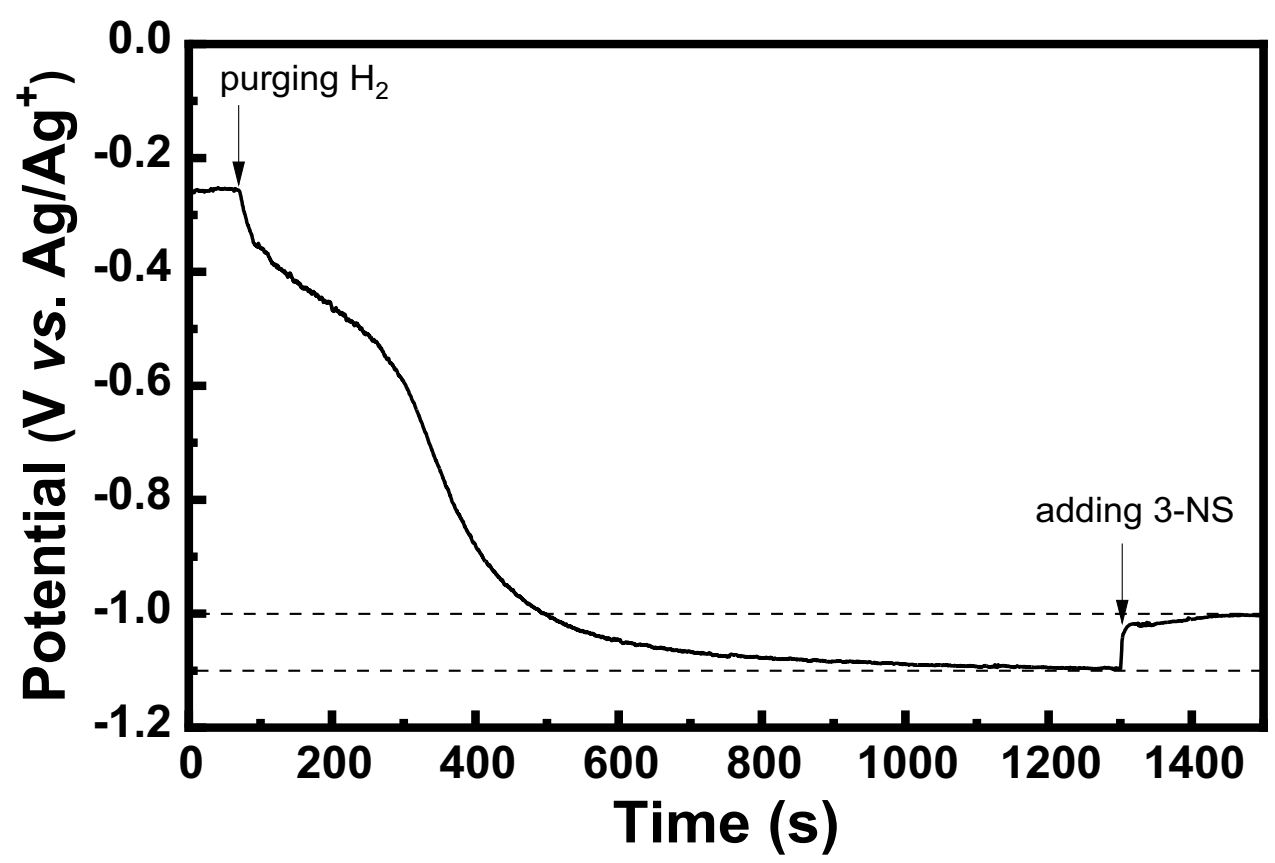

Figure S2. Representative example of the measured E_{OCV} in the experimental setup for 3-NS hydrogenation catalysis before, during and after addition of H_2 and 3-NS.

3.4 Thermal 3-NS hydrogenation at different applied potentials

Thermal 3-NS hydrogenation reactions at different applied potentials were done similarly to the procedure described above for OCV conditions. After introduction of 3-NS and an observed shift of E_{OCV} to ca. −1.0 V *vs*. Ag/Ag^+, the different constant potentials were applied to the Pt working electrode for 2 h. Evaluation of the catalytic performance was done as described for OCV conditions. No "on-the-fly" iR correction was applied in this work. This procedure was replicated three times to evaluate experimental reproducibility. An additional experiment was performed to evaluate the whole reaction time course and, for this, aliquots were taken every hour until full conversion of the reaction was reached (Figure S17).

3.5 EC-SEIRAS measurements

Detailed experimental procedures for EC-SEIRAS measurements can be found in SI section S9.

4. Product identification and reaction pathway analysis

4.1 Analysis of products observed during 3-NS hydrogenation catalysis: 3-ENB, 3-VA, and 3-EA

Products from catalytic reactions studied herein were identified using ^{1}H-NMR.

At the start of each 3-NS hydrogenation catalysis experiment, immediately after 3-NS was added, an aliquot of the reaction mixture (100 µL) was mixed with a solution of 5 mg TMB as internal standard in 500 µL $CDCl_3$ and analyzed by ^{1}H-NMR spectroscopy (Figure S3). The obtained NMR spectra showed signals associated with 3-NS (starting material), $TBAPF_6$ (supporting electrolyte), TMB (internal standard), MeCN (reaction solvent), deuterated chloroform (NMR solvent) (Table S1).

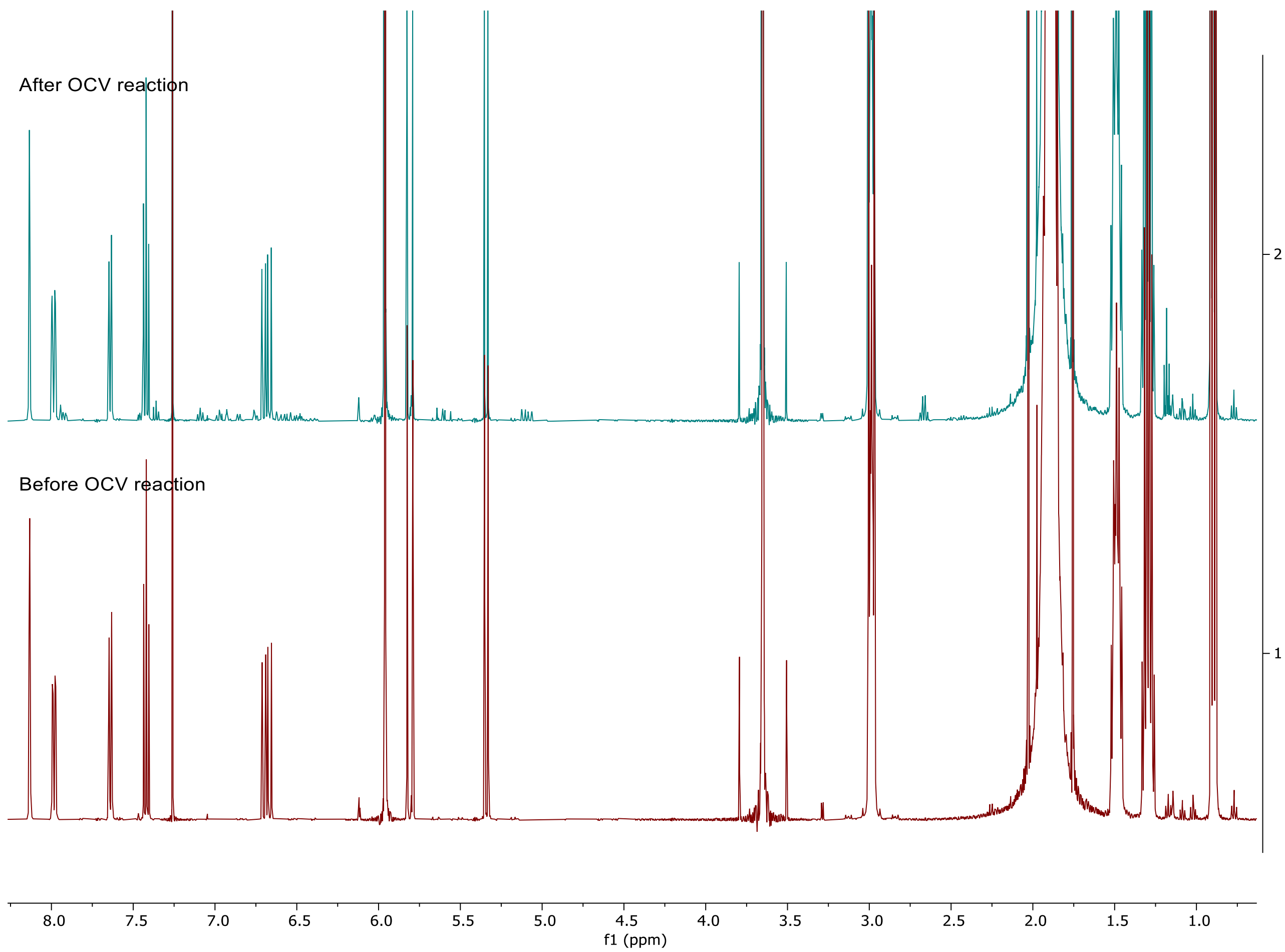

Figure S3. 500 MHz ^{1}H-NMR spectra of an aliquot of the reaction mixture at the start of 3-NS hydrogenation catalysis (bottom) and after 2 h reaction time at OCV (top) in $CDCl_3$.

Table S1. ^{1}H NMR signals of 3-NS, $TBAPF_6$, TMB, and MeCN.

Chemicals	^{1}H NMR (500 MHz, $CDCl_3$)
3-NS	8.17 (t, *J* = 2.0 Hz, 1H), 8.04 (ddd, *J* = 8.2, 2.3, 1.0 Hz, 1H), 7.66 (dt, *J* = 7.7, 1.2 Hz, 1H), 7.45 (t, *J* = 8.0 Hz, 1H), 6.72 (dd, *J* = 17.6, 10.9 Hz, 1H), 5.85 (d, *J* = 17.6 Hz, 1H), 5.40 (d, *J* = 10.9 Hz, 1H)
$TBAPF_6$	3.22–3.08 (m, 8H), 1.65–1.56 (m, 8H), 1.42 (m, 8H), 0.99 (t, *J* = 7.3 Hz, 12H)
TMB	6.09 (s, 3H), 3.77 (s, 9H)
MeCN	1.90 (s, 3H)

After 2 h reaction at OCV, another aliquot of the reaction mixture (100 µL) was mixed with a solution of 5 mg TMB as internal standard in 500 µL $CDCl_3$ and analyzed by ^{1}H-NMR spectroscopy (Figure S3). Signals associated with products were identified by comparison with the ^{1}H-NMR spectrum obtained at the start of hydrogenation catalysis.

After 2 h reaction at OCV, the intensities of NMR signals associated with 3-NS were decreased, as expected, and several groups of new signals appeared. We compared the new signals to ^{1}H-NMR spectra that we measured of possible hydrogenation products: 3-ENB, 3-VA, and 3-EA (Figure S4 and Table S2). This comparison suggested that 3-ENB, 3-VA, and 3-EA had been formed. These assignments were further verified by spiking the reaction mixture after 2 h reaction time at OCV separately with 3-ENB, 3-VA, and 3-EA (Figure S5).

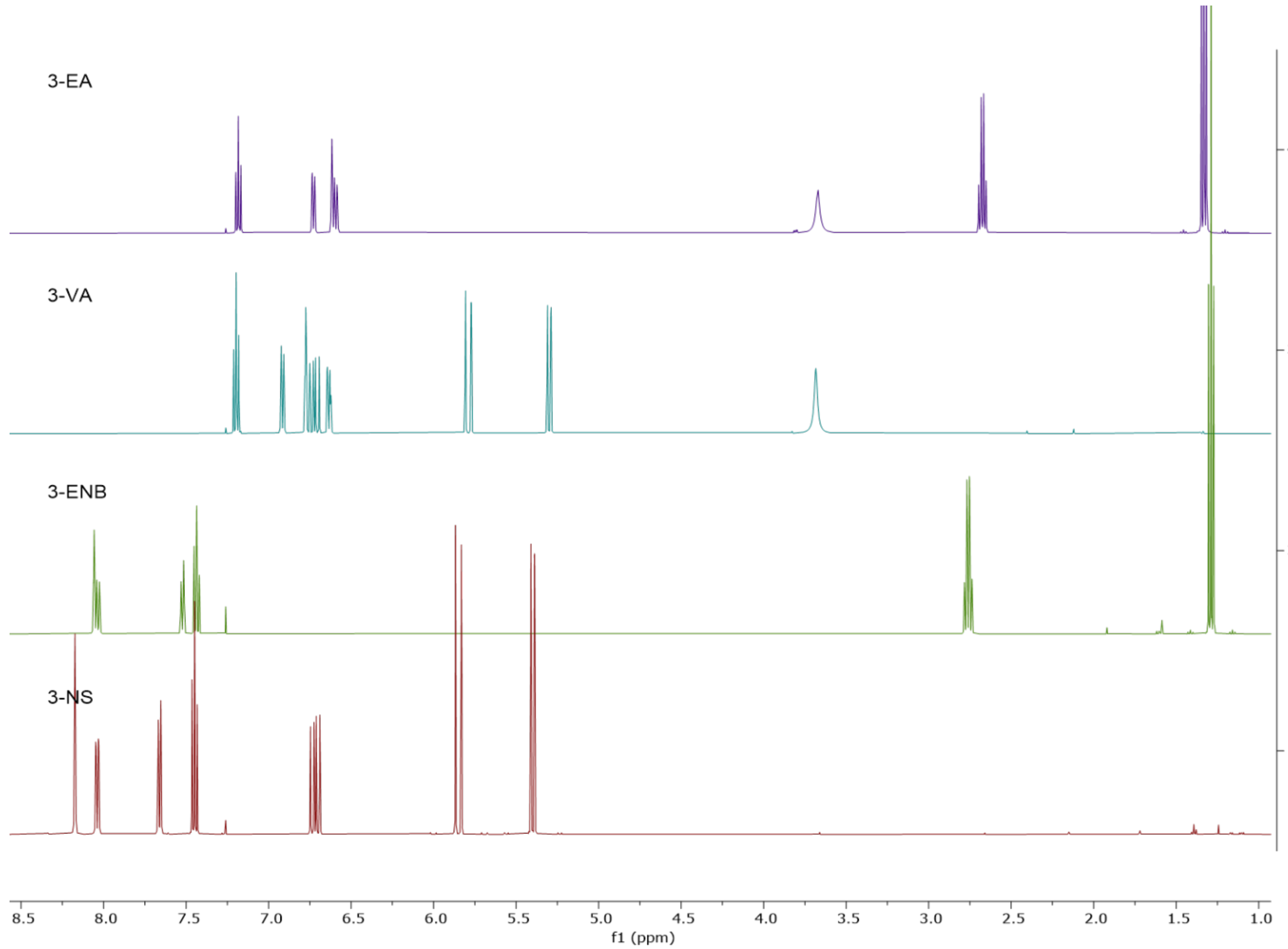

Figure S4. 500 MHz ^{1}H-NMR spectra of 3-NS, 3-ENB, 3-VA, and 3-EA in $CDCl_3$.

Table S2. ^{1}H NMR signals of commercial chemicals of 3-ENB, 3-VA, and 3-EA.

Chemicals	^{1}H NMR (500 MHz, $CDCl_3$)
3-ENB	8.06 (tt, *J* = 1.7, 0.5 Hz, 1H), 8.03 (ddd, *J* = 8.1, 2.3, 1.1 Hz, 1H), 7.52 (ddq, *J* = 7.5, 1.8, 0.6 Hz, 1H), 7.44 (t, *J* = 7.9 Hz, 1H), 2.76 (q, *J* = 7.6 Hz, 2H), 1.29 (t, *J* = 7.6 Hz, 3H)
3-VA	7.20 (t, *J* = 7.8 Hz, 1H), 6.92 (d, *J* = 7.6 Hz, 1H), 6.78 (t, *J* = 2.0 Hz, 1H), 6.72 (dd, *J* = 17.6, 10.8 Hz, 1H), 6.63 (ddd, *J* = 7.9, 2.4, 1.0 Hz, 1H), 5.79 (dd, *J* = 17.7, 1.0 Hz, 1H), 5.30 (dd, *J* = 10.9, 1.0 Hz, 1H), 3.68 (s, 2H)
3-EA	7.18 (t, *J* = 7.7 Hz, 1H), 6.73 (ddd, *J* = 7.5, 1.7, 0.6 Hz, 1H), 6.62 (tt, *J* = 1.7, 0.6 Hz, 1H), 6.59 (ddd, *J* = 7.8, 2.4, 0.9 Hz, 1H), 3.67 (s, 2H), 2.67 (q, *J* = 7.6 Hz, 2H), 1.33 (t, *J* = 7.6 Hz, 3H)

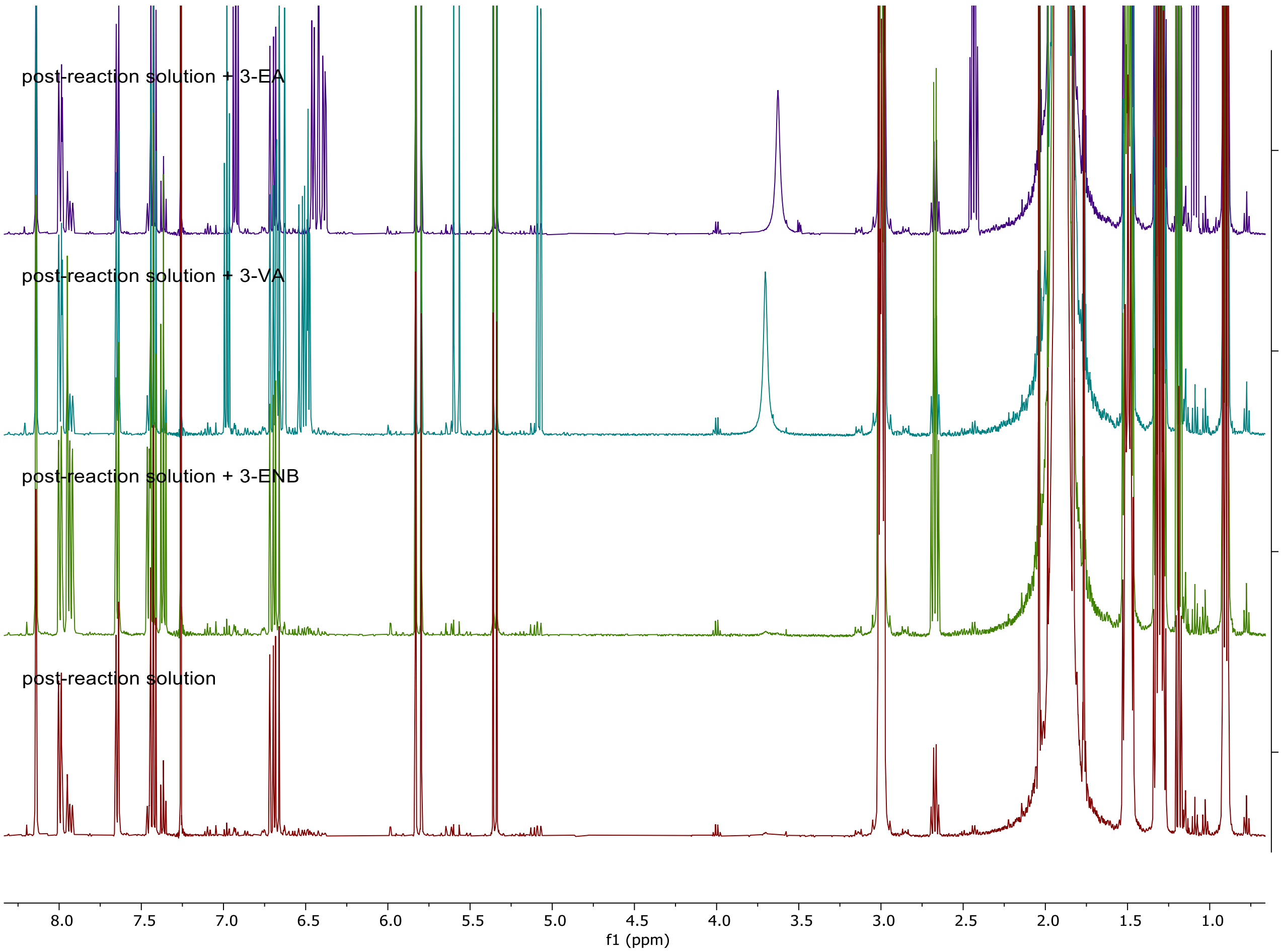

Figure S5. 500 MHz ^{1}H-NMR spectra in $CDCl_3$ of an aliquot of the reaction mixture after 2 h reaction at OCV (bottom, red), and this mixture spiked with either 3-ENB (green), 3-VA (blue), or 3-EA (purple).

4.2 Analysis of additional products observed during 3-NS hydrogenation catalysis: 3-VAOH and 3-EAOH

Above we showed the evidence for the formation of 3-ENB, 3-VA, and 3-EA. However, the reaction mixture after 2 h reaction time at OCV still showed several other unassigned signals (Figure S5). Below we show how we identified these products by analyzing the ^{1}H-NMR spectra in more detail. We also attempted to identify these unidentified products by separation of the products from the mixture and analysis by NMR, GC-MS, and LC-MS as well as by GC-MS and LC-MS of the crude reaction mixture. However, the products associated with the unassigned NMR signals could not be isolated or characterized by these methods.

^{1}H-NMR analysis of these unidentified products was made difficult by the presence of overlapping signals from 3-NS. To increase the signal intensities of unidentified products we ran the catalysis for longer reaction times twice, once 10 h and once 30 h. We focused our analysis on the signals that were already visible after 2 h reaction time. Figure S6 (region of 8.2–5.0 ppm) and S7 (region of 3.9–0.8 ppm) show the details to thirteen unassigned signals.

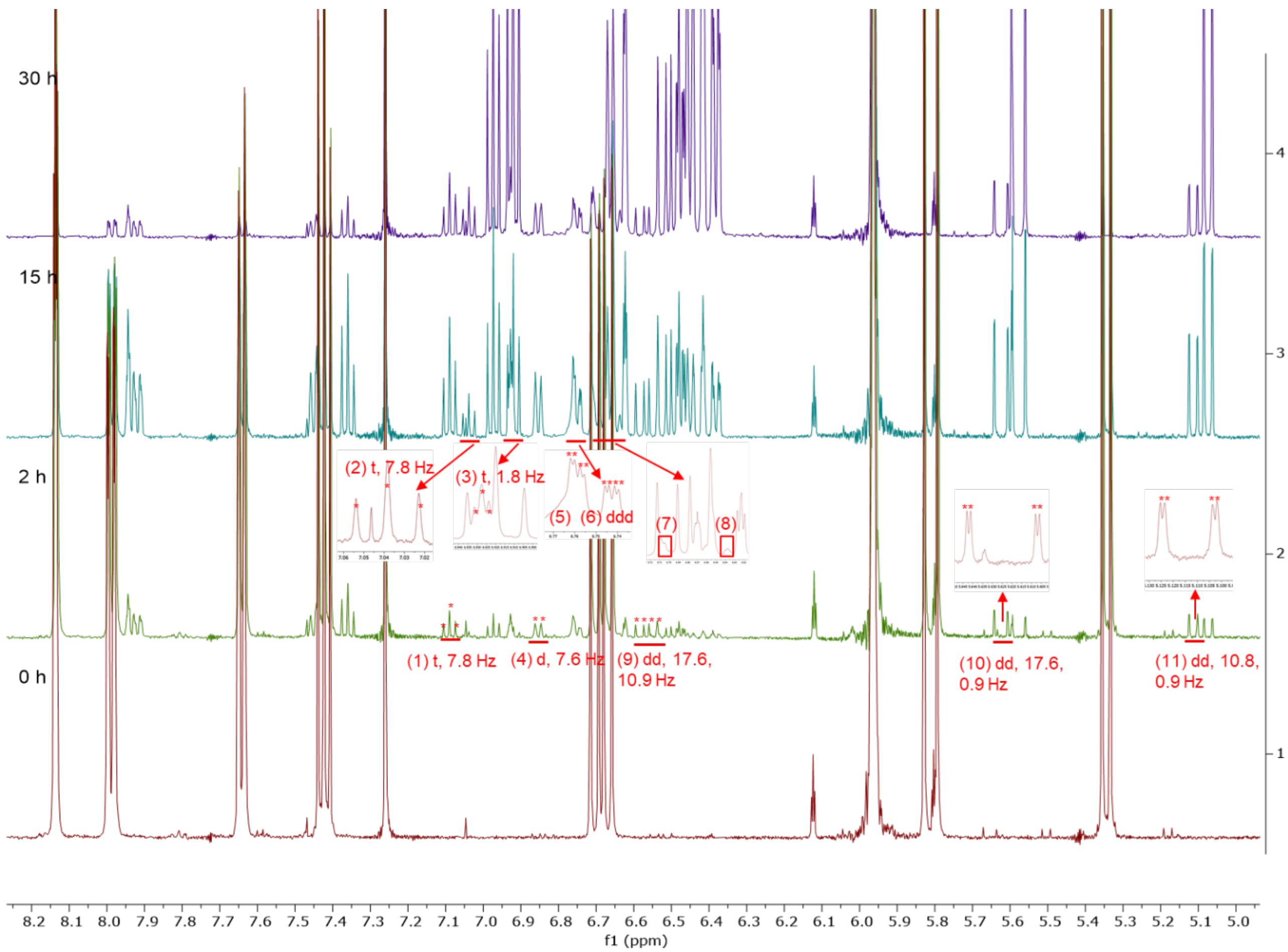

Figure S6. 500 MHz ^{1}H-NMR spectra in $CDCl_3$ of an aliquot of the reaction mixture at the start of 3-NS hydrogenation catalysis at OCV (0 h, bottom, red), the mixture after 2 h (green), 15 h (blue), and 30 h (purple) reaction time. The spectra are shown in the region 8.2–5.0 ppm to highlight unassigned signals.

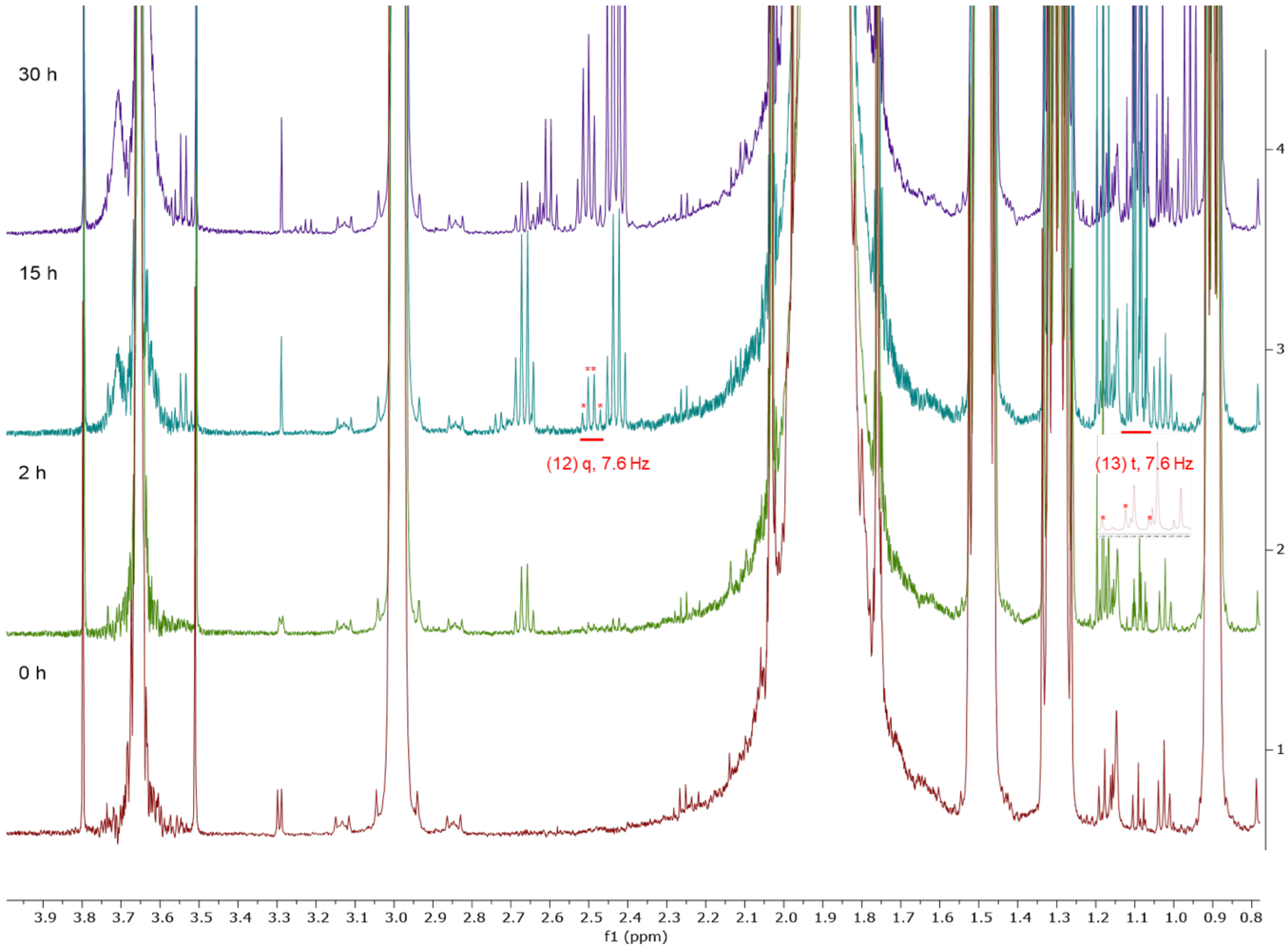

Figure S7. 500 MHz ^{1}H-NMR spectra in $CDCl_3$ of an aliquot of the reaction mixture at the start of 3-NS hydrogenation catalysis at OCV (0 h, bottom, red), the mixture after 2 h (green), 15 h (blue), and 30 h (purple) reaction time. The spectra are shown in the region 3.9–0.8 ppm to highlight unassigned signals.

To identify possible unidentified products, we considered possible reaction mechanisms of 3-NS hydrogenation. The hydrogenation of functionalized nitroarenes can occur by a direct route or a condensation route.[13,14] These possible hydrogenation reaction pathways are shown for 3-NS in Figure S8.[15] In both pathways, the nitro-group is first reduced to a nitroso-group and then, quickly further hydrogenated to the corresponding hydroxylamine.[16,17] Hence, the nitroso-based intermediate is typically only obtained in very small amounts.[16,17] In the direct route, the formation of the amine then requires breaking the N–O bond of the corresponding hydroxylamine. This is usually a slow step, which can cause the accumulation of the hydroxylamine.[16,17] Another route involves the condensation of a nitroso-intermediate and a hydroxylamine to form an azoxy-dimer. This is followed by hydrogenation steps to the corresponding azo- and hydrazo-dimers, and then two of the corresponding amines. Unidentified products observed during our 3-NS hydrogenation catalysis could hence either correspond to hydroxylamines or any of the dimers. We attempted to identify whether the unidentified products are hydroxylamines or dimeric species based on a mass balance with the consumed amount of 3-NS and the 3-ENB, 3-VA, and 3-EA products formed. However, the uncertainty of this analysis associated with the very small amount of

unidentified products formed and the standard uncertainty of NMR quantification was too large to obtain clear conclusions. However, the condensation route seems unlikely to occur under the conditions used herein for 3-NS hydrogenation catalysis as it typically occurs under strongly alkaline conditions.[16,17] Hence, the observed unidentified products are likely to be associated with hydroxylamines, with 3-vinyl-*N*-hydroxyaniline (3-VAOH) and 3-ethyl-*N*-hydroxyaniline (3-EAOH). Below, we analyze the obtained NMR spectra in more detail to test if the observed unassigned signals match what would be expected for 3-VAOH and 3-EAOH.

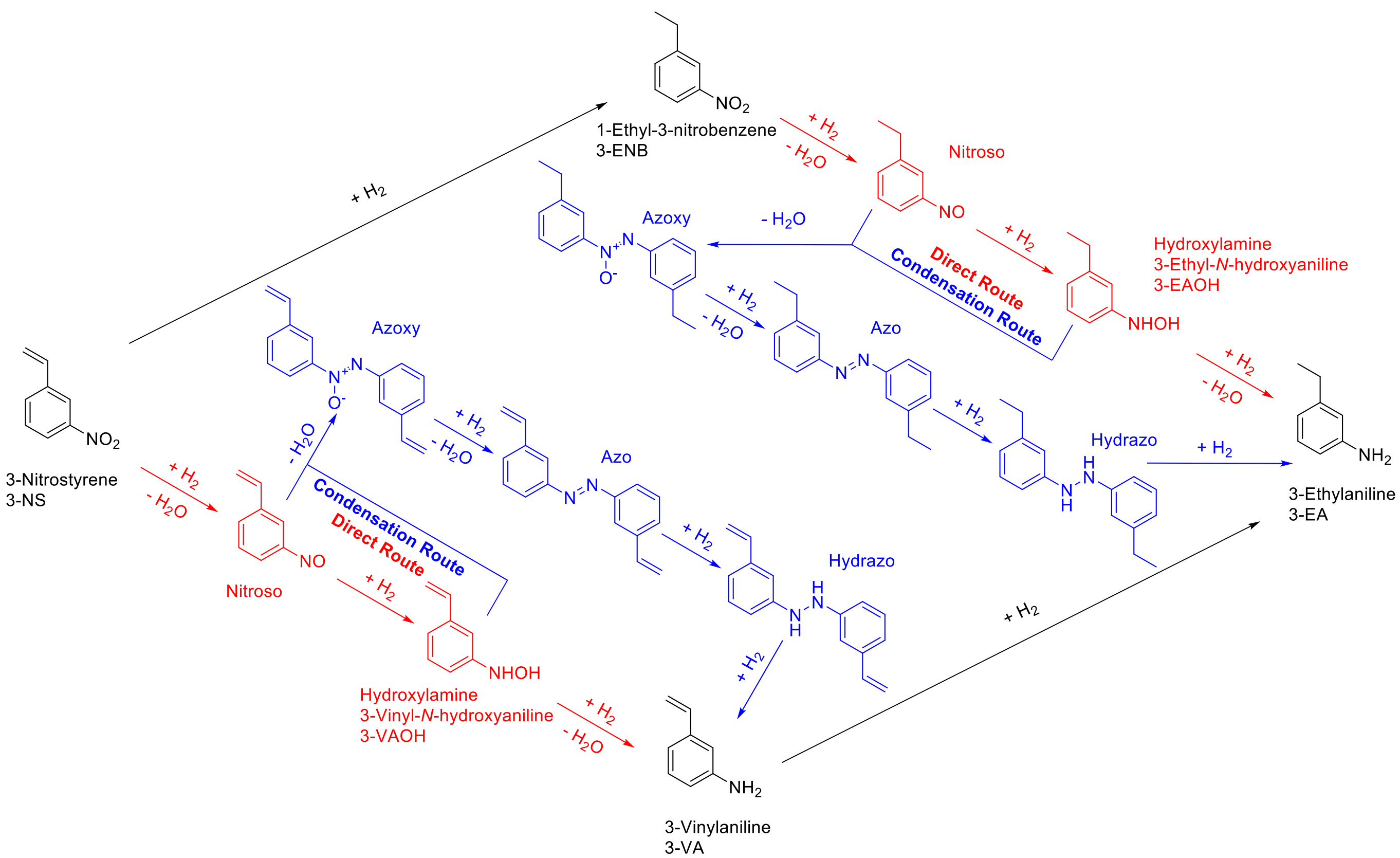

Figure S8. General possible reaction pathways of the 3-NS hydrogenation based on prior literatures.[16,17]

To analyze which of the thirteen small NMR signals observed belonged to the same compound we analyzed the integrals of these signals over time during 3-NS hydrogenation catalysis (Figure S9). For this, the integrals were referenced to the integral value of the signal at 3.7 ppm associated with the internal standard TMB, which was set to 1000. Figure S9 shows that the thirteen signals associated with unidentified products are divided into two groups: first group (1, 3, 4, 6, 9, 10, and 11) and second group (2, 5, 7, 8, 12, and 13).

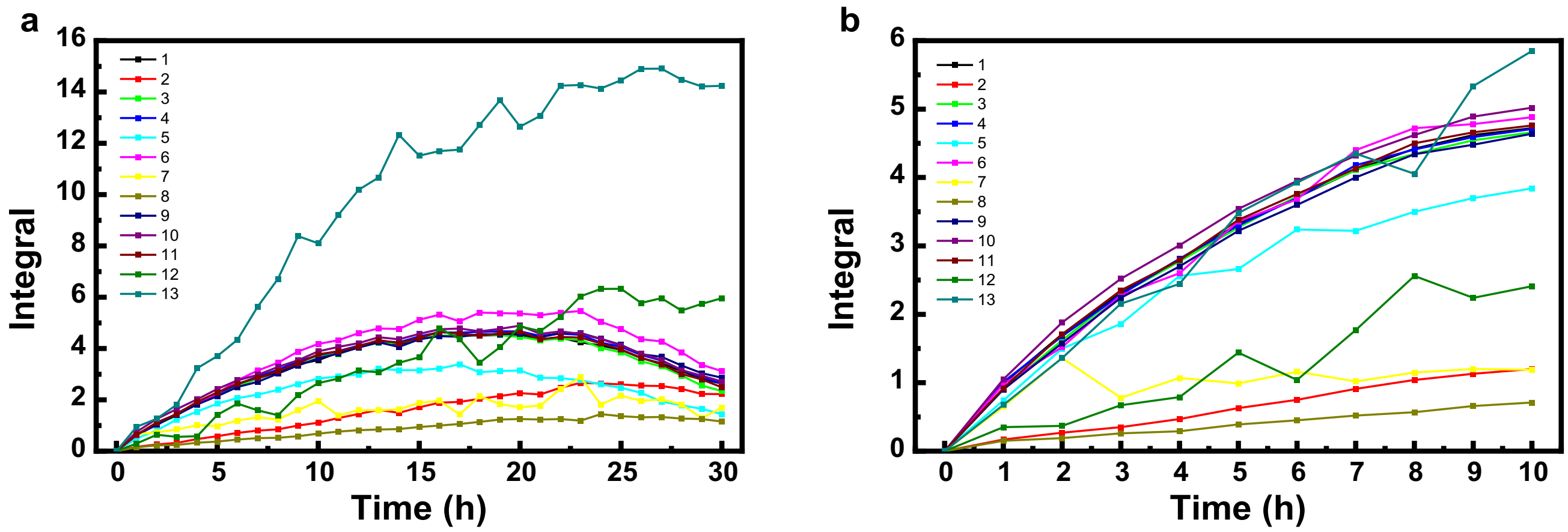

Figure S9. Integral values of thirteen ^{1}H-NMR signals associated with unidentified products obtained during the 3-NS hydrogenation after (a) 30 h and (b) 10 h reaction time.

Figure S10 shows the integral values of the first group. The evolution of these seven signals showed very similar trends. This was reproduced in two independent replicate experiments. We hence attributed these seven signals to one product. Peak 9 (dd, J = 17.6, 10.9 Hz), 10 (dd, J = 17.6, 0.9 Hz), and 11 (dd, J = 10.8, 0.9 Hz) show correlative J-coupling value consistent with those of a $–CH=CH_2$ group and similarly to the signals associated with the vinyl-group in 3-NS and 3-VA. The chemical shifts, patterns, and J-coupling values of the other four peaks (1, 3, 4, and 6) in the region of 7.1–6.7 ppm are similar to the signals associated with the protons of the benzene group in 3-VA. Based on these data associated with the $–CH=CH_2$ group and a benzene-group we hence attribute the seven peaks of the first group to 3-VAOH. This assignment is consistent with literature: ^{1}H NMR (500 MHz, $CDCl_3$): δ 7.24–7.21 (t, J = 6.5 Hz, 1H), 7.04–7.03 (m, 1H) 6.88–6.87 (d, J = 7.5 Hz), 6.70–6.64 (dd, $J1$ = 17.5; $J2$ =11 Hz, 1H), 5.74–5.71 (d, J = 17.5 Hz, 1H), 5.25–5.23 (d, J = 11 Hz, 1H),[18] and ^{1}H NMR (400 MHz, $CDCl_3$): δ 7.26 (m, 1H), 7.08 (m, 2H), 6.92–6.91 (m, 1H), 6.73–6.66 (m, 1H), 5.75 (d, J = 20 Hz, 1H), 5.27 (d, J = 8 Hz, 2H).[19]

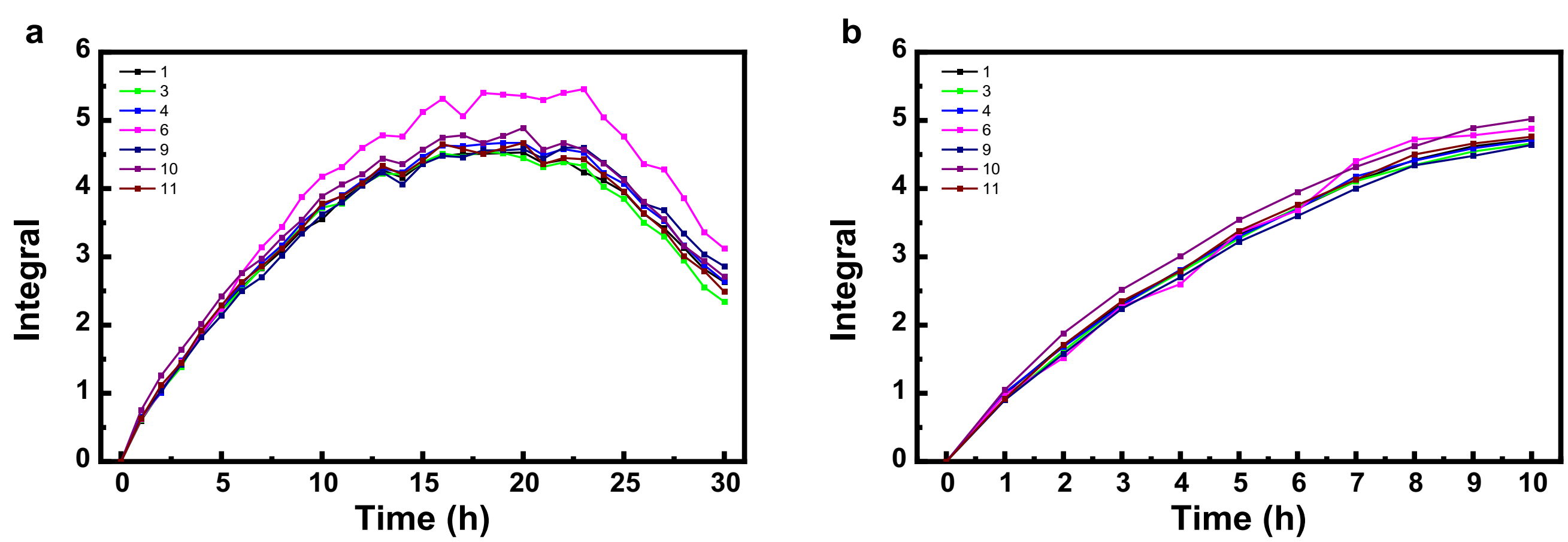

Figure S10. Integrated signal intensities of the first group of ^{1}H NMR signals associated with unidentified product (attributed to 3-VAOH) observed during the 3-NS hydrogenation after (a) 30 h and (b) 10 h reaction time.

Figure S11 shows the integral value of the second group. Peak 12 (q, J = 7.6 Hz), and 13 (t, J = 7.6 Hz) show coupling patterns and matching J-coupling constants consistent with a $–CH_2–CH_3$ group, also resembling the pattern obtained for 3-ENB and 3-EA. In Figure S11 the integrals of the peaks 12 and 13 are scaled by a factor of 1/2 and 1/3 according to the number of protons each signal corresponds to (2 and 3 protons, respectively). We note that the integrals of peak 13, 7, and 5 were difficult to determine accurately as these signals overlapped substantially with other signals. Similarly, peak 8 was close to other NMR signals and integrated peak area can likely not be accurately determined. The observation of four signals in the region 7.1–6.6 ppm seems consistent with a benzene-group. Based on the signals attributed to the $–CH_2–CH_3$ and benzene-groups, our considerations above, and the observation of 3-VAOH, we attribute the six NMR signals of the second group to 3-EAOH.

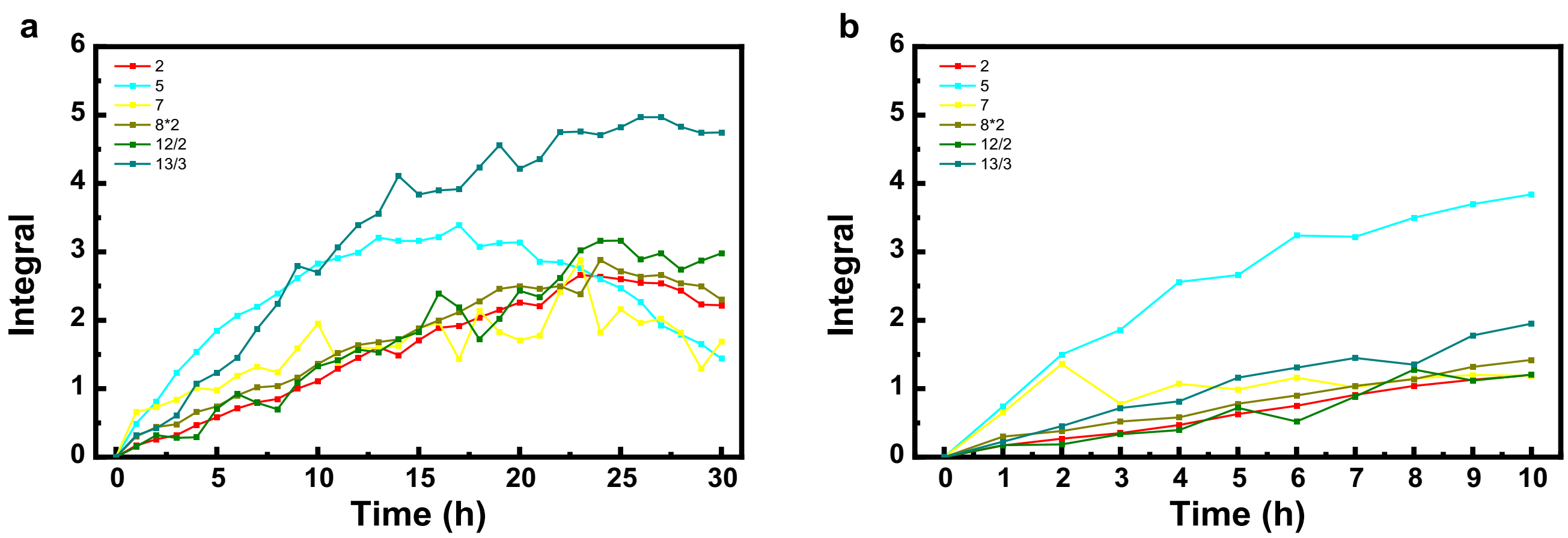

Figure S11. Integrated signal intensities of the second group of ^{1}H-NMR signals associated with unidentified product (attributed to 3-EAOH) observed during the 3-NS hydrogenation after (a) 30 h and (b) 10 h reaction time.

4.3 Reaction products and reaction pathway for 3-NS hydrogenation catalysis

Overall, based on our analysis above and prior literature, catalytic 3-NS hydrogenation at OCV leads to the products 3-ENB, 3-VA, and 3-EA, and likely 3-VAOH and 3-EAOH. It hence seems more likely that catalysis goes via the direct route (Figure S12).[16,17]

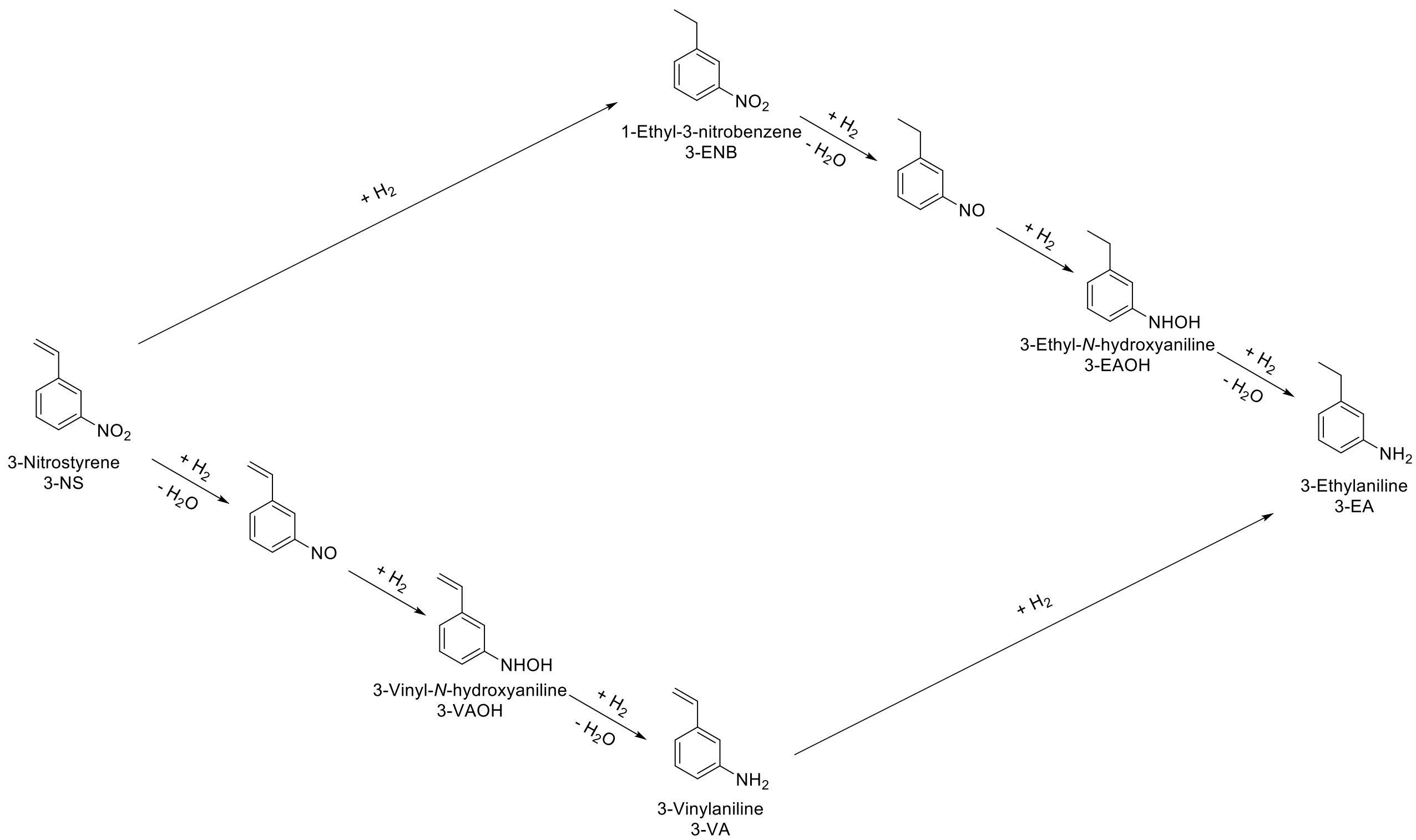

Figure S12. Proposed reaction pathways of the 3-NS hydrogenation at OCV.

4.4 Analysis of products or reactions at the counter electrode

Table S7 shows the measured ^{MeCN}pH values of the reaction mixture before and after 3-NS hydrogenation at OCV or −0.2 V in working and counter chambers. The relative basicity of the counter chamber with respect to the working chamber as well as with respect to the solution in the counter chamber before catalytic hydrogenation suggests that H^+ are being reduced at the counter electrode via the hydrogen evolution reaction (HER). The H^+ may originate from the Nafion N-117 membrane or perhaps from the hydrogen oxidation reaction (HOR) in the working chamber, though, transport through the separating Nafion-117 membrane is inefficient under our conditions (see Table S7). The observed much less acidic solution obtained in a single compartment cell experiment is in line with this separating effect of the N-117 membrane (Table S14). That the dominating reductive process at the counter electrode is associated with HER is consistent with the observation of small bubbles at the counter electrode during the hydrogenation catalysis at −0.2 V applied to the working electrode.

^{1}H-NMR spectra were measured of the solution in the counter chamber before and after hydrogenation catalysis at OCV or −0.2 V. For this, an aliquot of the reaction mixture in the counter chamber (100 µL) was taken at the start of each 3-NS hydrogenation catalysis experiment immediately after 3-NS addition. This aliquot was then mixed with a solution of 5 mg TMB as internal standard in 500 µL $CDCl_3$ and analyzed by ^{1}H-NMR spectroscopy. After 2 h reaction time at OCV or −0.2 V, another aliquot of the reaction mixture from the counter chamber (100 µL) was mixed with a solution of 5 mg TMB as internal standard in 500 µL $CDCl_3$ and analyzed by ^{1}H-NMR spectroscopy. Figure S13 shows the ^{1}H-NMR spectra obtained for the reaction done at −0.2 V as an example.

The ^{1}H-NMR spectra obtained from the aliquots of the counter chamber before and after hydrogenation catalysis done in the counter chamber mainly showed signals associated with 3-NS (starting material), $TBAPF_6$ (supporting electrolyte), TMB (internal standard), MeCN (reaction solvent), deuterated chloroform (NMR solvent) (Table S1 and Figure S13). The concentration of 3-NS in the counter chamber before and after catalysis remained the same within uncertainty. However, the reaction mixture of the counter chamber after 2 h reaction time at −0.2 V reaction showed additional signals of very low intensity (Figures S14). These small signals may be associated with organic products obtained from a minor reductive process occurring at the counter electrode, possibly from 3-NS. The signals do not match the NMR signals expected for 3-ENB, 3-VAOH, 3-VA, 3-EAOH, or 3-EA, and could not be more clearly assigned.

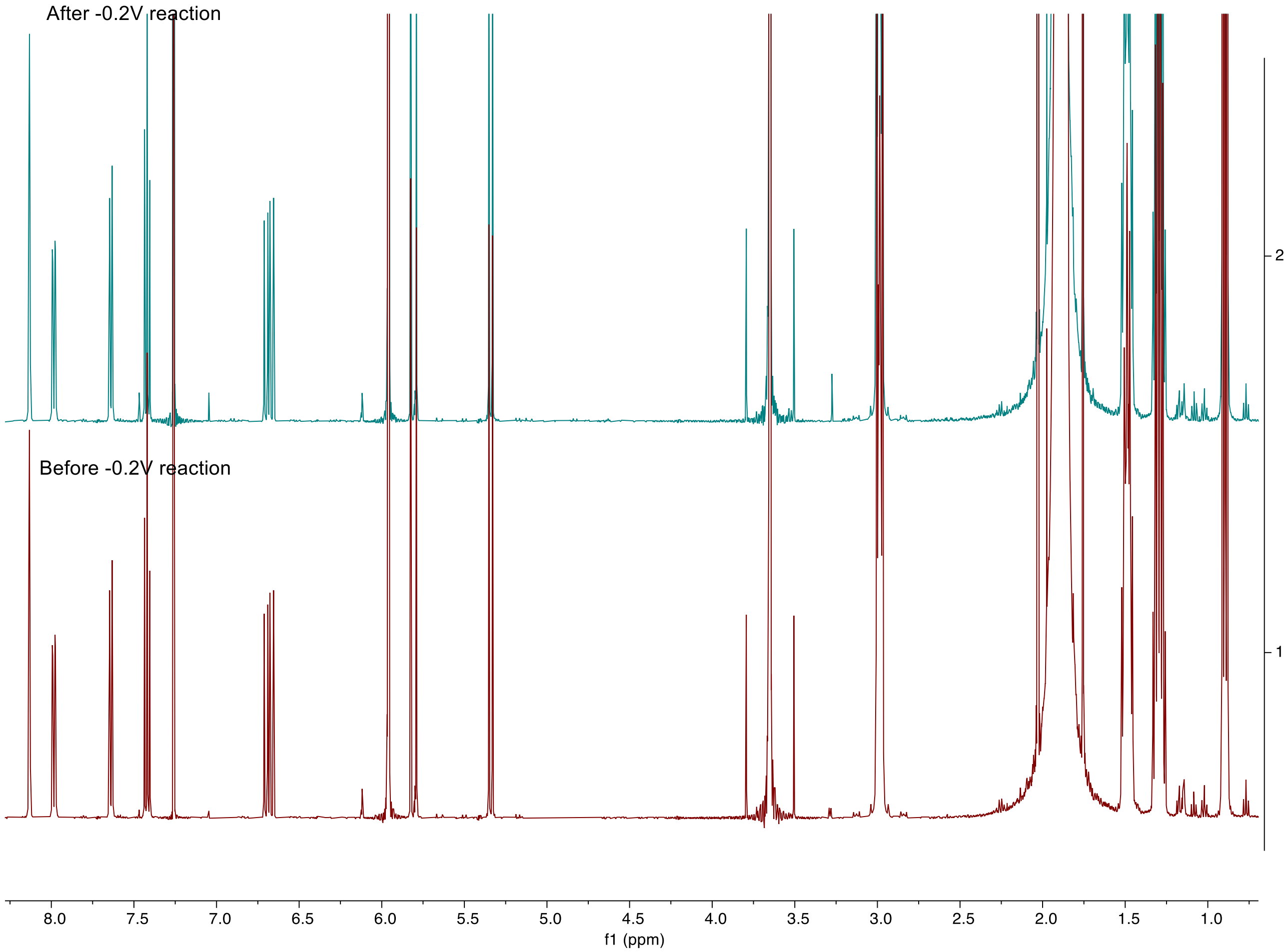

Figure S13. 500 MHz ^{1}H-NMR spectra in $CDCl_3$ of an aliquot of the reaction mixture in the counter chamber at the start of 3-NS hydrogenation catalysis (bottom) and after 2 h reaction time at −0.2 V (top).

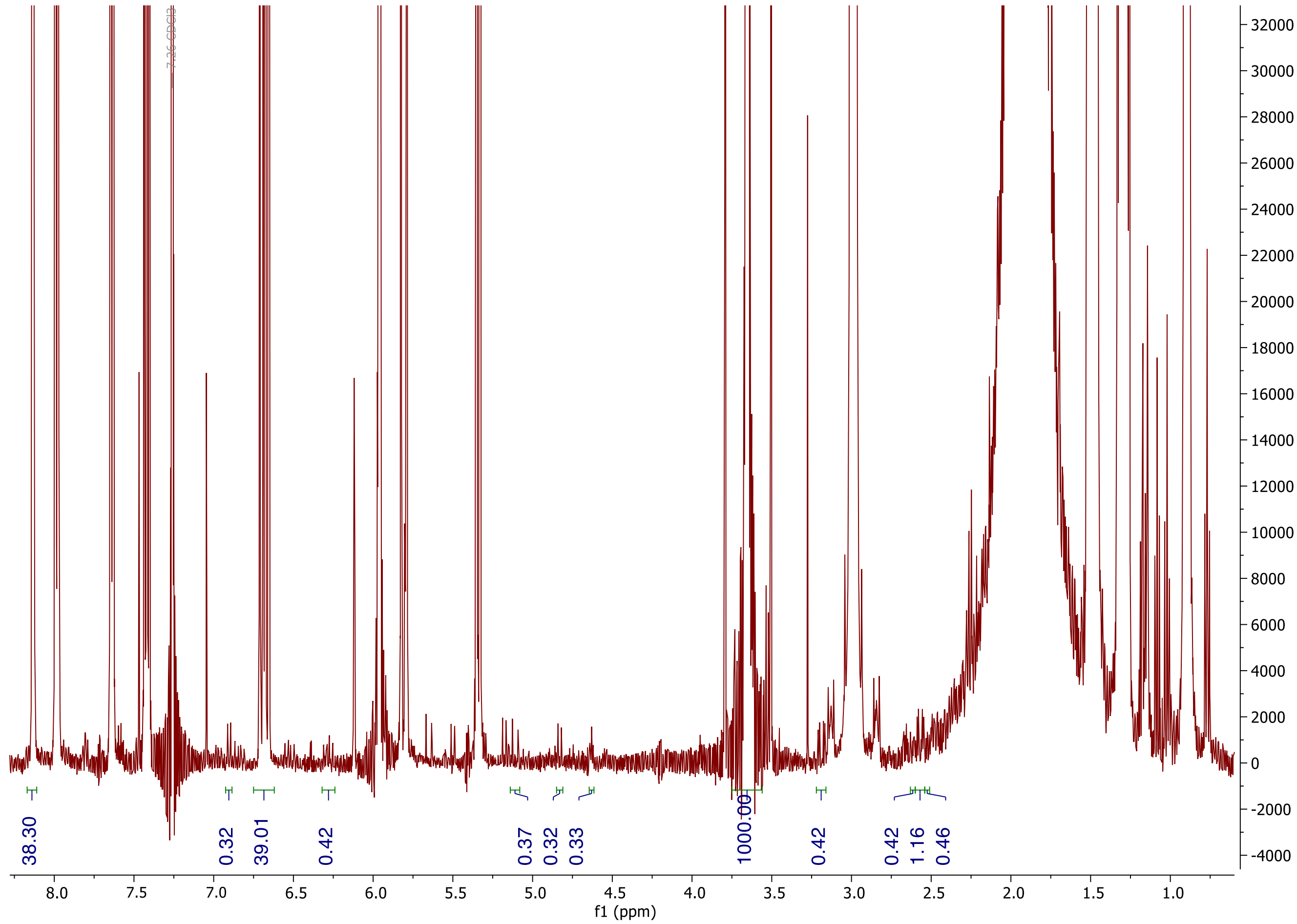

Figure S14. Zoom of the 500 MHz ^{1}H-NMR spectrum in $CDCl_3$ shown in Figure S13 on top of an aliquot taken from the solution in the counter chamber after 2 h reaction time at −0.2 V. The integrals shown were referenced to the integral value of the signal at 3.7 ppm associated with the internal standard TMB, which was set to 1000. Two signals from 3-NS at 8.17 ppm (t, J = 2.0 Hz, 1H) and 6.72 ppm (dd, J = 17.6, 10.9 Hz, 1H) had the integral values of 38.30 and 39.01. The integral values of the unassigned product(s) formed in the counter chamber were around 0.3–0.4 (the dd peak at 2.60–2.54 ppm overlaps with the strong signal of MeCN. A background correction from integrating the half of the peak region on both sides of the dd peak suggests that the corrected integral value of the dd peak at 2.60–2.54 ppm is 0.28).

5. Thermal 3-NS hydrogenation as a function of applied potential

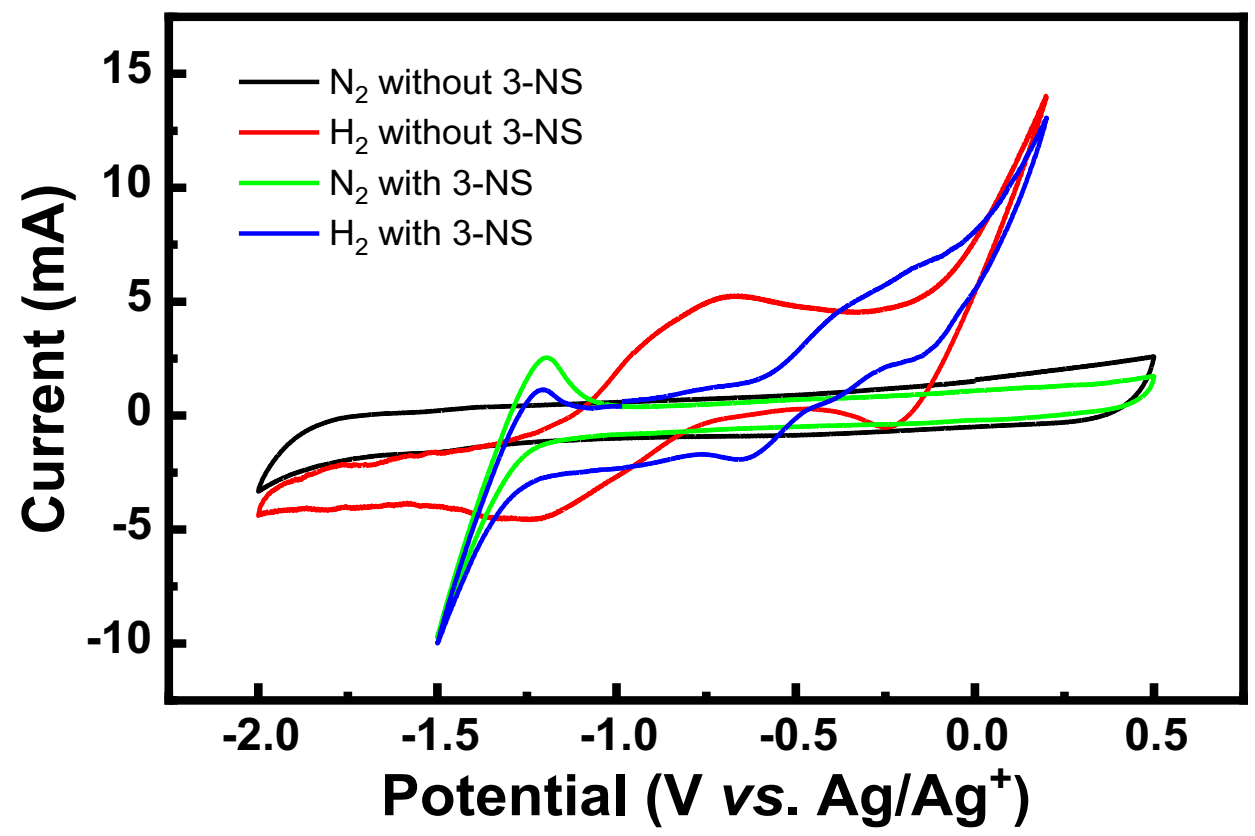

Figure S15. Cyclic voltammograms (CV) measured in an H-cell with a solution of 100 mM $TBAPF_6$ in MeCN and a Pt electrode, an Ag/Ag^+ electrode and a graphite rod as working, reference, and counter electrode, respectively. When the cell was sparged with $N_{2(g)}$ and in the absence of 3-NS (black CV), the CV showed no Faradaic currents between −2 and +0.5 V in line with the expected wide potential window of the Pt, MeCN and $TBAPF_6$. When sparging with $H_{2(g)}$ (red CV), oxidation and reduction currents were observed that are likely associated with hydrogen adsorption/desorption, the HOR, and the HER. In presence of 3-NS while sparging with $N_{2(g)}$ (green CV) a reduction current starting at −1.2 V was observed, which we attribute to the electrochemical reduction of 3-NS with residual water content in MeCN. Upon sparging a solution of 3-NS with $H_{2(g)}$ the CV (blue CV) shows new features compared to the green CV, which we hence attribute to hydrogen adsorption/desorption, HOR, and HER.

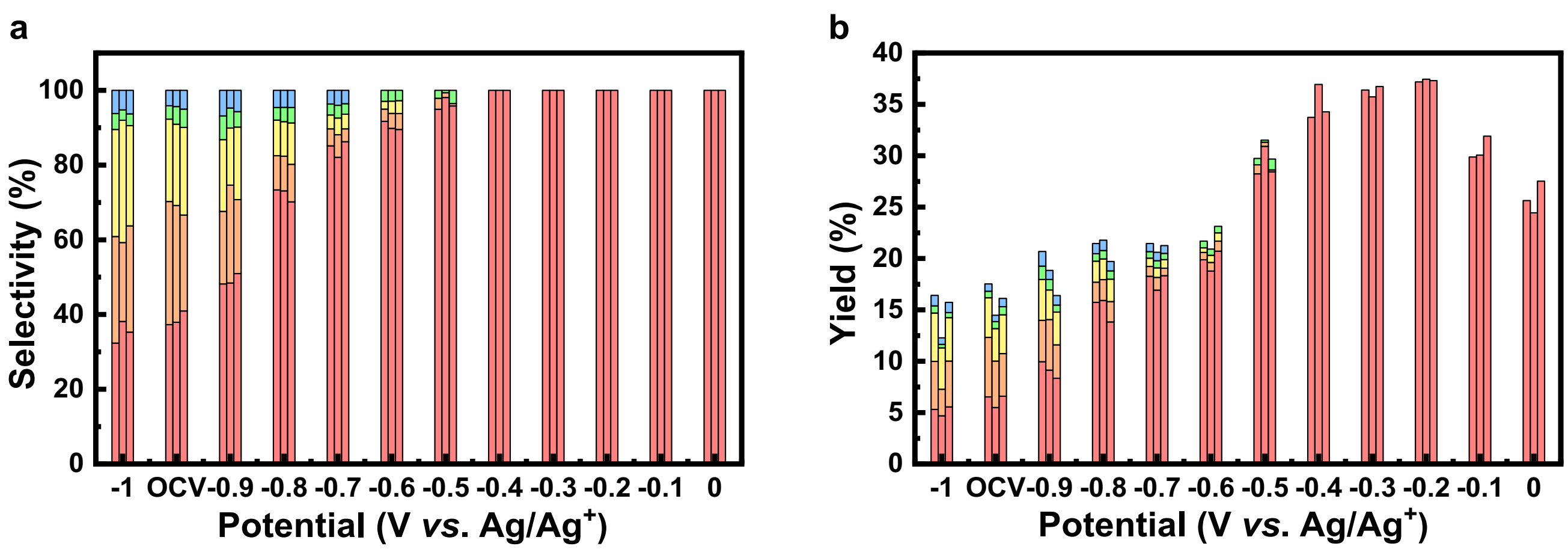

Figure S16. (a) Selectivity and (b) yield obtained in three independent replicate experiments of the thermal 3-NS hydrogenation and measured as a function of applied potential. Colored bars correspond to the observed products: 3-ENB (red), 3-VAOH (orange), 3-VA (yellow), 3-EAOH (green), and 3-EA (blue).

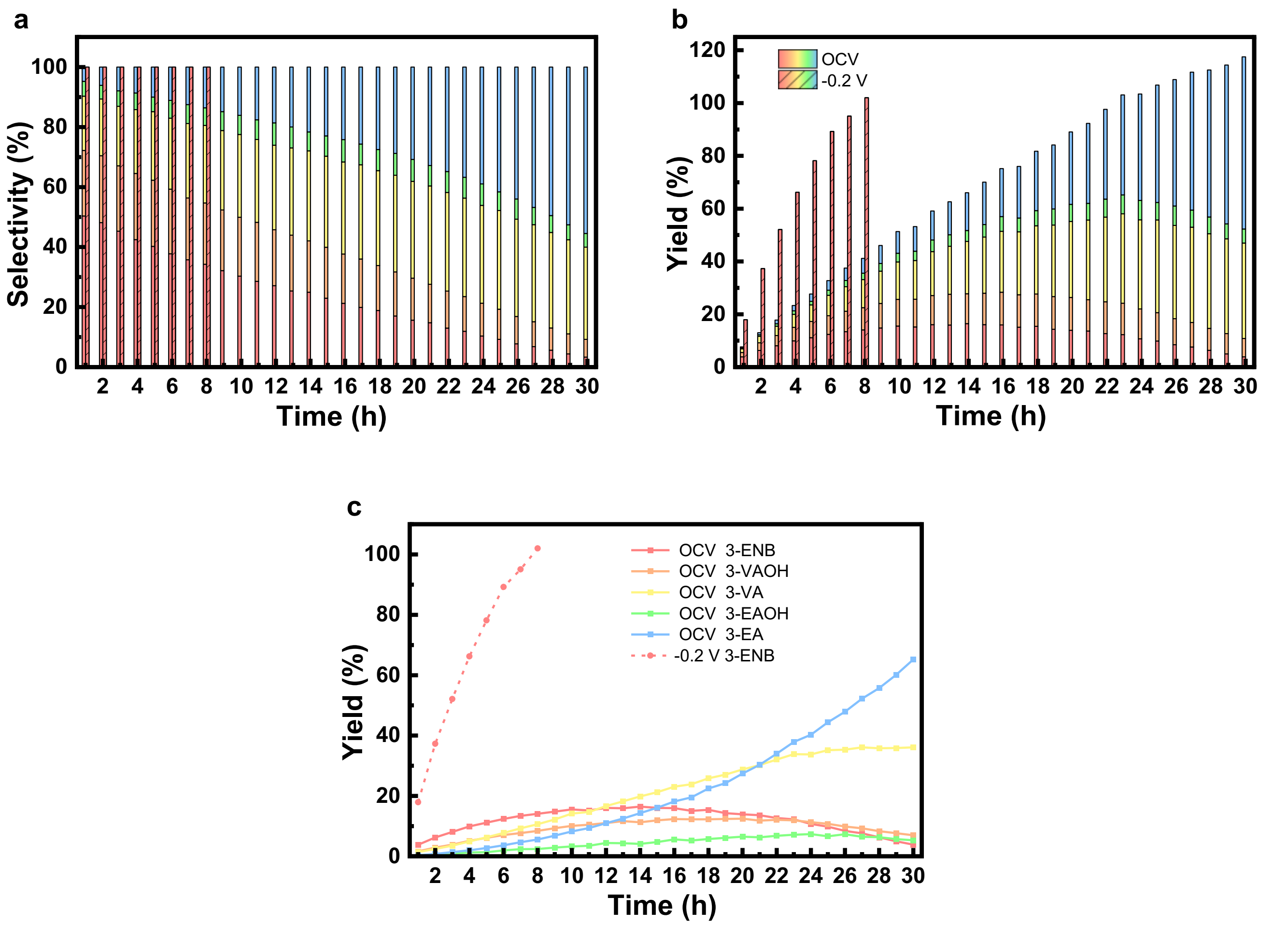

Figure S17. (a) Selectivity and (b) yield, (c) and yield shown for each product individually of the thermal 3-NS hydrogenation at OCV or at −0.2 V versus time, where the experiment was run until full conversion was reached. Aliquots for analysis were taken every hour. The formal yields of >100% obtained here are likely a result of the uncertainty with these measurements associated with effects such as possible small amounts of solvent and product evaporation, and slight reduction of the overall solution due to the aliquots taken, as well as substrates and products traversing through the Nafion-117 membrane, which are difficult to control in our setup at this low level. We note that these effects are negligible during the standard 2 h reaction time. At −0.2 V, only 3-ENB was observed as product even at full conversion. On the other hand, at OCV, five products were observed and the fully saturated hydrogenation product, 3-EA, continuously increased. Figure S17c shows product formation over time. At OCV, the amount of 3-ENB first increased and then decreased again (red solid lines). This suggests that 3-ENB is both a product and an intermediate, likely because 3-ENB can be further hydrogenated to 3-EAOH and 3-EA at OCV. The amount of 3-VA (yellow solid line) and 3-EA (blue, solid line) steadily increased over time until full conversion was reached. We note that this pattern does not necessarily imply that 3-VA is not further hydrogenated to 3-EA, as suggested in Figure S12. The steady increase in 3-VA may simply reflect differences in the rates of 3-VA formation and its subsequent hydrogenation to 3-EA.

Table S3. Representative strategies for selective hydrogenation of 3-NS using Pt-based catalysts. Literature reports for different strategies were chosen as example. More comprehensive overviews over prior work can be found elsewhere.[16,17,20,21]

Method	Catalyst	Main product	Over-hydrogenated product 3-EA observed	Solvent	T (°C)	P (bar)	Time (h)	Conversion (%)	Selectivity (%)	Reversible modification of the catalytic behavior	Ref.
EPOC	Pt	3-ENB	No	MeCN	RT	1	8	~100	100	Yes	This work
Metal oxide as the support	0.2%Pt/TiO_2	3-VA	Yes	Toluene	40	3	0.67	99	90	No	[22]
Carbon material as the support	Pt/ACH-150	3-ENB	Yes	Toluene	100	40	0.08	95	93	No	[23]
Nanosizing and TiO_2 support	0.2% Pt/TiO_2	3-VA	Yes	Toluene	40	3	6.5	95	93	No	[24]
Bimetallic catalyst and porous support	Pt-Zn/HPS	3-VA	Yes	Ethanol	75	10	N/A	~100	97	No	[25]
Bimetallic catalyst	Pt-Bi/TiO_2-1.5	3-VA	Yes	Water	85	20	4	100	96	No	[26]
Nano catalyst in ionic liquid and reaction medium	Pt-B@IL	3-ENB	Yes	Ethyl acetate	RT	1	N/A	100	95	No	[27]
Single-atom catalyst	SAC 0.08%Pt/FeOx	3-VA	Yes	Toluene	40	3	0.83	96.5	98.6	No	[28]

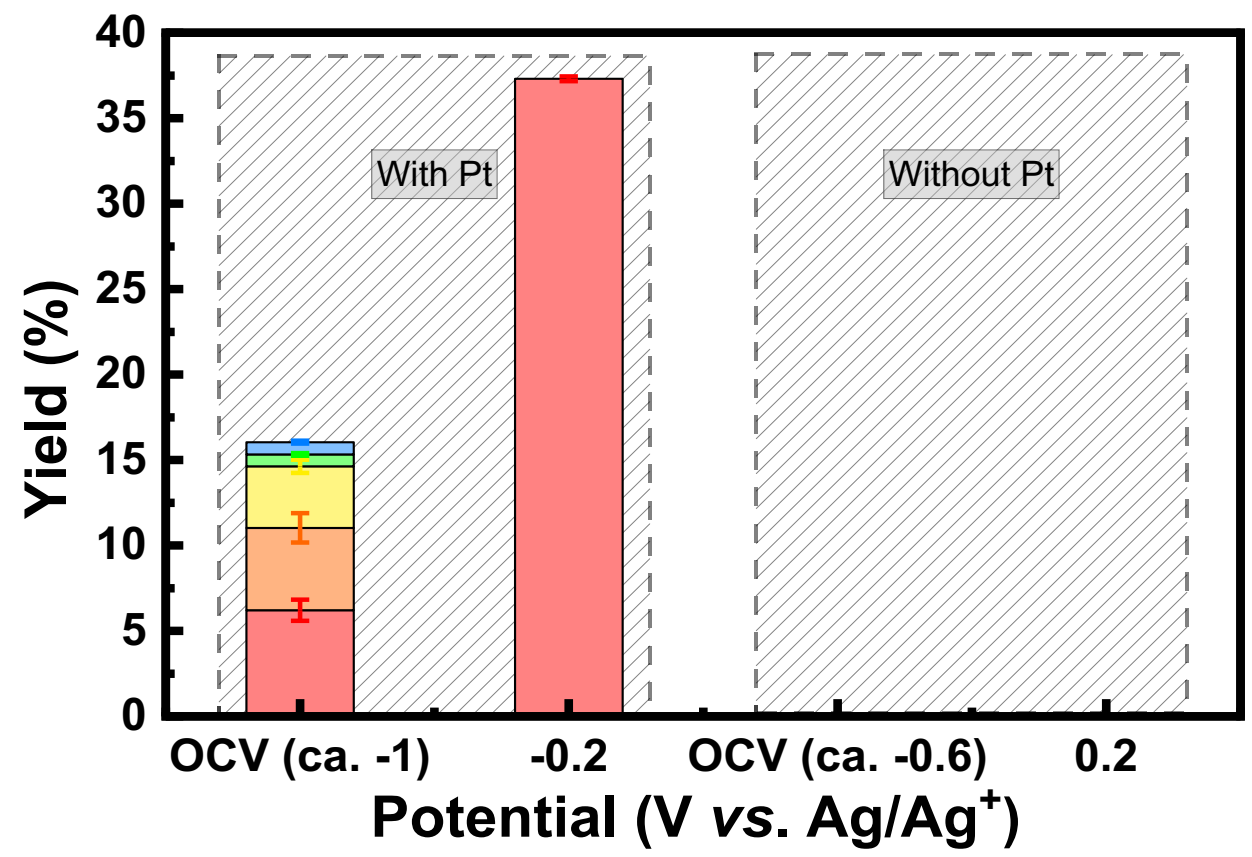

Figure S18. Control experiments showing that there is no background activity from the Ni foam used to support the Pt catalyst (right) and comparison to the high catalytic activity (product yield) obtained with Pt as a catalyst for the 3-NS hydrogenation (left, data is reproduced from Figure 1 in the main text). The bare Ni foam was cleaned by an ultrasonic bath using 1 M H_2SO_4, acetone and MQ water for five minutes each. Then, the catalytic activity of the bare Ni foam was tested for the hydrogenation of 3-NS at OCV as well as at positive applied potential. Upon adding the 3-NS into the H_2 saturated solution, an E_{OCV} of −0.6 V was obtained with the bare Ni foam, which is less negative than the −1.0 V typically obtained with Pt and in line with the lower ability of Ni to activate hydrogen. A positive applied potential of +0.2 V was used to test hydrogenation activity of the Ni foam, as this is more positive by +0.8 V compared to the E_{OCV} of the Ni foam, similarly to what we used for Pt. No hydrogenation products were observed at either OCV or applied potential using the bare Ni foam.

6. Testing the possibility of electrocatalytic 3-NS hydrogenation under the conditions studied herein

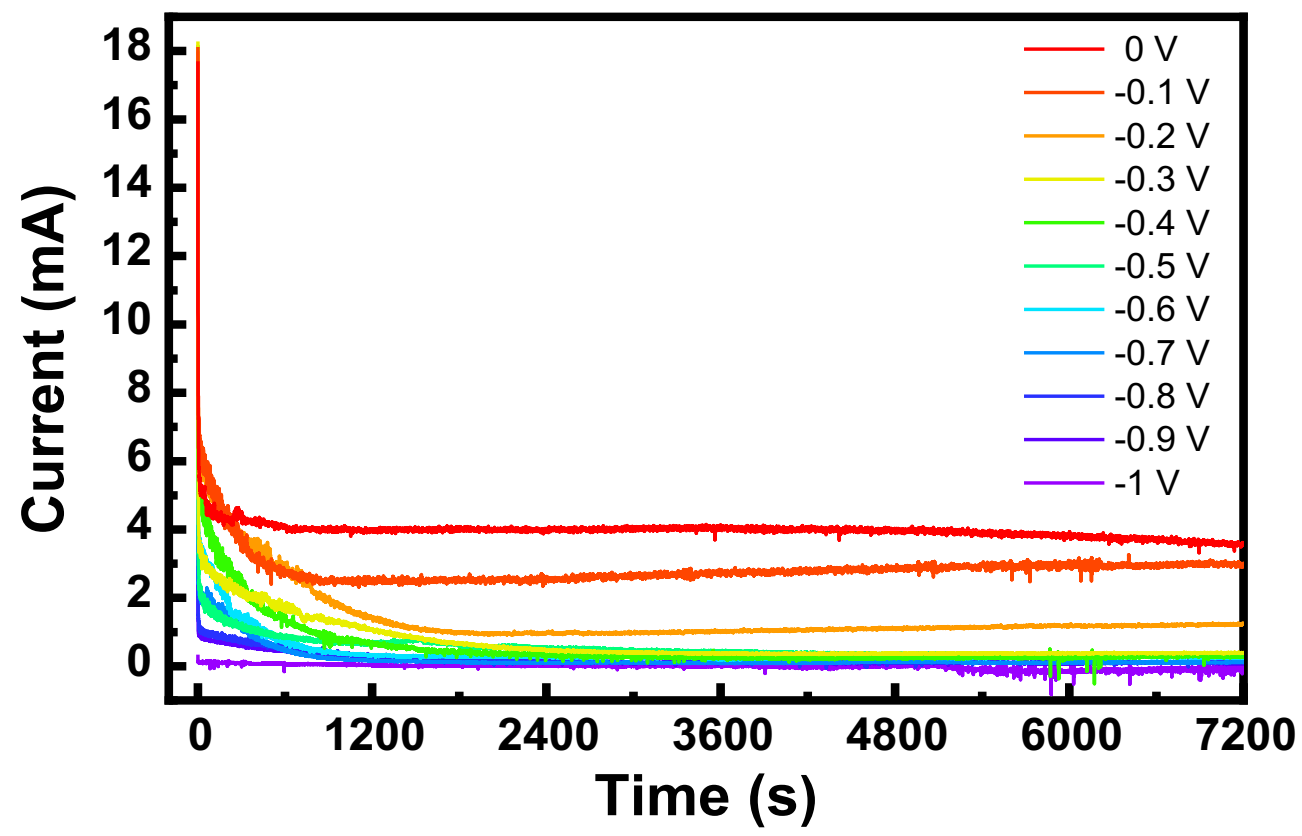

Figure S19. Current measured during the 3-NS hydrogenation at different applied potentials over 2 h reaction time. All currents measured at different applied potentials (except −1 V) are positive currents.

Table S4. Average electric charge, and average current measured at different applied potentials from three independent replicate experiments, and measured ^{MeCN}pH values in the working chamber solution after the hydrogenation of 3-NS. Absolute ^{MeCN}pH values should be cautiously interpreted due to the uncertainty associated with such measurements in organic solvents and with respect to a MeCN-based pH scale, but general trends observed should be more accurate. Detailed comments about pH measurement can be found in SI section S2.

	−1 V	−0.9 V	−0.8 V	−0.7 V	−0.6 V	−0.5 V	−0.4 V	−0.3 V	−0.2 V	−0.1 V	0 V
Average electric charge (C)	−0.39 ± 0.24	1.18 ± 0.24	1.33 ± 0.67	1.73 ± 0.59	2.43 ± 0.35	3.61 ± 0.91	4.97 ± 0.95	5.46 ± 0.93	9.04 ± 1.76	21.34 ± 1.77	28.86 ± 1.63
Average current[a] (mA)	−0.06 ± 0.03	0.16 ± 0.03	0.19 ± 0.09	0.24 ± 0.08	0.34 ± 0.05	0.50 ± 0.13	0.69 ± 0.13	0.76 ± 0.13	1.26 ± 0.24	2.96 ± 0.25	4.01 ± 0.23
^{MeCN}pH of working chamber solution[b]	8.53	−0.02	−0.05	−0.10	−0.25	−0.32	−0.58	−0.62	−0.88	−1.05	−1.15

[a] The average current values were determined with $i = \frac{Q}{t}$, where the Q is the average electric charge in this table and the t is the reaction time of 7200 s. [b] The ^{MeCN}pH value of reaction solution before reaction was 5.55.

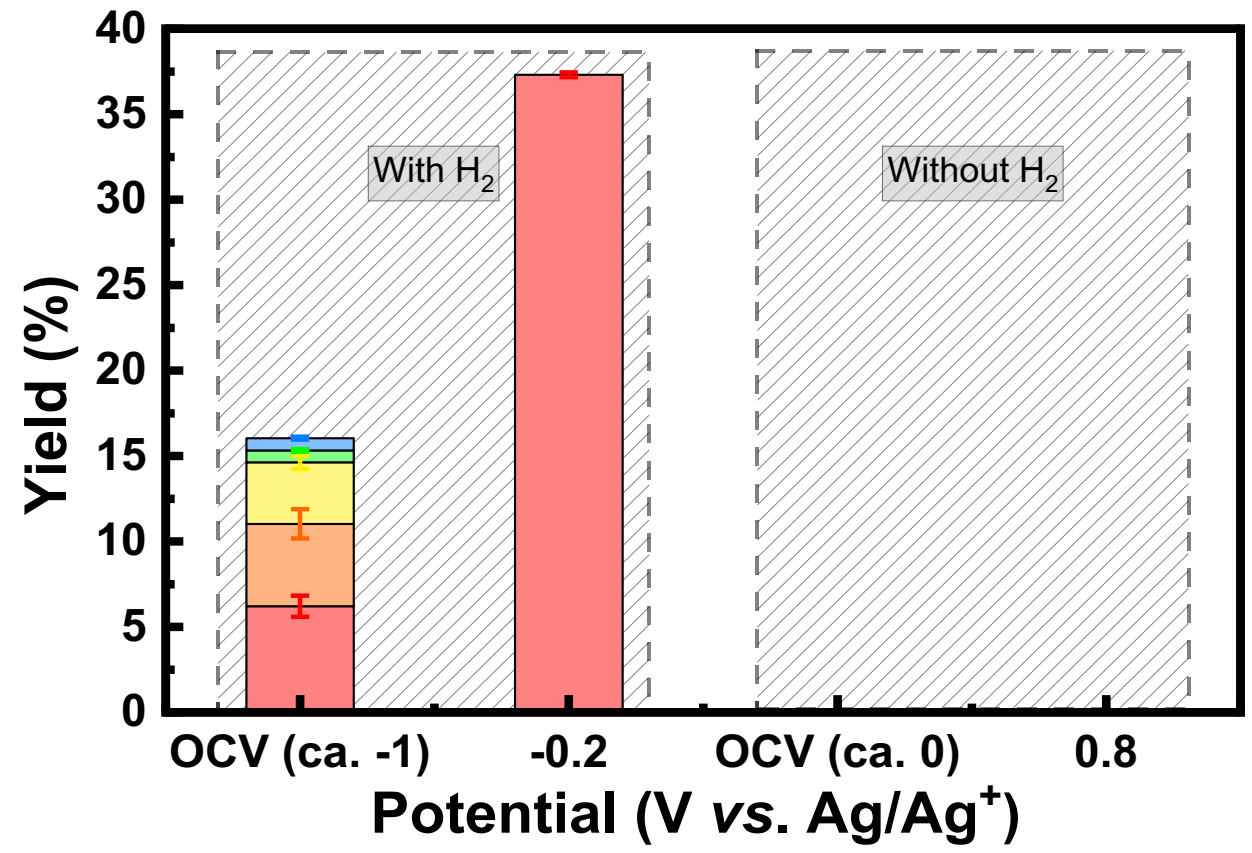

Figure S20. Control experiments showing that there is no product formation from 3-NS hydrogenation without a hydrogen gas feed (right) and comparison to the yield obtained in presence of $H_{2(g)}$ (left, data is reproduced from Figure 1 in the main text). The experimental setup and reaction conditions were similar as our standard conditions but without H_2 sparging. These conditions were tested both at E_{OCV} (which is at ca. 0 V in the absence of H_2) and at an applied positive potential that is more positive by +0.8 V compared to the E_{OCV} (i.e. the applied potential is +0.8 V *vs*. Ag/Ag$^+$), similarly to what we used in presence of $H_{2(g)}$. No hydrogenation product was observed at either OCV or at the applied positive potential in absence of $H_{2(g)}$.

Table S5. Water contents analysis of HPLC-grade MeCN and different reaction solutions. The water contents in working and counter chamber after reaction were both higher than the value before reaction (19.46 mM), which likely because of the trace amount water from hydrogen sparging.

Solution	Average water content[a] (ppm)	Concentration of water in solution[b] (mM)	Volume percent of water in solution[c] (%)
HPLC MeCN	142.6	6.30	0.01
Before reaction	440.2	19.46	0.04
Working chamber after OCV	1808.4	79.95	0.14
Counter chamber after OCV	1182.4	52.28	0.09
Working chamber after −0.2 V	1259.6	55.69	0.10
Counter chamber after −0.2 V	1090.4	48.21	0.09

[a] Average values based on three independent replicate measurements. [b] The values were determined with $c = \frac{n}{V} = \frac{m/M}{V}$, where m is the mass of the H_2O which was calculated by the water content in ppm multiplies mass of 20 mL solution (15.9296 g), M is the molar mass of H_2O (18.01528 g mol^{-1}), V is the volume of solution (20 mL). [c] The values were determined with $\frac{V_{water}}{V_{solution}} = \frac{m_{water}/\rho_{water}}{V_{solution}}$, where m is the mass of the H_2O which was calculated by the water content in ppm multiplies mass of 20 mL solution (15.9296 g), ρ is the density of H_2O (1.000 g mL^{-1}), $V_{solution}$ is the volume of solution (20 mL).

7. Testing catalyst stability when a positive potential is applied

7.1 Testing if original catalytic performance at OCV is recovered after an applied potential

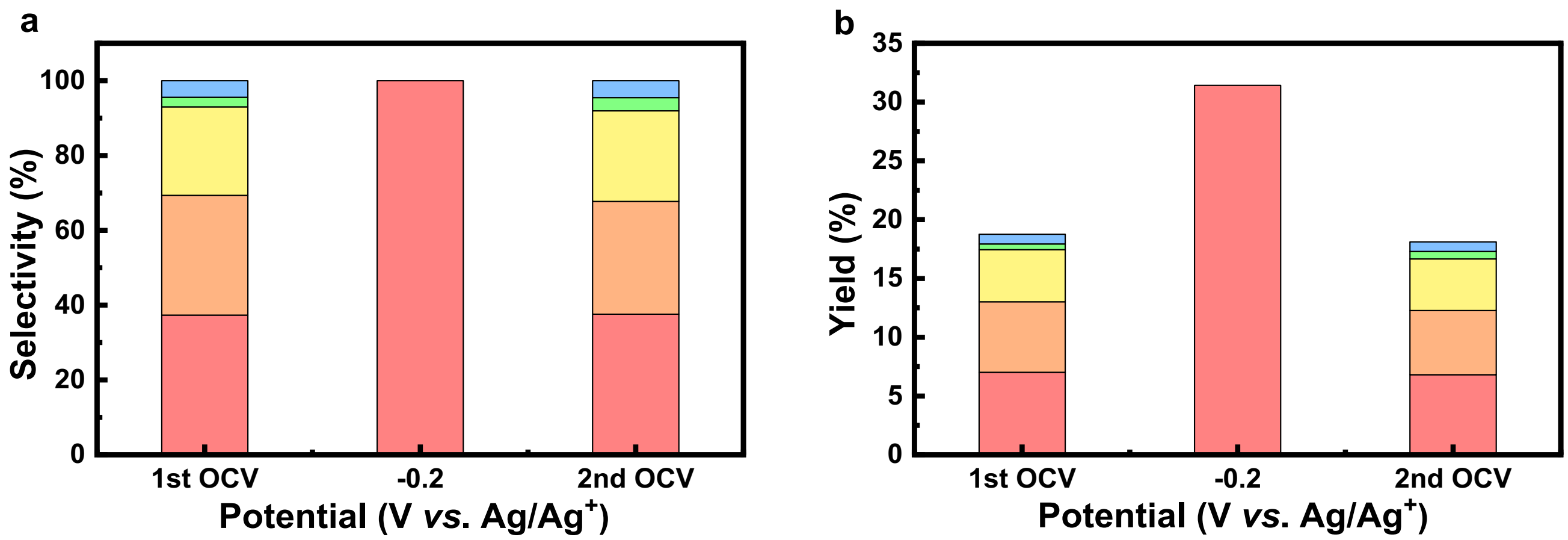

Figure S21. Results of a consecutive experiment of 3-NS hydrogenation run at OCV, then at −0.2 V, and then again at OCV with one and the same Pt electrode to check the stability of the catalyst when a positive potential is applied to it. Figures show (a) selectivity and (b) yield during the consecutive reactions. When the reaction was run at OCV for the second time, a similar catalytic performance was obtained as with the first reaction run at OCV. This suggests that the application of −0.2 V did not change the catalyst permanently.

7.2 Characterization of the as-prepared catalyst and the catalyst after reaction

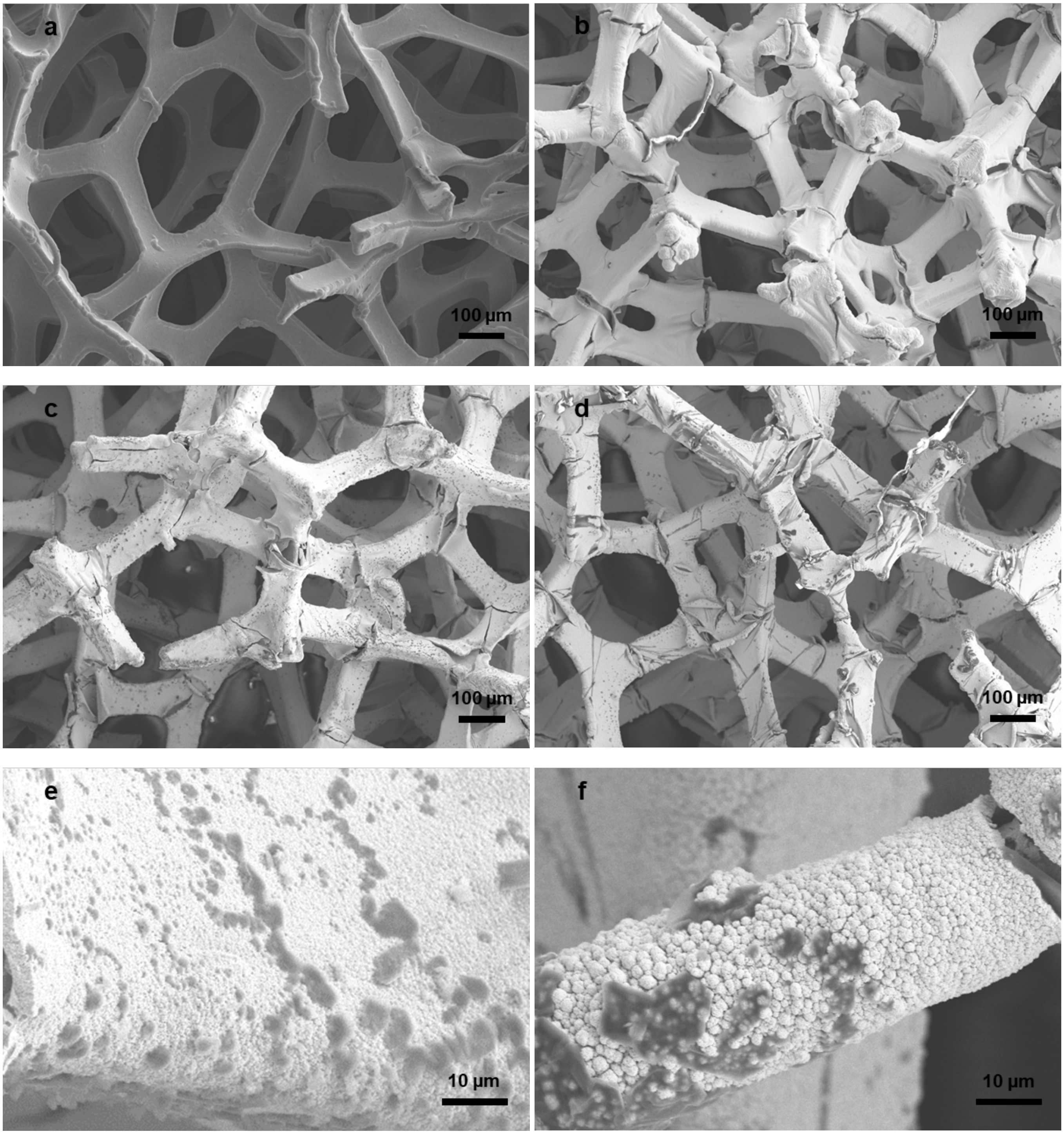

Figure S22. SEM images of (a) the bare Ni foam, (b) the Pt-coated Ni foam before catalysis, (c) and (e) the Pt-coated Ni foam after 3-NS hydrogenation at OCV at different magnifications, (d) and (f) the Pt-coated Ni foam after 3-NS hydrogenation at −0.2 V at different magnifications. The Pt surfaces after 3-NS hydrogenation both at OCV (c and e) and at −0.2 V (d and f) showed similar features, such as some dark spots and breaks in the coating. The data suggest that while there are some changes to the as-prepared catalyst upon 3-NS hydrogenation, the changes are similar at OCV and −0.2V.

Table S6. Elemental composition obtained from EDX analysis of the bare Ni foam, the Pt-coated Ni foam before catalysis, and Pt-coated Ni foam after 3-NS hydrogenation at OCV or −0.2 V. The data shown in this table are obtained from the average of the elemental composition measured at multiple individual spots on each sample. After catalysis both at OCV and −0.2 V, the data suggest that there is some F-content that is not previously contained in the as-prepared Pt on Ni foam. This F-content may stem from the supporting electrolyte $TBAPF_6$ used during catalysis. We speculate that the dark spots observed by SEM (Figure S22 c–f) may be linked to this F-content.

	C (%)	O (%)	Ni (%)	Pt (%)	F (%)
Ni foam	48.7	8.6	42.7	-	-
Pt coated Ni foam	56.1	24.3	4.1	15.5	-
after OCV reaction	57.8	12.5	5.8	12.4	11.5
after −0.2 V reaction	69.4	4.5	3.0	17.3	5.8

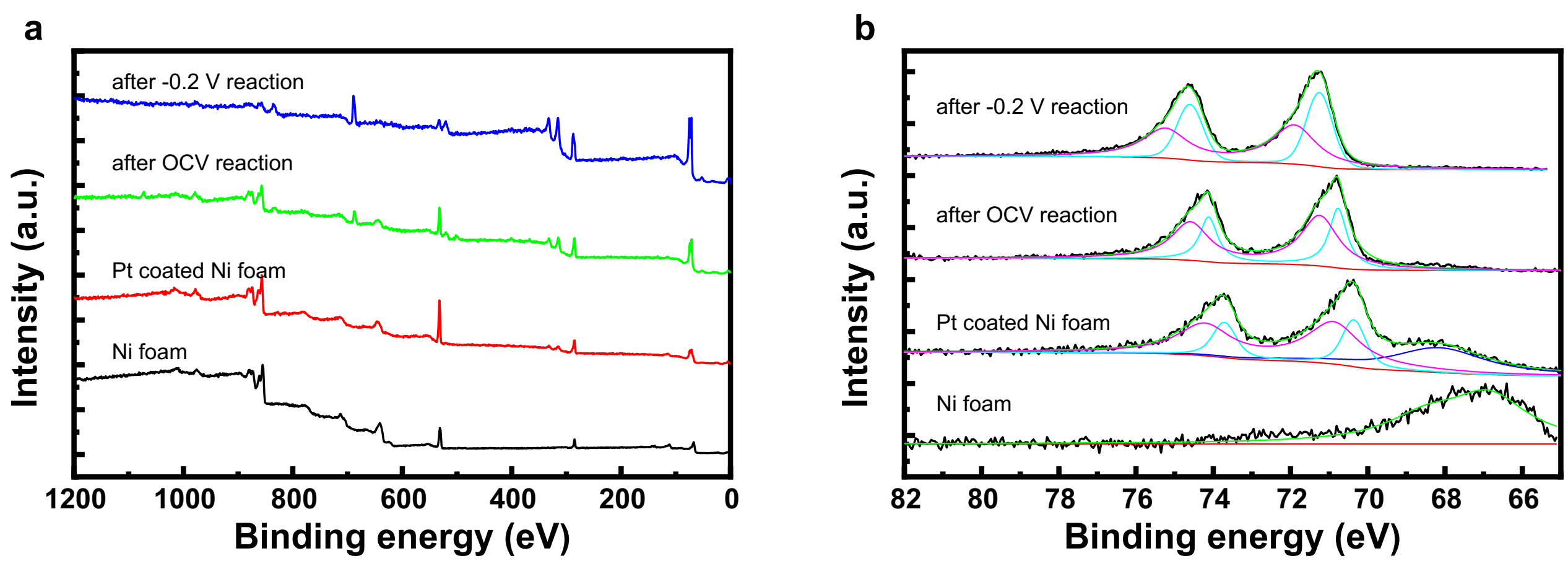

Figure S23. XPS (a) survey spectra and (b) Pt 4f spectra measured for the bare Ni foam, the Pt-coated Ni foam before catalysis, the Pt-coated Ni foam after 3-NS hydrogenation at OCV or −0.2 V (from bottom to top). The signal observed in the range of 66–68 eV in the Pt 4f spectra in (b) for the bare Ni foam is attributed to the Ni 3p peak.[29] The Pt-coated Ni foam before and after catalysis at OCV or −0.2 V showed the Pt $4f_{7/2}$ and Pt $4f_{5/2}$ signals at ~71 and ~74 eV, as expected for Pt metal.[29] The Pt 4f peaks obtained for Pt films after catalysis at OCV or at −0.2 V are only slightly shifted to higher binding energies compared to the as-synthesized catalyst suggesting that the catalyst is not substantially changed under reaction conditions.

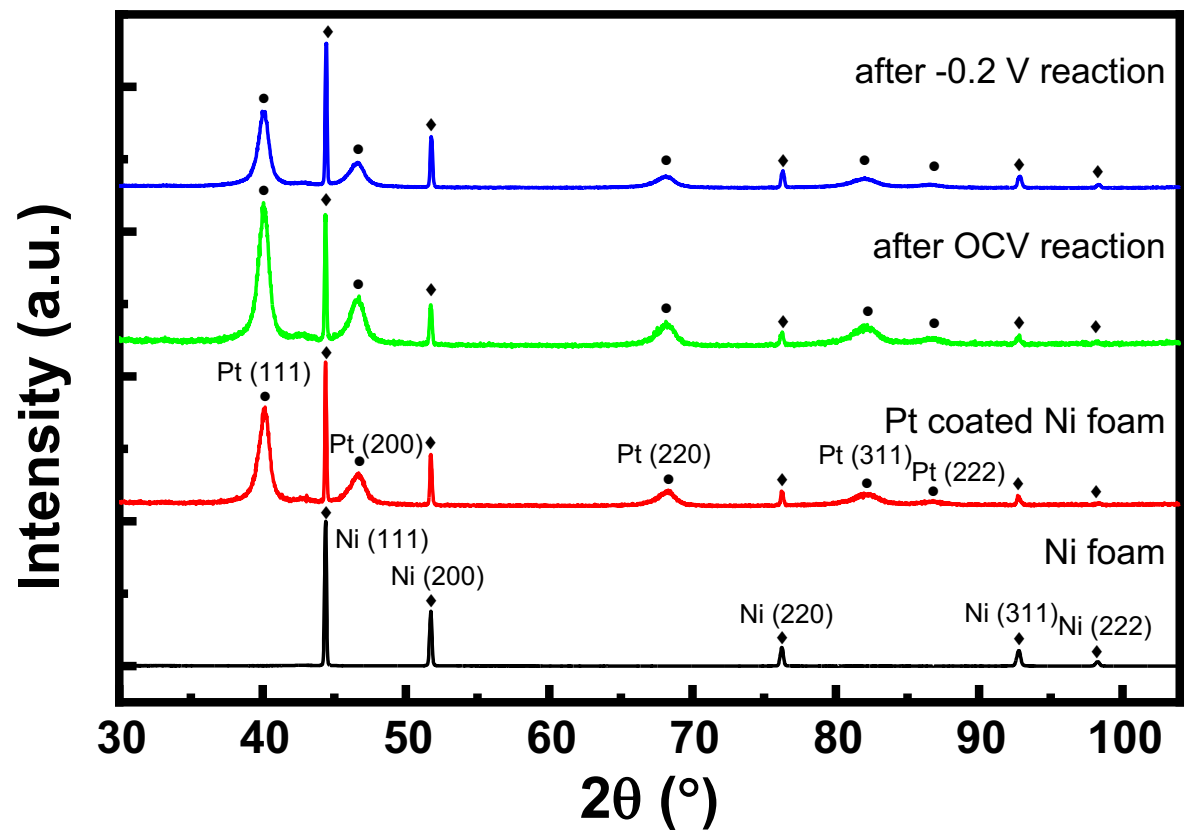

Figure S24. pXRD patterns of the bare Ni foam, the Pt-coated Ni foam before catalysis, the Pt-coated Ni foam after 3-NS hydrogenation at OCV or −0.2 V (from bottom to top). Peaks associated with Ni or Pt are designated with "♦" or "•", respectively. No new peaks were observed after 3-NS hydrogenation catalysis at either OCV or −0.2 V.

7.3 Testing the contribution to catalysis from possible leached components of the catalyst

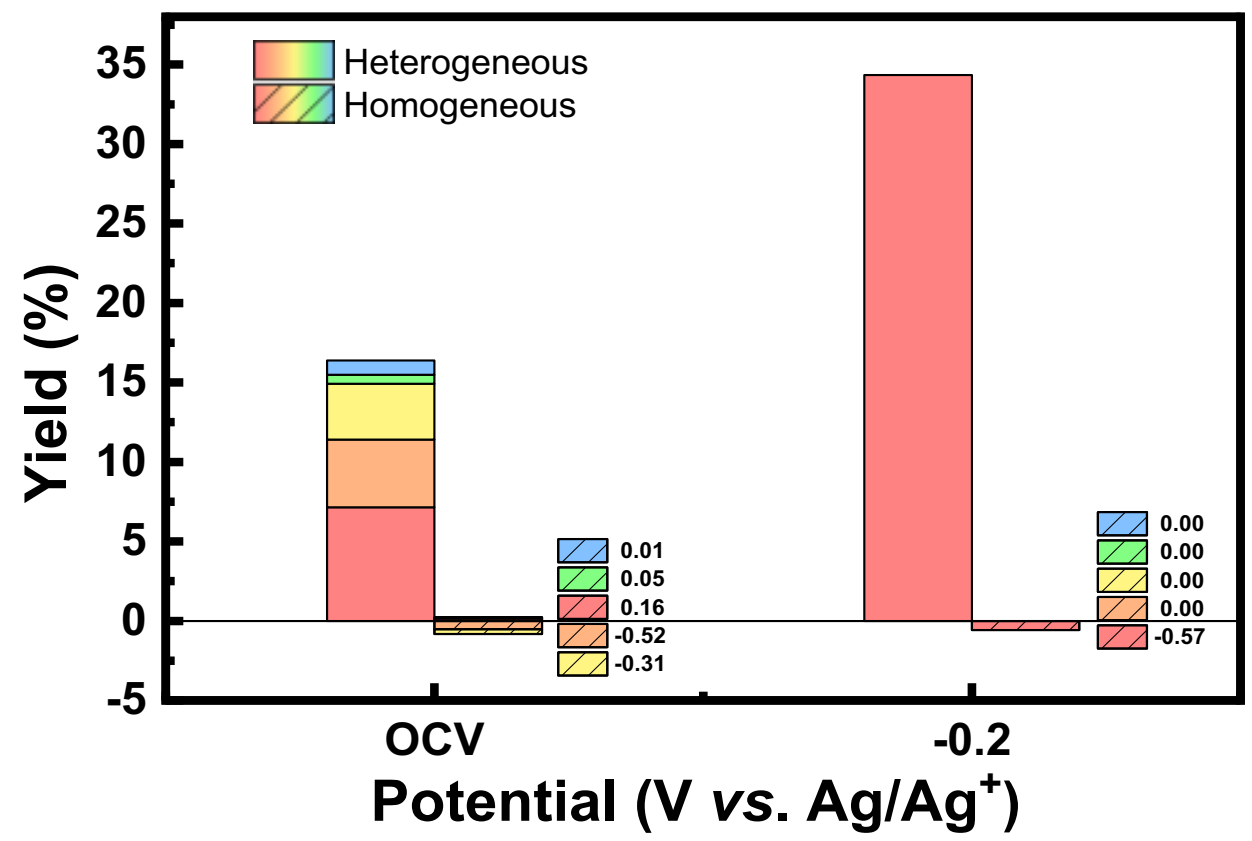

Figure S25. The contribution to catalysis from possible leached components of the Pt catalyst was tested by running first a standard experiment of 3-NS hydrogenation at either OCV or −0.2 V for 2 h with NMR quantification of the products formed (left columns, designated with “heterogeneous”). Then, the Pt electrode was removed from the setup and $H_{2(g)}$ was sparged through the reaction mixture for another 2 h. Then, the amount of product in solution was measured by NMR. The columns on the right (“homogeneous”) show the difference in the amount of product measured compared to what was measured after the first 2 h of catalysis in presence of Pt. The negligible change in amount of product showed that neither at OCV nor −0.2 V any potential leaching contributes significantly to the product formation observed herein.

8. The effect of the concurrent hydrogen oxidation reaction

8.1 Supporting data for experiments involving the addition of HPF_6

Table S7. Measured ^{MeCN}pH values of the reaction mixture before and after 3-NS hydrogenation at OCV or −0.2 V. Absolute ^{MeCN}pH values should be cautiously interpreted due to the uncertainty associated with such measurements in organic solvents and with respect to a MeCN-based pH scale, but general trends observed should be more accurate. Detailed comments about pH measurement can be found in SI section S2. The acidity of the solution in the working chamber after catalysis at −0.2 V is consistent with a concurrent HOR. The relative basicity of the counter chamber with respect to the working chamber could be the result of the HER and the separating Nafion-117 membrane that did not allow efficient proton transfer under these conditions.

	Before reaction	After reaction in working chamber	After reaction in counter chamber
OCV	5.55	8.10	5.42
−0.2 V		−0.88	13.35

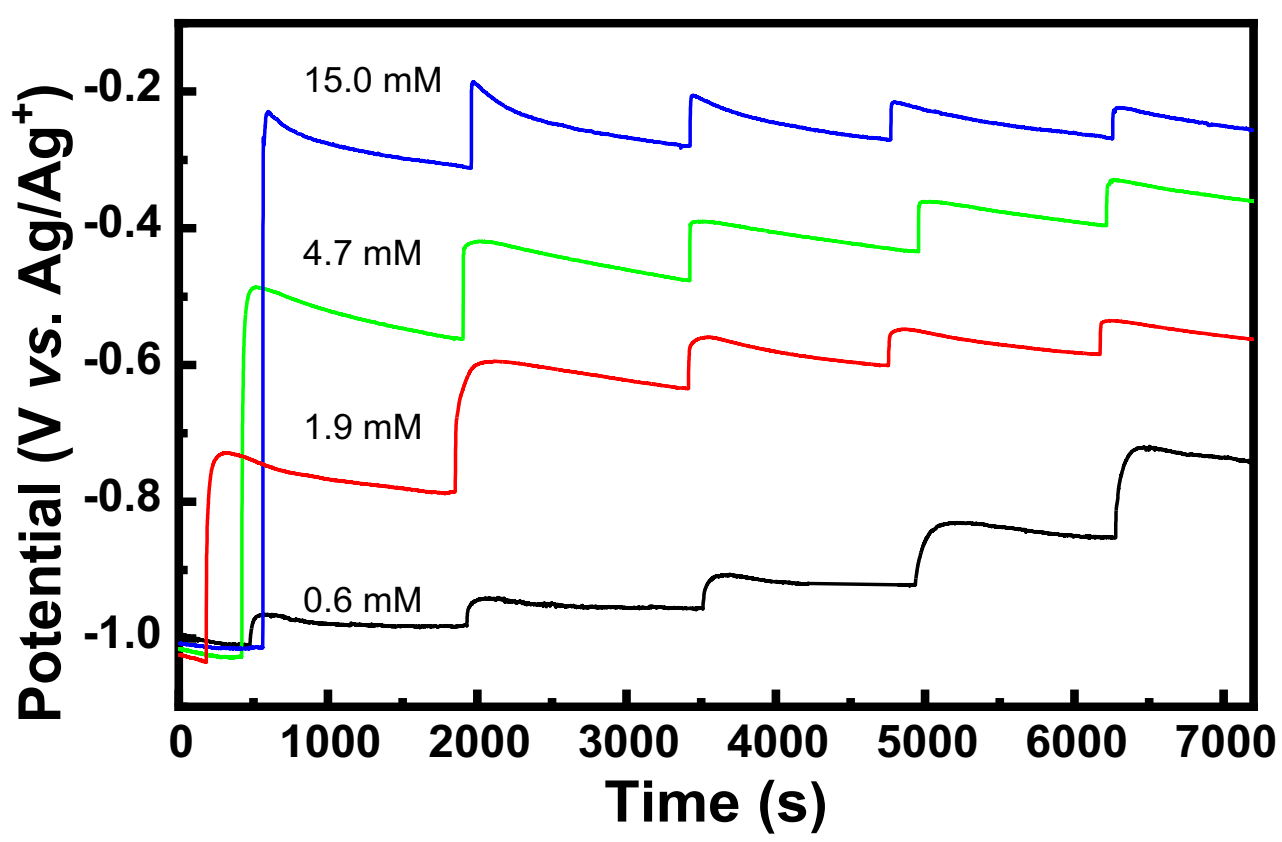

Figure S26. Measured E_{OCV} as a function of time for experiments of 3-NS hydrogenation and run at OCV while adding a fixed amount of HPF_6 in five portions. The resulting HPF_6 concentration in the solution from the total addition of all five portions are shown in numbers with each line in the plot. The addition of HPF_6 in multiple portions was done to be closer to the continuous proton production expected by a concurrent HOR.

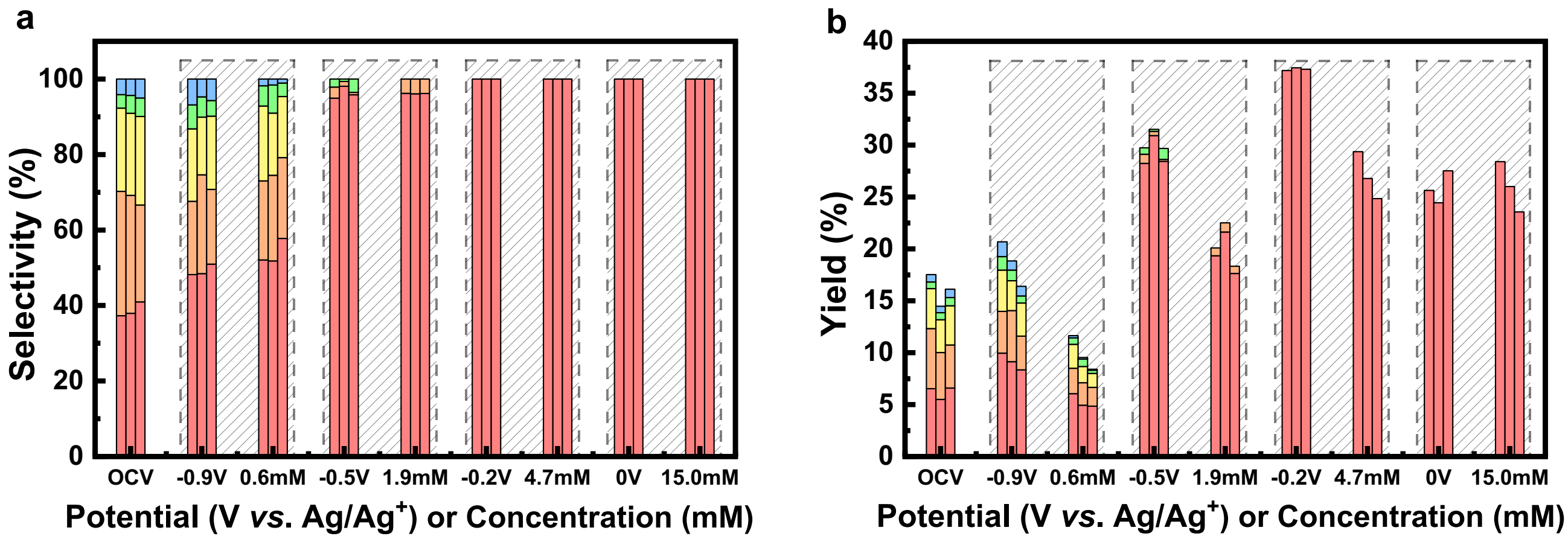

Figure S27. (a) Selectivity and (b) yield obtained in three independent replicate experiments of 3-NS hydrogenation at OCV with addition of HPF_6 and comparison to experiments done at different applied potential. The latter data is reproduced from Figure S16 and added here for comparison.

Table S8. Components of the reaction mixture used in standard experiments at OCV, experiments involving the addition of HPF_6, and two control experiments, where water, and $TBAPF_6$, or water, $TBAPF_6$, and TBAF were added to the reaction mixture. The solution of HPF_6 added contained 68.6% HPF_6, 30% H_2O, and 1.4% HF. The small amount of HF content is volatile. The control experiments were done to test whether the effects observed when HPF_6 was added were due to PF_6^-, H_2O, or F^-. The amount of H_2O, $TBAPF_6$, and TBAF in the control experiments were chosen such to match the expected H_2O, $TBAPF_6$, and TBAF content when a maximal amount of HPF_6 (15.0 mM) was added.

	Substrate	Supporting electrolyte	HPF_6 solution	$TBAPF_6$	H_2O	TBAF
OCV	100 mM 3-NS	100 mM $TBAPF_6$				
HPF_6 experiment	100 mM 3-NS	100 mM $TBAPF_6$	0.6, 1.9, 4.7, or 15.0 mM			
Control experiment	100 mM 3-NS	100 mM $TBAPF_6$		14.98 mM	55.52 mM	
Control experiment with TBAF	100 mM 3-NS	100 mM $TBAPF_6$		14.98 mM	53.05 mM	2.22 mM

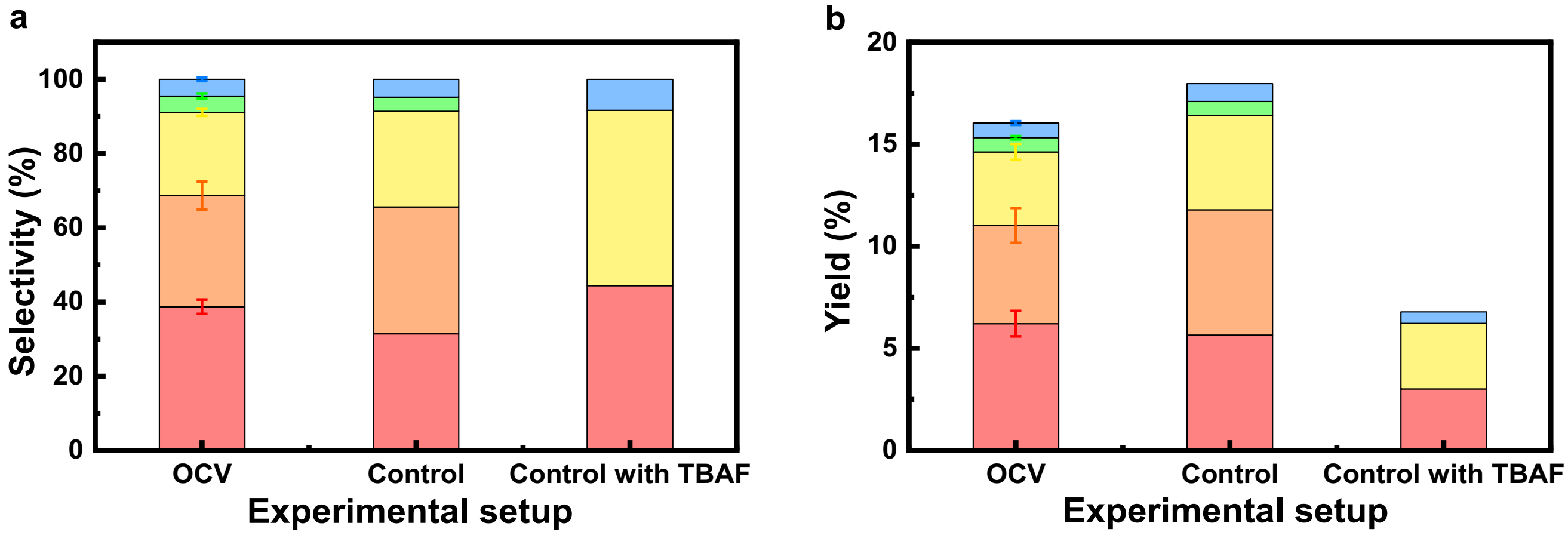

Figure S28. Results of the control experiments done to test whether effects observed when HPF_6 was added were due to PF_6^-, H_2O, or F^- and comparison to the data obtained for 3-NS hydrogenation under standard conditions at OCV (lest, data is reproduced from Figure 1 in the main text). One control experiment was done with water and $TBAPF_6$, and another one with water, $TBAPF_6$, and TBAF (also see Table S8): (a) Selectivity and (b) yield. The data show that the observed effects with HPF_6 are not due to added water or PF_6^- but attributable to the addition of H^+.

8.2 Isotopic labelling experiments with D_2O

Experiments with added D_2O were performed using a similar procedure as the standard 3-NS hydrogenation experiments (SI sections S3.3 and S3.4) using a solution of 100 mM $TBAPF_6$ and 30 mM D_2O in dry MeCN at both OCV and at an applied potential of −0.2 V. An aliquot of 100 µL was taken both before and after the reaction and analyzed by ^{1}H-NMR and GC-MS (Figures S29-S34 and Tables S9 and S10). For the GC-MS analysis the aliquot was treated beforehand with ca. 5 ml diethyl ether and the precipitated $TBAPF_6$ was removed using a syringe filter. The filtrate was further diluted with dry MeCN to a total volume of 20 ml (containing ca. 1 mM 3-NS). Mass spectra were analyzed for the relative amounts of D-labelled compounds with m/z of M+1 or M+2 versus the molecular ion peak with m/z of M.

To account for the relative amounts of M+1 and M+2 versus M obtained from the natural abundance of D, ^{13}C and ^{15}N isotopes, a control experiment was performed using a similar procedure as the standard 3-NS hydrogenation experiments (SI sections S3.3 and S3.4) using a solution of 100 mM $TBAPF_6$ and 30 mM MQ water (H_2O) in dry MeCN at an applied potential of −0.2 V. An aliquot of 100 µL was obtained both before and after the reaction and analyzed by ^{1}H-NMR and GC-MS (Figures S35-S37 and Table S11). For the GC-MS analysis the aliquot was treated beforehand with ca. 5 ml diethyl ether and the precipitated $TBAPF_6$ was removed using a syringe filter. The filtrate was further diluted with dry MeCN to a total volume of 20 ml (containing ca. 1 mM 3-NS).

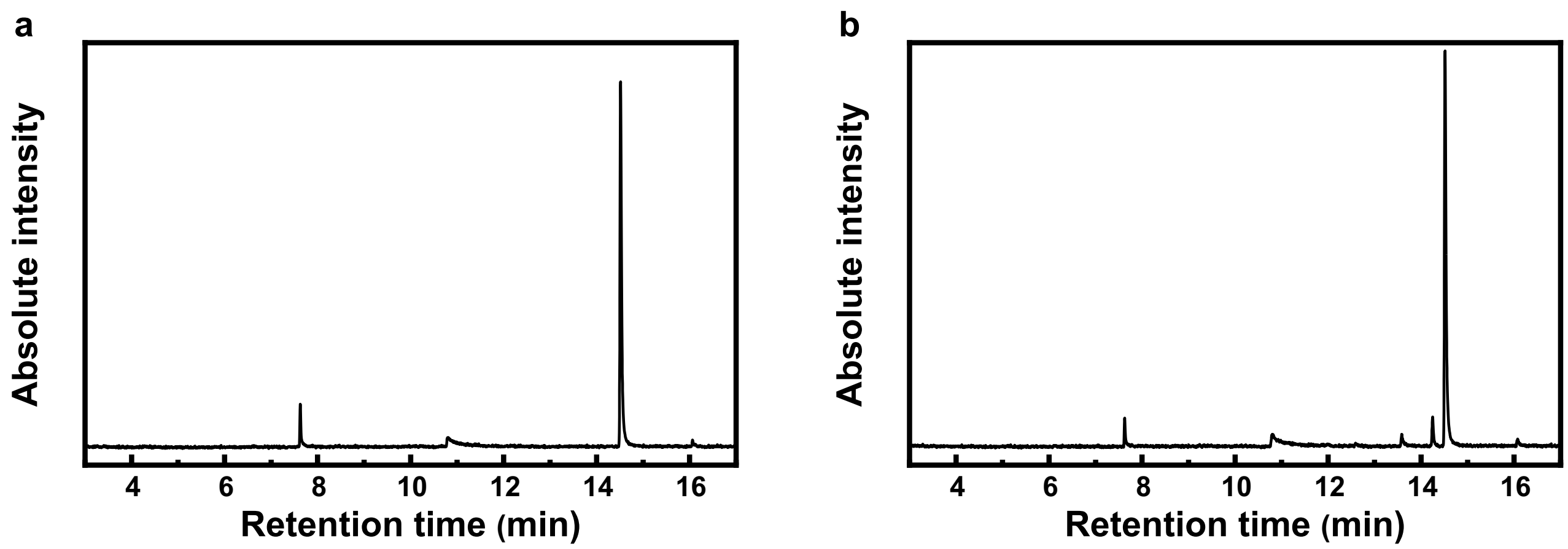

Figure S29. (a) GC-MS chromatogram at the beginning and (b) at the end of the reaction of the thermal 3-NS hydrogenation at OCV with 30 mM added D_2O.

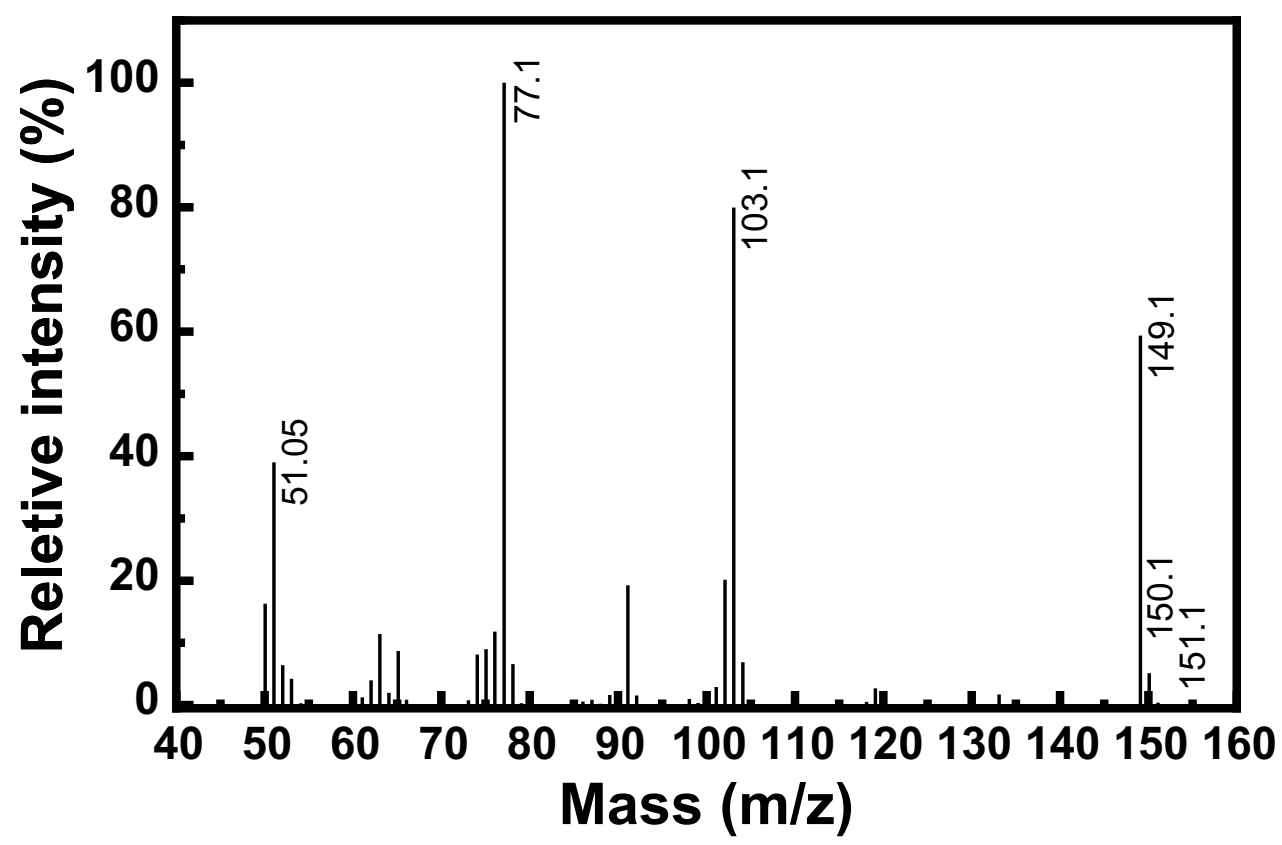

Figure S30. GC-MS mass spectrum of 3-NS (retention time 14.52 min) at the beginning the reaction of the thermal 3-NS hydrogenation at OCV with 30 mM added D_2O. Intensity values for M (m/z 149.1), M+1 (m/z 150.1), and M+2 (m/z 151.1) peaks of 3-NS are summarized in Table S9.

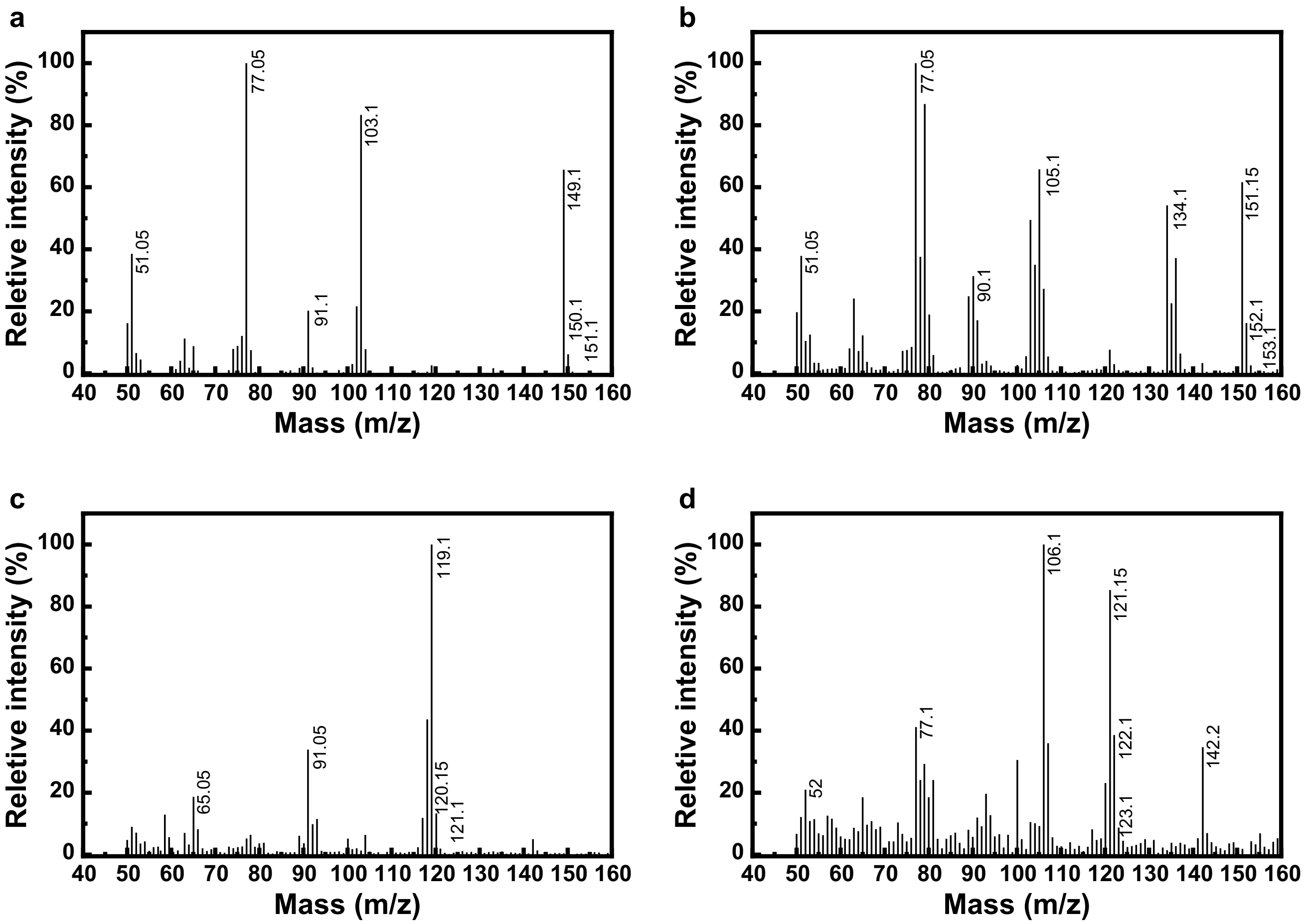

Figure S31. GC-MS mass spectra of (a) 3-NS (retention time 14.52 min), (b) 3-ENB (retention time 14.25 min), (c) 3-VA (retention time 13.58 min) and (d) 3-EA (retention time 12.59 min) at the end the reaction of the thermal 3-NS hydrogenation at OCV with 30 mM added D_2O. Intensity values for M, M+1, and M+2 peaks of each compound are summarized in Table S9.

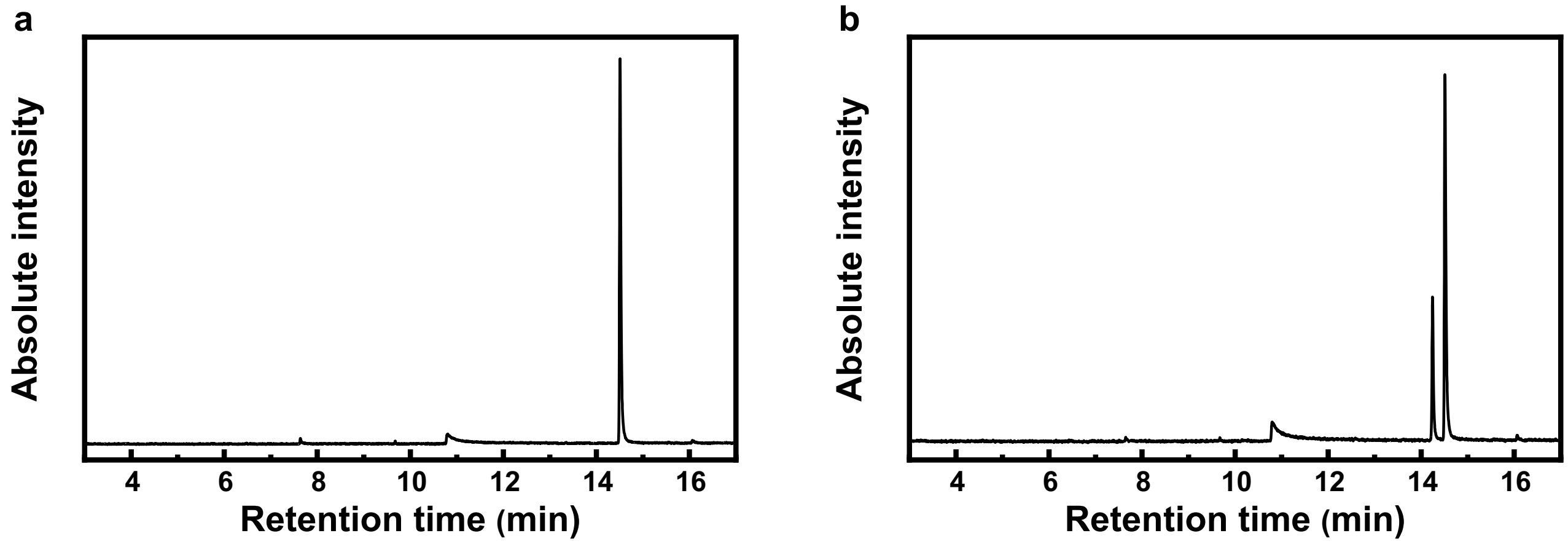

Figure S32. (a) GC-MS chromatogram at the beginning and (b) at the end of the reaction of the thermal 3-NS hydrogenation at −0.2 V with 30 mM added D_2O.

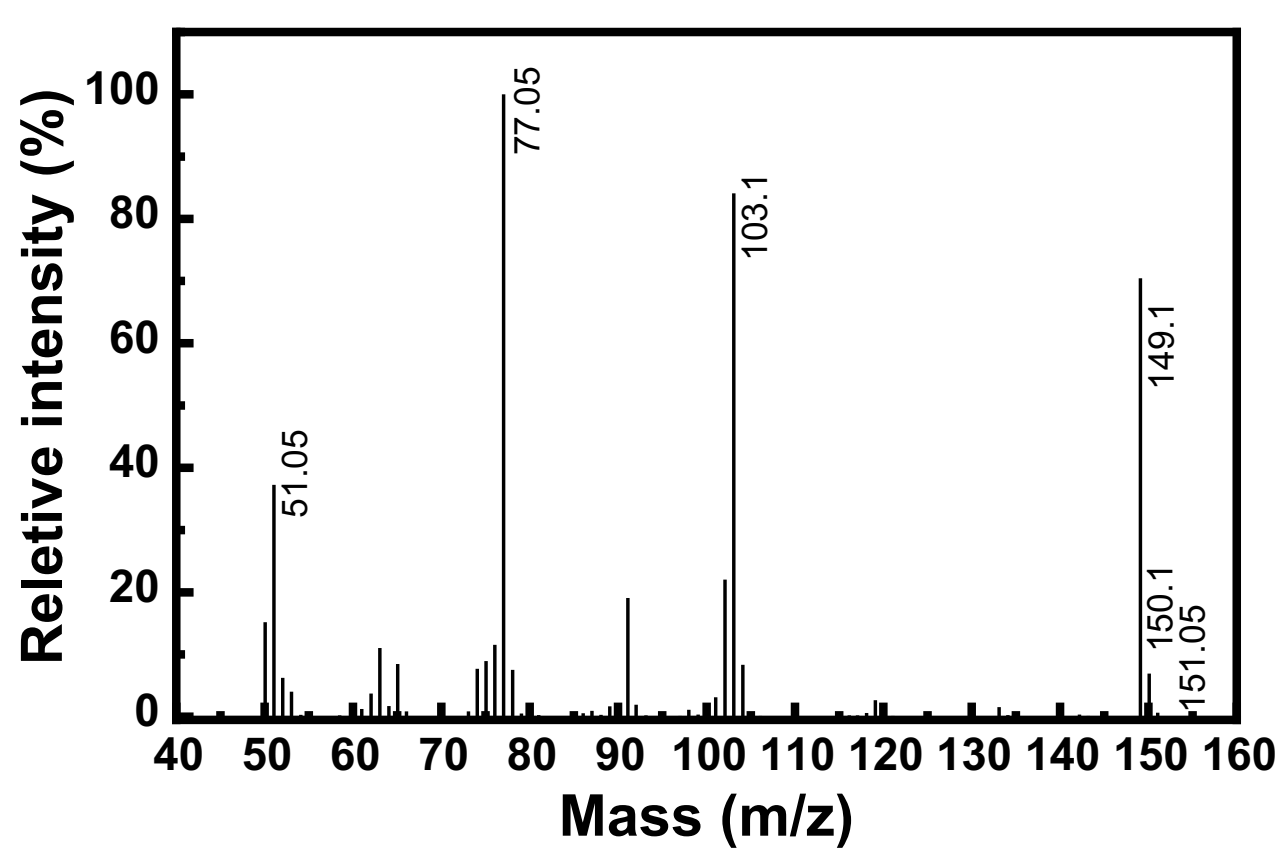

Figure S33. GC-MS mass spectrum of 3-NS (retention time 14.51 min) at the beginning the reaction of the thermal 3-NS hydrogenation at −0.2 V with 30 mM added D_2O. Intensity values for M (m/z 149.1), M+1 (m/z 150.1), and M+2 (m/z 151.1) peaks of 3-NS are summarized in Table S10.

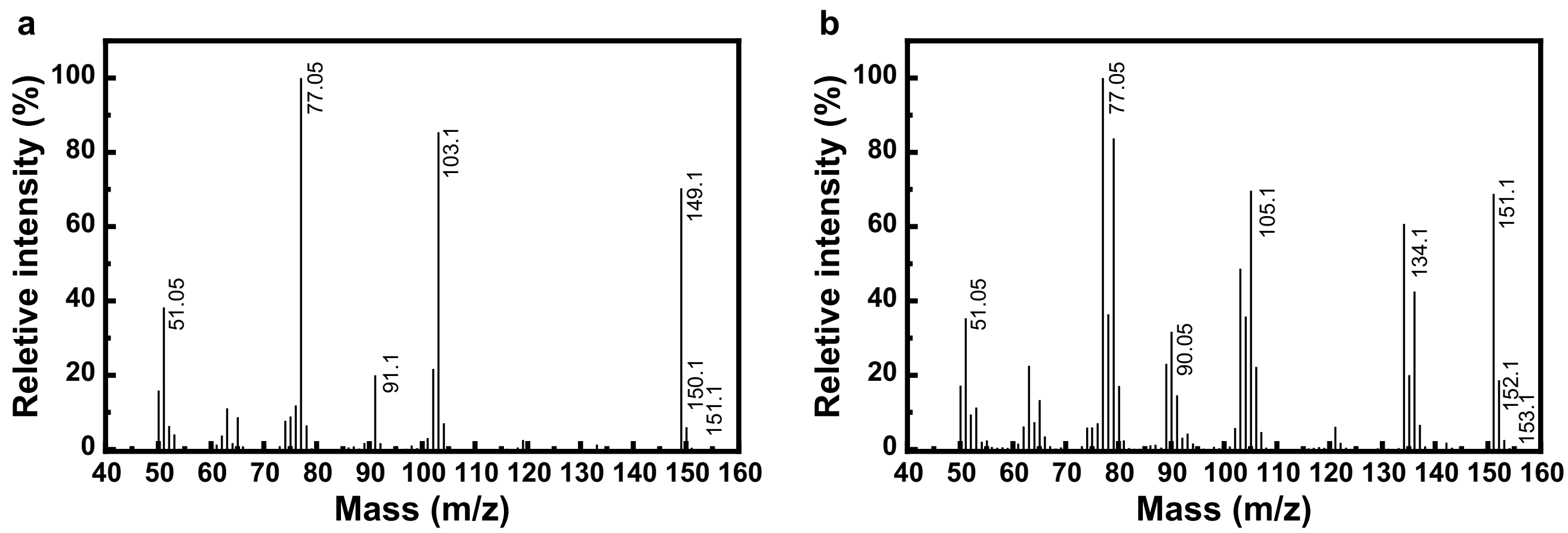

Figure S34. GC-MS mass spectra of (a) 3-NS (retention time 14.51 min) and (b) 3-ENB (retention time 14.24 min) at the end the reaction of the thermal 3-NS hydrogenation at −0.2 V with 30 mM added D_2O. Intensity values for M, M+1, and M+2 peaks of each compound are summarized in Table S10.

Table S9. Obtained GC-MS absolute intensities of 3-NS in the beginning and at the end of the reaction as well as of 3-ENB, 3-VA and 3-EA at the end of the reaction of the thermal 3-NS hydrogenation at OCV with 30 mM added D_2O.

Sampling	Compound	Mass (m/z)	Absolute intensity	Relative intensity to M (%)
Reaction beginning	3-NS	M (149)	132127	
		M+1 (150)	11457	**8.67**
		M+2 (151)	855	**0.65**
Reaction end	3-NS	M (149)	152311	
		M+1 (150)	14294	**9.38**
		M+2 (151)	1364	**0.90**
	3-ENB	M (151)	5566	
		M+1 (152)	1465	**26.32**
		M+2 (153)	231	**4.15**
	3-VA	M (119)	8913	
		M+1 (120)	1186	**13.31**
		M+2 (121)	178	**2.00**
	3-EA	M (121)	1396	
		M+1 (122)	631	**45.20**
		M+2 (123)	143	**10.24**

Table S10. Obtained GC-MS absolute intensities of 3-NS in the beginning and at the end of the reaction as well as of 3-ENB at the end of the reaction of the thermal 3-NS hydrogenation at −0.2 V with 30 mM added D_2O.

Sampling	Compound	Mass (m/z)	Absolute intensity	Relative intensity to M (%)
Reaction beginning	3-NS	M (149)	374897	
		M+1 (150)	32082	**8.56**
		M+2 (151)	2686	**0.72**
Reaction end	3-NS	M (149)	171495	
		M+1 (150)	16908	**9.86**
		M+2 (151)	1572	**0.92**
	3-ENB	M (151)	34106	
		M+1 (152)	9254	**27.13**
		M+2 (153)	1287	**3.77**

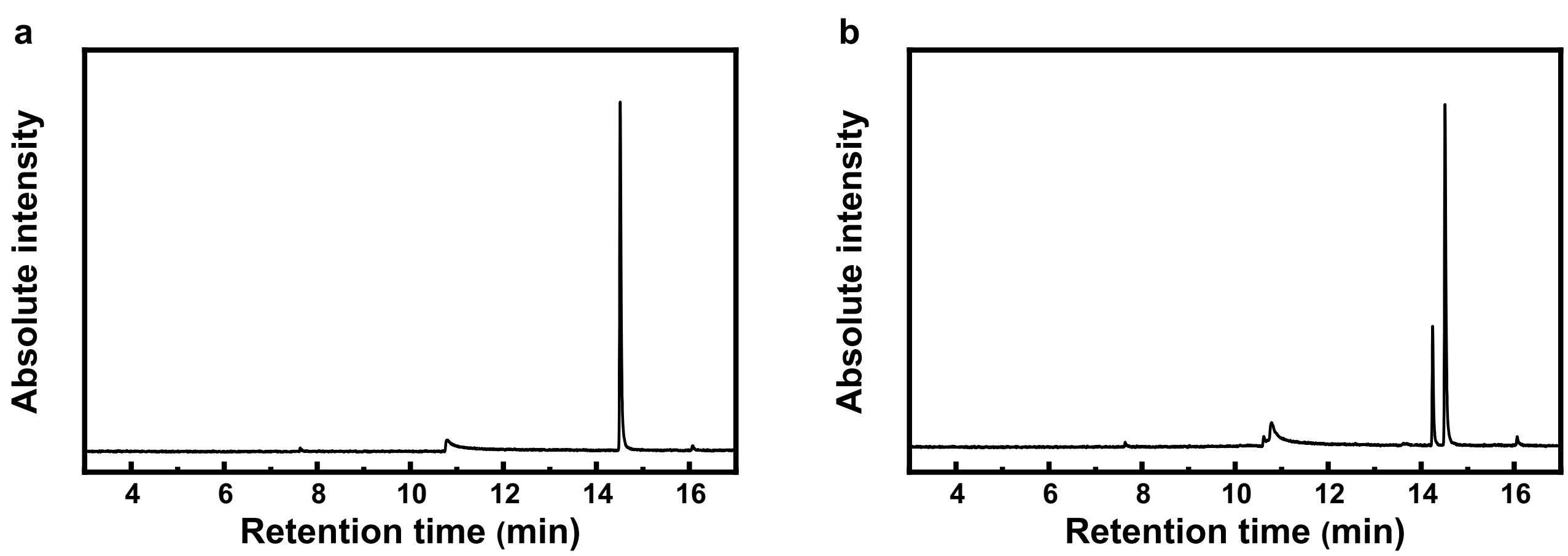

Figure S35. (a) GC-MS chromatogram at the beginning and (b) at the end of the reaction of the thermal 3-NS hydrogenation at −0.2 V with 30 mM added MQ water (H_2O).

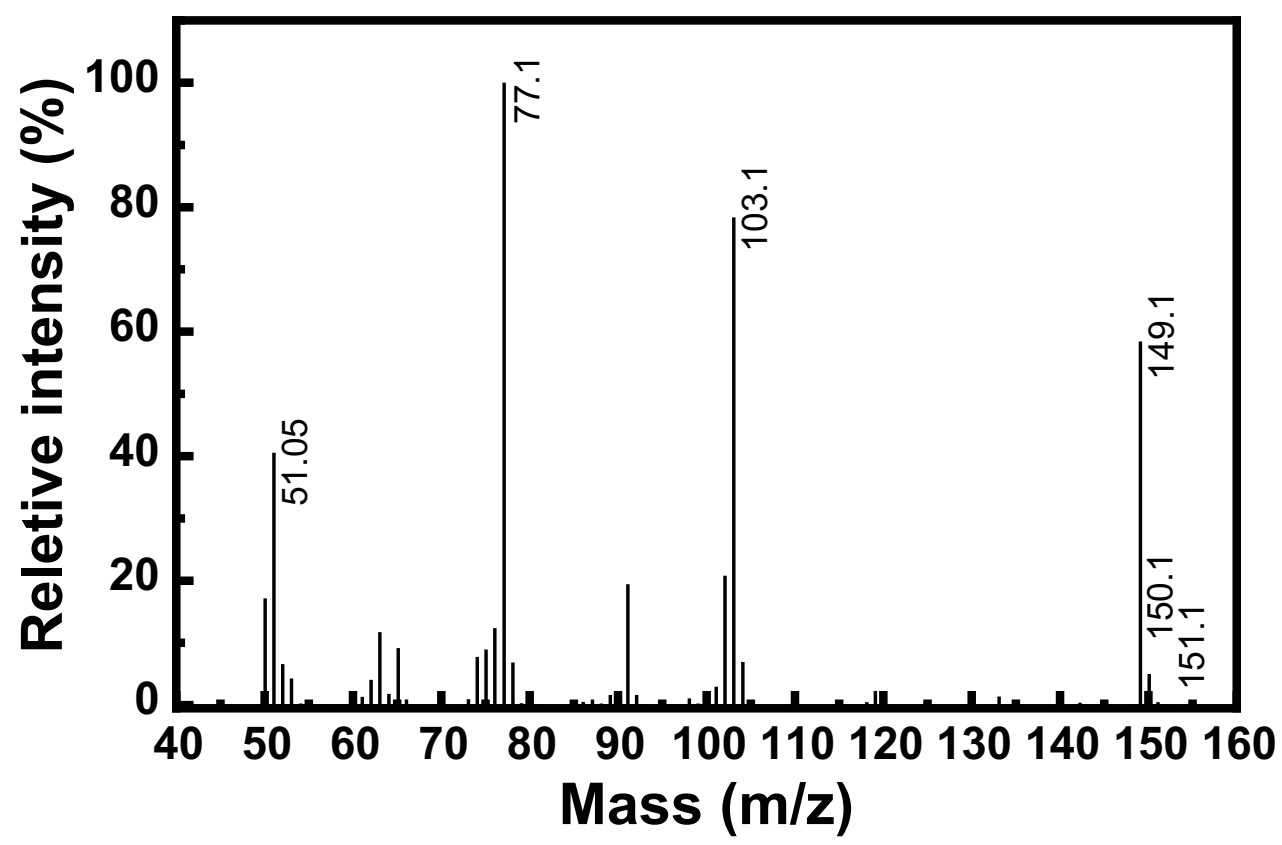

Figure S36. GC-MS mass spectrum of 3-NS (retention time 14.51 min) at the beginning the reaction of the thermal 3-NS hydrogenation at −0.2 V with 30 mM added MQ water (H_2O). Intensity values for M (m/z 149.1), M+1 (m/z 150.1), and M+2 (m/z 151.1) peaks of 3-NS are summarized in Table S11.

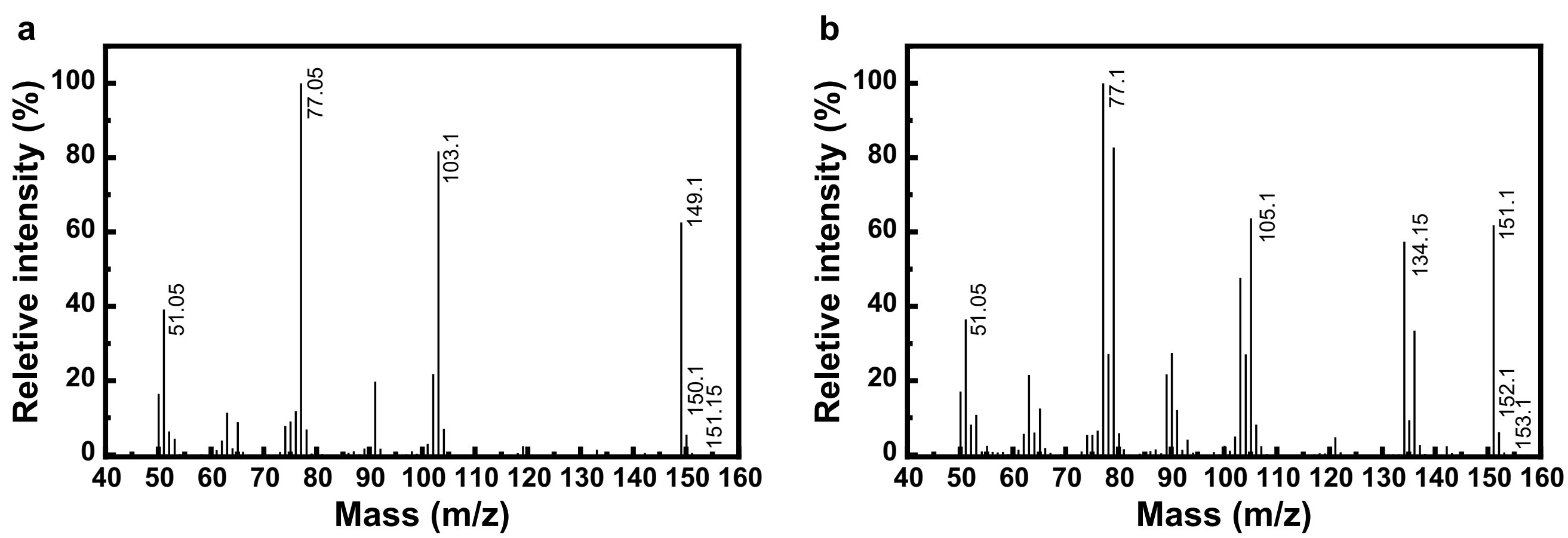

Figure S37. GC-MS mass spectra of (a) 3-NS (retention time 14.51 min) and (b) 3-ENB (retention time 14.24 min) at the end the reaction of the thermal 3-NS hydrogenation at −0.2 V with 30 mM added MQ water (H_2O). Intensity values for M, M+1, and M+2 peaks of each compound are summarized in Table S11.

Table S11. Obtained GC-MS absolute intensities of 3-NS in the beginning and at the end of the reaction as well as of 3-ENB at the end of the reaction of the thermal 3-NS hydrogenation at −0.2 V with 30 mM added MQ water (H_2O).

Sampling	Compound	Mass (m/z)	Absolute intensity	Relative intensity to M (%)
Reaction beginning	3-NS	M (149)	216526	
		M+1 (150)	18623	**8.60**
		M+2 (151)	1742	**0.80**
Reaction end	3-NS	M (149)	190220	
		M+1 (150)	16639	**8.75**
		M+2 (151)	1625	**0.85**
	3-ENB	M (151)	38642	
		M+1 (152)	3831	**9.91**
		M+2 (153)	431	**1.12**

8.3 The effect of H^+ from the Nafion-117 membrane

Table S12. Experimentally measured ^{MeCN}pH values in the working chamber after 3-NS hydrogenation, calculated ^{H2O}pH values were based on a pH scale in water and based on what would be obtained in the working chamber assuming that all experimentally measured oxidation current comes from HOR and generates H^+, experimentally measured ^{MeCN}pH values after addition of a certain amount HPF_6 into the working chamber solution prior to 3-NS hydrogenation. Absolute ^{MeCN}pH values should be cautiously interpreted due to the uncertainty associated with such measurements in organic solvents and with respect to a MeCN-based pH scale, but general trends observed should be more accurate. Detailed comments about pH measurement can be found in SI section S2. The amount of HPF_6 added was based on the assumption that all observed oxidation current is associated with HOR, and that HPF_6 completely dissociates. We tentatively attribute the higher ^{MeCN}pH values obtained by addition of HPF_6 (last column) versus the calculated ^{H2O}pH values based on observed HOR currents (middle column) to an incomplete dissociation of HPF_6 and/or differences in activity of H^+ in water versus MeCN. The large discrepancy between calculated ^{H2O}pH values (middle column) and experimentally measured ^{MeCN}pH values after catalysis (first column on the left) suggests that there is an additional proton source apart from HOR under catalytic conditions.

Experimentally measured ^{MeCN}pH values in the working chamber after catalysis at different applied potentials given in brackets	Calculated ^{H2O}pH values in the working chamber at different applied potentials given in brackets assuming that all experimentally measured oxidation current comes from HOR and generates H^+	Experimentally measured ^{MeCN}pH values in the working chamber after addition of a certain amount HPF_6 (resulting concentration given in brackets), at OCV
−0.02 (−0.9 V)	3.21 (−0.9 V)	4.15 (0.6 mM)
−0.32 (−0.5 V)	2.73 (−0.5 V)	3.62 (1.9 mM)
−0.88 (−0.2 V)	2.33 (−0.2 V)	3.03 (4.7 mM)
−1.15 (0 V)	1.82 (0 V)	2.53 (15.0 mM)

Table S13. Experimentally measured ^{MeCN}pH values in the working chamber before and after contacting the Pt electrode with 3-NS in the absence of H_2 at OCV (−0.2 V) or at 0.6 V (+0.8 V *vs*. OCV). Absolute ^{MeCN}pH values should be cautiously interpreted due to the uncertainty associated with such measurements in organic solvents and with respect to a MeCN-based pH scale, but general trends observed should be more accurate. Detailed comments about pH measurement can be found in SI section S2. In the absence of H_2, no product formation was observed and no HOR can occur. The table shows that the ^{MeCN}pH value was substantially lowered after 2 h at 0.6 V even in the absence of H_2.

	Before reaction	After reaction in working chamber
OCV	5.12	5.80
0.6 V		1.73

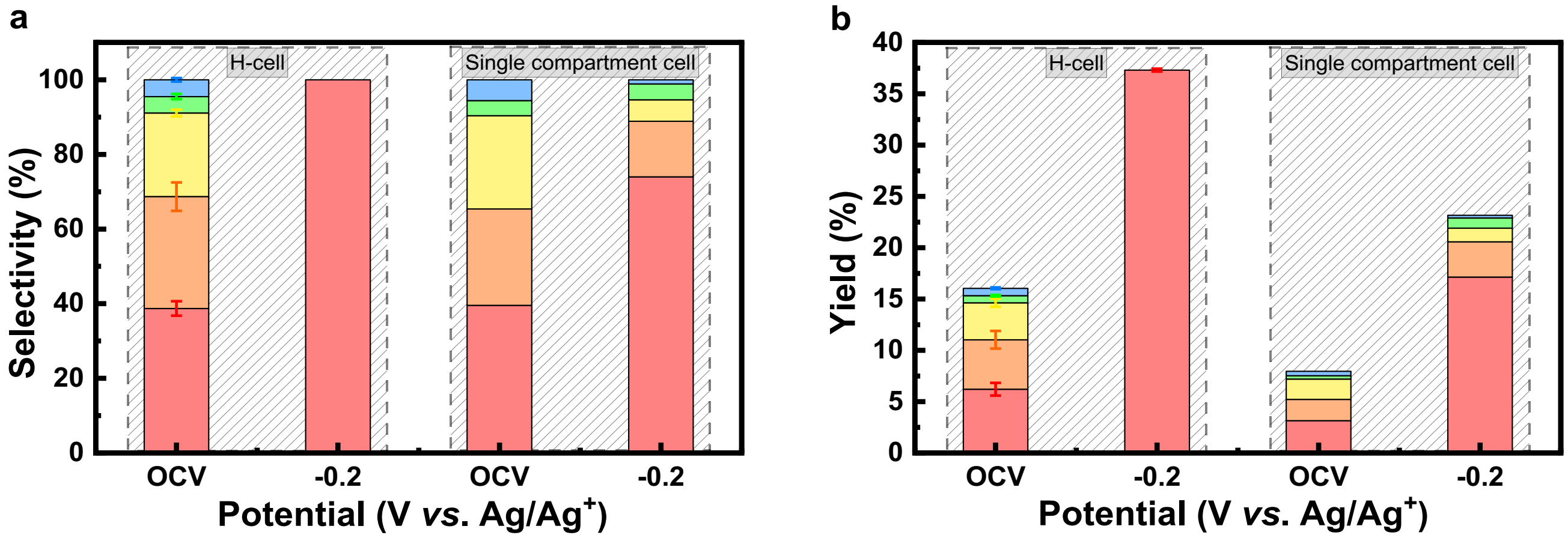

Figure S38. Comparison of the (a) selectivity and (b) yield obtained from the 3-NS hydrogenation done in either an H-cell (left, data is reproduced from Figure 1 in the main text) or a single compartment cell (right). Experimental conditions used in the two setups were similar apart from the lack of a separating membrane in the single compartment cell.

Table S14. Experimentally measured ^{MeCN}pH values before 3-NS hydrogenation and experimentally measured ^{MeCN}pH values and electric charge after the 3-NS hydrogenation at OCV or −0.2 V in an H-cell or a single compartment cell. Absolute ^{MeCN}pH values should be cautiously interpreted due to the uncertainty associated with such measurements in organic solvents and with respect to a MeCN-based pH scale, but general trends observed should be more accurate. Detailed comments about pH measurement can be found in SI section S2.

		Single compartment cell		H-cell		
	^{MeCN}pH value before reaction	^{MeCN}pH values after reaction	Electric charge (C)	^{MeCN}pH values after reaction, measured in the working chamber	^{MeCN}pH values after reaction, measured in the counter chamber	Average electric charge (C) from three independent replicate experiments
OCV	5.55	7.63		8.10	5.42	
−0.2 V		7.58	36.99	−0.88	13.35	9.04 ± 1.76

9. Mechanistic investigation using EC-SEIRAS

9.1 General considerations

Surface-enhanced infrared absorption spectro-electrochemistry (EC-SEIRAS) was measured on a Bruker VERTEX 80 fourier transform spectrometer equipped with a photovoltaic liquid N_2-cooled MCT (HgCdTe) detector under continuous purified air purge supplied by a purge gas generator (Peak Scientific Instruments PG14L). SEIRA spectra were collected using the VeeMax III attenuated total reflectance (ATR) accessory (PIKE Technologies) and a commercial air-tight J2 spectroelectrochemical infrared (IR) cell (Jackfish SEC) with a Si face-angled crystal. The incident IR beam was p-polarized with a manual precision ZnSe polarizer (PIKE Technologies) and the beam was filtered before the detector with a Bruker F321-H optical filter ($< 4000\ cm^{-1}$). IR spectra were collected at $2\ cm^{-1}$ resolution and 32 scans per spectrum with an aperture set to the largest value (8 mm) to maximize signal without saturation. Fourier transformation was done using the Mertz phase correction and Norton-Beer, Medium apodization function with a phase resolution of 32 and a zerofilling factor of 4. Bruker OPUS software was used for IR data collection and analysis. All SEIRA spectra measured herein were analyzed using OriginPro 2023. The spectra were fitted with a baseline correction or simply aligned as indicated in the respective figure captions.

Potentials were applied using a BioLogic SP-50e potentiostat during EC-SEIRAS measurements. The working electrode was the gold film on the Si ATR crystal. A leakless Ag/AgCl electrode (3.4 M KCl, ET069, eDAQ Pty Ltd) was used as a reference electrode. The Ag/AgCl electrode was calibrated with respect to $K_3[Fe(CN)_6]/K_4[Fe(CN)_6]$ (=161 mV *vs*. Ag/AgCl at 25 °C).[30] A Pt coil (DekResearch) was used as a counter electrode for the electropolishing and the Pt electrodeposition step and a graphite rod (DekResearch) was used for EC-SEIRAS measurements involving 3-NS. Electrochemical experiments were performed in degassed solvent by sparging the solution with $N_{2(g)}$ for >10 min. Figure S39 shows the spectroelectrochemical cell and setup used for EC-SEIRAS measurements involving 3-NS and H_2-flow.

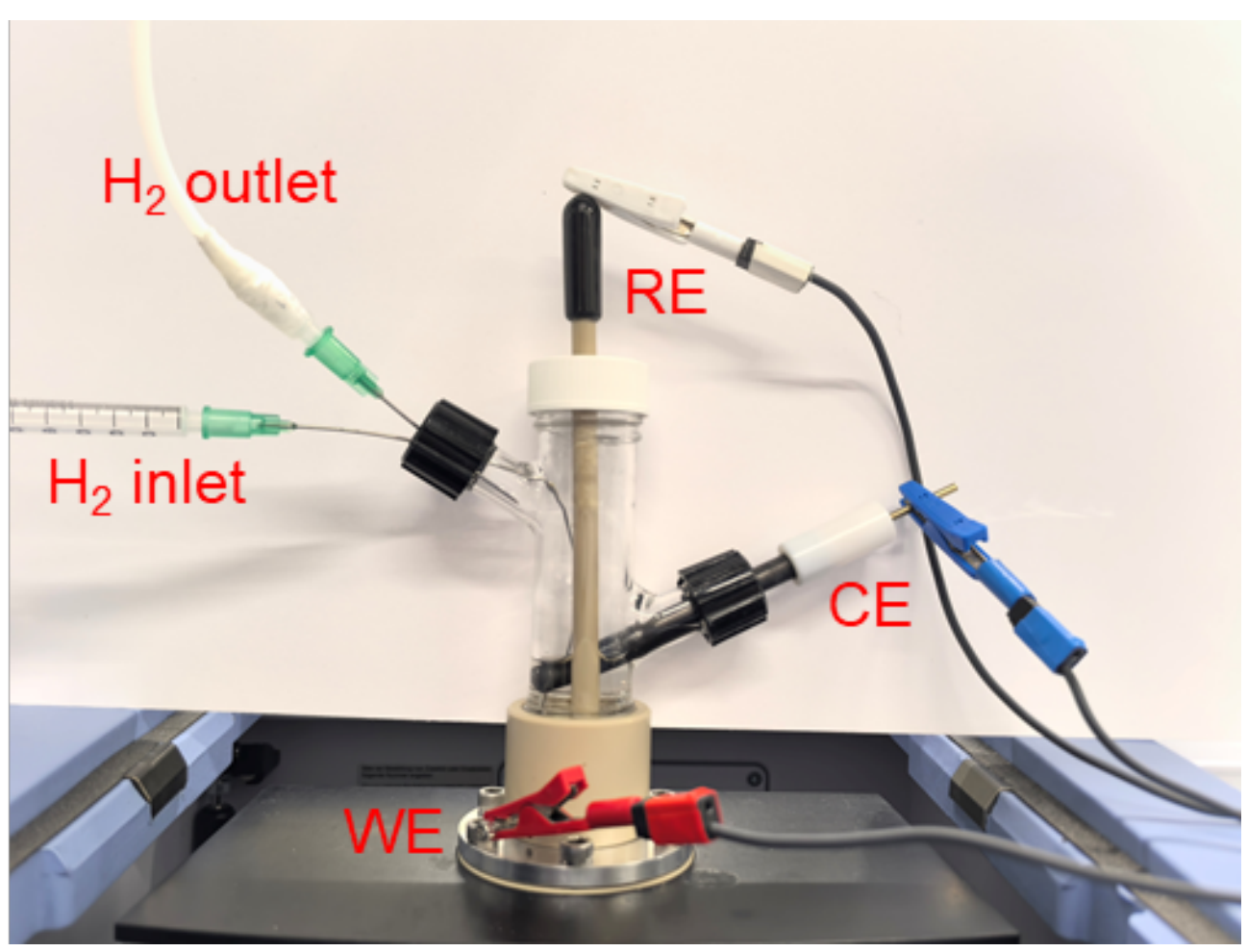

Figure S39. Photograph of the spectroelectrochemical cell and setup used for EC-SEIRAS measurements during 3-NS hydrogenation.

9.2 Preparation of the gold film and the Pt overlayer film

The gold film was prepared on a Si ATR crystal, similarly to our earlier report.[31] Briefly, the Si ATR crystal was first polished using a 0.05 μm alumina powder, then sonicated in acetone and MQ water for 5 min each, and finally rinsed several times with MQ water. After the Si crystal surface was dried with a lens tissue, a gold layer with a thickness of ~50 nm was deposited on the Si crystal with a Leica EM ACE600 sputter coater. The sputtering was performed at 30 mA under argon plasma at a pressure of 0.025 mbar to obtain a gold film. The thickness of the gold film was controlled by a quartz crystal microbalance (QCM).

Subsequently, a previously reported electrochemical method was adapted and used to remove the impurities from the surface of the sputtered gold layer and to obtain better signal-to-noise ratios in SEIRA spectra.[31,32] For that, the spectroelectrochemical cell was assembled and filled with 7.5 ml of a 300 mM sodium acetate buffer solution (pH = 6). The pH value of the acetate buffer solution was adjusted before use by addition of a solution of 0.1 M CH_3COOH until a pH value pH ≈ 6 was reached as monitored by a Mettler Toledo FiveEasy pH meter F20 with a LE 409 sensor. Cyclic voltammetry (CV) was then performed between −0.35 V and +0.30 V *vs*. Ag/AgCl at a scan rate of 100 mV s^{-1} for 30 cycles. A freshly prepared gold film was used for each experiment.

A pinhole-free Pt overlayer film was prepared on the electropolished Au by an electrochemical galvanostatic deposition method following a reported procedure.[33] A fresh solution of 4 mM $H_2PtCl_6{\cdot}6H_2O$ and 700 mM Na_2HPO_4 in MQ water was added to the spectroelectrochemical cell and sparged with $N_{2(g)}$, and the OCV was recorded. Then, a constant current of −176.9 µA cm^{-2} was applied for 120 s. Afterwards, the prepared film was washed twice with MQ water, then twice with MeCN, and finally once with dry MeCN.

9.3 Characterization of the prepared Pt film on Au

The electropolished Au film and the deposited Pt film on Au were characterized by CV and CO adsorption, which provided information on chemical composition, the electrochemically active surface area (ECSA), roughness, thickness, and the absence of pinholes in the Pt film.

9.3.1 Characterization by CV

To characterize the films by CV, the electropolished Au and the freshly deposited Pt films were washed twice with copious amount of MQ water. Then, a solution of 100 mM $HClO_4$ in MQ water was added. Then, a CV was recorded between −0.35 V and +1.35 V *vs*. Ag/AgCl at 100 mV s^{-1} in 100 mM $HClO_4$ (Figure S40a). During the forward scan with onset potentials of ca. +1.0 V and ca. +0.90 V the formation of Au oxide as well as of Pt oxide can be observed, respectively.[33] The reduction peak of Au oxide[33] at +0.73 V *vs*. Ag/AgCl obtained in the backwards scan on the bare Au is absent for the CV data obtained for the deposited Pt film. Instead, the CV obtained for the deposited Pt shows a reduction peak of the Pt oxide at +0.17 V.[33] We previously reported similar CV data for the Au film.[31] Our data show that Pt has been successfully deposited on Au and suggest (combined with the CO data described below) that a pinhole-free Pt film was obtained by the galvanostatic deposition method described above. We note that this characterization by CV involved the application of very positive potentials in a $HClO_4$ solution, which may lead to the oxidation and dissolution of the films and the adsorption of ClO_4^- anions. For this reason, the CVs were carried out as separate experiments with films of Au and Pt that were then not further used for other EC-SEIRAS measurements involving 3-NS. Hence, freshly prepared films were used for each experiment.

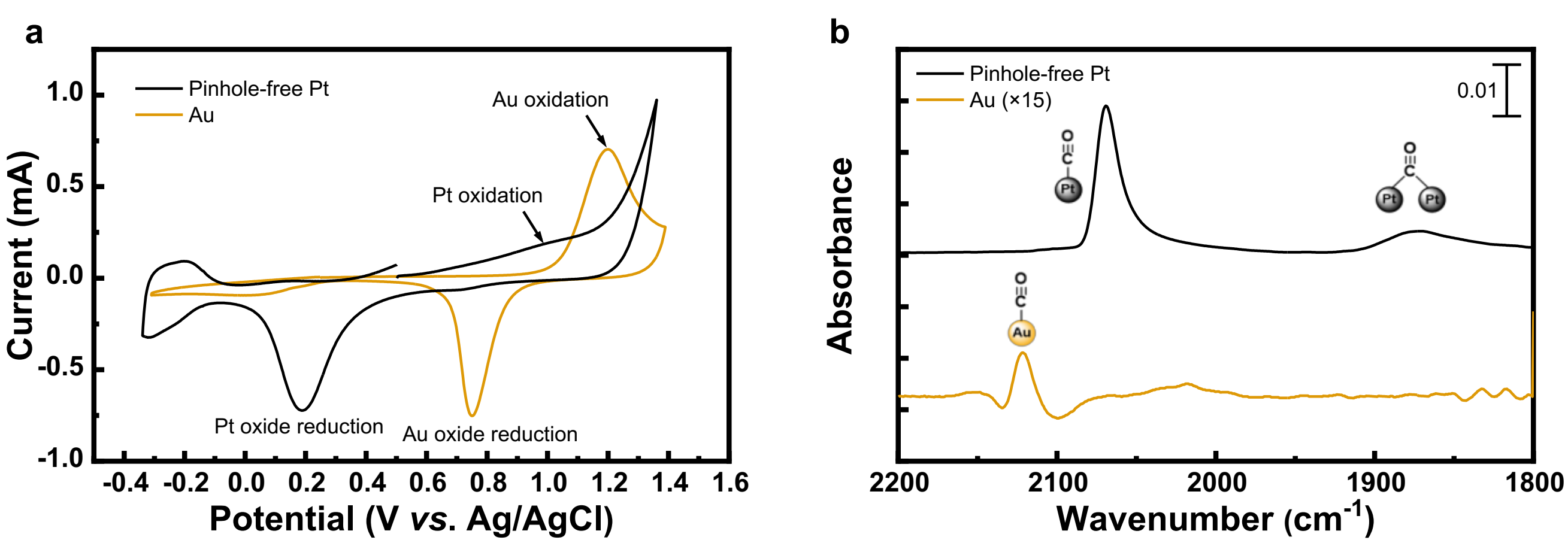

Figure S40. (a) CVs for the pinhole-free Pt (black line) and Au films (yellow line) in 100 mM $HClO_4$ measured with a scan rate of 100 mV s^{-1}. (b) SEIRA spectra of CO adsorbed on the pinhole-free Pt film (black line, top) and on Au film (yellow line, bottom). We had previously reported similar data for another bare Au film prepared by similar methods.[31]

9.3.2 Determination of the electrochemically active surface area, roughness, and thickness

The geometric area of the exposed films is A_{geo} = 0.636 cm^2. The ECSA of the Au and Pt films were determined by integrating the reduction peak of the corresponding metal oxides (921.5 µC for Au and 1277 µC for Pt) and dividing it by the theoretical charge required to reduce one monolayer of the metal oxide per cm^2 (400 µC cm^{-2} for Au[34] and 440 µC cm^{-2} for Pt[35]). We obtained an ECSA of 2.29 cm^2 for Au and 2.90 cm^2 for Pt. The roughness factor was calculated by dividing the ECSA with A_{geo} and was found to be ca. 4 for Au and ca. 5 for Pt.

The thickness (d) of the Pt film was estimated based on the charge passed during the galvanostatic deposition of a four-electron transfer reduction reaction assuming 100% Faradaic yield:

$$\mathrm{Pt}^{4+} + 4e^{-} \rightarrow \mathrm{Pt}^{0} \qquad \text{eq S1}$$

Using eq S2 we obtained a thickness of 5.0 nm for the Pt film. This equals to ca. 26 monolayers based on the lattice constant of 3.91 Å assuming a face-centered cubic (fcc) crystal structure and two atomic layers per unit cell in one direction.

$$\mathrm{d\ (cm)} = \frac{I\,(A) \cdot t\,(s) \cdot M\,(g\ mol^{-1})}{A_{geo}\,(cm^{2}) \cdot z \cdot \rho\,(g\ cm^{-3}) \cdot F\,(C\ mol^{-1})} \qquad \text{eq S2}$$

where I is the applied deposition current (−112.5 µA), t is the deposition time (120 s), M is the molar mass of Pt (195.09 g mol^{-1}), A_{geo} is the geometric surface area of the exposed metal film (0.636 cm^2), z is the number of electrons needed for the reduction reaction (4 for eq S1), ρ is the density of the Pt film (21.45 g cm^{-3}) and F is the Faraday constant (96485 C mol^{-1}).

9.3.3 Characterization by CO adsorption

The Au and Pt films were further characterized by SEIRAS measurements of CO adsorbed on the films. CO shows characteristic adsorption modes and vibration frequencies on different surfaces, thereby providing evidence for the chemical composition of a film and the possible presence of pinholes.[31,33,36] The cell containing 100 mM $HClO_4$ was sparged with three balloons of $CO_{(g)}$ in the fume hood, then SEIRA spectra were recorded at OCV (Figure S40b). The SEIRA spectrum of the Au film showed a band at 2125 cm^{-1}, which can be attributed to a terminal (atop) coordination of CO on Au.[33] For the Pt film, two bands at 2070 and 1873 cm^{-1} were observed, which can be assigned to the terminal and bridged-bound CO on Pt, respectively.[33] The absorption band at 2125 cm^{-1} that is characteristic of CO adsorption on Au is absent for the deposited Pt film. This, together with the CV analysis described above, suggests that the Pt film is pinhole-free.

9.4 Experimental procedures for EC-SEIRAS measurements involving 3-NS

9.4.1 EC-SEIRAS experiments of 3-NS on Pt in the absence of H_2 as a function of applied potential

The spectroelectrochemical cell was assembled and a Pt film on Au was prepared and washed as described above. After adding dry MeCN, a SEIRA background spectrum was recorded. Then, 7.4 mL of a 100 mM solution of $TBAPF_6$ in dry MeCN was added to the spectroelectrochemical cell and the OCV was recorded. After waiting 15 min, a potential of +0.65 V *vs*. Ag/AgCl (+0.80 V *vs*. OCV) was applied for 3 min and a background SEIRA spectrum was recorded for the following SEIRAS experiments. The applied positive potential helped to minimize contributions from the MeCN solvent in later SEIRAS measurements. Then, 104.5 µL of 3-NS was added to the solution to obtain a 100 mM final concentration, and a SEIRA spectrum (Figure S41) as well as the OCV were recorded simultaneously. Positive potentials were applied in the potential range between the measured OCV value (typically ca. −0.15 V *vs*. Ag/AgCl) to +1.0 V *vs*. OCV (typically +0.85 V *vs*. Ag/AgCl) in increments of 50 mV (forward scan), and then back towards less positive applied potentials to OCV (reverse scan). Each potential was held for 90 s during which SEIRA spectra were recorded every 45 s. Then, another SEIRA background spectrum was recorded for an experiment run consecutively after this experiment, which is described in SI section S9.4.2. The recorded background spectrum is needed to obtain the desorption spectrum of weakly-bound 3-NS species in the next experiment. This procedure was reproduced three times in individual replicate experiments, each with a freshly prepared Pt film. The obtained EC-SEIRAS data as a function of applied potential are shown and discussed in SI section S9.7.

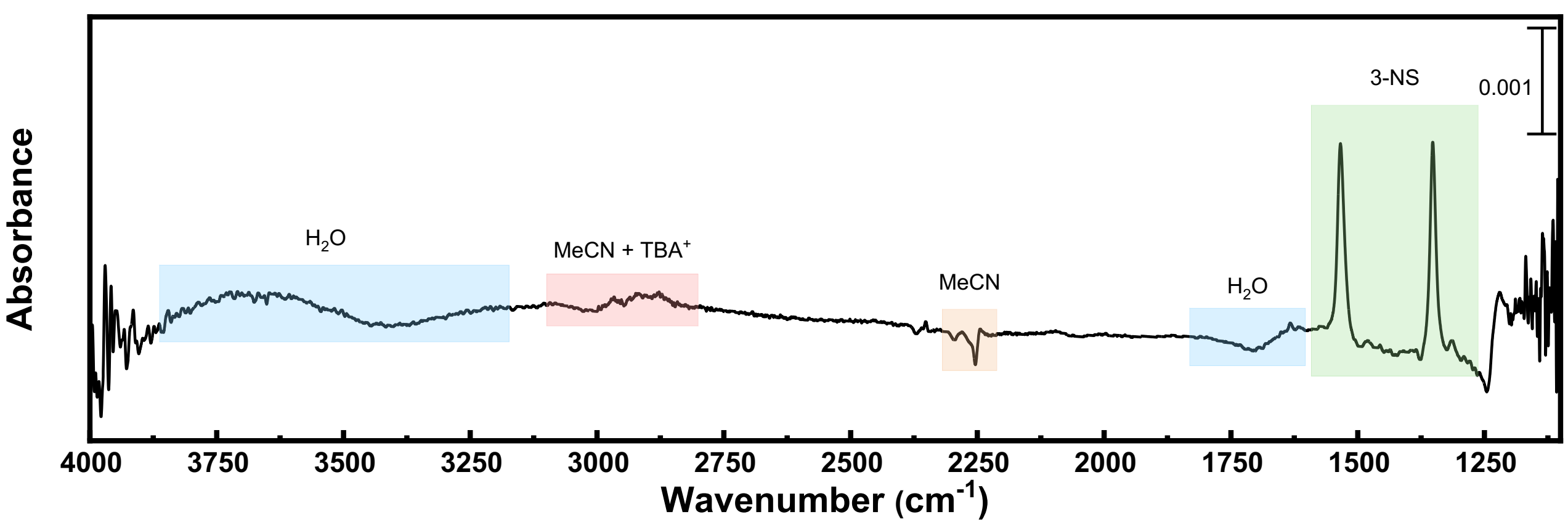

Figure S41. Representative SEIRA spectrum of a solution of 100 mM 3-NS and 100 mM $TBAPF_6$ in MeCN in contact with Pt. Band assignments are further discussed below in SI section S9.5.

9.4.2 EC-SEIRAS experiments of 3-NS on Pt in the absence of H_2 as a function of applied potential, involving strongly surface-bound 3-NS only

To probe solely strongly surface-bound (chemisorbed) 3-NS molecules, we removed solvated and physisorbed or weakly-bound 3-NS species as follows. After performing the EC-SEIRAS measurements described in SI section S9.4.1 in the presence of 3-NS without H_2 (forward and reverse scans), the solution was removed from the cell, and the Pt film was washed twice with dry MeCN. The reference and the counter

electrodes were rinsed with copious amounts of dry MeCN. Then, 7.4 mL of a solution of 100 mM $TBAPF_6$ in dry MeCN was added. No 3-NS was added. This process is termed as "solution exchange" throughout this section. Then, two SEIRA spectra were recorded, one with respect to the background spectra taken for the $TBAPF_6$ solution in MeCN on Pt (before addition of 3-NS and before varying the applied potential in section S9.4.1) and one with respect to the SEIRA spectrum recorded after EC-SEIRAS measurements of a 3-NS solution as a function of applied potential (at the end of section S9.4.1). These two spectra showed solely strongly surface-bound 3-NS_{ads} and the dissolved/weakly-bound 3-NS_{solv} species that have been removed during the solution exchange, respectively (Figure S42a). The band intensities of the $\tilde{\nu}_s(NO_2)$ and $\tilde{\nu}_{as}(NO_2)$ modes (also see SI section S9.5 for assignments) of the remaining strongly surface-bound 3-NS_{ads} is ca. 30% of the corresponding bands obtained by SEIRAS of the 100 mM NS solution. Similar SEIRAS data can be obtained if instead of the doing the forward and reverse EC-SEIRAS measurements using a 3-NS solution as described in SI section S9.4.1, first, a positive potential is applied for several minutes. However, this typically resulted in lower 3-NS_{ads} band intensity (Figure S42b) or MeCN adsorption bands that interfered to a larger extent with EC-SEIRAS analysis of bands associated with 3-NS_{ads}. For this reason, we typically first performed forward and reverse EC-SEIRAS measurements as described above. Then, positive potentials were applied in the potential range between the measured OCV value (typically ca. −0.15 V *vs*. Ag/AgCl, the OCV values were roughly the same for 100 mM 3-NS solution and after the solution exchange) to +1.0 V *vs*. OCV (typically +0.85 V *vs*. Ag/AgCl) in increments of 50 mV (forward scan) and then back towards less positive applied potentials to OCV (reverse scan). Each potential was held for 90 s, during which SEIRA spectra were recorded every 45 s. This procedure was replicated four times in individual replicate experiments, each with a freshly prepared Pt film. The obtained EC-SEIRAS data as a function of applied potential are shown and discussed in SI section S9.8.

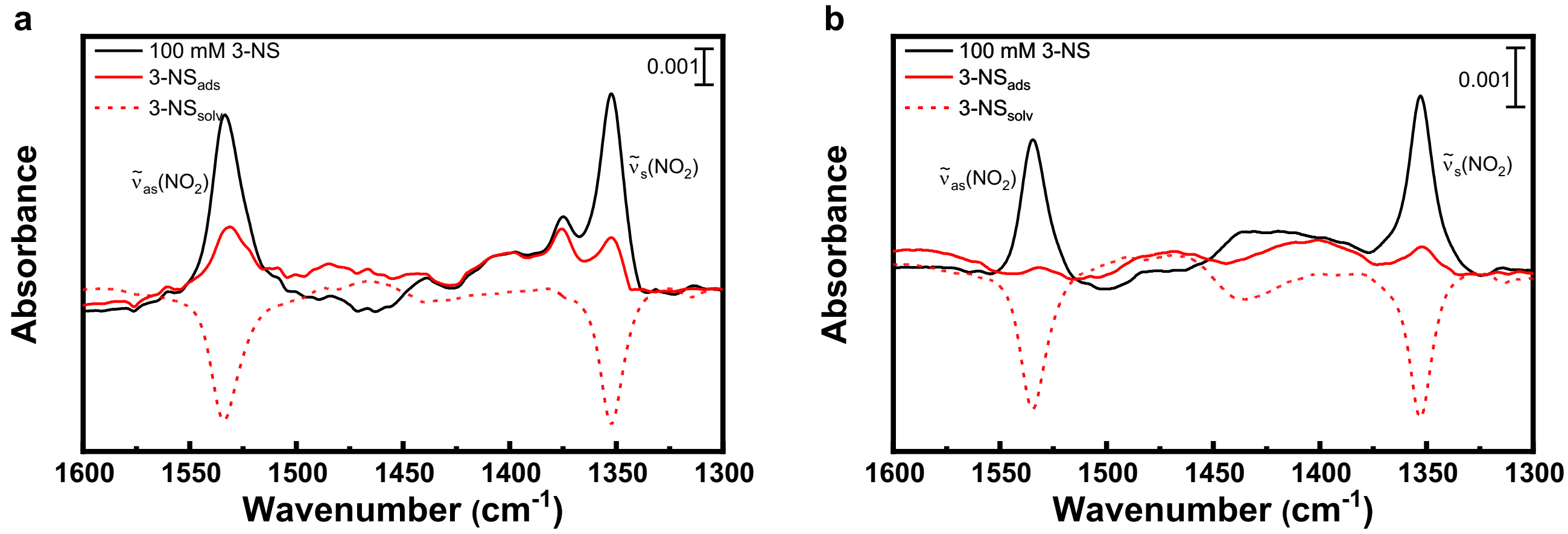

Figure S42. (a) SEIRA spectra recorded at OCV of 100 mM 3-NS on Pt with respect to a background spectrum collected with 100 mM $TBAPF_6$ in MeCN on Pt (black line), strongly-adsorbed 3-NS_{ads} on Pt in contact with 100 mM $TBAPF_6$ in MeCN (obtained after forward and backward scans with 100 mM 3-NS and solution exchange) with respect to a background spectrum collected with 100 mM $TBAPF_6$ in MeCN on Pt (red, solid line), and 3-NS_{solv} (obtained after forward and backward scans with 100 mM 3-NS and solution exchange) with respect to a background spectrum recorded at the end of the backward scan with 100 mM 3-NS and 100 mM $TBAPF_6$ in MeCN on Pt (red, dashed line) in the absence of H_2. (b) SEIRA spectra recorded at OCV of 100 mM 3-NS with respect to a background spectrum collected with 100 mM $TBAPF_6$ in MeCN on Pt (black line), strongly-adsorbed 3-NS_{ads} on Pt in contact with 100 mM $TBAPF_6$ in MeCN (after applying 0.30 V *vs*. Ag/AgCl for 5 min and solution exchange) with respect to a background spectrum collected with 100 mM $TBAPF_6$ in MeCN on Pt (red, solid line), and 3-NS_{solv} (after applying 0.30 V *vs*. Ag/AgCl for 5 min and solution exchange) with respect to a background spectra recorded at the end of the backward scan with 100 mM 3-NS and 100 mM $TBAPF_6$ in MeCN on Pt (red, dashed line) in the absence of H_2.

9.4.3 EC-SEIRAS experiments of 3-NS on Pt in the presence of H_2 as a function of applied potential

We then studied the effect of an applied electrochemical potential on the adsorption of 3-NS in the presence of H_2 by EC-SEIRAS. After the assembly of the spectroelectrochemical cell, the preparation and washing of a Pt film on Au as described above, 7.4 mL of a 100 mM solution of $TBAPF_6$ in dry MeCN was added to the spectroelectrochemical cell and the OCV as well as a background SEIRA spectrum were recorded. Then, the $N_{2(g)}$ inlet was changed to a $H_{2(g)}$ flow (roughly 5.5 sccm), a gas-outlet was set up to ensure a continuous purge and ambient pressure, and the OCV was continuously recorded. After starting the $H_{2(g)}$ purge, the OCV dropped to ca. −0.80 V *vs*. Ag/AgCl (ca. −1.15 V *vs*. Ag/Ag^+) within 20–30 min and then remained stable, similarly to what was observed in our standard H-cell setup used in this manuscript. After the OCV had stabilized, a SEIRA spectrum was recorded (Figure S43, black line). This spectrum shows a broad band centered at ca. 2065–2050 cm^{-1}, which based on prior literature can be attributed to adsorbed (atop) hydrogen on Pt (Pt–H).[37-39] Then, a potential of +0.80 V *vs*. OCV (typically ca. 0.00 V *vs*. Ag/AgCl) was applied for 3 min and a SEIRA spectrum (Figure S43, red line) as well as a new background SEIRA spectrum was recorded directly afterwards for the following SEIRAS experiments. The applied positive potential helped to minimize contributions from the MeCN solvent in later SEIRAS measurements. Applying a positive potential *vs*. OCV resulted in the decrease in the intensity of the Pt–H band, suggesting a lower H* surface concentration at more positive applied potentials (see also SI section S9.9).[37,40] Then, 104.5 μL of 3-NS was added to the solution to obtain a 3-NS solution of 100 mM final concentration in the cell. After stabilization of the OCV value (after ca. 10 min), two SEIRA spectra were recorded, one with

respect to each of the previously measured backgrounds (one shown as green lines in Figure S43 and the other shown in Figure S44). Then, we applied different potentials more positive than the measured OCV (typically ca. −0.70 V *vs*. Ag/AgCl) to +1.00 V *vs*. OCV (typically +0.30 V *vs*. Ag/AgCl) in increments of 50 mV (forward scan) and then again back to less positive applied potentials to OCV (reverse scan). Each potential was held for 90 s during which SEIRA spectra were recorded every 45 s. The current passed during application of a potential was recorded. This procedure was reproduced two times in individual replicate experiments, each with a freshly prepared Pt film. The obtained EC-SEIRAS data as a function of applied potential are shown and discussed in SI section S9.9.

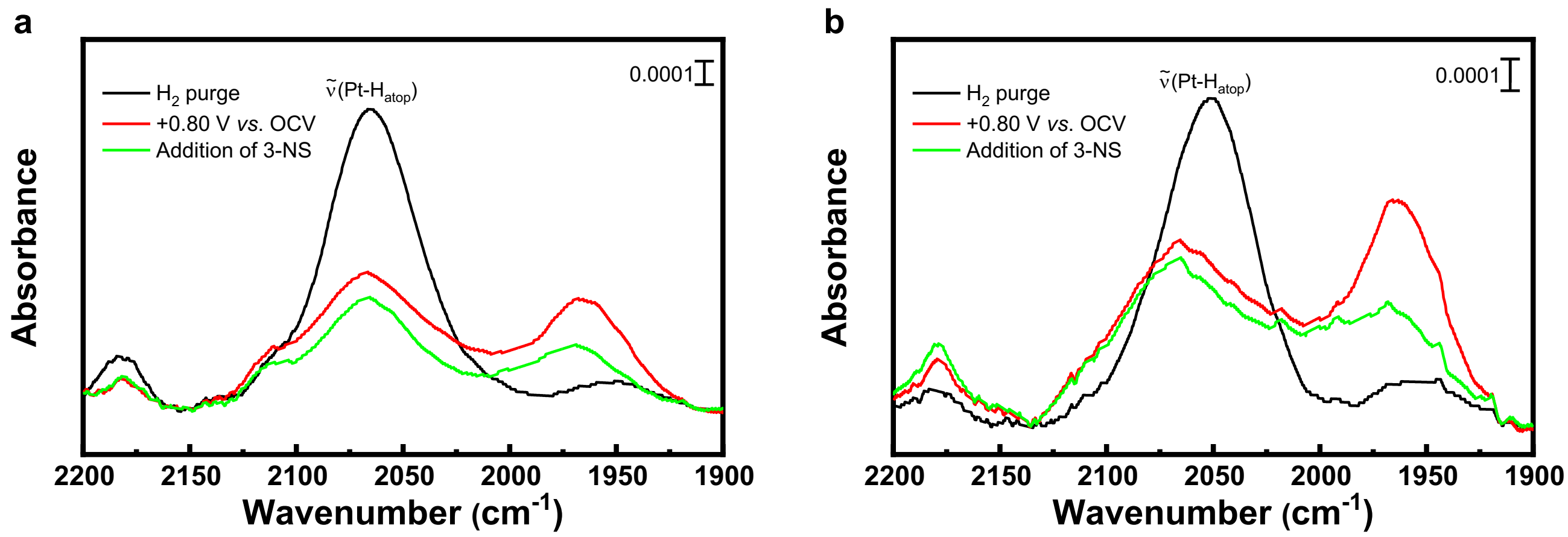

Figure S43. (a) and (b) Two replicate SEIRA spectra recorded at OCV of 100 mM $TBAPF_6$ in MeCN on Pt in the presence of H_2 (black lines), the same solution in the presence of H_2 while applying +0.80 V *vs*. OCV for 3 min (red lines), and after the addition of 104.5 µL of 3-NS in the presence of H_2 (green lines) with respect to background SEIRA spectra collected on Pt with 100 mM $TBAPF_6$ in MeCN in the absence of H_2. Band assignments are further discussed below in SI section S9.5.

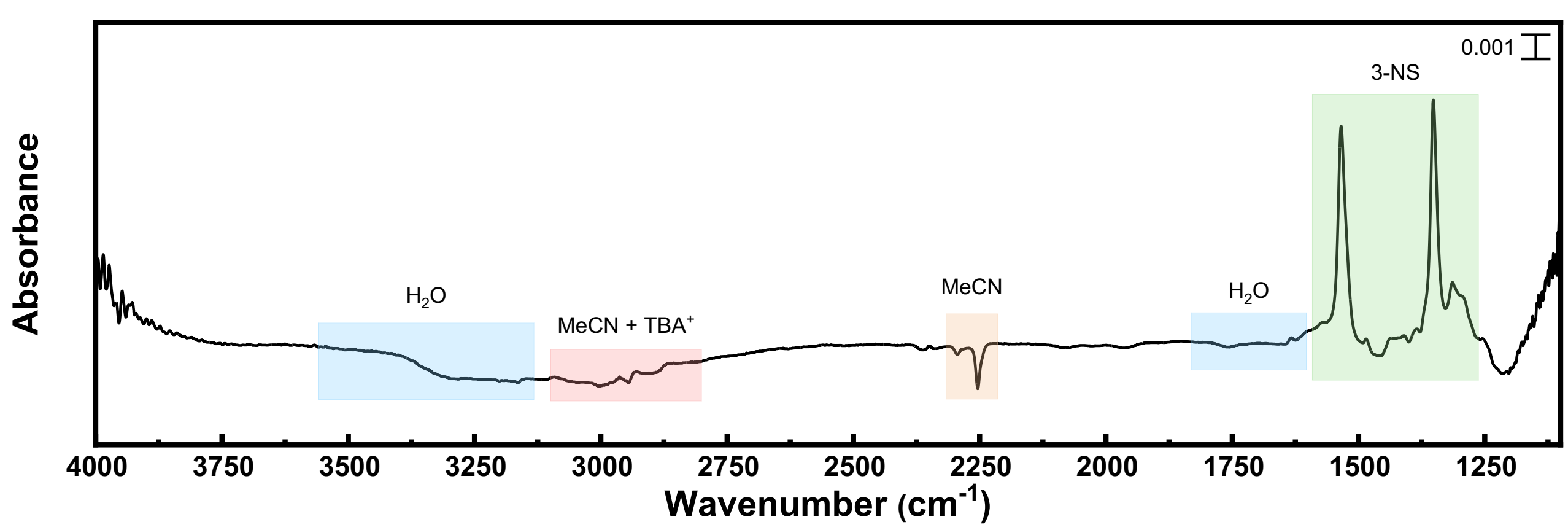

Figure S44. Representative SEIRA spectrum of a solution of 100 mM 3-NS and 100 mM $TBAPF_6$ in MeCN in contact with Pt in presence of $H_{2(g)}$ with respect to a background collected with 100 mM $TBAPF_6$ in MeCN on Pt in the presence of H_2. Band assignments are further discussed below in SI section S9.5.

9.4.4 EC-SEIRAS experiments of 3-NS on Pt in the presence of H_2 as a function of time, involving strongly surface-bound 3-NS only

To investigate surface-adsorbed 3-NS species in the presence of H_2, a solution exchange experiment was performed. For that, a H_2-saturated 100 mM 3-NS and 100 mM $TBAPF_6$ solution in 7.4 mL MeCN was prepared as described in SI section S9.4.3. Then, the H_2-flow was stopped by closing the valve and the spectrochemical cell was closed. A positive potential of +0.80 V *vs*. OCV (0.00 V *vs*. Ag/AgCl) was applied for 15 min. A background SEIRA spectrum was recorded.. Next, the solution was removed from the cell, and the cell was washed twice with dry MeCN and 7.4 mL of a fresh solution of 100 mM $TBAPF_6$ in dry MeCN was added. No 3-NS was added. Then, two SEIRA spectra were recorded, one with respect to the background spectra taken with the $TBAPF_6$ solution in MeCN on Pt (before addition of 3-NS), and one with respect to the new background scan recorded after applying 0.00 V *vs*. Ag/AgCl for 15 min. These two spectra showed solely surface-bound 3-NS_{ads} and the solvated/weakly-bound 3-NS_{solv} species that have been removed during the solution exchange (Figure S45). The amount of observed NS_{ads} is lower compared to that in the absence of H_2 (Figure S42a), which can result from the reduction of surface-bound species through adsorbed H atoms and/or from a hindered adsorption of 3-NS species on the H_2-saturated surface. After the solution exchange, a slow H_2 flow was set (ca. 1 bubble/2 s) and series of SEIRA spectra were recorded every minute until the OCV reached a stable value of ca. −0.8 V *vs*. Ag/AgCl. The obtained EC-SEIRAS data as a function of time are shown and discussed in SI section S9.10.

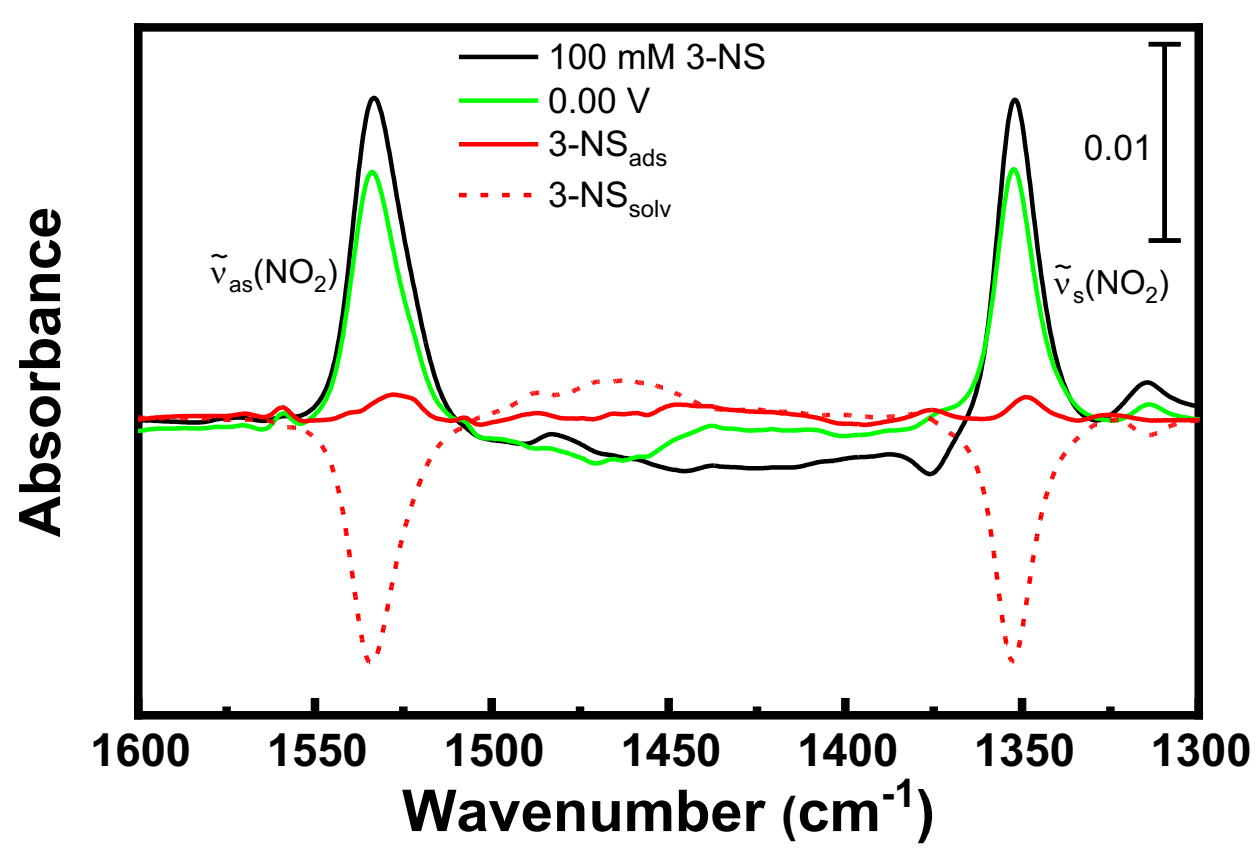

Figure S45. SEIRA spectra recorded at OCV of 100 mM 3-NS and 100 mM $TBAPF_6$ in MeCN on Pt in the presence of H_2 with respect to a background collected with 100 mM $TBAPF_6$ in MeCN on Pt in the presence of H_2 (black line), the same solution of 3-NS in the presence of H_2 while applying 0.00 V *vs.* Ag/AgCl for 15 min (green line), 3-NS_{ads} after solution exchange in the absence of H_2 (before re-starting the H_2 sparge) with respect to a background collected with 100 mM $TBAPF_6$ in MeCN in the presence of H_2 (red, solid line), and 3-NS_{solv} after solution exchange in the absence of H_2 with respect to the SEIRA spectrum recorded after applying a positive potential (red, dashed line).

9.5 Band assignments

To identify observed SEIRAS bands associated with 3-NS, we recorded SEIRA spectra of a more concentrated solution of 3-NS (1 M instead of the standard 100 mM used above) in a solution of 100 mM $TBAPF_6$ in MeCN in the absence of H_2 on different surfaces: the bare Si ATR crystal, the Au film, and the Pt film on Au (Figure S46a). While the latter two provide surface-enhanced IR spectra with significant contribution of surface-bound species, the IR measurements on the bare Si crystal should predominantly correspond to 3-NS in solution. All the three recorded spectra show two main absorption bands between 1600 and 1300 cm^{-1}, at ~1530 and ~1350 cm^{-1}. Based on prior work[41-43] and by comparison to SEIRA spectra obtained on Au of a 1 M solution of nitrobenzene or a 1 M solution of styrene in 100 mM $TBAPF_6$ in MeCN (Figure S46b), we attribute the two bands to the antisymmetric ($\tilde{\nu}_{as}(NO_2)$, ~1530 cm^{-1}) and symmetric ($\tilde{\nu}_s(NO_2)$, ~1350 cm^{-1}) stretching vibrations of the NO_2 group in 3-NS. The C=C stretching frequency ($\tilde{\nu}$(C=C)) of the vinyl group in 3-NS was previously reported at ca. 1630 cm^{-1}.[41,42] This band could not be clearly observed on either Au, Pt or the Si crystal, probably due to low signal intensity and/or overlap with the broad absorption band at ca. 1600 cm^{-1} associated with the H–O–H bending mode of H_2O.

Multiple bands between ~1530 and ~1350 cm^{-1} (between $\tilde{\nu}_{as}(NO_2)$ and $\tilde{\nu}_s(NO_2)$) were observed when measuring solutions of 3-NS in MeCN on Au or Pt, especially at positive applied potentials (also see below). We attribute these to overlapping aromatic ring vibrations of 3-NS[41,42] as well as to several vibrational modes of MeCN[44,45]. A relatively intense and sharp band at 1375 cm^{-1} can be more clearly assigned and is associated with the CH_3 symmetric deformation mode $\delta_s(CH_3)$ of MeCN.[44,45] Additionally, MeCN shows CH_3 antisymmetric and symmetric stretching vibrations at 3000 and 2940 cm^{-1}, respectively (Figure S47)[44,45] as well as a CN stretching vibration ($\tilde{\nu}$(CN)) at ~2250 cm^{-1} (Figure S46).

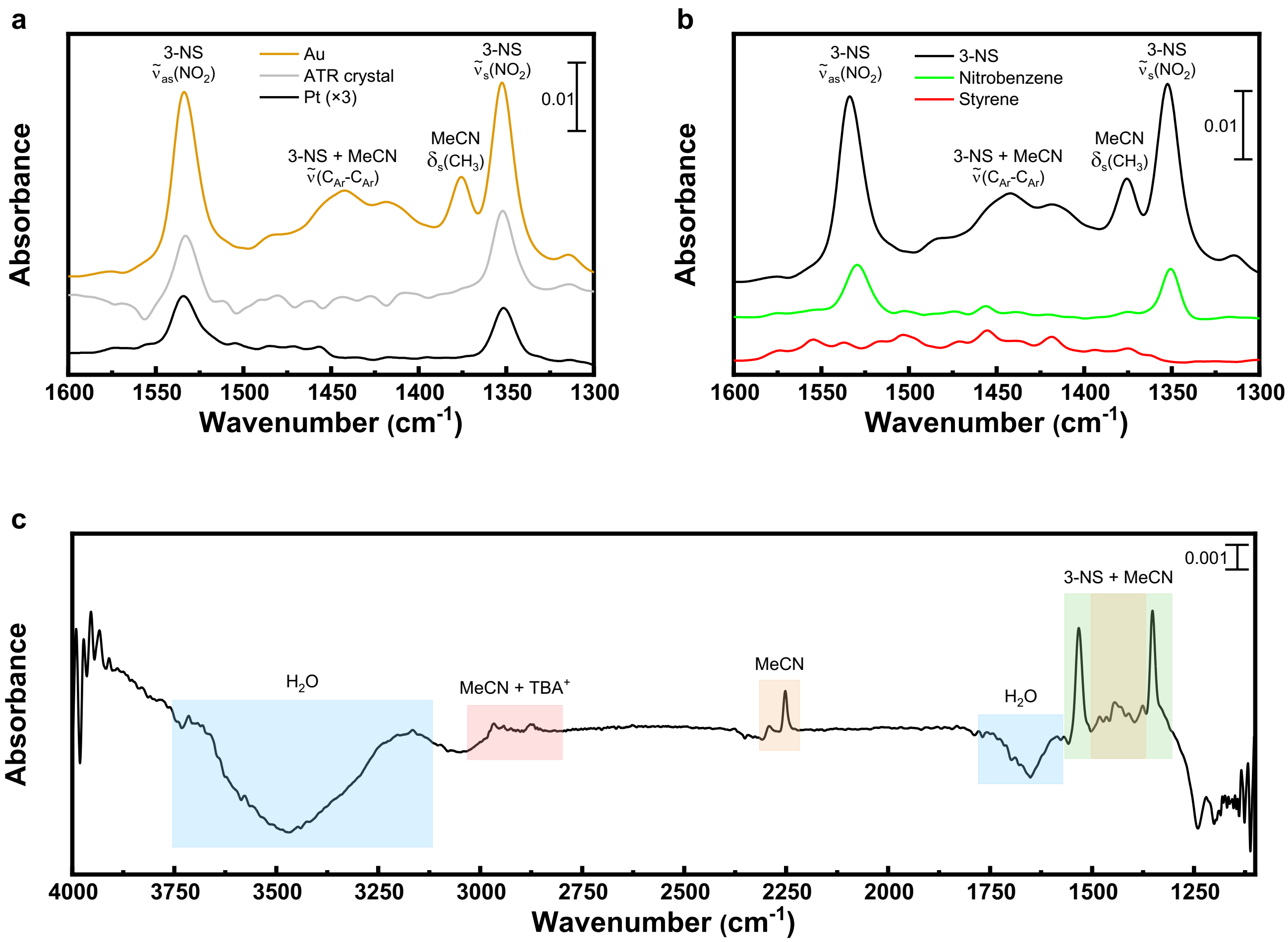

Figure S46. (a) SEIRA spectra recorded of 1 M 3-NS and 100 mM $TBAPF_6$ in MeCN in the absence of H_2 on the Si crystal (grey line), the Au film (golden line), and the Pt overlayer film (spectrum vertically enlarged by a factor of 3, black line). (b) SEIRA spectra recorded of 1 M 3-NS (black line), 1 M nitrobenzene (green line), and 1 M styrene (red line) in MeCN, $TBAPF_6$ on a Au film in the absence of H_2. (c) SEIRA spectrum recorded of 1 M 3-NS and 100 mM $TBAPF_6$ in MeCN in the absence of H_2 on Pt overlayer film after 1 h showing the full range between 4000 and 1100 cm^{-1} with respect to a background collected with 100 mM $TBAPF_6$ in MeCN on Pt in the absence of H_2.

Recording a SEIRA spectra with 100 mM $TBAPF_6$ in MeCN solution with respect to a MeCN background showed three new bands (2970, 2940, and 2880 cm^{-1})[46,47], which can be attributed to the C–H stretching vibrations of TBA^+, see Figure S47. The vibration frequencies of the observed bands for 3-NS, MeCN and $TBAPF_6$ are summarized in Table S15.

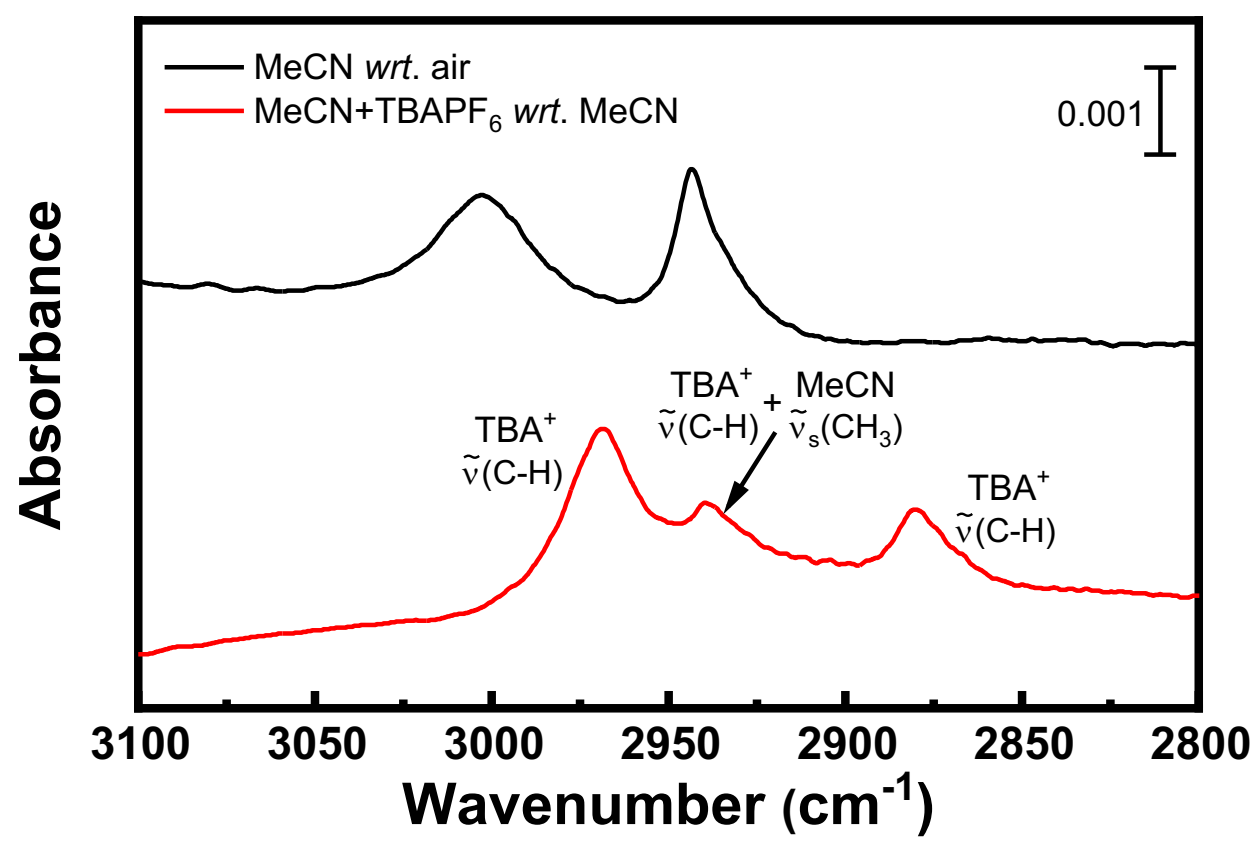

Figure S47. SEIRA spectra recorded of pure MeCN on Pt with respect to a background spectrum measured of the Pt layer in air (black line), and 100 mM $TBAPF_6$ in MeCN on Pt with respect to a background spectrum measured of MeCN on Pt (red line).

Recording a SEIRA spectrum of Pt in contact with a H_2-saturated 100 mM $TBAPF_6$ in MeCN solution with respect to a background collected with 100 mM $TBAPF_6$ in MeCN on Pt in the absence of H_2 showed a new band centered at ca. 2050–2065 cm^{-1}, which can be attributed to the Pt–H vibration of adsorbed hydrogen at atop sites of Pt ($\tilde{\nu}$(Pt–H_{atop})).[37-39,48]

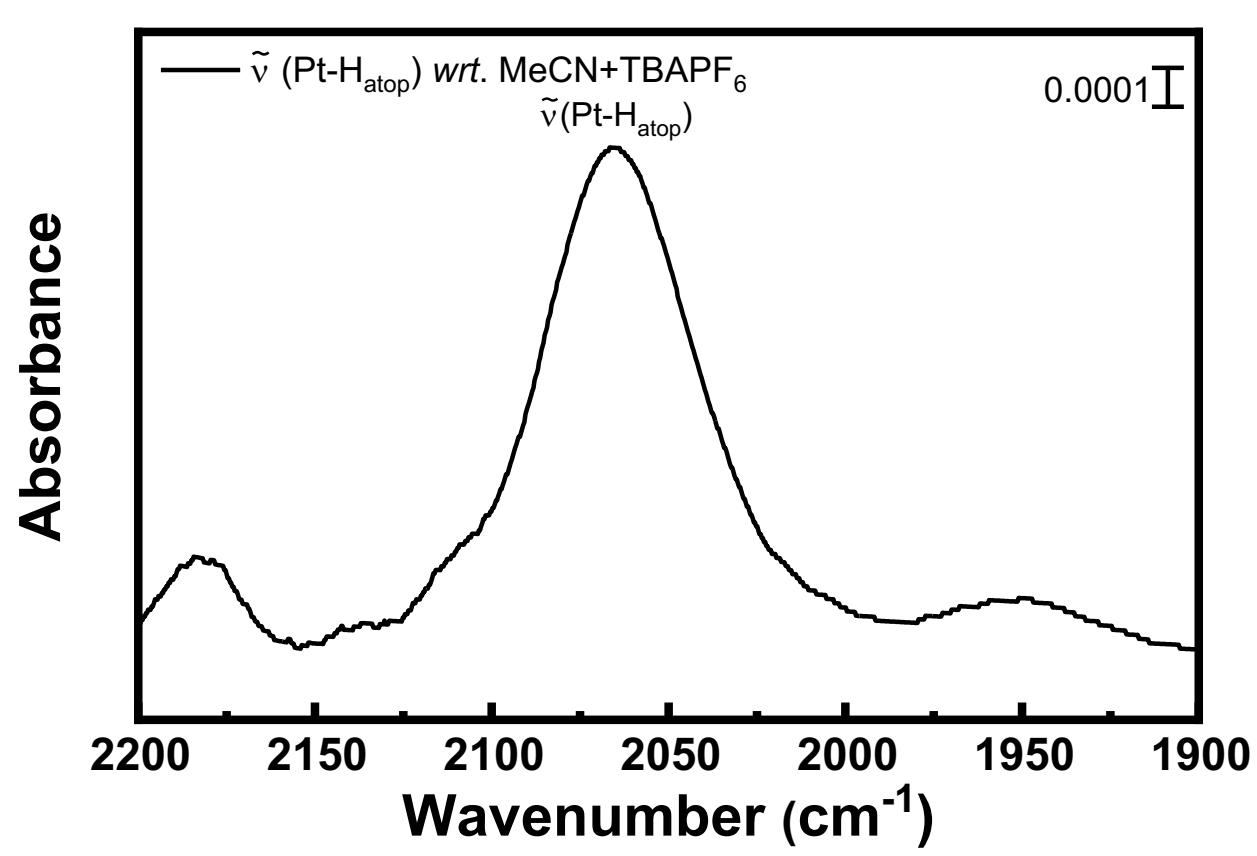

Figure S48. SEIRA spectrum recorded at OCV of Pt in contact with 100 mM $TBAPF_6$ in MeCN in the presence of H_2 with respect to a background collected with 100 mM $TBAPF_6$ in MeCN on Pt in the absence of H_2.

Table S15. Vibrational bands associated with 3-NS, MeCN, TBA^+, and Pt–H.

Species	IR frequency (cm^{-1})	Band assignment	Reference (cm^{-1})
3-NS	*not observed*	$\tilde{\nu}$ (C=C)	1630[42], 1636[41]
	1534	$\tilde{\nu}_{as}$ (NO_2)	1530[41]
	1495–1390	$\tilde{\nu}$ (C_{Ar}–C_{Ar})	1494 and 1450[42], 1448[49]
	1352	$\tilde{\nu}_{s}$ (NO_2)	1350[41]
MeCN	3003	$\tilde{\nu}_{as}$ (CH_3)	3001.7[45], 3000[44]
	2943	$\tilde{\nu}_{s}$ (CH_3)	2940.9[45], 2945[44]
	2315–2273	Combination modes	2289[45], 2630–2290[44], 2292[47]
	2252	$\tilde{\nu}$ (CN)	2252.2[45], 2256[44], 2253[47]
	1495–1390	δ_{as} (CH_3), combination modes	1410.0 and 1448.3[45], 1420 and 1444[44]
	1376	δ_{s} (CH_3)	1374.5[45], 1378[44]
TBA^+	2968<br>2939<br>2880	$\tilde{\nu}$ (C–H) of TBA^+	3000–2900[46], 2968–2897[47]
Pt–H	2065–2050	$\tilde{\nu}$ (Pt–H_{atop})	2052[38], 2050–2100[39], 2090[37]

9.6 Implications from observed SEIRAS bands associated with 3-NS from surface selection rules

According to the surface selection rule of SEIRAS, only the component that is perpendicular to the surface of a dipole moment change associated with a molecular vibration can be observed.[50,51] The components parallel to the surface are SEIRAS inactive. Based on prior theoretical and experimental work,[42,52,53] we initially consider two vertical binding modes of 3-NS on Pt, κ^2- or κ^1-bound 3-NS interacting via the NO_2-group with the surface, or an idealized parallel adsorption mode, in which the vinyl-group, the aromatic ring as well as the NO_2-group interact with the surface (Figure S49). According to surface selection rules, for the κ^2-bound vertical 3-NS the $\tilde{\nu}_s(NO_2)$ mode should be observed by SEIRAS but not the $\tilde{\nu}_{as}(NO_2)$ mode.[51] The $\tilde{\nu}$(C=C) mode may also be observed in this configuration.[50,52] For the idealized parallel adsorption mode, neither $\tilde{\nu}_s(NO_2)$, nor $\tilde{\nu}_{as}(NO_2)$, nor $\tilde{\nu}$(C=C) should be observed (Figure S49). Figure S49a shows that both the $\tilde{\nu}_s(NO_2)$ and the $\tilde{\nu}_{as}(NO_2)$ modes are observed by SEIRAS on Pt. This is in particular also the case when we are probing strongly surface-bound 3-NS_{ads} (Figure S54), which excludes any possible contribution from 3-NS in solution close to the surface and shows that both vibrational modes are observed for surface-bound 3-NS_{ads}. This suggests that while both the vertical κ^2-bound 3-NS as well as the idealized parallel mode of 3-NS can still occur, other adsorption modes must contribute to ensemble of adsorbed 3-NS molecules measured by SEIRAS under our conditions. The vertical κ^1-bound 3-NS should lead to observable $\tilde{\nu}_s(NO_2)$ and $\tilde{\nu}_{as}(NO_2)$ modes (as well as $\tilde{\nu}$(C=C)). However, binding of nitrostyrene to Pt through the NO_2-group has previously been shown to be weak in general, and with the κ^1-bound adsorption mode having the lowest adsorption energy compared to the κ^2-bound or parallel-bound nitrostyrene on Pt.[54,55] The vertical κ^1-bound 3-NS hence seems unlikely to explain our SEIRAS data. Parallel adsorption modes have previously been reported to have the more favorable adsorption energies by up to a factor of four versus vertical adsorption modes of nitrostyrene on Pt.[52,54] Prior theoretical work suggests that nitrostyrene at higher (more realistic) coverages prefers a tilted mode from the idealized parallel adsorption, in which there is a stronger interaction between the surface and the vinyl-group and the nitro–phenyl-group tilts up.[54] This is consistent with extensive prior literature on the binding of olefins to the surface that can go towards a di–σ binding mode.[56-58] In such a tilted parallel adsorption mode, both the $\tilde{\nu}_s(NO_2)$ and $\tilde{\nu}_{as}(NO_2)$ modes have a perpendicular component with respect to the surface and both modes could be observed. *Therefore, we attribute the observation of both $\tilde{\nu}_s(NO_2)$ and $\tilde{\nu}_{as}(NO_2)$ modes for 3-NS on Pt by SEIRAS to an ensemble of 3-NS adsorption modes that includes a vinyl-bound tilted adsorption mode of 3-NS (Figure S49) and possibly other adsorption modes*.

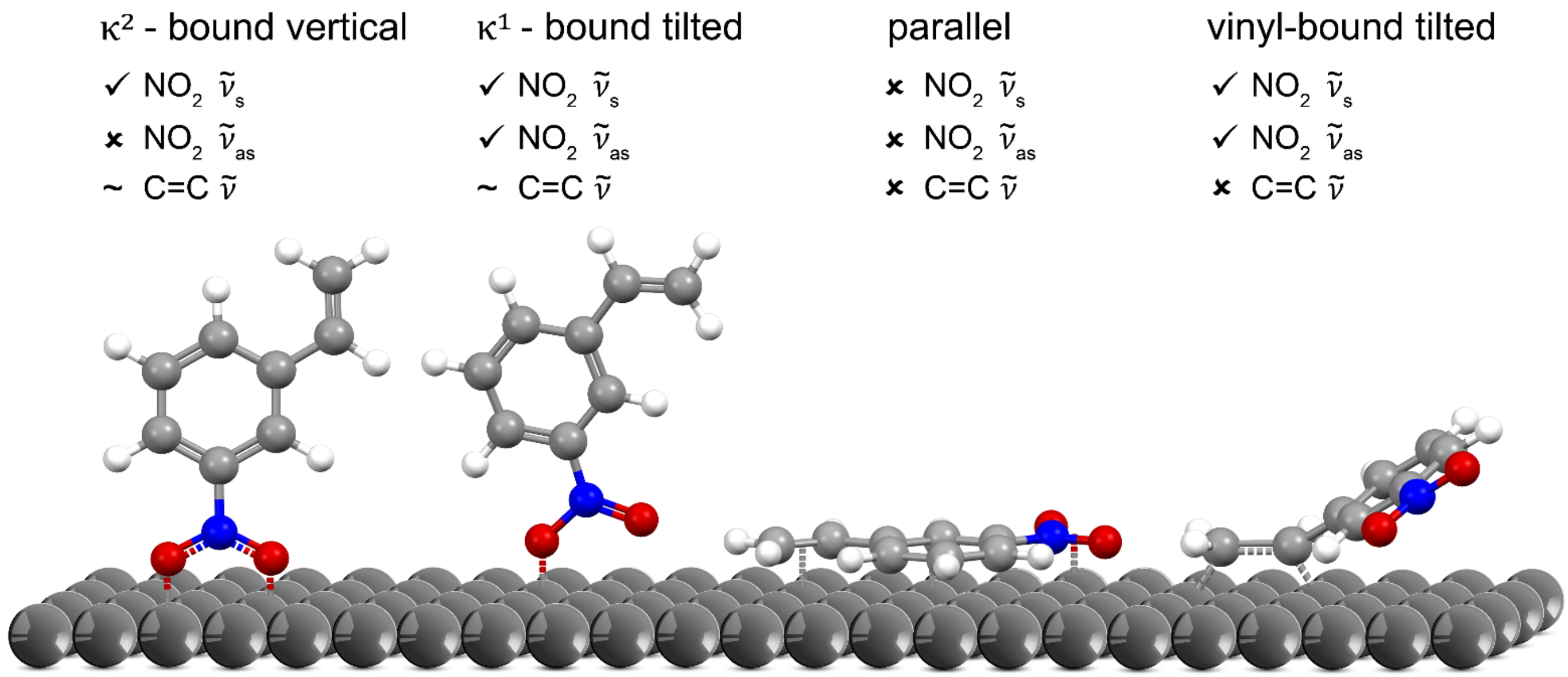

Figure S49. Graphic representation of possible adsorption modes of surface-bound 3-NS_{ads} to visualize different surface geometries and above associated bands that should be observable or not based surface selection rules. From left to the right: κ^2-bound vertical, κ^1-bound, ideal parallel, and vinyl-bound tilted parallel adsorption modes.

9.7 EC-SEIRAS data of 3-NS on Pt in the absence of H_2 as a function of applied potential

9.7.1 Forward scan from OCV to more positive applied potentials

Figure S50 shows three replicate experiments of the EC-SEIRAS measurements done on Pt in the presence of 3-NS in a solution of $TBAPF_6$ in MeCN and in the absence of H_2 as a function of applied potential during a forward scan from OCV to more positive applied potentials (also see SI section S9.4.1 for experimental details). While the $\tilde{\nu}$(C=C) of 3-NS could not be observed, both the $\tilde{\nu}_s(NO_2)$ (~1350 cm^{-1}) and $\tilde{\nu}_{as}(NO_2)$ (~1530 cm^{-1}) bands were observed and increased in intensity with more positive applied potentials. Similarly, the intensity of the CH_3 symmetric deformation mode $\delta_s(CH_3)$ of MeCN (~1375 cm^{-1}) also showed an increase in intensity with more positive applied potentials. In line with the expected positive polarization of the surface at more positive applied potentials, we also observed a decrease of the bands at 2970, 2940, and 2880 cm^{-1} associated with TBA^+ (Figure S51).[46,47]

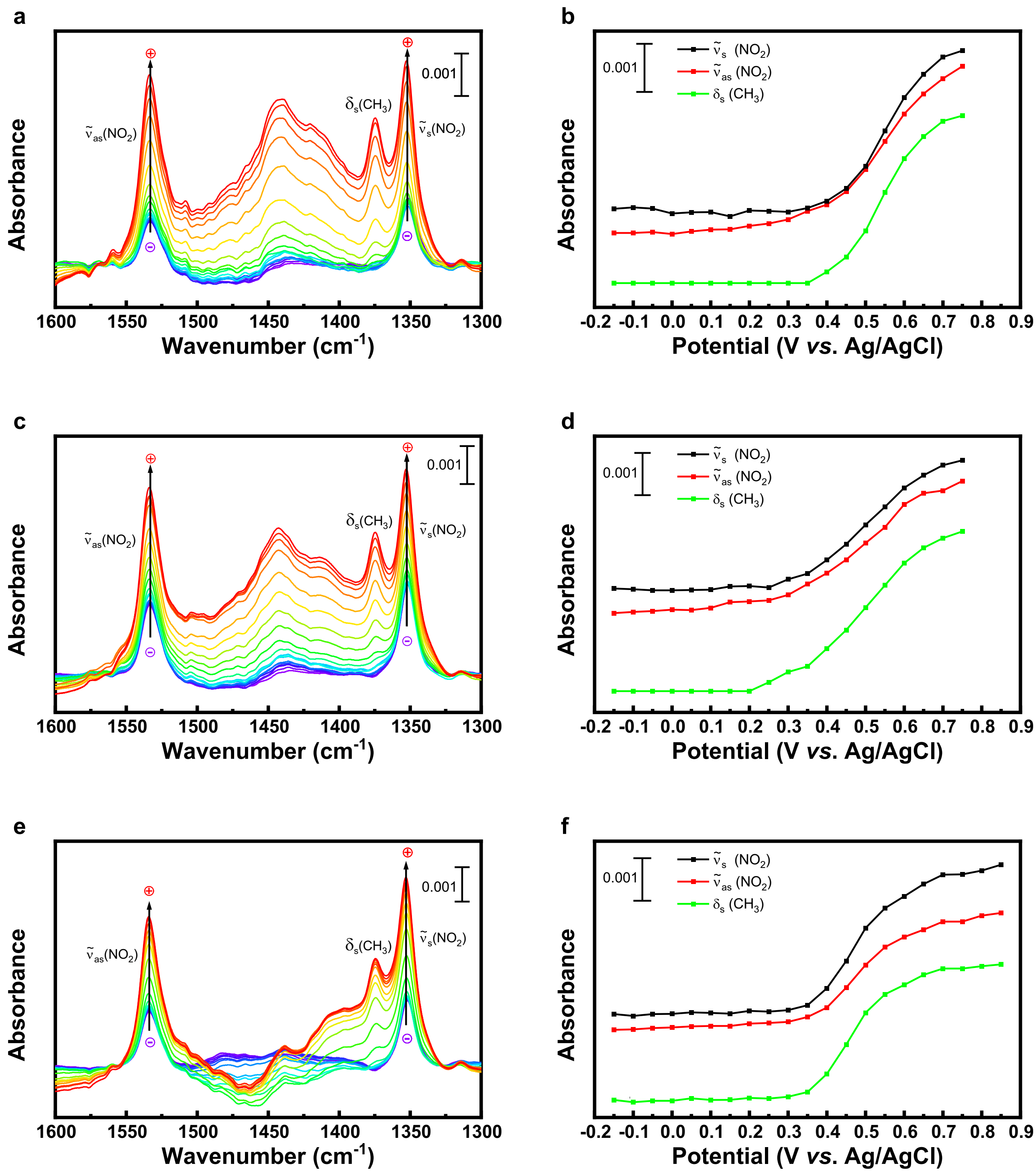

Figure S50. (a, c, e) Three replicate experiments of SEIRA spectra of 100 mM 3-NS and 100 mM $TBAPF_6$ in MeCN on Pt in the absence of H_2 while applying increasingly positive potentials from 0.0 to +1.0 V *vs*. OCV (purple to red) with respect to a background SEIRA spectrum measured of 100 mM $TBAPF_6$ in MeCN on Pt directly after applying +0.80 V *vs*. OCV. SEIRA spectra have been background corrected with a multipoint background using anchor points at 1795, 1560, 1320, and 1275 cm^{-1}. One SEIRA spectrum at OCV and one at +1.0 V *vs*. OCV have been reproduced from the main text Figure 3b. (b, d, f) Absorbance values of $\tilde{\nu}_s(NO_2)$ and $\tilde{\nu}_{as}(NO_2)$ of 3-NS, and $\delta_s(CH_3)$ of MeCN as a function of applied potential.

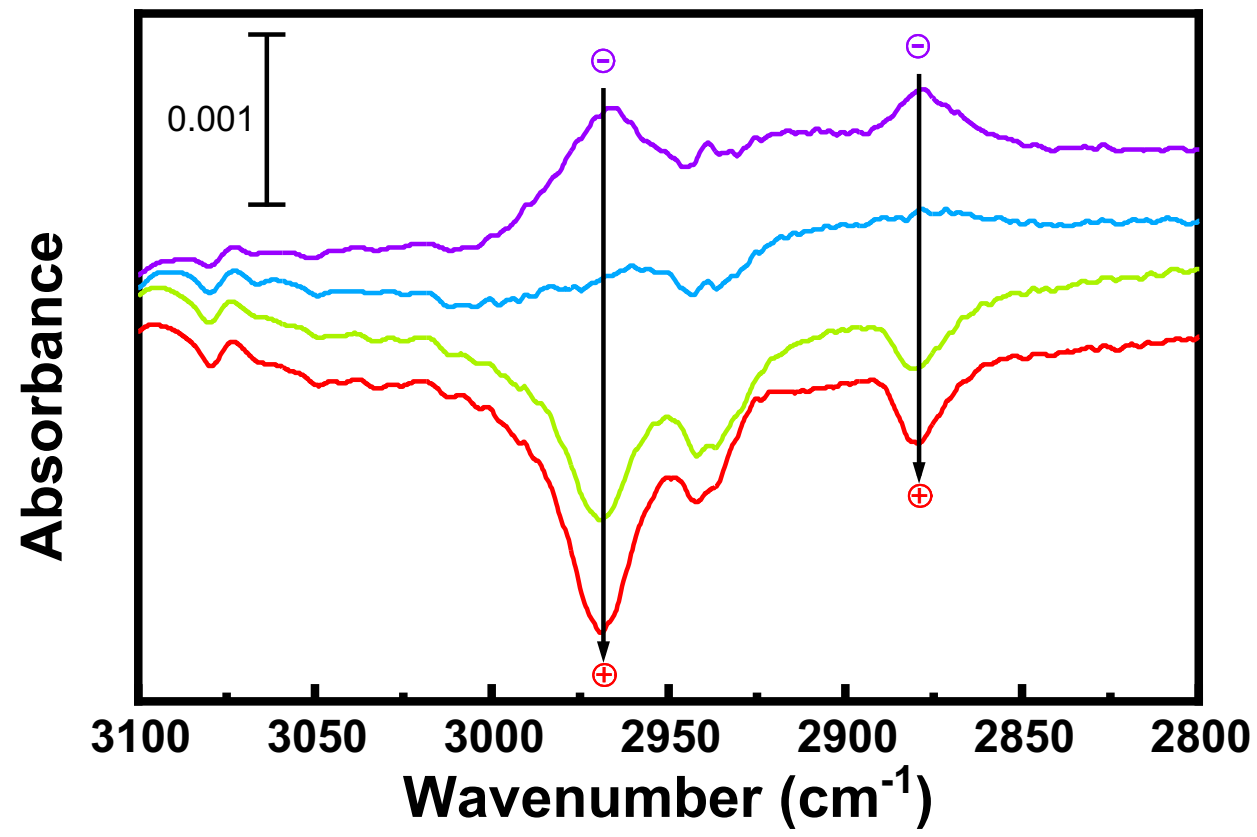

Figure S51. SEIRA spectra of 100 mM 3-NS and 100 mM $TBAPF_6$ in MeCN solution on Pt in the absence of H_2 while applying increasingly positive potentials (0.00, +0.35, +0.70, and +1.00 V *vs*. OCV, purple to red) during a forward scan with respect to a background SEIRA spectrum measured of 100 mM $TBAPF_6$ in MeCN on Pt directly after applying +0.80 V *vs*. OCV.

9.7.2 Reverse scan from more positive applied potentials to OCV

Figure S52 shows three replicate experiments of the EC-SEIRAS measurements done on Pt in the presence of 3-NS in a solution of $TBAPF_6$ in MeCN and in the absence of H_2 as a function of applied potential during the reverse scans from positive applied potentials back to OCV. When going to less positive applied potentials, the intensity of the $\tilde{\nu}_s(NO_2)$ (~1350 cm^{-1}) and $\tilde{\nu}_{as}(NO_2)$ (~1530 cm^{-1}) bands of 3-NS stayed relatively constant compared to the large changes observed during the forward scan. The intensity of the $\tilde{\nu}_s(NO_2)$ band tended to increase further, and the intensity of the $\tilde{\nu}_{as}(NO_2)$ band was more constant with varied applied potential. However, these changes in intensity of the $\tilde{\nu}_{as}(NO_2)$ and the $\tilde{\nu}_s(NO_2)$ are relatively small and especially the $\tilde{\nu}_s(NO_2)$ intensity changes can also depend on the applied baseline correction. These small changes in intensity can hence not unambiguously be interpreted. However, the data clearly show that the observations made above for bands associated with the NO_2-group of 3-NS and with MeCN when going to more positive applied potentials were not reversible in the reverse scan. However, in line with the expected less positive polarization of the surface at less positive applied potentials, we also observed an increase of the bands at 2970, 2940, and 2880 cm^{-1} associated with TBA^+ (Figure S53).[46,47]

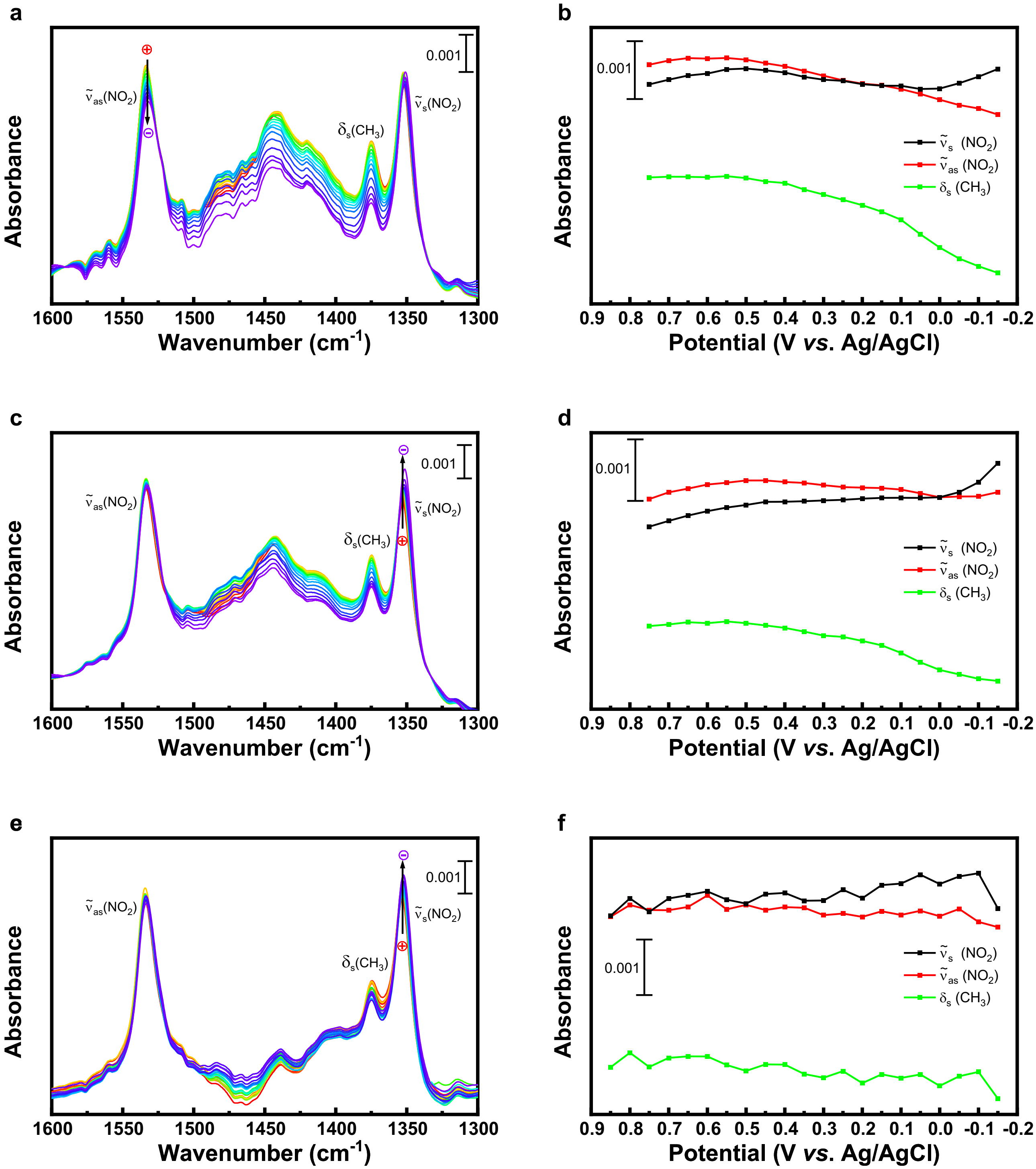

Figure S52. (a, c, e) Three replicate experiments of SEIRA spectra of 100 mM 3-NS and 100 mM $TBAPF_6$ in MeCN on Pt in the absence of H_2 while going from more positive to less positive potentials from +1.0 to 0.0 V *vs*. OCV (red to purple) with respect to a background SEIRA spectrum measured of 100 mM $TBAPF_6$ in MeCN on Pt directly after applying +0.80 V *vs*. OCV. SEIRA spectra have been background corrected with a multipoint background using anchor points at 1795, 1560, and 1335 cm^{-1}. One SEIRA spectrum at OCV and one at +1.0 V *vs*. OCV have been reproduced from the main text Figure 3b. (b, d, f) Absorbance values of $\tilde{\nu}_s(NO_2)$ and $\tilde{\nu}_{as}(NO_2)$ of 3-NS, and $\delta_s(CH_3)$ of MeCN as a function of applied potential.

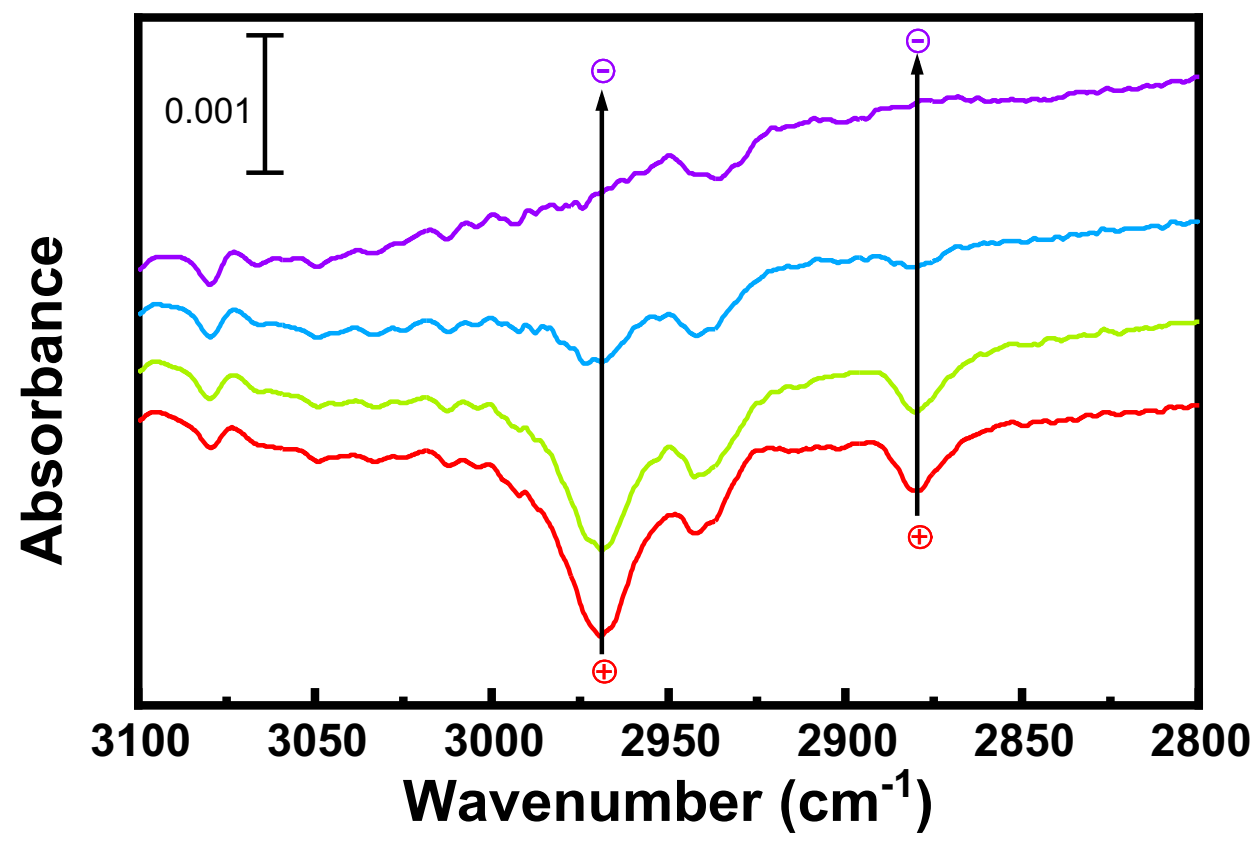

Figure S53. SEIRA spectra of 100 mM 3-NS and 100 mM $TBAPF_6$ in MeCN solution on Pt in the absence of H_2 while going from more positive to less positive applied potentials (+1.00, +0.70, +0.35, and 0.00 V *vs*. OCV, red to purple) during a reverse scan with respect to a background SEIRA spectrum measured of 100 mM $TBAPF_6$ in MeCN on Pt directly after applying +0.80 V *vs*. OCV.

9.7.3 Discussion

Above we show that the intensities of the $\tilde{\nu}_s(NO_2)$ and $\tilde{\nu}_{as}(NO_2)$ bands increased in a similar fashion as the $\delta_s(CH_3)$ band of MeCN with more positive applied potentials in the forward scan and that these bands did not decrease in a reversible manner when going back to less positive applied potentials in a reverse scan. These observations raise the question what the changes in intensity can be attributed to. SEIRAS band intensities are a function of the concentration of adsorbed species near the surface, but also dependent on their orientation or possible changes in SEIRAS enhancement e.g. due to surface reconstruction of the Pt catalyst.[51] It seems unlikely that an orientation change of 3-NS is *solely* responsible to the large increase in both the $\tilde{\nu}_s(NO_2)$ and $\tilde{\nu}_{as}(NO_2)$ band intensities in the forward scan, though, such orientation changes could contribute to our observations (also see below). Characterization of the Pt electrode of our standard H-cell experiments before and after catalysis and measurement of the performance of the Pt catalyst in 3-NS hydrogenation reaction in consecutive experiments at OCV, then at −0.2 V *vs*. Ag/Ag^+, and then again at OCV (SI section S7.1) suggested that the Pt catalyst does not change significantly. Substantial changes in roughness or surface area would have been expected to lead to differences in catalytic activity during the first and second experiments at OCV in the consecutive experiment, but this was not observed. Furthermore, our EC-SEIRAS data discussed below obtained for strongly surface-bound 3-NS only (in the absence of 3-NS in solution that would be available for increased surface adsorption) showed essentially no increase of band intensity of the $\tilde{\nu}_s(NO_2)$ and $\tilde{\nu}_{as}(NO_2)$ bands at more positive applied potentials (see next SI section S9.8). If an increase of SEIRAS enhancement e.g. due to surface roughening occurred at more positive applied potentials, increased band intensities should still have been observed under these conditions. However, this was not the case. Based on all this combined, *we attribute the increase in band intensity of the* $\tilde{\nu}_s(NO_2)$ *and* $\tilde{\nu}_{as}(NO_2)$ *modes observed at more positive applied potentials predominantly to an*

increased concentration of 3-NS at or near the Pt surface. Change in the orientation of 3-NS could also contribute to these observations as further discussed below.

9.8 EC-SEIRAS data of 3-NS on Pt in the absence of H_2 as a function of applied potential, involving strongly surface-bound 3-NS only

After conducting EC-SEIRAS experiments in the presence of the 3-NS solution, the exchange of the 3-NS solution with a $TBAPF_6$/MeCN solution allowed EC-SEIRAS measurements that probe only strongly surface-bound 3-NS_{ads} (also see SI section S9.4.2). This enabled the observation of vibrational shifts and intensity changes due to orientation changes more clearly, as the EC-SEIRAS data obtained above in the presence of a 3-NS solution is dominated by the adsorption of more 3-NS molecules from the solution to the surface (SI section S9.7 and Figures. S50 and S52).

9.8.1 Forward scan from OCV to more positive applied potentials

EC-SEIRA spectra recorded for strongly surface-bound 3-NS on Pt immersed in a $TBAPF_6$/MeCN solution as a function of applied potential going from OCV to more positive applied potentials are shown in Figure S54. In these data, the band intensity of the $\tilde{\nu}_s(NO_2)$ mode increased when going to more positive applied potentials, while the band intensity of the $\tilde{\nu}_{as}(NO_2)$ decreased to a similar extent. These changes in intensity are only roughly 5% of the change in intensity observed for the same bands when going to more positive applied potentials in the presence of a 3-NS solution (cf. Figures. S50 and S54). Both the $\tilde{\nu}_s(NO_2)$ and $\tilde{\nu}_{as}(NO_2)$ of the 3-NS_{ads} in Figure S54 showed a blue-shift of 1.7 ± 0.8 cm^{-1} V^{-1} and 3.0 ± 1.2 cm^{-1} V^{-1}, respectively, when going to more positive applied potentials. The band intensity of the $\delta_s(CH_3)$ mode of MeCN at 1375 cm^{-1} showed a large increase in intensity when going to more positive applied potentials, similarly to what has been observed in the EC-SEIRAS experiments in 3-NS solution (SI section S9.7.1 and Figure S50). Similar trends were observed in four replicate experiments (Figure S54). In only one out of four replicate experiments we observed a slight deviation from these trends, where the band intensity of the $\tilde{\nu}_s(NO_2)$ mode decreased again but only at very positive applied potentials (Figure S54g). Overall the trends observed in intensity changes and vibrational frequency shifts were very reproducible.

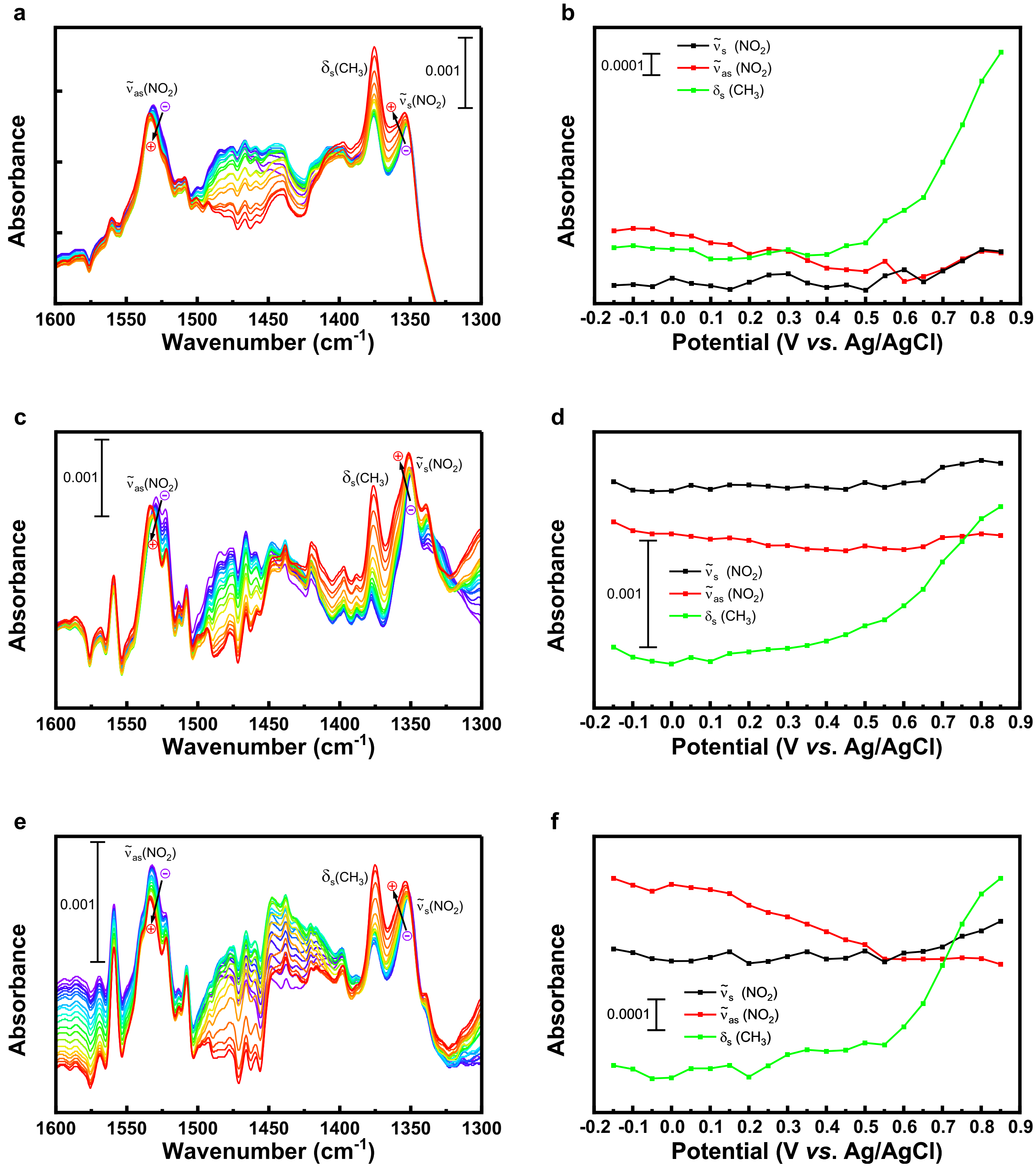

(Figure S54 caption on the next page)

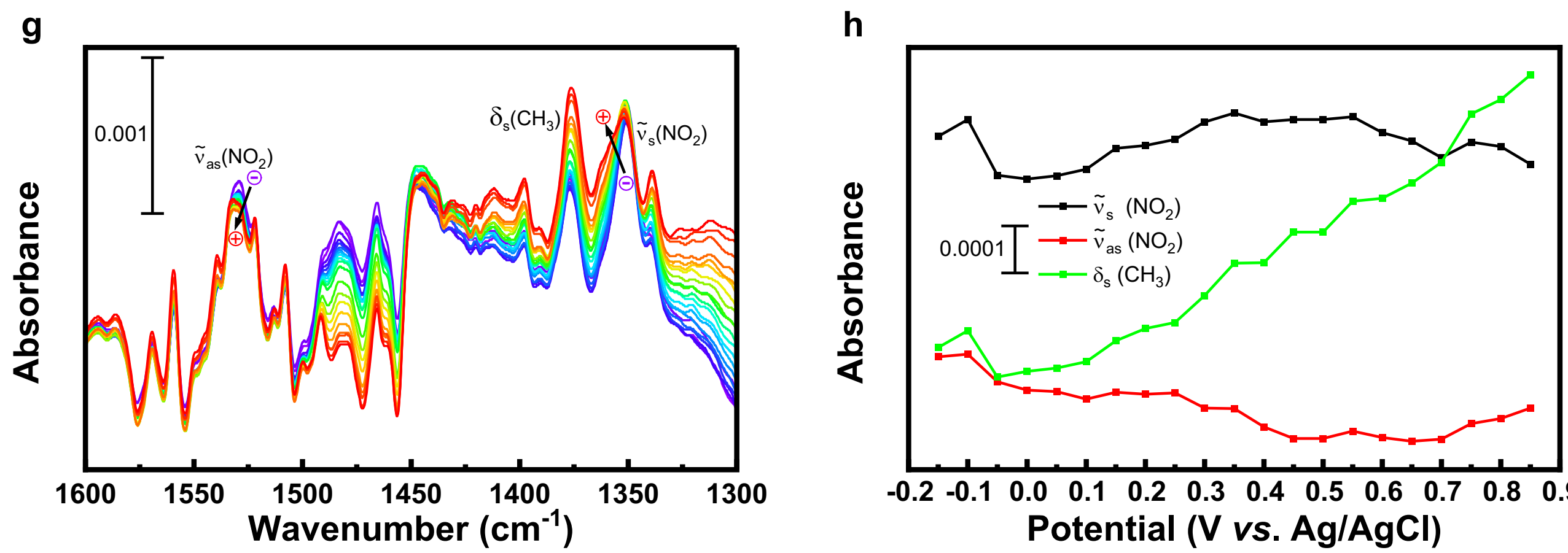

Figure S54. (a, c, e, g) Four replicate experiments of SEIRA spectra of surface-bound 3-NS_{ads} in 100 mM $TBAPF_6$ in MeCN on Pt in the absence of H_2 while going to more positive potentials from 0.0 to +1.0 V *vs*. OCV (purple to red) with respect to a background SEIRA spectrum measured of 100 mM $TBAPF_6$ in MeCN on Pt directly after applying +0.80 V *vs*. OCV. Spectra have been aligned at ca. 1750 cm^{-1} to show trends more clearly. SEIRA spectra in (a) have been reproduced from the main text Figure 3e and shown here for convenience. (b, d, f, h) Absorbance values of $\tilde{\nu}_s(NO_2)$ and $\tilde{\nu}_{as}(NO_2)$ of 3-NS, and $\delta_s(CH_3)$ of MeCN as a function of applied potential.

9.8.2 Reverse scan from more positive applied potentials to OCV

Figure S55 shows two replicate EC-SEIRAS spectra recorded for strongly surface-bound 3-NS on Pt immersed in a $TBAPF_6$/MeCN solution in the absence of H_2 as a function of applied potential during the reverse scans from positive applied potentials back to OCV. The data showed that the band intensity of the $\tilde{\nu}_s(NO_2)$ mode decreases when going to less positive applied potentials with a similar intensity change (but in opposite direction) as during the forward scan. The band intensity of the $\tilde{\nu}_{as}(NO_2)$ showed different tendencies in two replicate experiments, in one showing a further decrease with less positive applied potentials, meanwhile in the second experiment the intensity stayed relatively constant. Both the $\tilde{\nu}_s(NO_2)$ and $\tilde{\nu}_{as}(NO_2)$ of the 3-NS_{ads} in Figure S55 showed a red-shift of 1.7 cm^{-1} V^{-1} and 2.9 cm^{-1} V^{-1}, respectively, when going to less positive applied potentials. The band intensity of the $\delta_s(CH_3)$ mode of MeCN at 1375 cm^{-1} also decreased in intensity when going to less positive applied potentials.

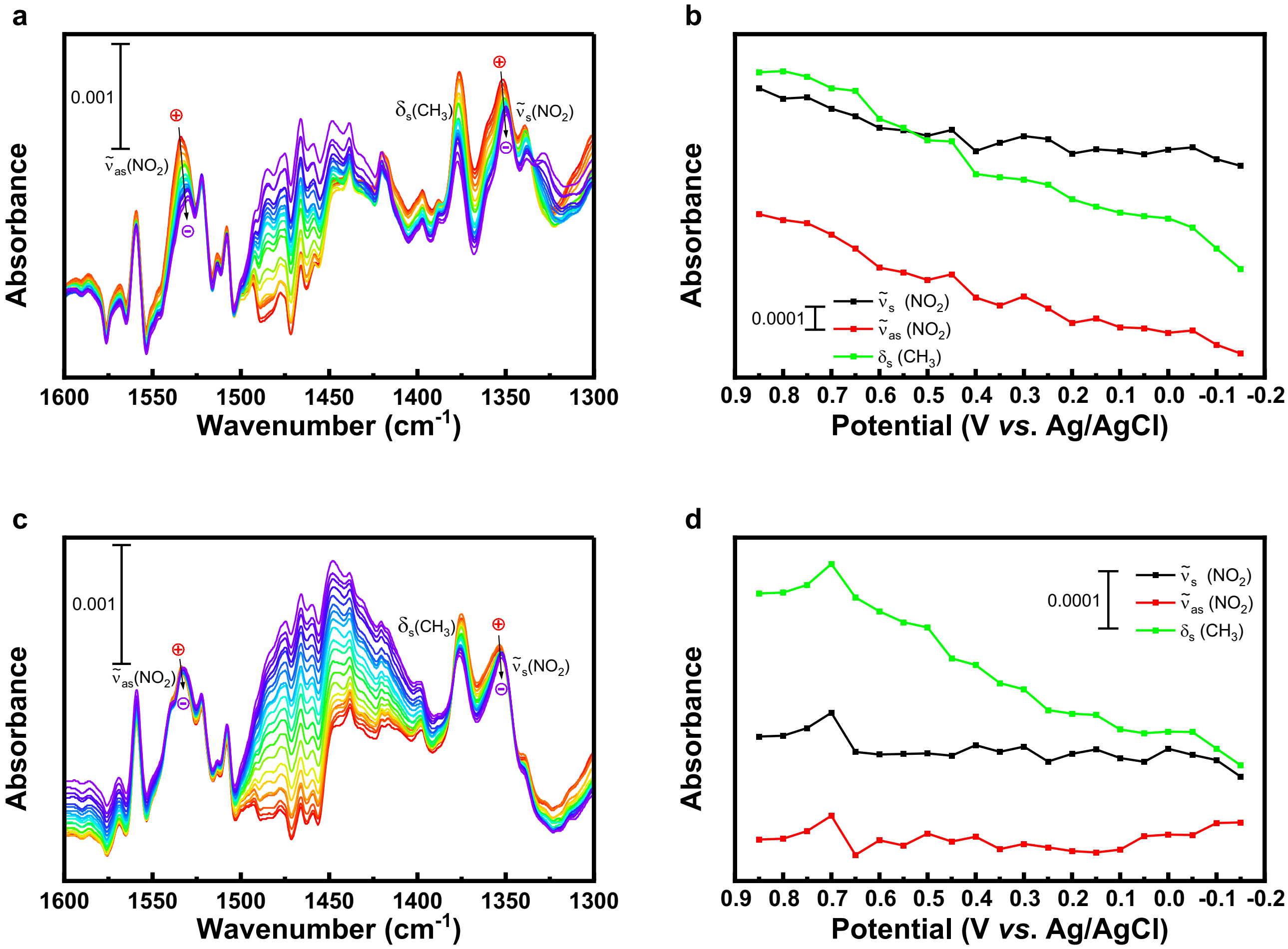

Figure S55. (a, c) Two replicate experiments of SEIRA spectra of surface-bound 3-NS$_{ads}$ in 100 mM TBAPF$_6$ in MeCN on Pt in the absence of H_2 while going to less positive potentials from +1.0 to 0.0 V *vs*. OCV (purple to red) with respect to a background SEIRA spectrum measured of 100 mM TBAPF$_6$ in MeCN on Pt directly after applying +0.80 V *vs*. OCV. Spectra have been aligned at ca. 1750 cm^{-1} to show trends more clearly. (b, d) Absorbance values of $\tilde{\nu}_s(NO_2)$ and $\tilde{\nu}_{as}(NO_2)$ of 3-NS, and $\delta_s(CH_3)$ of MeCN as a function of applied potential.

9.8.3 Discussion

Comparison of the EC-SEIRAS experiments of strongly surface-bound 3-NS$_{ads}$ only versus those in the presence of a 100 mM 3-NS solution shows that in the former case a much smaller change in $\tilde{\nu}_s(NO_2)$ and $\tilde{\nu}_{as}(NO_2)$ band intensities was observed. If an increase of the SEIRAS enhancement through surface reconstruction or surface roughening at more positive applied potential had been responsible for the large band intensity increase observed in presence of the 3-NS solution, we would have expected a similar band intensity increase for the experiments involving strongly surface-bound 3-NS$_{ads}$ only. However, this was not the case. Therefore, we attribute the large band intensity increase observed in presence of a 3-NS solution to an increase in the concentration of 3-NS on or near the surface, as discussed in SI section S9.7.3. This is in line with our consecutive OCV, −0.2 V, OCV experiments in the H-cell setup and the characterization of the Pt catalyst after catalysis that suggested no significant change of the Pt catalyst upon

applying a positive potential. The relatively small changes in $\tilde{\nu}_s(NO_2)$ and $\tilde{\nu}_{as}(NO_2)$ band intensities observed by the EC-SEIRAS experiments of strongly surface-bound 3-NS_{ads} described in this section are consistent with the lack of 3-NS in solution and hence no additional adsorption of 3-NS can take place. The relatively small changes in $\tilde{\nu}_s(NO_2)$ and $\tilde{\nu}_{as}(NO_2)$ band intensities observed by the EC-SEIRAS experiments of strongly surface-bound 3-NS_{ads} described in this section are further consistent with the idea that the 3-NS_{ads} are strongly surface-bound and hence do not desorb significantly under our conditions. *We mainly attribute the small intensity changes to changes in orientation of the strongly surface-bound 3-NS_{ads}.* We note that all this is also consistent with the observation of similarly large increase in band intensities for the $\delta_s(CH_3)$ mode of MeCN at more positive applied potentials, both when probing only strongly surface-bound 3-NS_{ads} as well as in presence of a 3-NS solution, since there is a large excess of MeCN available that can adsorb in both cases.

In SI section S9.6 we have shown that observation of both the $\tilde{\nu}_s(NO_2)$ as well as the $\tilde{\nu}_{as}(NO_2)$ mode of 3-NS when probing strongly surface-bound 3-NS_{ads} indicates that the ensemble of surface-bound species probed by EC-SEIRAS must include either a vertical κ^1-bound 3-NS or a tilted parallel adsorption mode of 3-NS. We have also discussed that the tilted parallel seems more likely based on prior theoretical work.[54,55] Here, we discuss the observed changes in intensity and wavenumber for the $\tilde{\nu}_s(NO_2)$ and $\tilde{\nu}_{as}(NO_2)$ modes as a function of applied potential.

To explain the observation of an increasing band intensity of the $\tilde{\nu}_s(NO_2)$ mode and a decreasing intensity of the $\tilde{\nu}_{as}(NO_2)$ mode with more positive applied potentials we consider possible orientation changes of the vertical κ^1-bound 3-NS or of the tilted parallel adsorption mode. For instance, a rotation of the vertical κ^1-bound 3-NS towards a κ^2-bound 3-NS could explain the observed intensity changes (Figure S56). However, in this case, we would expect that the larger backdonation obtained from the additional interaction of a Pt atom with a second oxygen of the NO_2-group should lead to a red-shift of both the $\tilde{\nu}_s(NO_2)$ and the $\tilde{\nu}_{as}(NO_2)$ modes.[54,55] This is contrary to the blue-shifts observed. This combined with prior work that suggests that κ^1-bound 3-NS has a small adsorption energy makes it unlikely that a rotation of the vertical κ^1-bound 3-NS explains our EC-SEIRAS observations.[54,55] Instead, a change in orientation of the tilted parallel adsorption mode would be consistent with both observed intensity changes as well as observed wavenumber shifts. Specifically, 3-NS could tilt up further (with the phenyl group moving away from the surface), which would allow rotation around the C_{vinyl}–C_{phenyl} bond (Figure S56). The stronger the activation of the vinyl-group, the more the binding of the vinyl-group could be described by a di–σ–type binding instead of a π–bonding.[56-58] Concomitantly, the hybridization of the vinyl–C atoms would be better described by a sp^3 symmetry rather than the original sp^2 symmetry. This symmetry change would reduce the barrier associated with the rotation around the C_{vinyl}–C_{phenyl} bond. This deviation from planarity in the 3-NS molecule would gradually make the $\tilde{\nu}_s(NO_2)$ mode more perpendicular with respect to the surface, while the $\tilde{\nu}_{as}(NO_2)$ mode would become more parallel. This would be consistent with a more intense $\tilde{\nu}_s(NO_2)$ mode and a less intense $\tilde{\nu}_{as}(NO_2)$ mode when going to more positive applied potentials. This change in orientation would also increase the distance between the Pt surface and the NO_2-group, which should decrease the backdonation from Pt into the anti–bonding π^* orbital of the NO_2-group and therefore lead to an observed blue-shift for both the $\tilde{\nu}_s(NO_2)$ and the $\tilde{\nu}_{as}(NO_2)$ modes. We stress again that this orientation change in the tilted parallel adsorption mode is also associated with an increased activation of the vinyl-group implying an enhanced affinity for the electron-rich vinyl group. This would be in line with the experimentally observed enhanced selectivity for the hydrogenation of this C=C bond over the NO_2-group at more positive applied potentials.

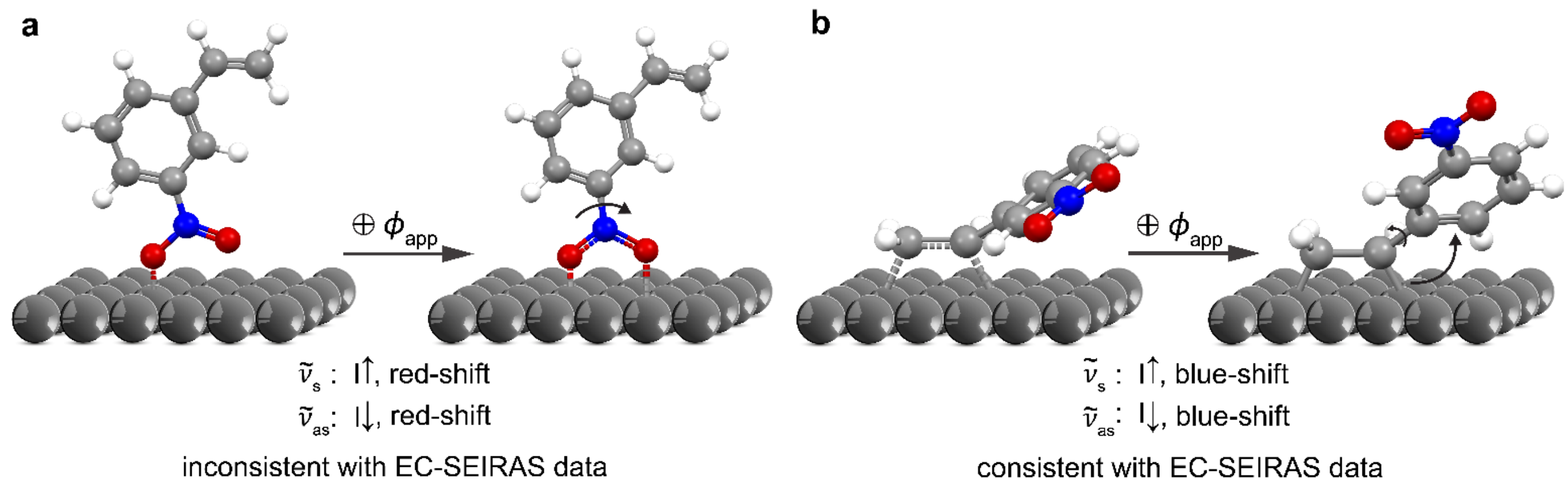

Figure S56. Visual representation of generally considered orientation changes of 3-NS_{ads} when going to more positive applied potentials that would be consistent with the observed increasing intensity of the $\tilde{\nu}_s(NO_2)$ mode and the decreasing intensity of the $\tilde{\nu}_{as}(NO_2)$ mode by EC-SEIRAS. (a) This orientation change includes a rotation of the vertical κ^1-bound 3-NS towards a κ^2-bound 3-NS. This should lead to red-shifts of both the $\tilde{\nu}_s(NO_2)$ and $\tilde{\nu}_{as}(NO_2)$ modes, which is inconsistent with what was observed. (b) This orientation change includes a tilting up of the phenyl ring away from the surface and a rotation around the C_{vinyl}–C_{phenyl} bond. This should lead to blue-shifts of both the $\tilde{\nu}_s(NO_2)$ and $\tilde{\nu}_{as}(NO_2)$ modes, which is consistent with what was observed. This figure is meant to merely illustrate the proposed reorientation and does not accurately reflect angles and distances of the real system. Arrows in (a) and (b) mark the orientation change that occurred when going from less to more positive applied potentials.

9.9 EC-SEIRAS data of 3-NS on Pt in the presence of H_2 as a function of applied potential

9.9.1 Forward scan from OCV to more positive applied potentials

Figure S57 shows EC-SEIRAS data of Pt immersed in a 100 mM 3-NS solution in the presence of H_2 as a function of applied potential for a forward scan going from OCV to more positive applied potentials measured with respect to a background SEIRA spectrum of 100 mM $TBAPF_6$ in MeCN on Pt in the presence of H_2 directly after applying +0.80 V *vs*. OCV (also see SI section S9.4.3 for further experimental details). The data showed an increase in band intensity of the $\tilde{\nu}_s(NO_2)$ and the $\tilde{\nu}_{as}(NO_2)$ modes of 3-NS with more positive applied potentials. This is qualitatively similar to what had been observed in the absence of H_2 (SI section S9.7.1), though, with a smaller relative intensity increase observed in the presence of H_2. Bands associated with TBA^+ decreased in intensity at more positive applied potentials as expected for a positively polarized surface that should reduce the TBA^+ concentration in the vicinity of the surface (Figure S58). Changes in the SEIRA spectra with applied potential could also be observed in the region of 2200–1900 cm^{-1} that includes modes associated with Pt–H. To analyze the potential dependence of Pt–H bands more clearly, the EC-SEIRA spectra obtained during the EC-SEIRA measurement of the 100 mM 3-NS solution in the presence of H_2 were also referenced to the SEIRA spectrum recorded of 100 mM $TBAPF_6$ in MeCN on Pt in the absence of H_2 obtained in the beginning of the experiment (SI section S9.4.3). These data showed a decrease in the intensity of the band associated with the Pt–H of adsorbed hydrogen atop Pt sites with more positive applied potentials (Figure S59, also see SI section S9.5 for band assignments). When more positive applied potentials were applied the current increased positively up to tens of μA. Similar trends were observed in two replicate experiments.

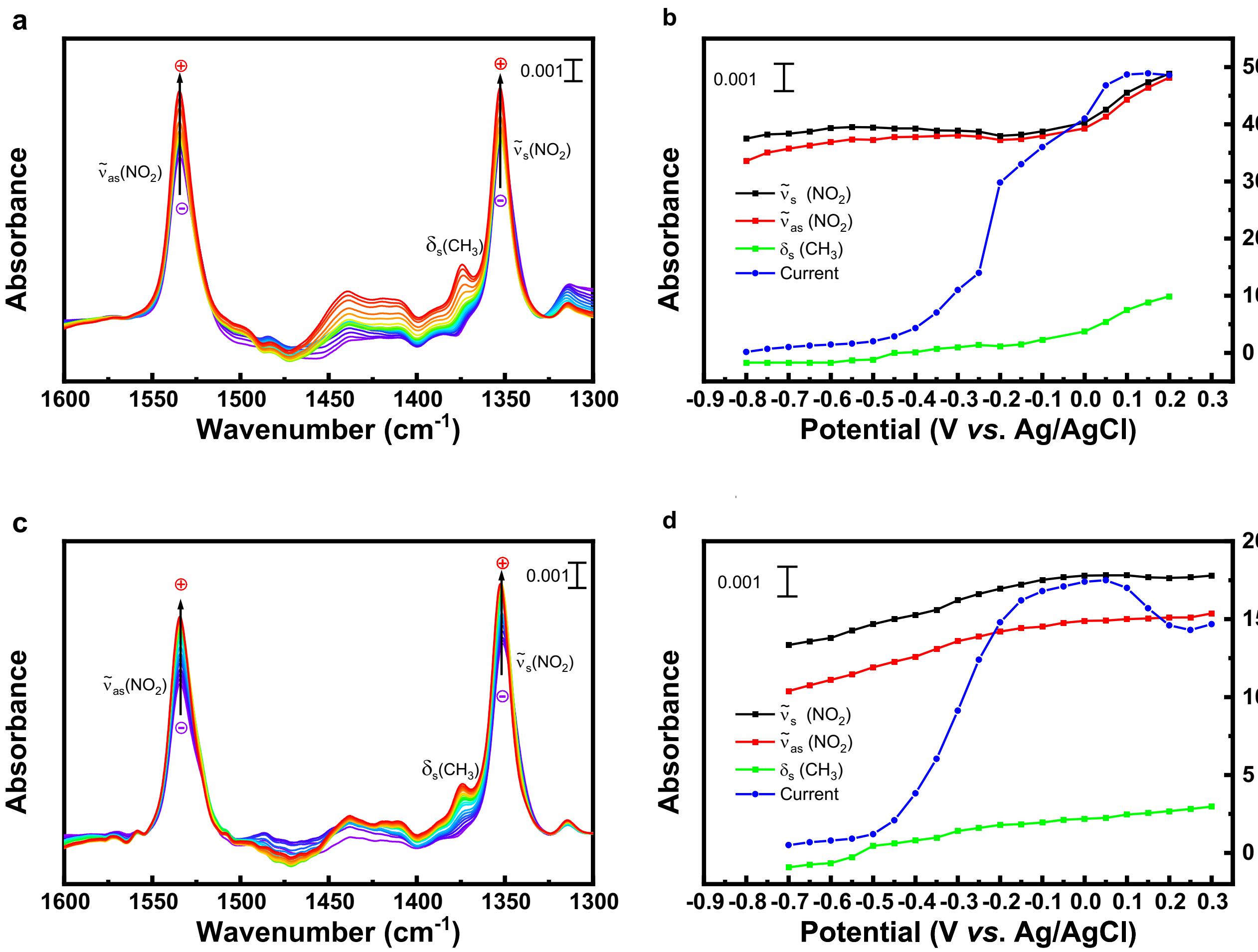

Figure S57. (a, c) Two replicate experiments of SEIRA spectra of 100 mM 3-NS and 100 mM $TBAPF_6$ in MeCN on Pt in the presence of H_2 while applying increasingly positive potentials from 0.0 to +1.0 V *vs*. OCV (purple to red) with respect to a background SEIRA spectrum measured of 100 mM $TBAPF_6$ in MeCN on Pt in the presence of H_2 directly after applying +0.80 V *vs*. OCV. SEIRA spectra have been background corrected with a multipoint background using anchor points at 1795, 1560, 1325, and 1270 cm^{-1}. One SEIRA spectrum at OCV and one at +1.0 V *vs*. OCV have been reproduced from the main text Figure 3c. (b, d) Absorbance values of the $\tilde{\nu}_s(NO_2)$ (black line, left y-axis) and $\tilde{\nu}_{as}(NO_2)$ bands (red line, left y-axis) of 3-NS, and $\delta_s(CH_3)$ of MeCN (green line, left y-axis) as a function of applied potential. Measured current at different applied potentials (blue line, right y-axis).

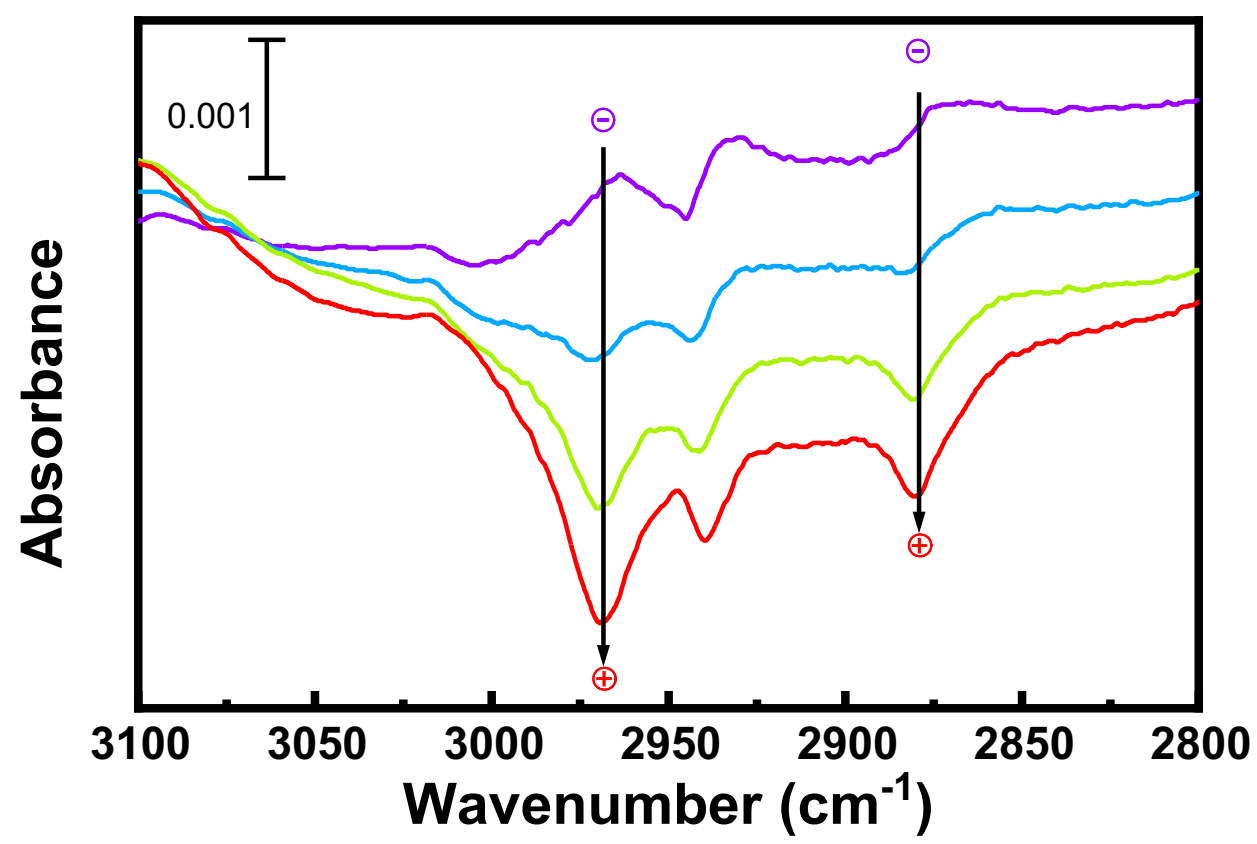

Figure S58. SEIRA spectra of 100 mM 3-NS and 100 mM $TBAPF_6$ in MeCN solution on Pt in the presence of H_2 while going from 0.00 V *vs*. OCV to more positive applied potentials (0.00, +0.20, +0.50, and +1.00 V *vs*. OCV, purple to red) during a forward scan. The SEIRA spectra were measured with respect to a background SEIRA spectrum measured of 100 mM $TBAPF_6$ in MeCN on Pt in the presence of H_2 directly after applying +0.80 V *vs*. OCV.

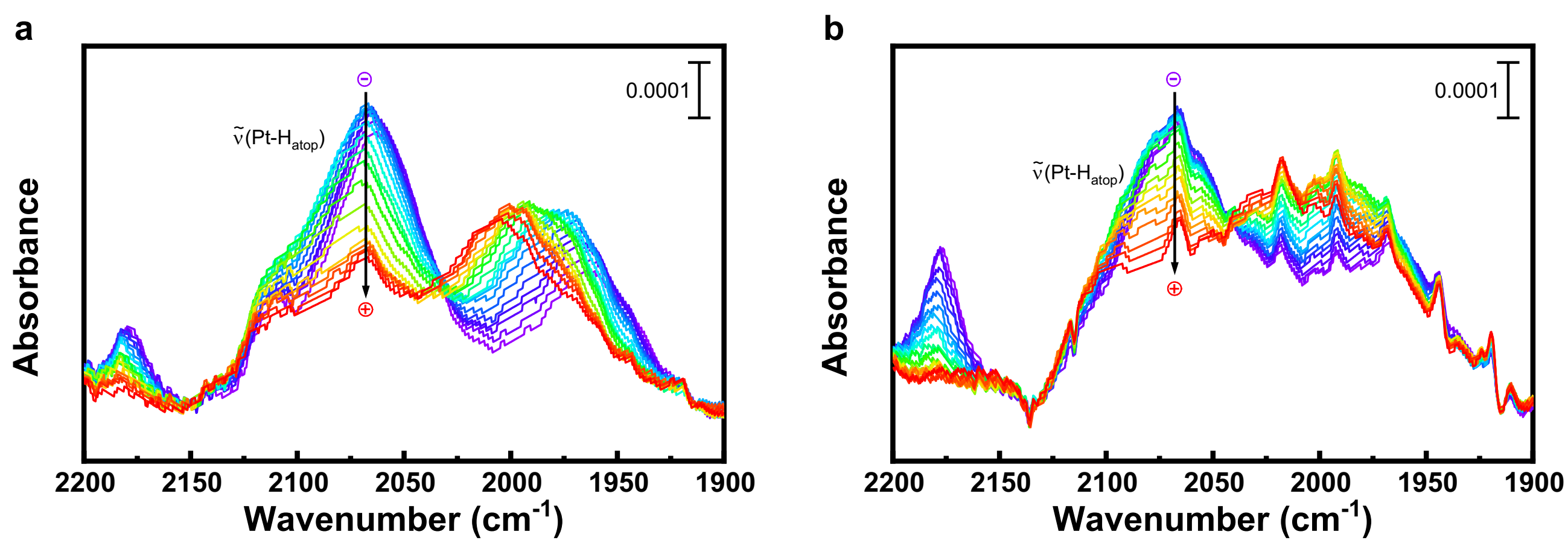

Figure S59. Two replicate SEIRA spectra of 100 mM 3-NS and 100 mM $TBAPF_6$ in MeCN on Pt in the presence of H_2 while applying increasingly positive potentials from 0.0 to +1.0 V *vs*. OCV (purple to red) with respect to a background SEIRA spectrum collected with 100 mM $TBAPF_6$ in MeCN on Pt in the absence of H_2. SEIRA spectra have been background corrected with a multipoint background using anchor points at 2480, 2425, 2215, 2135, 1915, and 1805 cm^{-1}.

As shown in SI section S9.4.3, applying a positive potential before the addition of 3-NS influenced the intensity of the Pt–H band. As mentioned above, the application of the positive applied potential before 3-NS addition to obtain a background spectrum was needed to reduce contributions of the MeCN solvent in the spectral regions associated with NO_2-vibrations. To test if similar results would be obtained if this step was omitted, we performed an EC-SEIRAS experiment, where 3-NS was directly added to the H_2-saturated solution of 100 mM $TBAPF_6$ in MeCN on Pt at OCV without first applying a positive potential. EC-SEIRA spectra were then recorded while applying positive potentials in the potential range from 0.0 V

to +1.0 V *vs*. OCV (forward scan) and then again back to less positive applied potentials to OCV (reverse scan, see SI section S9.9.2) with respect to the H_2-saturated solution of 100 mM $TBAPF_6$ in MeCN on Pt (further experimental details can be found in SI section S9.4.3). The data showed an increase of the band intensities of both the $\tilde{\nu}_s(NO_2)$ and the $\tilde{\nu}_{as}(NO_2)$ modes of 3-NS with more positive applied potentials, similarly to what was observed when a positive potential was first applied (cf. Figures S57 and S60). When no positive potential was first applied, the $\tilde{\nu}_s(NO_2)$ and $\tilde{\nu}_{as}(NO_2)$ intensities were lower in the beginning compared to what had been observed when a positive potential of +0.80 V *vs*. OCV was applied prior to the addition of 3-NS, though (cf. Figures S57 and S60). Figure S61 shows the potential dependence of the Pt–H band after referencing the obtained EC-SEIRA spectra to the solution of 100 mM $TBAPF_6$ in MeCN on Pt in the absence of H_2. The Pt–H band associated with adsorbed hydrogen at atop Pt sites decreased in intensity with more positive applied potential, qualitatively similarly to what was observed when first a positive potential was applied (cf. Figures S59 and S61).

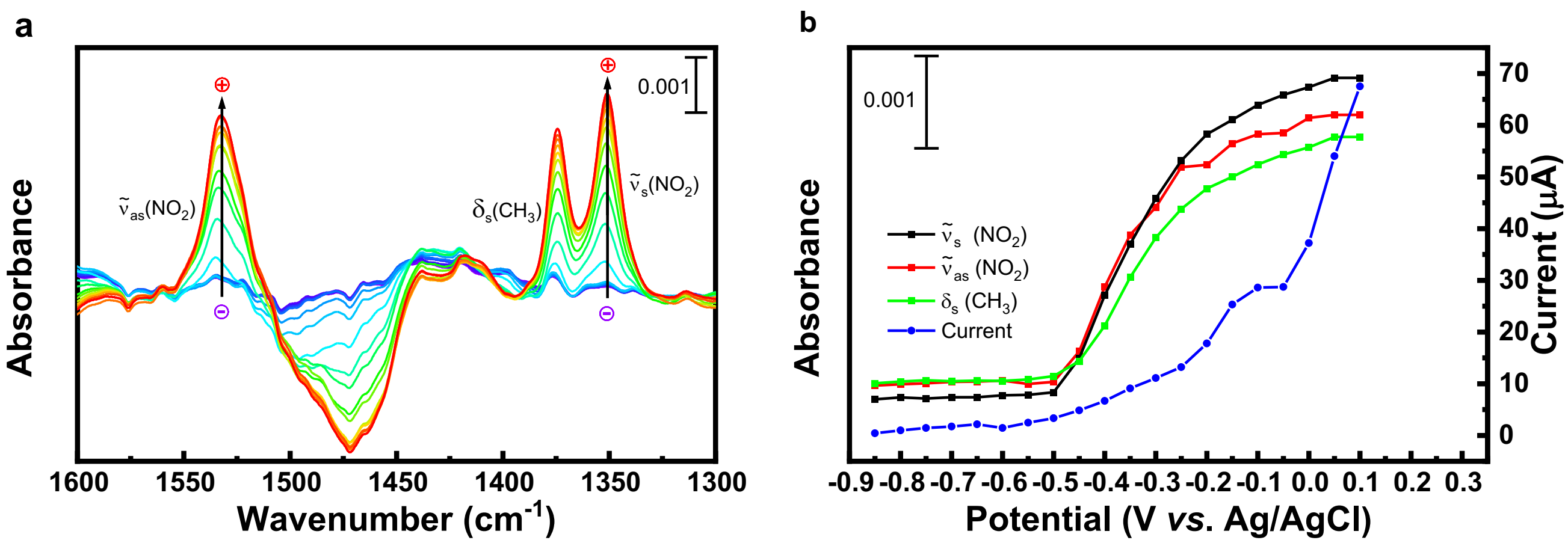

Figure S60. (a) SEIRA spectra of 100 mM 3-NS and 100 mM $TBAPF_6$ in MeCN on Pt in the presence of H_2 while applying increasingly positive potentials from 0.0 to +1.0 V *vs*. OCV (purple to red) with respect to a background SEIRA spectrum measured of 100 mM $TBAPF_6$ in MeCN on Pt in the presence of H_2 at OCV. No positive potential of +0.80 V *vs*. OCV was applied prior to the addition of 3-NS. SEIRA spectra have been background corrected with a multipoint background using anchor points at 1795, 1560, 1320, and 1305 cm^{-1}. (b) Absorbance values of the $\tilde{\nu}_s(NO_2)$ (black line, left y-axis) and $\tilde{\nu}_{as}(NO_2)$ bands (red line, left y-axis) of 3-NS, and $\delta_s(CH_3)$ of MeCN (green line, left y-axis) as a function of applied potential. Measured current at different applied potentials (blue line, right y-axis).

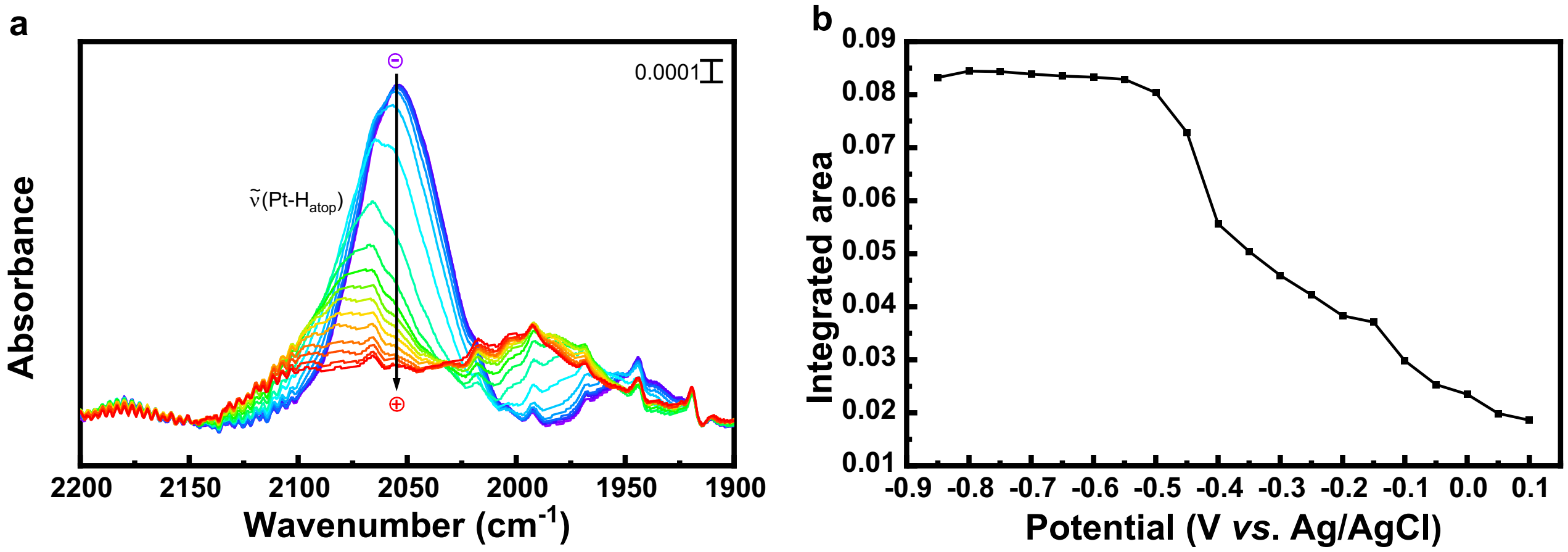

Figure S61. (a) SEIRA spectra of 100 mM 3-NS and 100 mM $TBAPF_6$ in MeCN on Pt in the presence of H_2 while applying increasingly positive potentials from 0.0 to +1.0 V *vs*. OCV (purple to red) with respect to a background SEIRA spectrum collected with 100 mM $TBAPF_6$ in MeCN on Pt in the absence of H_2. No positive potential of +0.80 V *vs*. OCV was applied prior to the addition of 3-NS. Figure S61a has been reproduced from the main text, Figure 3d, and shown here for convenience. SEIRA spectra have been background corrected with a multipoint background using anchor points at 2480, 2315, 2215, 2145, 1910, and 1815 cm^{-1}. (b) Integrated area of the Pt–H_{atop} bands as a function of applied potential.

9.9.2 Reverse scan from more positive applied potentials to OCV

Figure S62 shows EC-SEIRAS data obtained for Pt immersed in a 3-NS solution in the presence of H_2 as a function of applied potential for a reverse scan when going from more positive applied potentials back to OCV measured with respect to a background SEIRA spectrum of 100 mM $TBAPF_6$ in MeCN on Pt in the presence of H_2 directly after applying +0.80 V *vs*. OCV. In contrast to what was observed in absence of H_2 when band intensities remained relatively constant (SI section S9.7.2 and Figure S52), the data showed a sharp decrease in band intensity when going to less positive applied potentials for both the $\tilde{\nu}_s(NO_2)$ and the $\tilde{\nu}_{as}(NO_2)$ modes of 3-NS. Bands associated with TBA^+ increased in intensity at less positive applied potentials as expected for a less positively polarized surface that should increase the TBA^+ concentration in the vicinity (Figure S63). At more positive applied potentials we recorded positive currents up to ca. 10–150 μA that decreased when going to less positive applied potentials. Similar qualitative trends were observed in two replicate experiments.

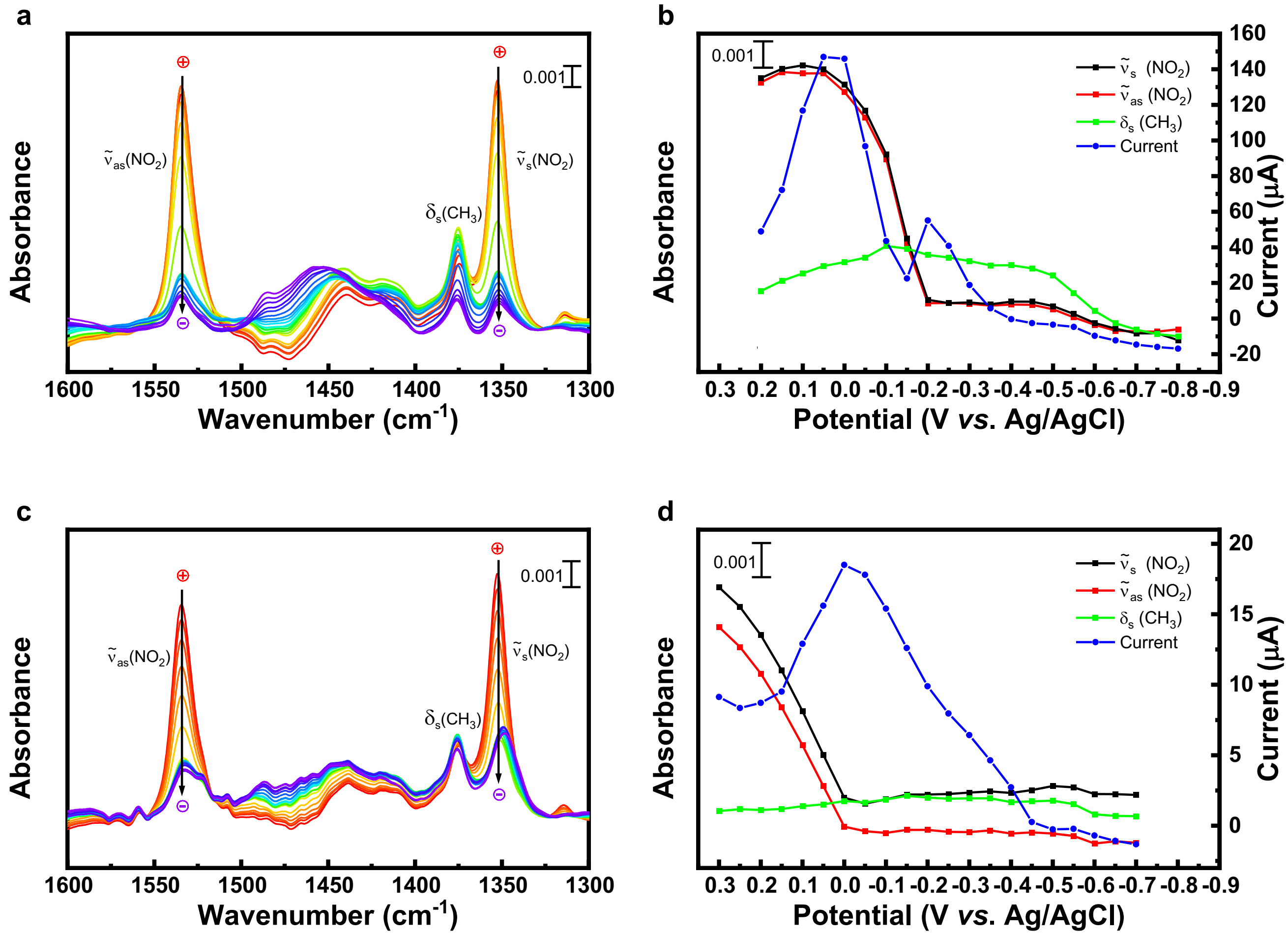

Figure S62. (a, c) Two replicate experiments of SEIRA spectra of 100 mM 3-NS and 100 mM $TBAPF_6$ in MeCN on Pt in the presence of H_2 while going from more positive applied potentials of +1.0 V *vs*. OCV to 0.0 V *vs*. OCV (red to purple) with respect to a background SEIRA spectrum measured of 100 mM $TBAPF_6$ in MeCN on Pt in the presence of H_2 directly after applying +0.80 V *vs*. OCV. SEIRA spectra have been background corrected with a multipoint background using anchor points at 1795, 1560, 1325, and 1270 cm^{-1}. One SEIRA spectrum at OCV and one at +1.0 V *vs*. OCV have been reproduced from the main text Figure 3c. (b, d) Absorbance values of the $\tilde{\nu}_s(NO_2)$ (black line, left y-axis) and $\tilde{\nu}_{as}(NO_2)$ bands (red line, left y-axis) of 3-NS, and $\delta_s(CH_3)$ of MeCN (green line, left y-axis) as a function of applied potential. Measured current at different applied potentials (blue line, right y-axis).

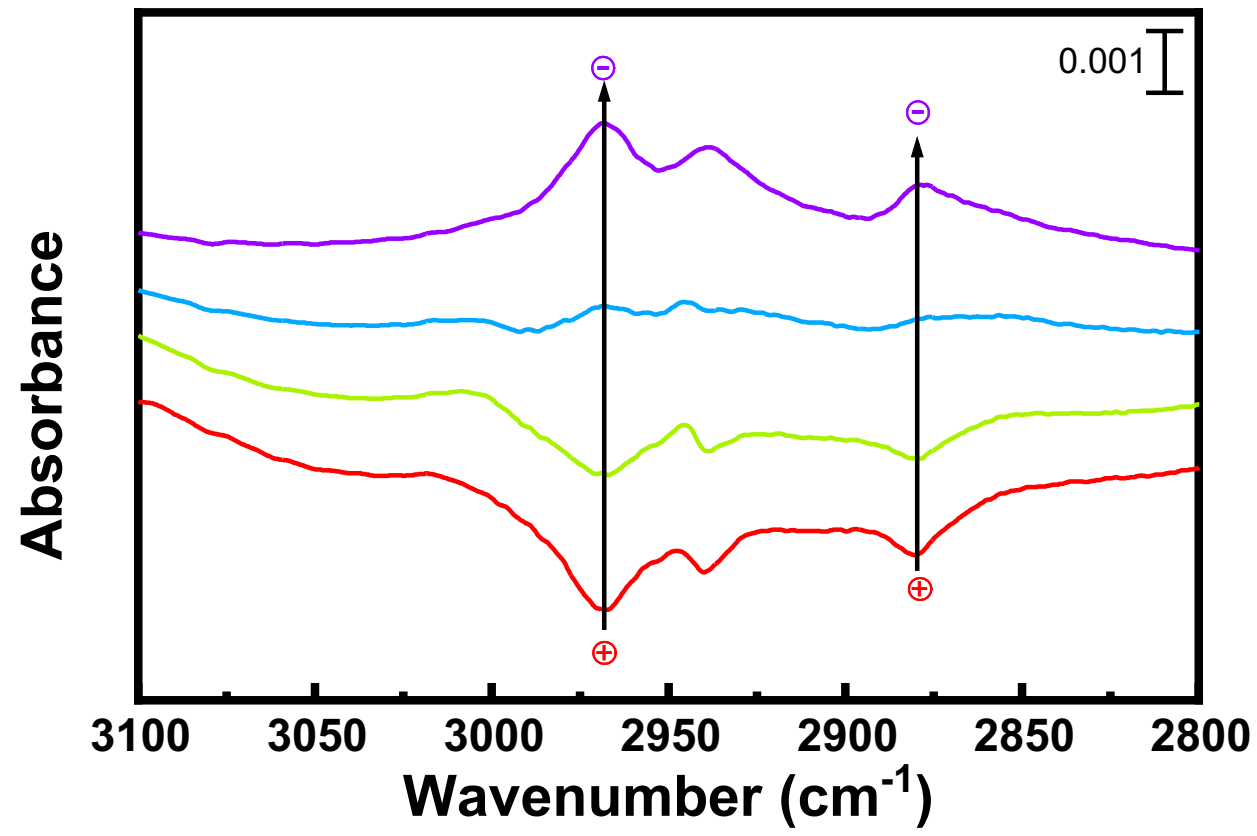

Figure S63. SEIRA spectra of 100 mM 3-NS and 100 mM $TBAPF_6$ in MeCN solution on Pt in the presence of H_2 while going from more positive applied potentials to OCV (+1.00, +0.55, +0.25, and 0.00 V *vs*. OCV, red to purple) during a reverse scan. The SEIRA spectra were measured with respect to a background SEIRA spectrum measured of 100 mM $TBAPF_6$ in MeCN on Pt in the presence of H_2 directly after applying +0.80 V *vs*. OCV.

To analyze the potential dependence of Pt–H bands more clearly, the EC-SEIRA spectra obtained during the EC-SEIRA measurement of the 100 mM 3-NS solution in the presence of H_2 during the reverse scan were referenced to the SEIRA spectrum recorded of 100 mM $TBAPF_6$ in MeCN on Pt in the absence of H_2 and are shown in Figure S64. The Pt–H_{atop} band intensity increased when going to less positive potentials, in a qualitatively reversible manner compared to the forward scan (cf. Figures. S64 and S59).

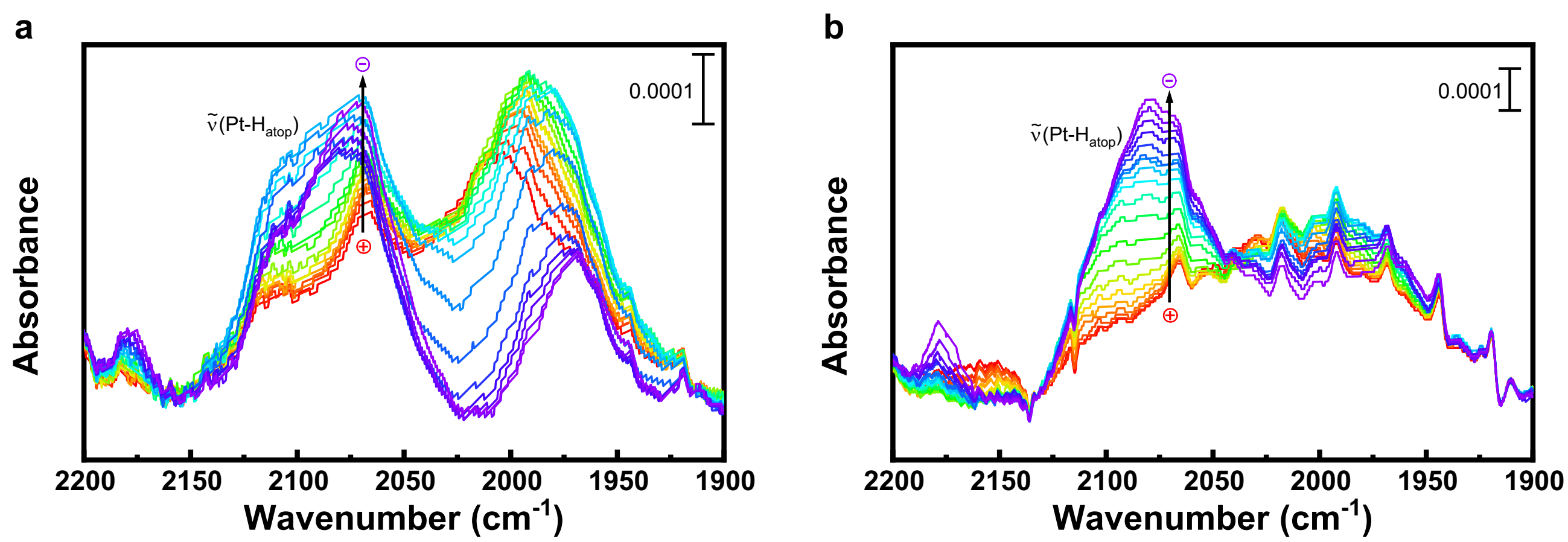

Figure S64. Two replicate EC-SEIRAS experiments showing EC-SEIRA spectra of 100 mM 3-NS and 100 mM $TBAPF_6$ in MeCN on Pt in the presence of H_2 while going from more positive applied potentials of +1.0 V *vs*. OCV to 0.0 V *vs*. OCV (red to purple) with respect to a background SEIRA spectrum collected with 100 mM $TBAPF_6$ in MeCN on Pt in the absence of H_2. SEIRA spectra have been background corrected with a mulitpoint background using anchor points at 2480, 2425, 2215, 2135, 1915, and 1805 cm^{-1}.

Figure S65 shows EC-SEIRAS data obtained for Pt immersed in a 3-NS solution in the presence of H_2 as a function of applied potential for a reverse scan when going from more positive applied potentials back to OCV, where no positive applied potential of +0.80 V *vs*. OCV was applied prior to the addition of 3-NS. This shows a gradual decrease in band intensity for both the $\tilde{\nu}_s(NO_2)$ and the $\tilde{\nu}_{as}(NO_2)$ modes of 3-NS when going to less positive applied potentials. This is qualitatively similar to what was observed when a positive potential was applied prior to the EC-SEIRAS measurements as function of an applied potential (cf. Figures S62 and S65). Figure S66 shows the potential dependence of the Pt–H band after referencing the obtained EC-SEIRA spectra during the reverse scan to the SEIRAS spectrum measured of the solution of 100 mM $TBAPF_6$ in MeCN on Pt in the absence of H_2. The Pt–H_{atop} band increased in intensity with less positive applied potential, in a qualitatively but not quantitatively reversible manner compared to the forward scan (cf. Figures. S61 and S66). These observations are similar to what was observed when a positive potential was applied prior to the EC-SEIRAS measurements as function of an applied potential (cf. Figures. S64 and S66).

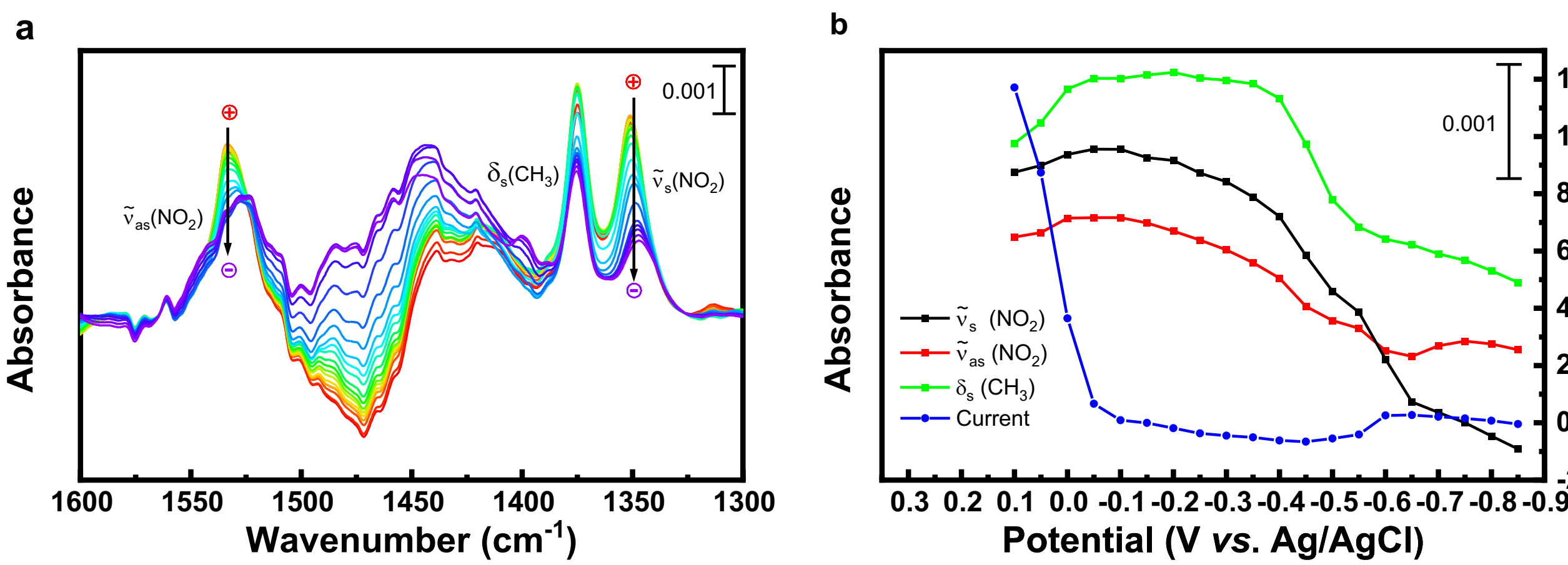

Figure S65. (a) SEIRA spectra of 100 mM 3-NS and 100 mM $TBAPF_6$ in MeCN on Pt in the presence of H_2 while going from more positive applied potentials of +1.0 V *vs*. OCV to 0.0 V *vs*. OCV (red to purple) with respect to a background SEIRA spectrum measured of 100 mM $TBAPF_6$ in MeCN on Pt in the presence of H_2 at OCV. No positive potential of +0.80 V *vs*. OCV was applied prior to the addition of 3-NS. SEIRA spectra have been background corrected with a mulitpoint background using anchor points at 1795, 1560, 1320, and 1305 cm^{-1}. (b) Absorbance values of the $\tilde{\nu}_s(NO_2)$ (black line, left y-axis) and $\tilde{\nu}_{as}(NO_2)$ bands (red line, left y-axis) of 3-NS, and $\delta_s(CH_3)$ of MeCN (green line, left y-axis) as a function of applied potential. Measured current at different applied potentials (blue line, right y-axis).

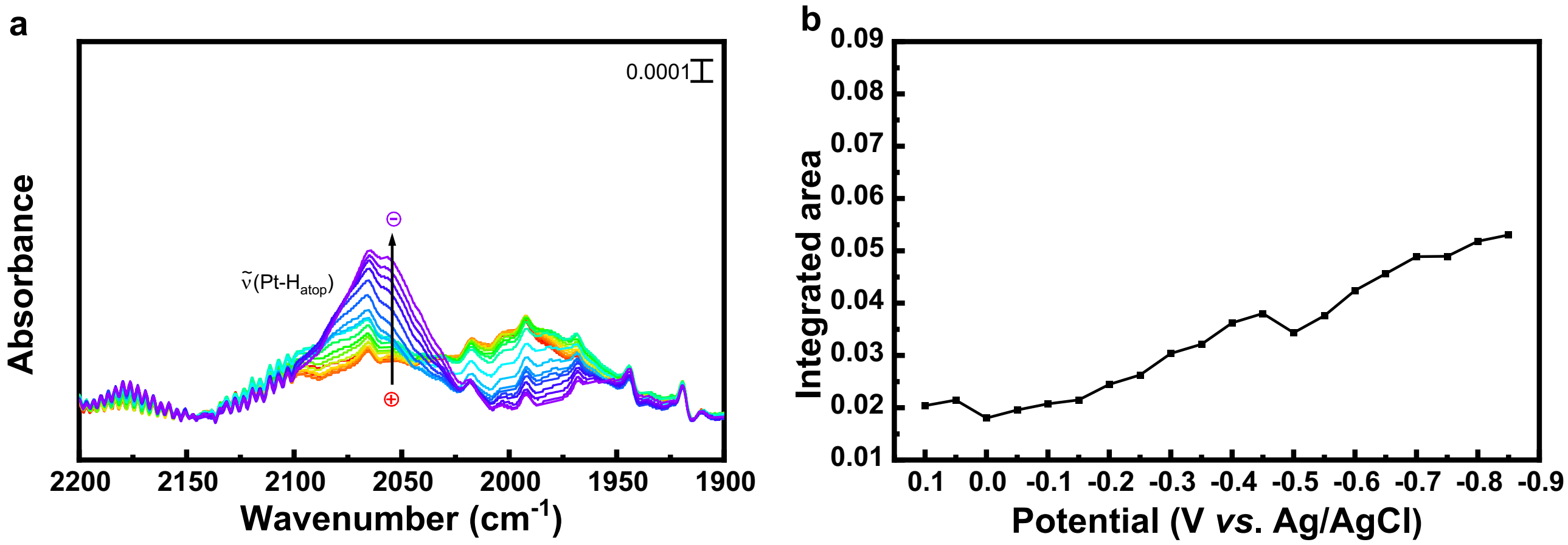

Figure S66. (a) SEIRA spectra of 100 mM 3-NS and 100 mM $TBAPF_6$ in MeCN on Pt in the presence of H_2 while going from more positive applied potentials of +1.0 V *vs*. OCV to 0.0 V *vs*. OCV (red to purple) with respect to a background SEIRA spectrum collected with 100 mM $TABPF_6$ in MeCN on Pt in the absence of H_2. No positive potential of +0.80 V *vs*. OCV was applied prior to the addition of 3-NS. SEIRA spectra have been background corrected with a multipoint background using anchor points at 2480, 2315, 2215, 2145, 1910, and 1815 cm^{-1}. (b) Integrated area of the Pt–H_{atop} band as a function of applied potential.

9.9.3 Discussion

Similarly to what was observed in absence of H_2, we observed increased band intensities for the $\tilde{\nu}_s(NO_2)$ and the $\tilde{\nu}_{as}(NO_2)$ modes of 3-NS when going to more positive applied potentials in the forward scan. As discussed in SI section S9.7.3, the increased $\tilde{\nu}_s(NO_2)$ and $\tilde{\nu}_{as}(NO_2)$ band intensities at more positive applied potentials can be attributed to a promoted adsorption of 3-NS. These data hence show that the 3-NS adsorption is promoted at more positive applied potentials in absence as well as in the presence of H_2. However, there are also some notable differences observed in the presence of H_2 compared to the EC-SEIRAS data obtained in absence of H_2 (SI section S9.7):

(i) A decrease of band intensities for the $\tilde{\nu}_s(NO_2)$ and the $\tilde{\nu}_{as}(NO_2)$ modes of 3-NS when going back to less positive applied potentials in the reverse scan was observed in the presence of H_2 but not in absence of H_2. We tentatively attribute this observation to the fast reaction of 3-NS with H_2 to form reaction products that subsequently desorb from the surface or a competitive adsorption of 3-NS and hydrogen at the surface. Either effect would decrease the surface concentration of 3-NS. This decrease in 3-NS surface concentration can be more effectively compensated with a promoted adsorption of 3-NS at more positive applied potentials, which leads to the overall higher band intensities of 3-NS at more positive applied potentials.

(ii) A second notable difference to the EC-SEIRAS data obtained in the absence of H_2 is the observation of significant positive currents at more positive applied potentials during EC-SEIRAS measurements in the presence of H_2. In the absence of H_2 only very small positive currents (<10 μA) were observed. SEIRAS bands associated with Pt–H can only be observed in the presence of H_2. These bands showed a decrease in intensity with when going from 0.0 V to +1.0 V *vs*. OCV. Measured currents and Pt–H band intensities combined are consistent with a lower H* concentration at more positive potentials due to concurrently occurring HOR. This agrees with results obtained in our standard H-cell setup.

More detailed insight may come from a comparison of EC-SEIRAS data obtained using a positive applied potential of +0.80 V prior to the addition of 3-NS (Figure S57) versus data obtained when no potential was applied prior to the experiment (Figure S60). In the former case, the $\tilde{\nu}_s(NO_2)$ and $\tilde{\nu}_{as}(NO_2)$ bands were observed at some intensity even at OCV and increased less with positive applied potentials. Without application of a positive applied potential, only very small $\tilde{\nu}_s(NO_2)$ and $\tilde{\nu}_{as}(NO_2)$ bands can be observed at OCV but these increase more with positive applied potentials. These differences can perhaps be understood by analyzing the Pt–H bands. These bands were much more intense when no positive potential was applied before the measurement (cf. Figures S59 and S61). This could indicate that H* and 3-NS adsorb competitively on Pt in agreement with our findings on varying 3-NS concentration in an H-cell as well as with related prior literature.[59]

9.10 EC-SEIRAS data of 3-NS on Pt in presence of H_2 at OCV monitored as a function of time, involving strongly surface-bound 3-NS only

To obtain further information on the reaction of surface-bound 3-NS with H_2, we measured EC-SEIRAS of strongly surface-bound 3-NS_{ads} only at OCV while purging H_2, monitored as a function of time (also see SI section S9.4.4 for experimental details). A gradual decrease in band intensity of both the $\tilde{\nu}_s(NO_2)$ and the $\tilde{\nu}_{as}(NO_2)$ modes of 3-NS_{ads} were observed with a disappearance of the bands observed within ca. 15 min (Figure S67a). Simultaneously a decrease in E_{OCV} was observed from ca. −0.25 to −0.75 V *vs*. Ag/AgCl (Figure S67b). Figure S68 shows the Pt–H band associated with terminal bound hydrogen at atop Pt sites before and during $H_{2(g)}$ purge after referencing the obtained EC-SEIRA spectra to the solution of 100 mM $TBAPF_6$ in MeCN on Pt in the absence of H_2. After the solution exchange, before $H_{2(g)}$ is being purged only a very low intensity Pt–H_{atop} band is visible, suggesting that through the positive applied potential (SI section S9.4.4) and solution exchange the majority of the adsorbed hydrogen (stemming from the preceding experiment involving EC-SEIRAS of 3-NS on Pt in the presence of H_2) was removed. The Pt–H_{atop} band increased gradually while purging $H_{2(g)}$ and reached roughly the same integrated area value after 25 min as obtained in the SEIRAS experiment with 100 mM 3-NS in the presence of H_2 at less positive applied potentials close to OCV (cf. Figure S61).

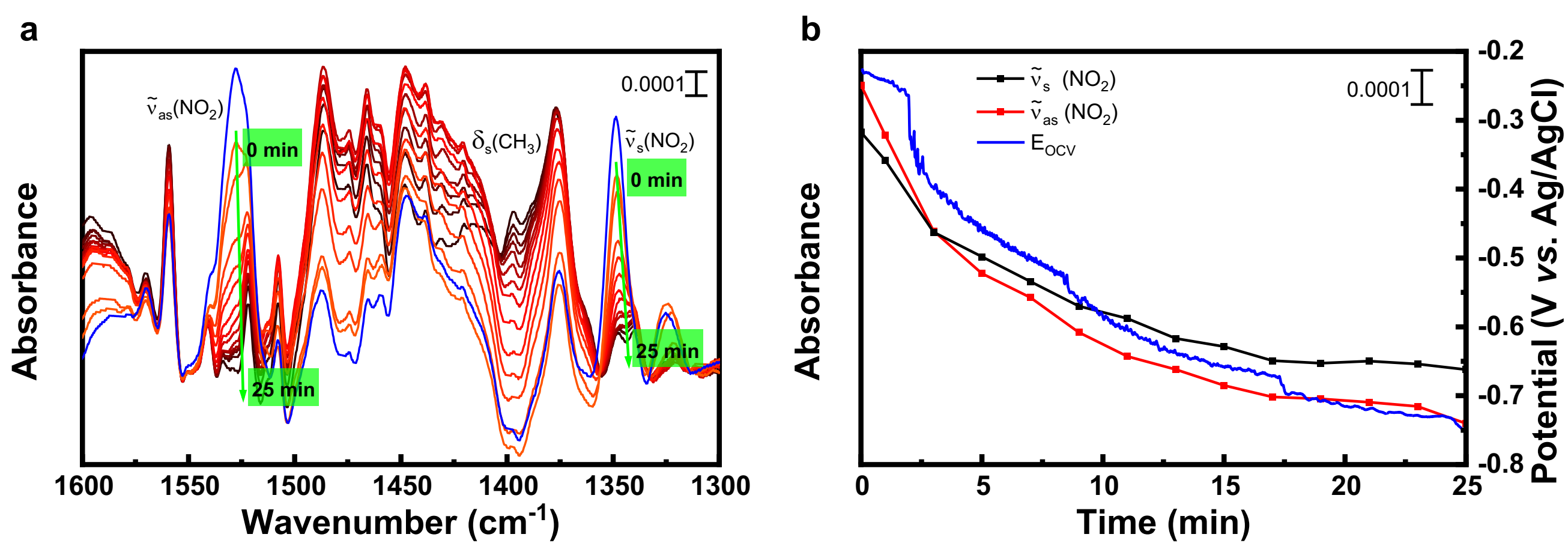

Figure S67. (a) SEIRA spectra of surface-bound 3-NS_{ads} in 100 mM $TBAPF_6$ in MeCN on Pt before (blue line) and during purging of $H_{2(g)}$ measured at OCV as a function of time from 0 to 25 min (light red to dark red) with respect to a background SEIRA spectrum measured of 100 mM $TBAPF_6$ in MeCN on Pt in the presence of H_2 directly after applying +0.80 V *vs*. OCV. SEIRA spectra have been background corrected with a multipoint background using anchor points at 1795, 1550, 1330, and 1300 cm^{-1} (b) Absorbance values of the $\tilde{\nu}_s(NO_2)$ (black line, left y-axis) and the $\tilde{\nu}_{as}(NO_2)$ band (red line, left y-axis) of 3-NS and E_{OCV} (blue line, right y-axis) as a function of time.

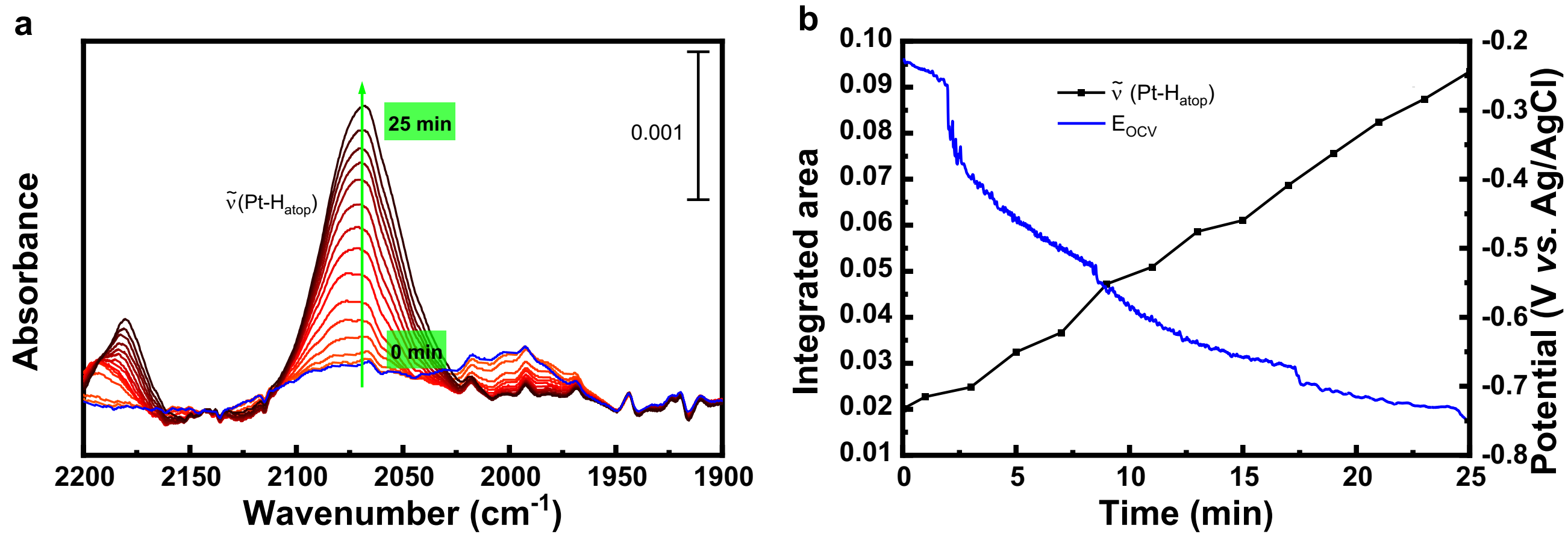

Figure S68. SEIRA spectra showing the increasing Pt–H band with time obtained during the experiments involving surface-bound 3-NS$_{ads}$ in 100 mM TBAPF$_6$ in MeCN on Pt before (blue line) and during purging of $H_{2(g)}$ measured at OCV as a function of time from 0 to 25 min (light red to dark red) with respect to a background SEIRA spectrum collected with 100 mM TBAPF$_6$ in MeCN on Pt in the absence of H_2. SEIRA spectra have been background corrected with a multipoint background using anchor points at 2480, 2425, 2315, 2215, 2145, 1950, and 1805 cm^{-1}. (b) Integrated area of the Pt–H$_{atop}$ band (black line, left y-axis) and E$_{OCV}$ (blue line, right y-axis) as a function of applied potential.

The observed disappearance of the $\tilde{\nu}_s(NO_2)$ and the $\tilde{\nu}_{as}(NO_2)$ bands of 3-NS$_{ads}$ in the presence of H_2 within 15 min is different to the small and reversible intensity changes that had been observed for strongly surface-bound 3-NS in the absence of H_2 on a time scale of roughly 30 min (cf. SI section S9.8). Based on our experiments in an H- or single-compartment cell that show that 3-NS can be hydrogenated to different products under similar reaction conditions, we attribute the disappearance of the $\tilde{\nu}_s(NO_2)$ and the $\tilde{\nu}_{as}(NO_2)$ bands of strongly surface-bound 3-NS in the presence of H_2 to the reaction of 3-NS with H_2 to form hydrogenation products and the subsequent desorption of these products from the surface. The EC-SEIRAS data hence seem to suggest that the strongly surface-bound 3-NS$_{ads}$ can be hydrogenated. However, based on the SEIRAS data on their own a simple exchange of adsorbed 3-NS with H_2 cannot be absolutely excluded.

9.11 Conclusions from EC-SEIRAS studies and relevance of these studies in the context of standard H-cell experiments of catalytic 3-NS hydrogenation

The EC-SEIRAS data discussed above suggest that (i) the 3-NS adsorption is promoted at more positive applied potentials, both in the absence as well as in the presence of H_2, (ii) the ensemble of surface-bound 3-NS includes a tilted parallel adsorption mode, (iii) positive applied potentials induce a change in the orientation of the surface-adsorbed 3-NS that is associated with a stronger activation of the C=C group by the Pt surface, and (iv) the H-coverage is lower at more positive applied potentials compared to less positive applied potentials.

While performing EC-SEIRAS experiments required us to deviate somewhat from our standard H-cell conditions (i.e., use of a relatively flat and small-area Pt electrode on Au for EC-SEIRAS versus the Pt deposited on a Ni foam, the use of a single-compartment spectroelectrochemical cell under EC-SEIRAS conditions versus the typical H-cell conditions, and no stirring under SEIRAS conditions versus stirring in the H-cell), most of the other reactions conditions including substrate and electrolyte concentration, catalyst identity, H_2 flow rate, and solvent were similar in the two experimental setups. EC-SEIRAS data seemed to suggest that the observed surface-adsorbed 3-NS molecules can participate in hydrogenation reactions and may hence be catalytically relevant. We further showed that HOR processes are obtained at more positive applied potentials both in the EC-SEIRAS setup as well as under standard H-cell conditions. Combined, these data suggest that while differences in results are expected due to differences in conditions used, results obtained using the EC-SEIRAS setup are likely relevant to results obtained in H-cell conditions.

Therefore, the effects (i-iv) inferred from EC-SEIRAS data could contribute to the observed higher hydrogenation catalysis yields at more positive applied potentials (effects i and iv), and the higher selectivity for 3-ENB from the hydrogenation of the vinyl-group of 3-NS at more positive applied potentials (effects ii and iii), which were both observed in our standard H-cell catalytic experiments.

10. Supplementary data on the catalytic hydrogenation of other substrates and using other catalysts

10.1 Testing catalytic hydrogenation of other substrates as a function of applied potential

To test the effect on the applied potential on overhydrogenation as well as to further investigate the affinity of Pt for the electron-rich C=C over electron-deficient NO_2-groups in different substrates, we probed the catalytic hydrogenation of 3-ENB with H_2 using Pt as catalyst at both OCV (ca. −1 V) and at positive applied potential (-0.2 V). 3-ENB hydrogenation was done with 9 mM 3-ENB added to both working and counter chamber. Otherwise, we used similar experimental conditions as used for the catalytic hydrogenation of 3-NS described in the SI sections S3.3 and S3.4.

Table S16. Yield and selectivity for the catalytic hydrogenation of 3-ENB using Pt at OCV or at −0.2 V.

Substrate	Reaction condition	Product	Selectivity (%)	Yield (%)
3-ENB	OCV	3-EAOH	27.1	6.1
		3-EA	72.9	16.3
	−0.2 V	No product		

To test whether our observations also apply to hydrogenation reactions of other substrates, we probed the effect of an applied potential on the catalytic hydrogenation of styrene, nitrobenzene, a 1:1 mixture of styrene and nitrobenzene, and *trans*-cinnamaldehyde using Pt as catalyst. For this, we used similar experimental conditions as used for the catalytic hydrogenation of 3-NS described in the SI section S3. The experiments were done with 100 mM styrene, 100 mM nitrobenzene, 50 mM styrene and 50 mM nitrobenzene, or 100 mM *trans*-cinnamaldehyde. We tested catalysis in each case at OCV (ca. −1 V), −0.6 V, and −0.2 V. These data are shown in Figures S69-S71 and Table S17.

We also tested other styrenyl-based substrates with other functional group combinations as well as substrates with non-conjugated olefin groups, namely 3-vinylanisole, 3-bromostyrene, and 4-vinylbenzoic acid as well as 2-allylbenzaldehyde, 1-allyl-4-methoxybenzene, and 4-pentenal, respectively (Table S18 and Figure S72). Furthermore, we also tested 5-nitroindole as an additional substrate (Table S18). The experiments were done with 100 mM of the substrate, except in case of 4-vinylbenzoic acid where 13 mM solution of 4-vinylbenzoic acid was used due to a limited availability and limited solubility of the substrate in acetonitrile. We tested catalysis in each case at OCV (ca. −1 V) and at −0.2 V and otherwise similar experimental conditions as used for the catalytic hydrogenation of 3-NS described in the SI sections S3.3 and S3.4.

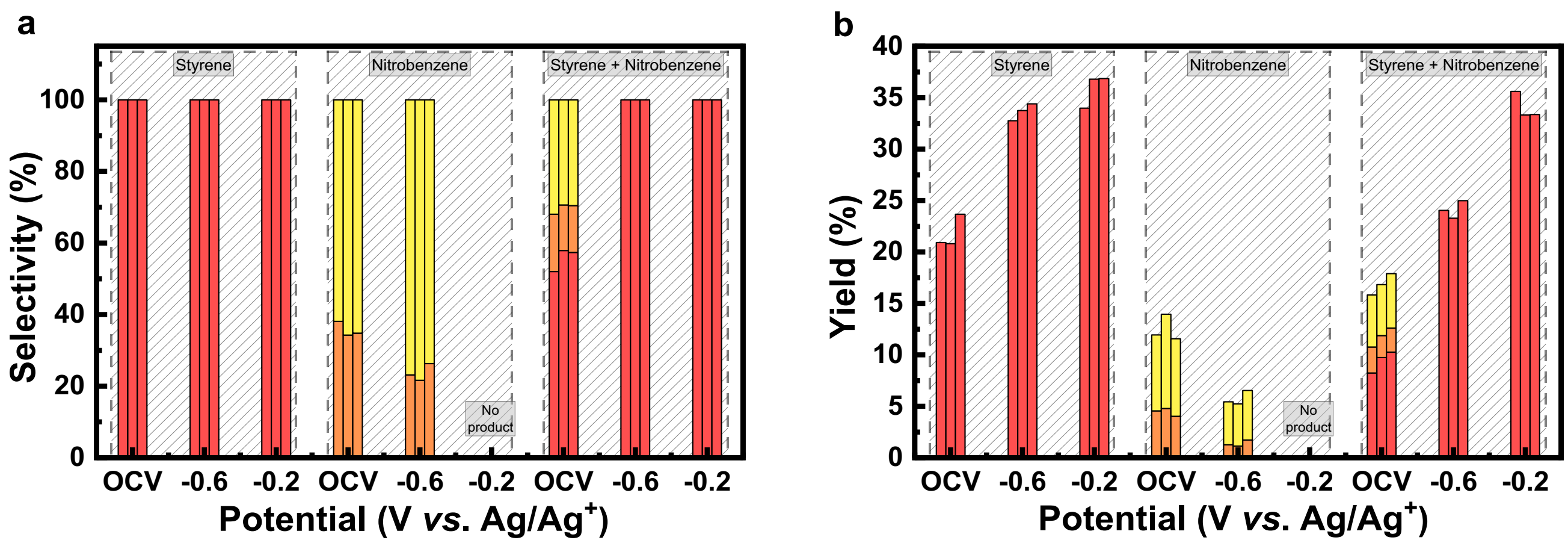

Figure S69. (a) Selectivity and (b) yield obtained in three independent replicate experiments for the catalytic hydrogenation of styrene, nitrobenzene, or a competitive hydrogenation of 1:1 mixture of styrene and nitrobenzene using Pt as catalyst, each shown as a function of applied potential. Colored bars correspond to the observed products: ethylbenzene (red), *N*-phenylhydroxylamine (orange), and aniline (yellow).

Table S17. Optimized ground state geometries and frontier molecular orbitals of styrene, nitrobenzene, and *trans*-cinnamaldehyde using Gaussian 09 program with the B3LYP functional and the 6-31G(d) basis set. Solvent effects were taken into account using the Polarizable Continuum Model (PCM) with acetonitrile as solvent. HOMO: highest occupied molecular orbital; LUMO: lowest unoccupied molecular orbital.

	Optimized structure	HOMO	LUMO
Styrene	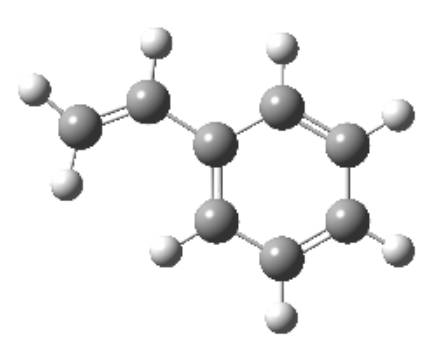	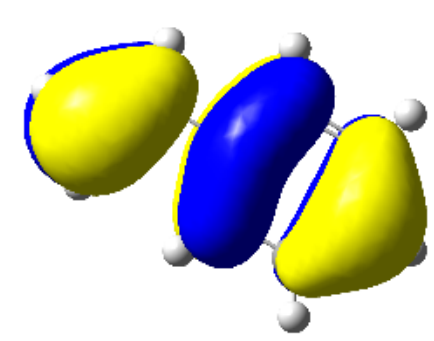	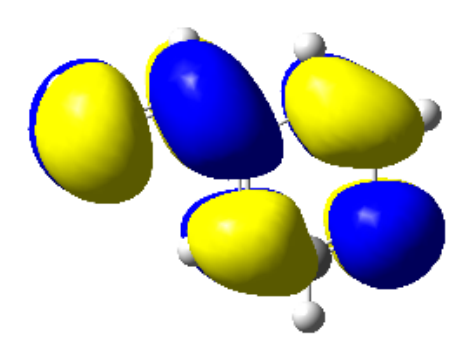
Nitrobenzene	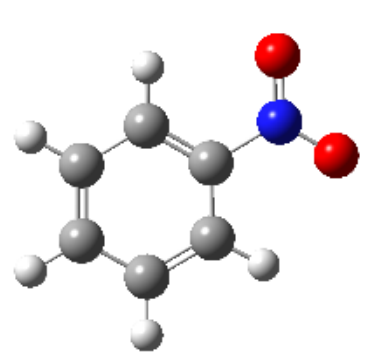	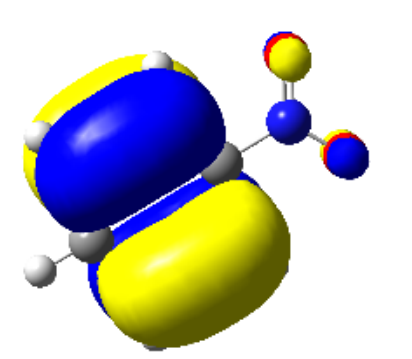	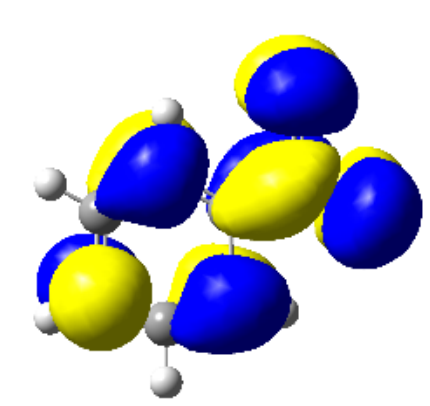
trans-Cinnamaldehyde	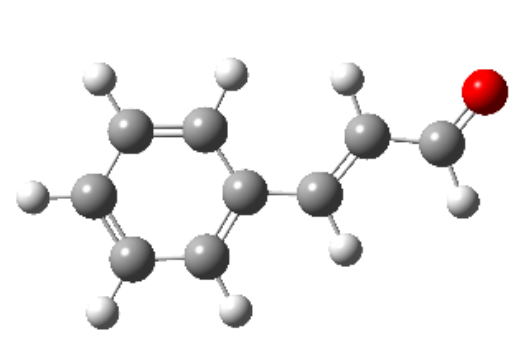	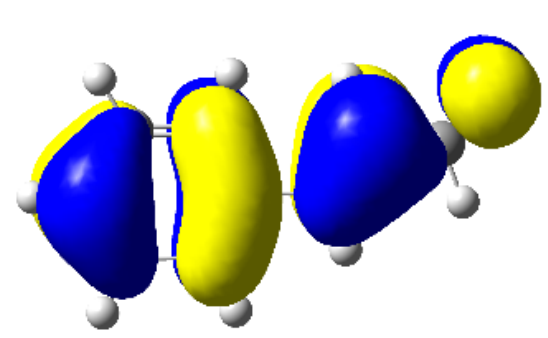	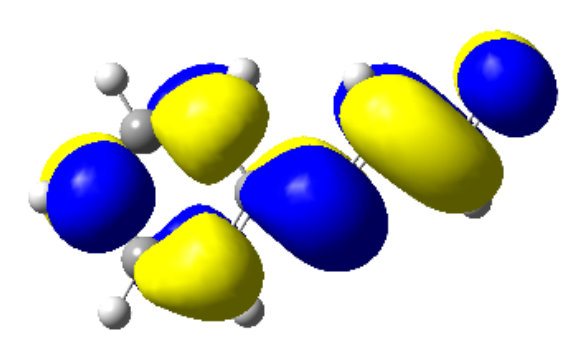

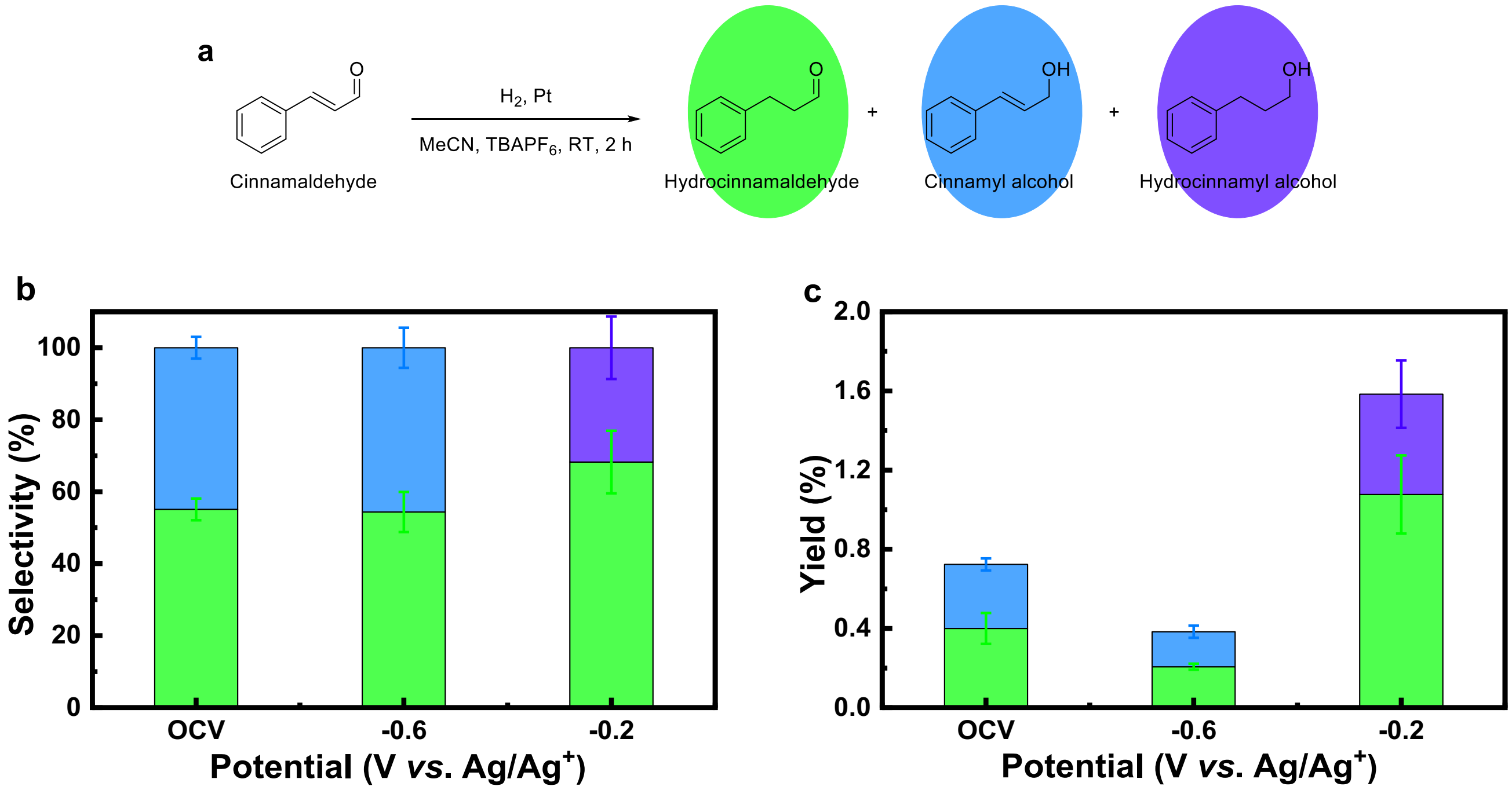

Figure S70. (a) Hydrogenation of *trans*-cinnamaldehyde using Pt as catalyst and associated (b) selectivity and (c) yield as a function of applied potential. Colored bars correspond to the observed products: hydrocinnamaldehyde (green), cinnamyl alcohol (blue), and hydrocinnamyl alcohol (purple). Mean and standard deviation shown for each set of conditions in (b) and (c) were derived from three independent replicate experiments.

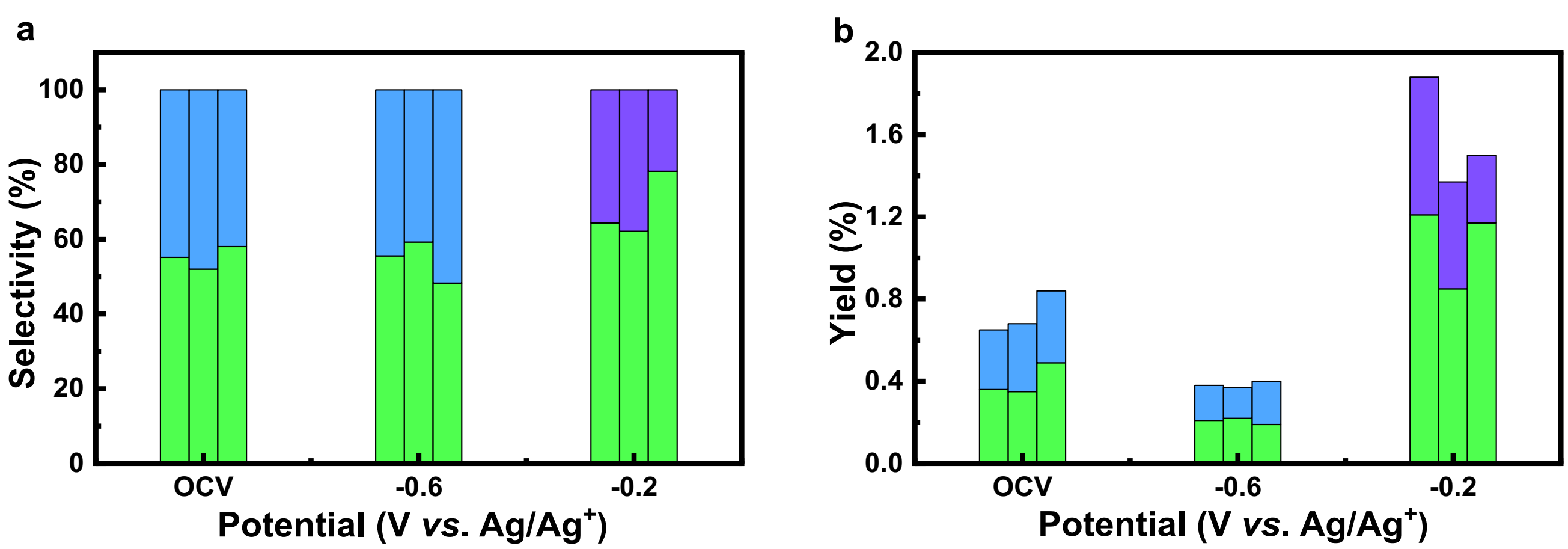

Figure S71. (a) Selectivity and (b) yield obtained in three independent replicate experiments of the catalytic hydrogenation of *trans*-cinnamaldehyde as a function of applied potential. Colored bars correspond to the observed products: hydrocinnamaldeyhde (green), cinnamyl alcohol (blue), and hydrocinnamyl alcohol (purple).

Table S18. Comparison of the catalytic hydrogenation performance of Pt at OCV and −0.2 V for different substrates. We note that experiments using 4-vinylbenzoic acid as substrate were done with a 13 mM solution of the substrate due to limitations in availability and solubility of the compound, while all other substrates have been tested under our standard conditions using a 100 mM solution of the substrate. For this reason, quantitative comparisons of the enhancement in yield obtained with different substrates should be done with care in case of 4-vinylbenzoic acid.

Substrate	Product	Reaction condition	Selectivity (%)	Yield (%)
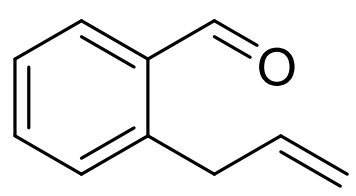	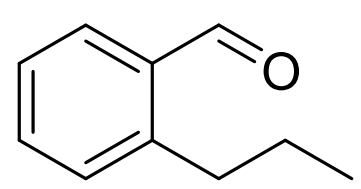	OCV	100	17.36
		−0.2 V	100	61.05
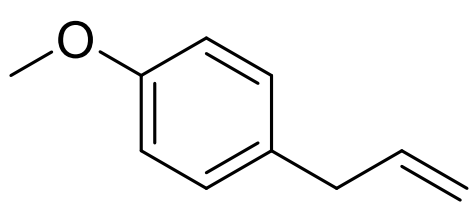	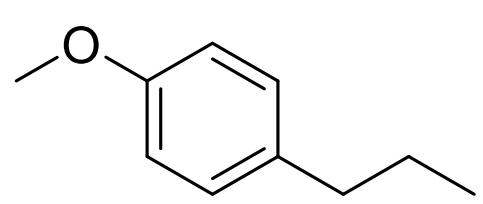	OCV	100	14.79
		−0.2 V	100	25.93
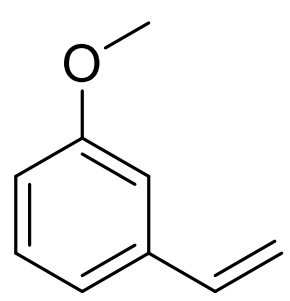	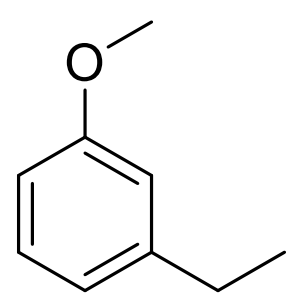	OCV	100	14.15
		−0.2 V	100	43.40
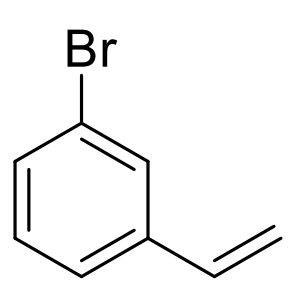	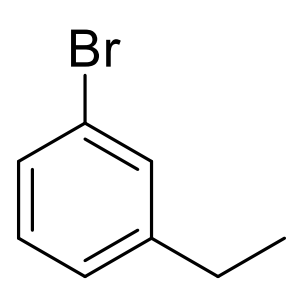	OCV	100	30.79
		−0.2 V	100	36.54
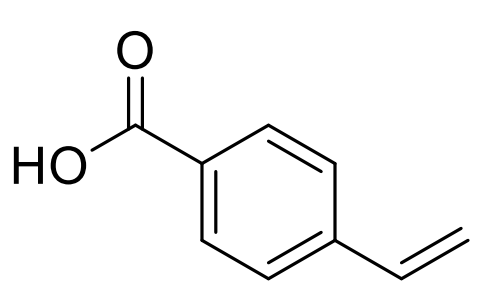	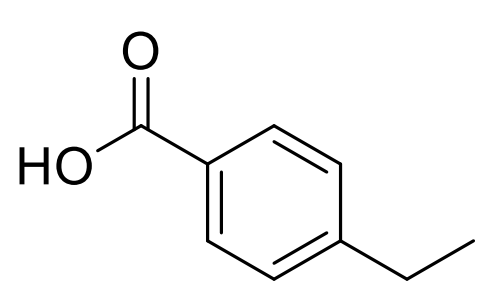	OCV	100	7.53
		−0.2 V	100	94.85
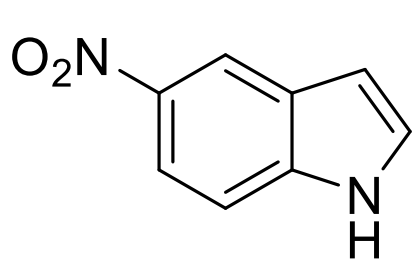	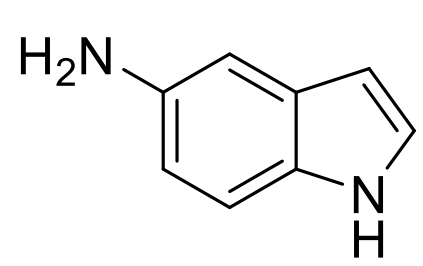	OCV	100	1.58
		−0.2 V	No product	

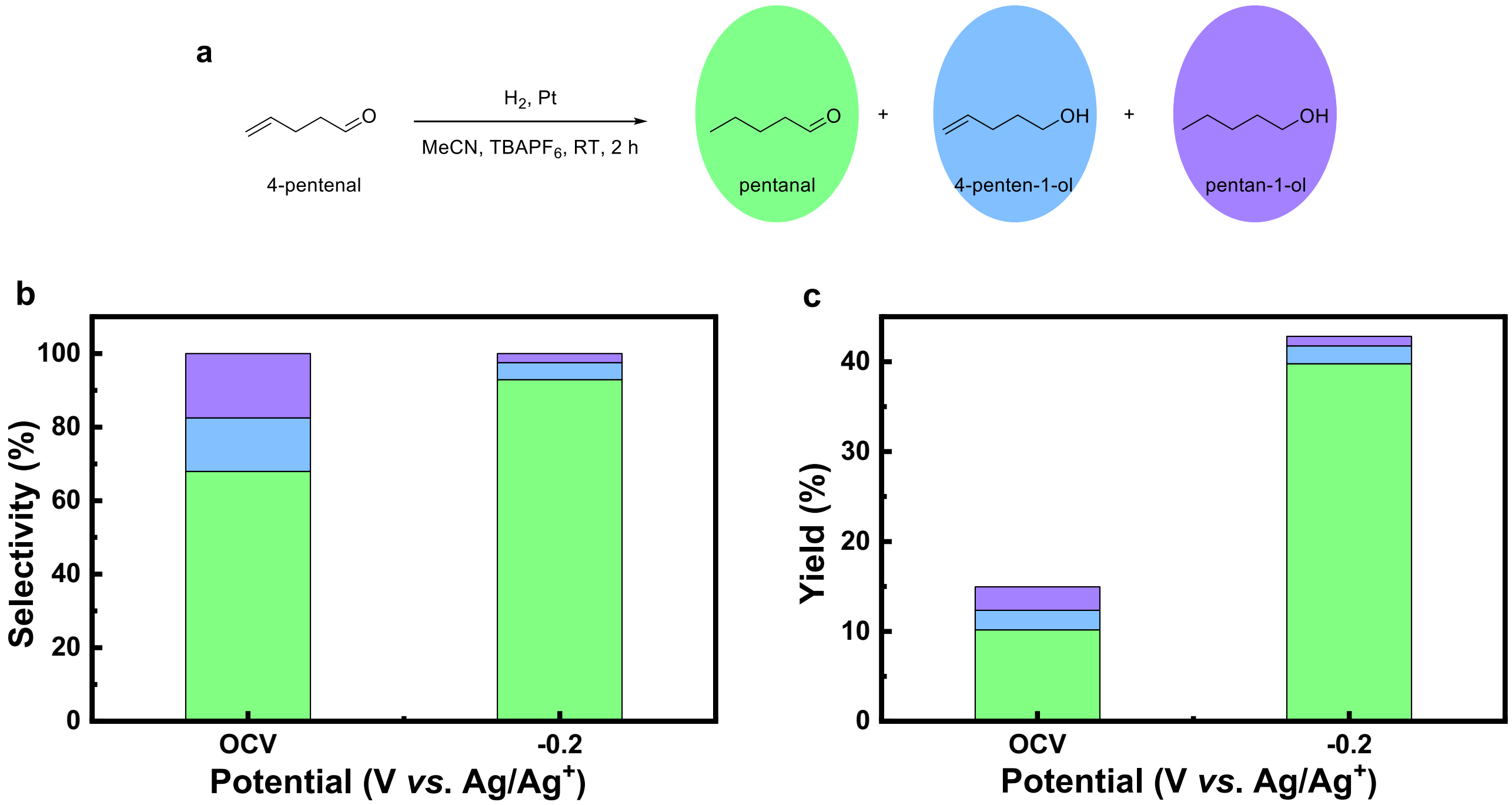

Figure S72. (a) Hydrogenation of 4-pentenal using Pt as catalyst and associated (b) selectivity and (c) yield as a function of applied potential. Colored bars correspond to the observed products: pentanal (green), 4-penten-1-ol (blue), and pentan-1-ol (purple).

10.2 Testing catalytic hydrogenation of 3-NS as a function of applied potential using other catalysts

To test whether our observations apply more broadly to hydrogenation reactions using other catalysts, we probed the effect of an applied potential on the catalytic hydrogenation of 3-NS using Pd as catalyst under otherwise similar reaction conditions as previously used for Pt. Catalytic hydrogenation was tested at OCV (ca. −1 V), −0.6 V, and −0.2 V. Pd electrode was prepared by a similar method and setup as used by Pt electrode using a deposition solution containing 10 mM $PdCl_2$ and 250 mM $HClO_4$ in MQ water.

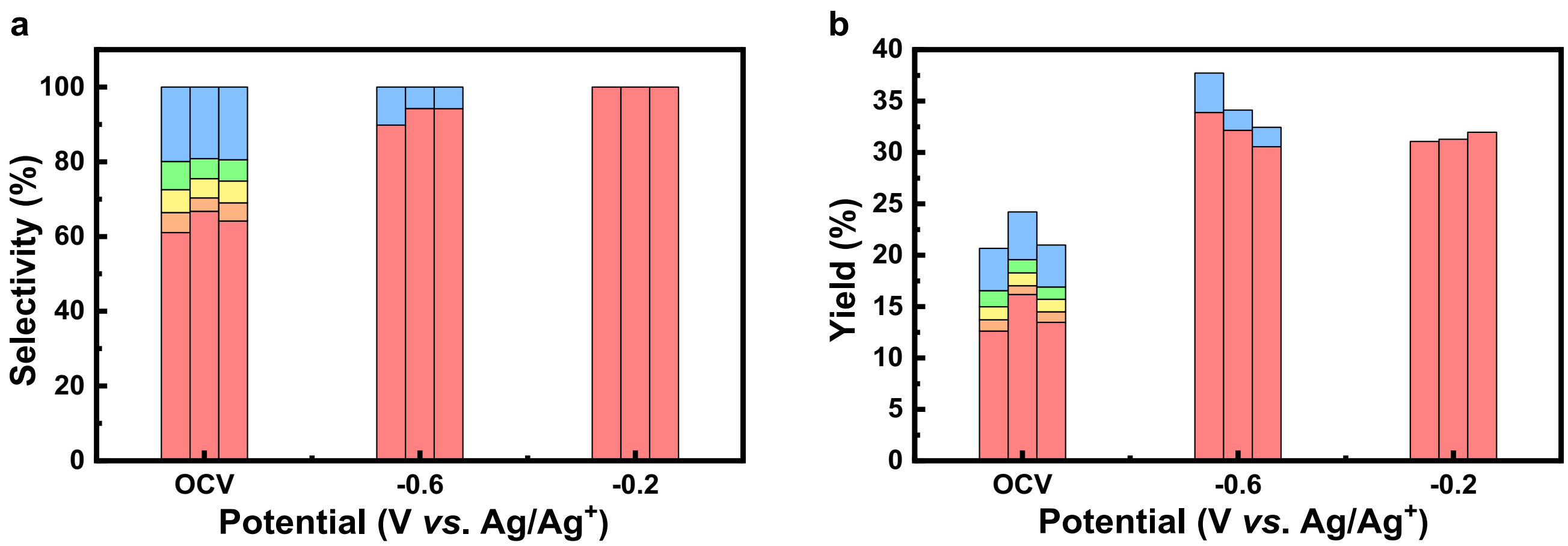

Figure S73. (a) Selectivity and (b) yield obtained in three independent replicate experiments of the catalytic 3-NS hydrogenation using Pd as catalyst as a function of applied potential. Colored bars correspond to the observed products: 3-ENB (red), 3-VAOH (orange), 3-VA (yellow), 3-EAOH (green), and 3-EA (blue).

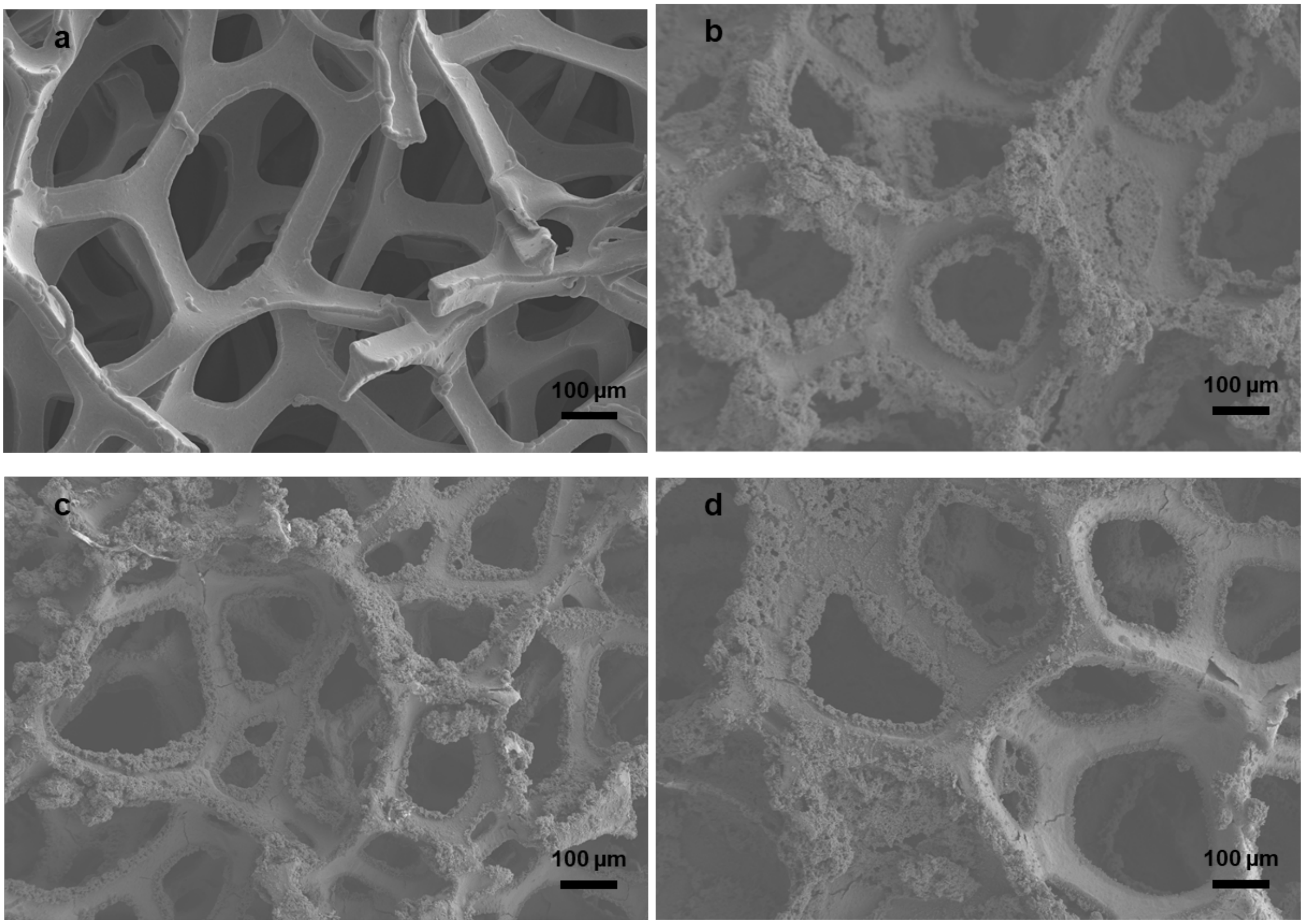

Figure S74. SEM images of (a) the bare Ni foam (image reproduced from Figure S22), (b) the Pd-coated Ni foam before catalysis, the Pd-coated Ni foam after hydrogenation at OCV (c) or −0.2 V (d). The SEM images of the Pd catalyst look similar before and after catalysis, which would be consistent with the idea that the applied potential of −0.2 V does not change the catalyst permanently.

Table S19. Elemental composition obtained by EDX analysis of the bare Ni foam, the Pd-coated Ni foam before catalysis, and the Pd-coated Ni foam after 3-NS hydrogenation at OCV or −0.2 V. The data shown in this table are obtained from the average of the elemental composition measured at multiple individual spots on each sample. EDX data for the bare Ni foam is reproduced from Table S6.

	C (%)	O (%)	Ni (%)	Pd (%)
Ni foam	48.7	8.6	42.7	-
Pd coated Ni foam	40.7	2.5	0.7	56.1
after OCV reaction	45.2	7.4	7.3	40.2
after −0.2 V reaction	42.1	3.9	3.5	50.5

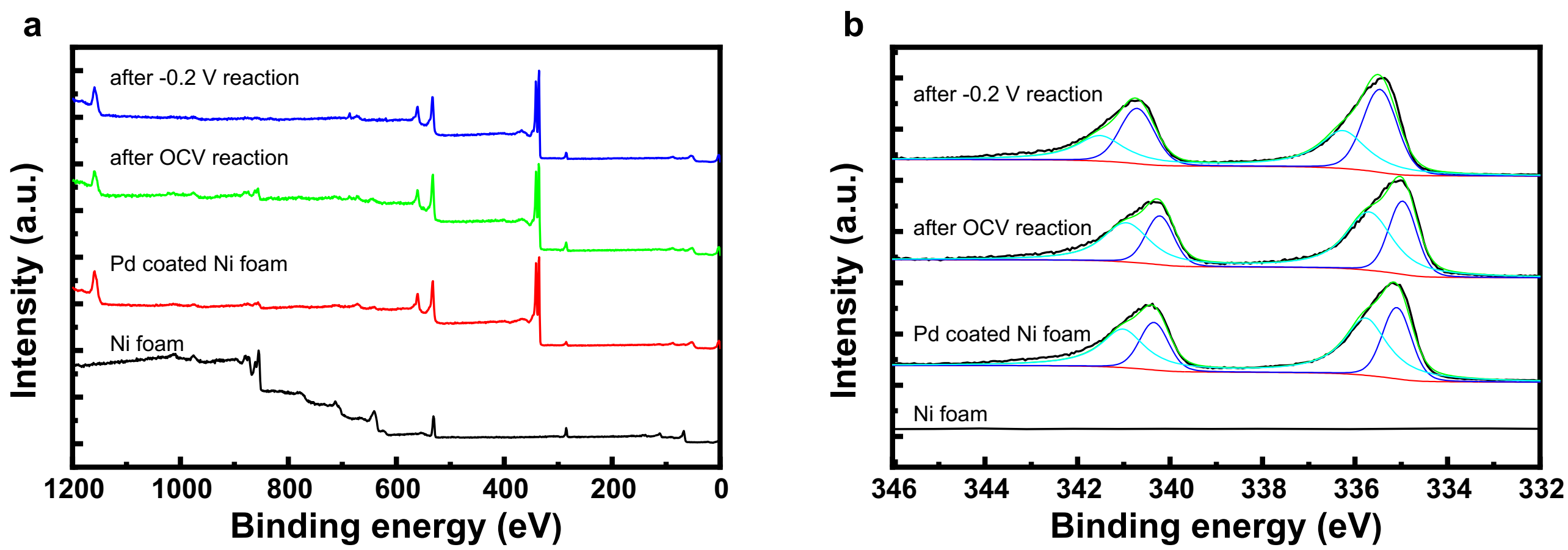

Figure S75. XPS (a) survey spectra, and (b) Pd 3d spectra measured for the bare Ni foam, the Pd-coated Ni foam before catalysis, and the Pd-coated Ni foam after 3-NS hydrogenation at OCV or −0.2 V (from bottom to top). The XPS survey spectrum of the bare Ni foam is reproduced from Figure S23 and shown here for comparison. The Pd-coated Ni foam before and after catalysis at OCV or −0.2 V showed the Pd $3d_{5/2}$ and Pd $3d_{3/2}$ signals at ~335 and ~340 eV, as expected for Pd metal.[29] The Pd 3d peaks obtained for Pd films after catalysis at OCV or at −0.2 V are only slightly shifted to higher binding energies compared to the as-synthesized catalyst suggesting that the catalyst is not substantially changed under reaction conditions.

To test the effect of an applied electrochemical potential on the hydrogenation catalysis using non-noble metal catalysts, we probed the catalytic hydrogenation of 3-NS at OCV and positive applied potentials using cobalt phosphide (CoP), cobalt sulfide (CoS), Ni foam, and Cu foam as catalysts under otherwise similar reaction conditions as previously used for Pt. Prior work suggests that these materials may be active hydrogenation catalysts.[60-63]

CoP: CoP was deposited on Ni foam (2 × 1 cm^2) from a solution of 50 mM $CoSO_4 \cdot 7H_2O$, 500 mM $NaH_2PO_2 \cdot H_2O$, and 100 mM CH_3COONa in MQ water by applying cyclic voltammetry in the potential range from −1.2 to 0.5 V *vs*. Ag/AgCl for 15 cycles at 10 mV s^{-1} at 100 rpm stirring. Catalytic hydrogenation of 3-NS with H_2 was tested at OCV (ca. −0.5 V), and 0 V for 2 h reaction time each. Analysis of the reaction mixture in the working chamber by ^{1}H-NMR did not show any product formation at either OCV or positive applied potential suggesting that CoP is not catalytically active under the conditions studied herein.

CoS: CoS was deposited on Ni foam (2 × 1 cm^2) from a solution of 10 mM $CoCl_2 \cdot 6H_2O$ and 1 M thiourea in MQ water by applying cyclic voltammetry in the potential range from −1.2 to 0.2 V *vs*. Ag/AgCl for 10 cycles at 10 mV s^{-1} at 100 rpm stirring. Catalytic hydrogenation of 3-NS with H_2 was tested at OCV (ca. −0.4 V), and 0 V for 2 h reaction time each. Analysis of the reaction mixture in the working chamber by ^{1}H NMR did not show any product formation at either OCV or positive applied potential suggesting that CoS is not catalytically active under the conditions studied herein.

Ni foam: Ni foam (2 × 1 cm^2) was cleaned by an ultrasonic bath using 1 M H_2SO_4, acetone and MQ water for five minutes each. Catalytic hydrogenation of 3-NS with H_2 was tested at OCV (ca. −0.6 V), and 0.2 V for 2 h reaction time each. Analysis of the reaction mixture in the working chamber by ^{1}H-NMR did not show any product formation at either OCV or positive applied potential suggesting that Ni foam is not catalytically active under the conditions studied herein (see Figure S18).

Cu foam: Cu foam (2×1 cm^2) was cleaned by an ultrasonic bath using 1 M H_2SO_4, acetone and MQ water for five minutes each. Catalytic hydrogenation of 3-NS with H_2 was tested at OCV (ca. −0.6 V), and −0.4 V for 2 h reaction time each. Analysis of the reaction mixture in the working chamber by ^{1}H-NMR did not show any product formation at either OCV or positive applied potential suggesting that Cu foam is not catalytically active under the conditions studied herein.

11. Kinetic experiments

11.1 Variation of the hydrogen flow rate

Experiments with varied hydrogen flow rate were performed using a similar procedure as the standard 3-NS hydrogenation experiments at 5 sccm hydrogen flow. Varied flow rate experiments were carried out with 2.5, 5, 7.5, or 10 sccm, separately. For each flow rate, we did three independent replicate experiments.

Table S20. Average electric charge from three independent replicate experiments with varied hydrogen flow rate at −0.2 V, and ^{MeCN}pH values measured in the working chamber solution after catalysis at −0.2 V. Absolute ^{MeCN}pH values should be cautiously interpreted due to the uncertainty associated with such measurements in organic solvents and with respect to a MeCN-based pH scale, but general trends observed should be more accurate. Detailed comments about pH measurement can be found in SI section S2.

	2.5 sccm	5 sccm	7.5 sccm	10 sccm
Average electric charge (C)	7.52 ± 0.58	7.92 ± 0.61	8.61 ± 0.09	9.21 ± 0.62
^{MeCN}pH value	−0.41	−0.51	−0.54	−0.68

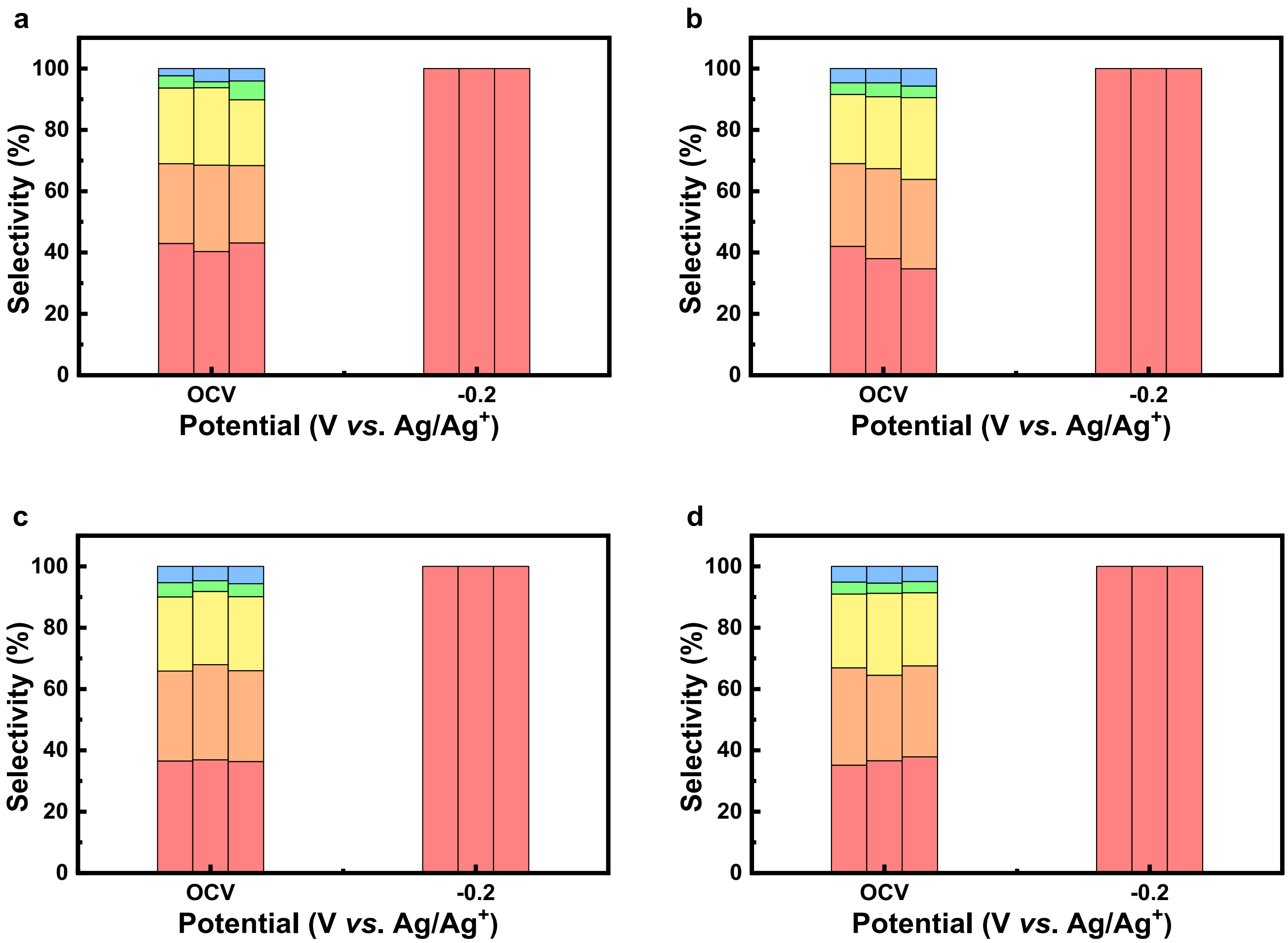

Figure S76. Selectivity obtained in three independent replicate experiments of the 3-NS hydrogenation with varied hydrogen flow rate of (a) 2.5 sccm, (b) 5 sccm, (c) 7.5 sccm, and (d) 10 sccm.

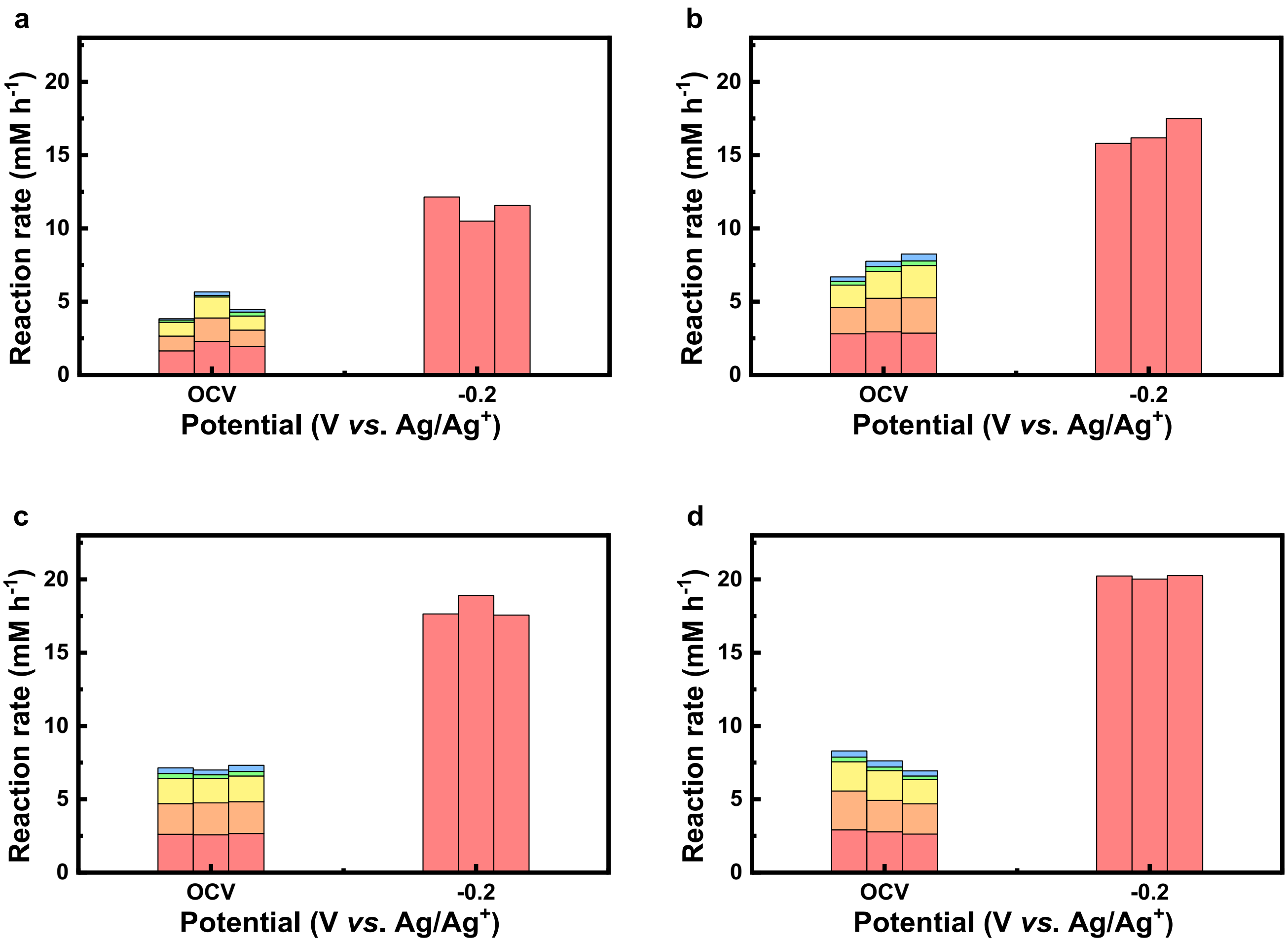

Figure S77. Reaction rate obtained in three independent replicate experiments of the 3-NS hydrogenation with varied hydrogen flow rate of (a) 2.5 sccm, (b) 5 sccm, (c) 7.5 sccm, and (d) 10 sccm.

11.2 Variation of the 3-NS concentration

Experiments with varied 3-NS concentration were performed using a similar procedure as the standard 3-NS hydrogenation experiments using a solution of 100 mM 3-NS. Varied 3-NS concentration experiments were carried out with 25, 50, 100, or 200 mM, separately. For each concentration used, we did three independent replicate experiments.

Table S21. Average electric charge from three independent replicate experiments with varied 3-NS concentration at −0.2 V, and ^{MeCN}pH values measured in the working chamber solution after catalysis at −0.2 V. Absolute ^{MeCN}pH values should be cautiously interpreted due to the uncertainty associated with such measurements in organic solvents and with respect to a MeCN-based pH scale, but general trends observed should be more accurate. Detailed comments about pH measurement can be found in SI section S2.

	25 mM	50 mM	100 mM	200 mM
Average electric charge (C)	9.14 ± 1.43	8.69 ± 1.26	7.92 ± 0.61	6.87 ± 0.69
^{MeCN}pH value	−0.67	−0.55	−0.51	−0.35

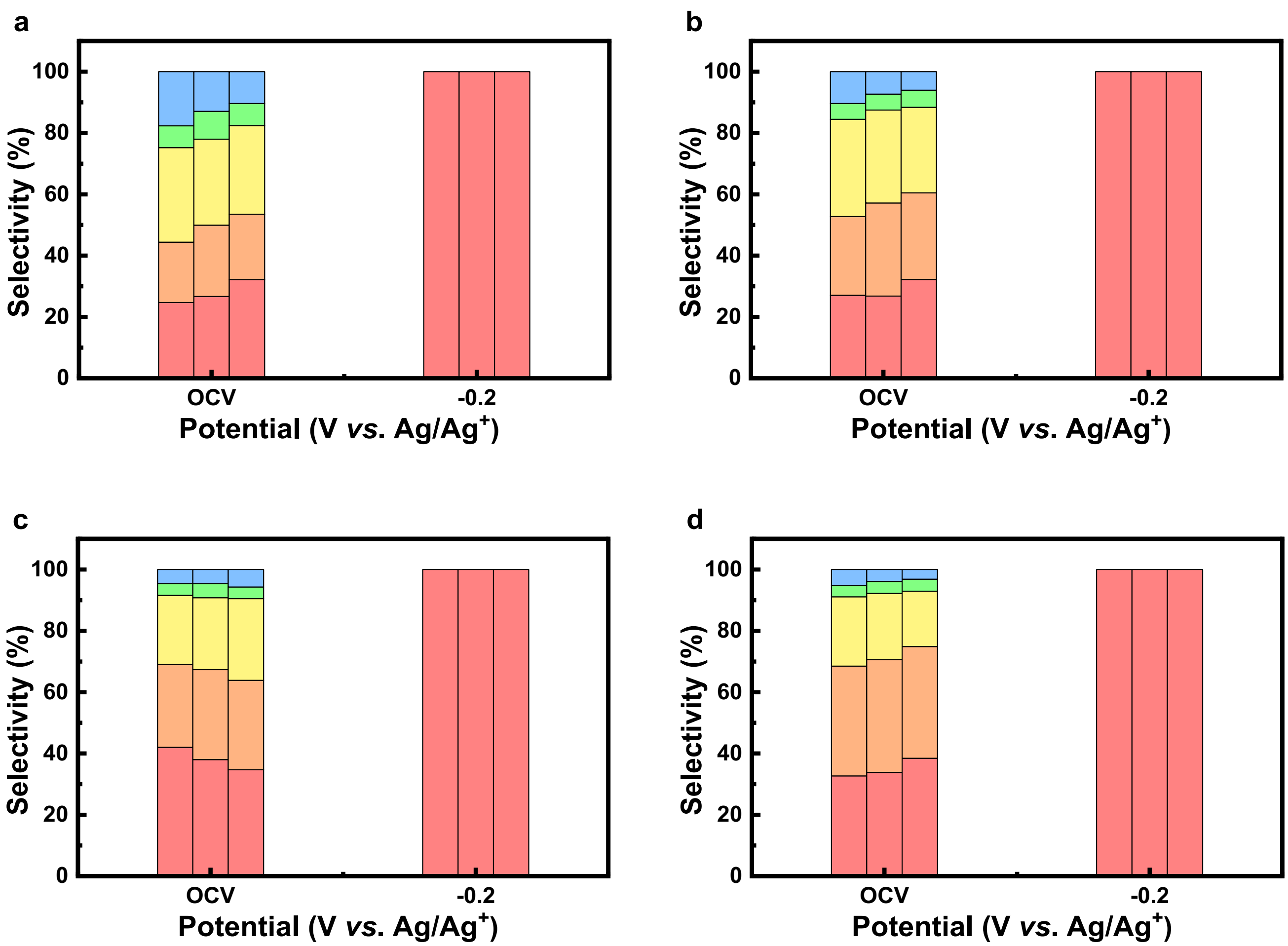

Figure S78. Selectivity obtained in three independent replicate experiments of the 3-NS hydrogenation with varied 3-NS concentration of (a) 25 mM, (b) 50 mM, (c) 100 mM, and (d) 200 mM.

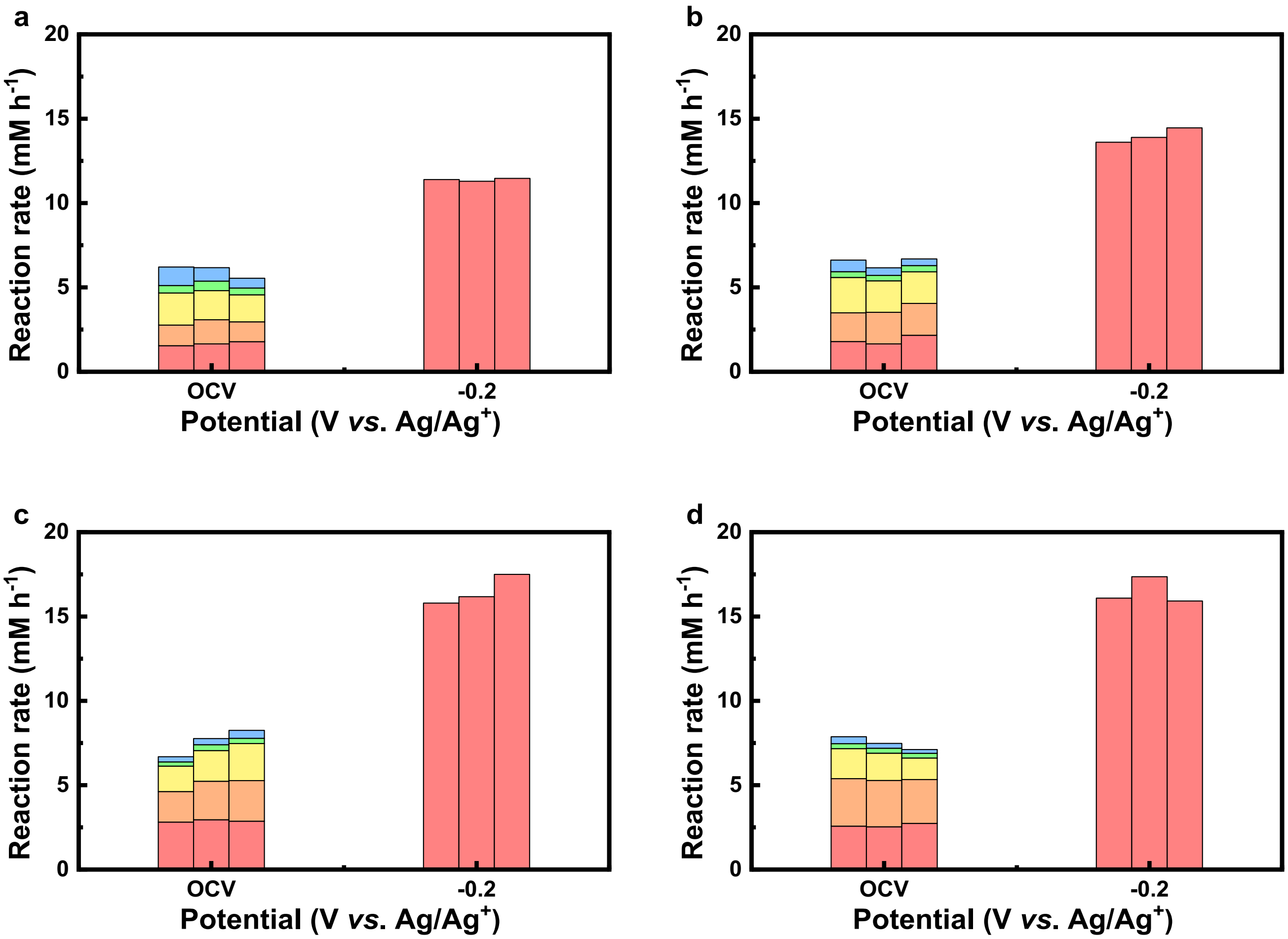

Figure S79. Reaction rate obtained in three independent replicate experiments of the 3-NS hydrogenation with varied 3-NS concentration of (a) 25 mM, (b) 50 mM, (c) 100 mM, and (d) 200 mM.

11.3 Variation of the reaction temperature

Experiments with varied reaction temperature were performed using a similar procedure as the standard 3-NS hydrogenation experiments at room temperature (25 ± 1 °C). Experiments at varied temperature were carried out at 5 °C, 15 °C, 25 °C (room temperature), 30 °C, or 35 °C, separately. For each temperature, we did three independent replicate experiments.

Table S22. Average electric charge from three independent replicate experiments with varied reaction temperature at −0.2 V, and ^{MeCN}pH values measured in the working chamber solution after catalysis at −0.2 V. Absolute ^{MeCN}pH values should be cautiously interpreted due to the uncertainty associated with such measurements in organic solvents and with respect to a MeCN-based pH scale, but general trends observed should be more accurate. Detailed comments about pH measurement can be found in SI section S2.

	5 °C	15 °C	25 °C	30 °C	35 °C
Average electric charge (C)	4.36 ± 1.20	5.29 ± 1.73	7.91 ± 0.61	10.31 ± 3.98	8.10 ± 2.43
^{MeCN}pH value	0.48	−0.05	−0.51	−0.75	−0.52

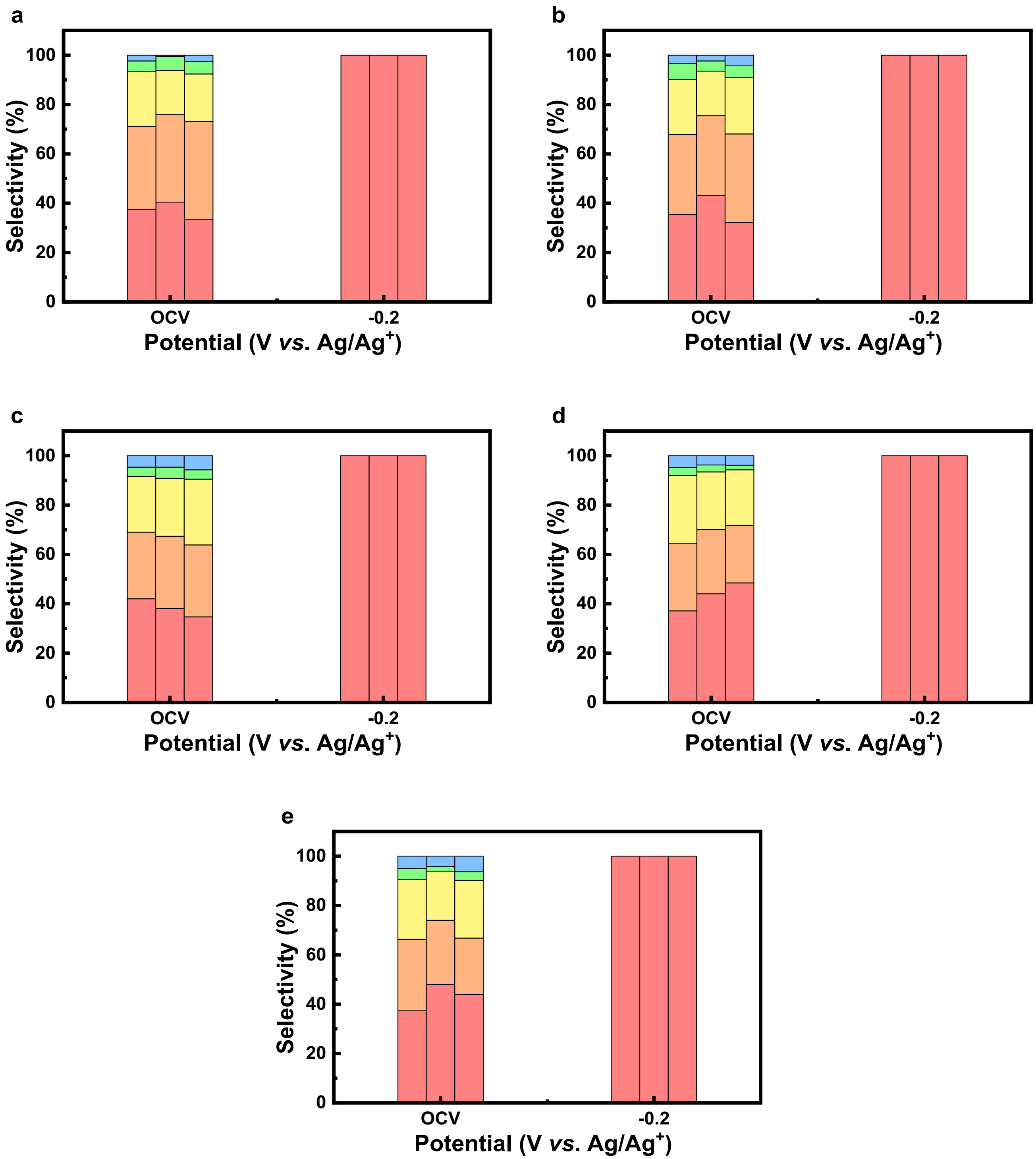

Figure S80. Selectivity obtained in three independent replicate experiments of the 3-NS hydrogenation with varied reaction temperature of (a) 5 °C, (b) 15 °C, (c) 25 °C, (d) 30 °C, and (e) 35 °C.

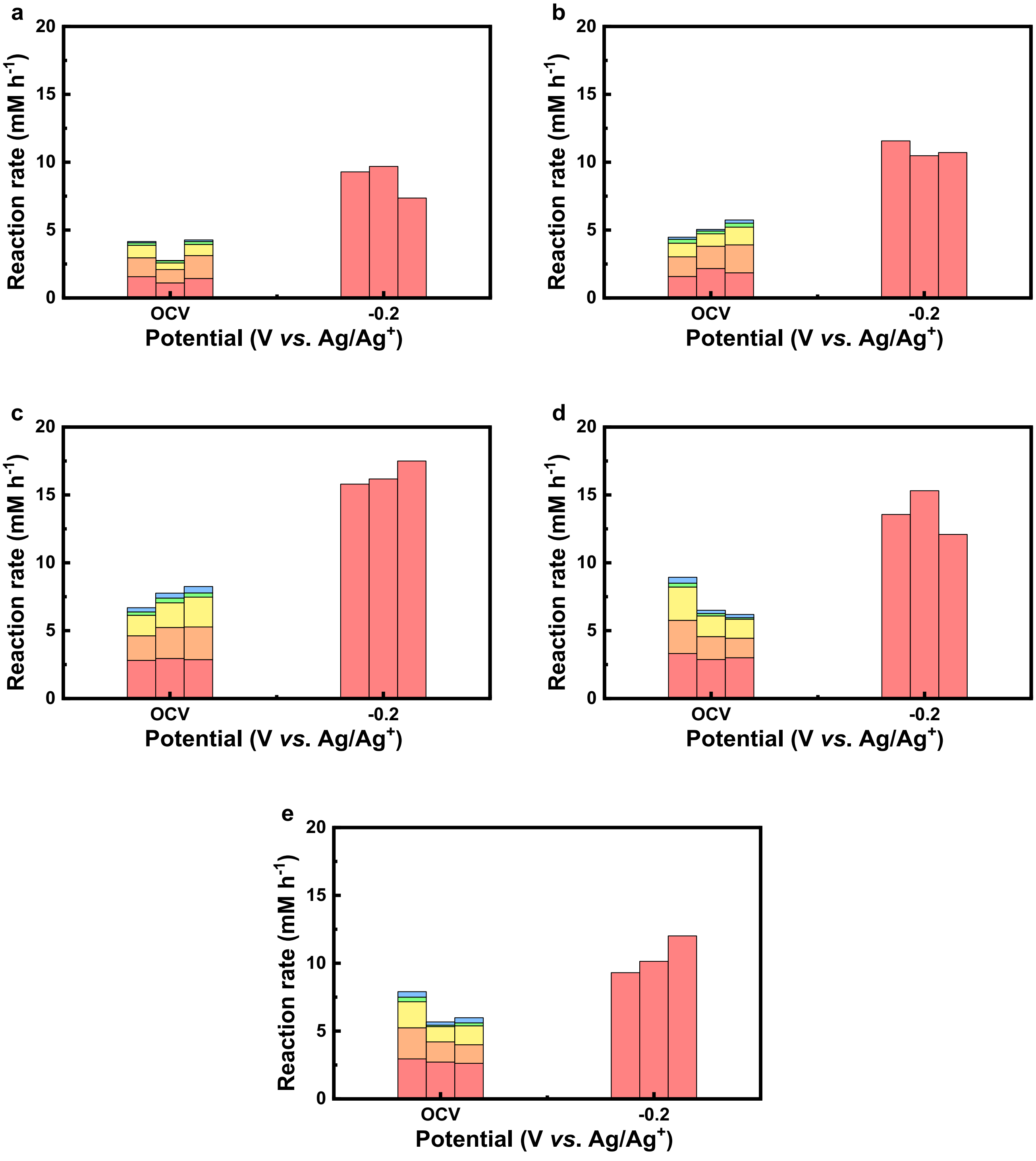

Figure S81. Reaction rate obtained in three independent replicate experiments of the 3-NS hydrogenation with varied reaction temperature of (a) 5 °C, (b) 15 °C, (c) 25 °C, (d) 30 °C, and (e) 35 °C.

11.4 Estimation of activation energy

The activation energy can be determined using the Arrhenius equation:

$$k = Ae^{-E_a/RT} \quad \text{eq S3}$$

where k is the rate constant, A is the pre-exponential factor, E_a is the activation energy, R is the universal gas constant (8.314 J mol^{-1} K^{-1}), and T is the absolute temperature in Kelvin.

From eq S3, we obtain

$$\ln k = \ln A + \frac{-E_A}{R}\frac{1}{T} \quad \text{eq S4}$$

The activation energy can be obtained by plotting $\ln k$ versus $\frac{1}{T}$ from the value of the slope that is equal to $\frac{-E_A}{R}$.

From a generalized rate law:

$$v = k[A]^x[B]^y \quad \text{eq S5}$$

where k is the rate constant, $[A]$ and $[B]$ are the concentrations of species A and B, x and y are the reaction order for A and B, respectively. From eq S5, we obtain

$$\ln v = \ln k + x\ln[A] + y\ln[B] \quad \text{eq S6}$$

Since the H_2 concentration was controlled by flow rate rather than partial pressure or concentration, we are unable to determine reactant concentrations and reaction order accurately with the data in hand. Still, some qualitative insight may still be obtained from our data. While [A] and [B] change during a reaction, the rate orders x and y are constant for a specific reaction. To determine a rate constant k, usually initial concentrations of A and B ($[A_{initial}]$ and $[B_{initial}]$) are used, which to a rough, first approximation may be considered constant during an initial phase of catalysis. Hence, in this first reaction phase one could perhaps approximate $\ln k$ as:

$$\ln k = \ln v - constant \rightarrow \ln v - constant = \ln A + \frac{-E_A}{R}\frac{1}{T} \rightarrow \ln v = \frac{-E_A}{R}\frac{1}{T} + \ln A + constant \quad \text{eq S7}$$

Therefore, plotting $\ln v$ versus $\frac{1}{T}$ instead of $\ln k$ versus $\frac{1}{T}$ could provide an approximation to $\frac{-E_A}{R}$. We note that the thereby determined absolute values for the apparent activation energies E_{app} (approximating E_A in the equations above) should be analyzed with care in light of the approximations made to derive them.

11.5 Variation of the $TBAPF_6$ concentration

Experiments with varied $TBAPF_6$ concentration were performed using a similar procedure as the standard 3-NS hydrogenation experiments using a solution of 100 mM $TBAPF_6$. Varied $TBAPF_6$ concentration experiments were carried out with 50, 100, or 150 mM, separately. For each concentration used, we did three independent replicate experiments.

Table S23. Average electric charge from three independent replicate experiments with varied $TBAPF_6$ concentration at −0.2 V, and ^{MeCN}pH values measured in the working chamber solution after catalysis at −0.2 V. Absolute ^{MeCN}pH values should be cautiously interpreted due to the uncertainty associated with such measurements in organic solvents and with respect to a MeCN-based pH scale, but general trends observed should be more accurate. Detailed comments about pH measurement can be found in SI section S2.

	50 mM	100 mM	150 mM
Average electric charge (C)	7.97 ± 0.56	7.76 ± 0.13	8.72 ± 0.94
^{MeCN}pH value	−0.52	−0.50	−0.55

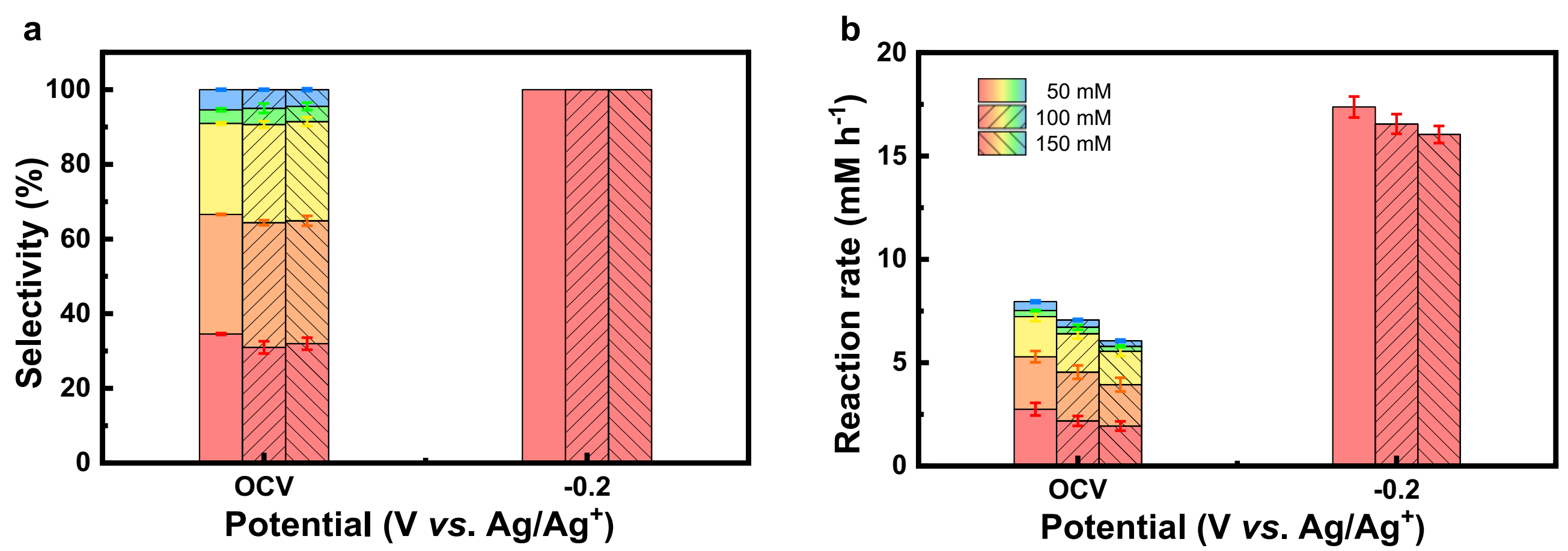

Figure S82. (a) Selectivity and (b) reaction rate of the 3-NS hydrogenation with variation of the $TBAPF_6$ concentration. Mean and standard deviation shown for each set of conditions were derived from three independent replicate experiments.

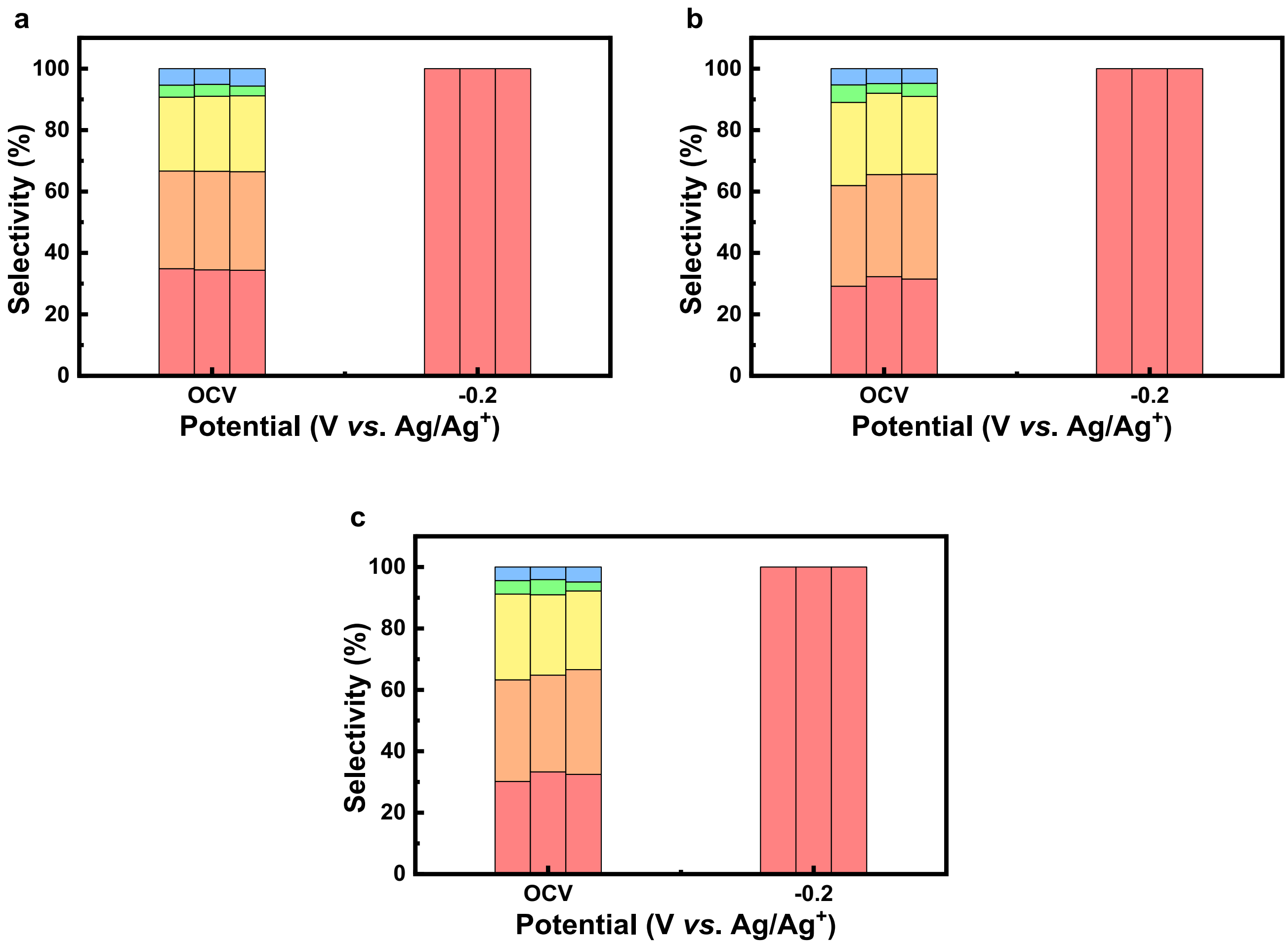

Figure S83. Selectivity obtained in three independent replicate experiments of the 3-NS hydrogenation with varied $TBAPF_6$ concentration of (a) 50 mM, (b) 100 mM, and (c) 150 mM.

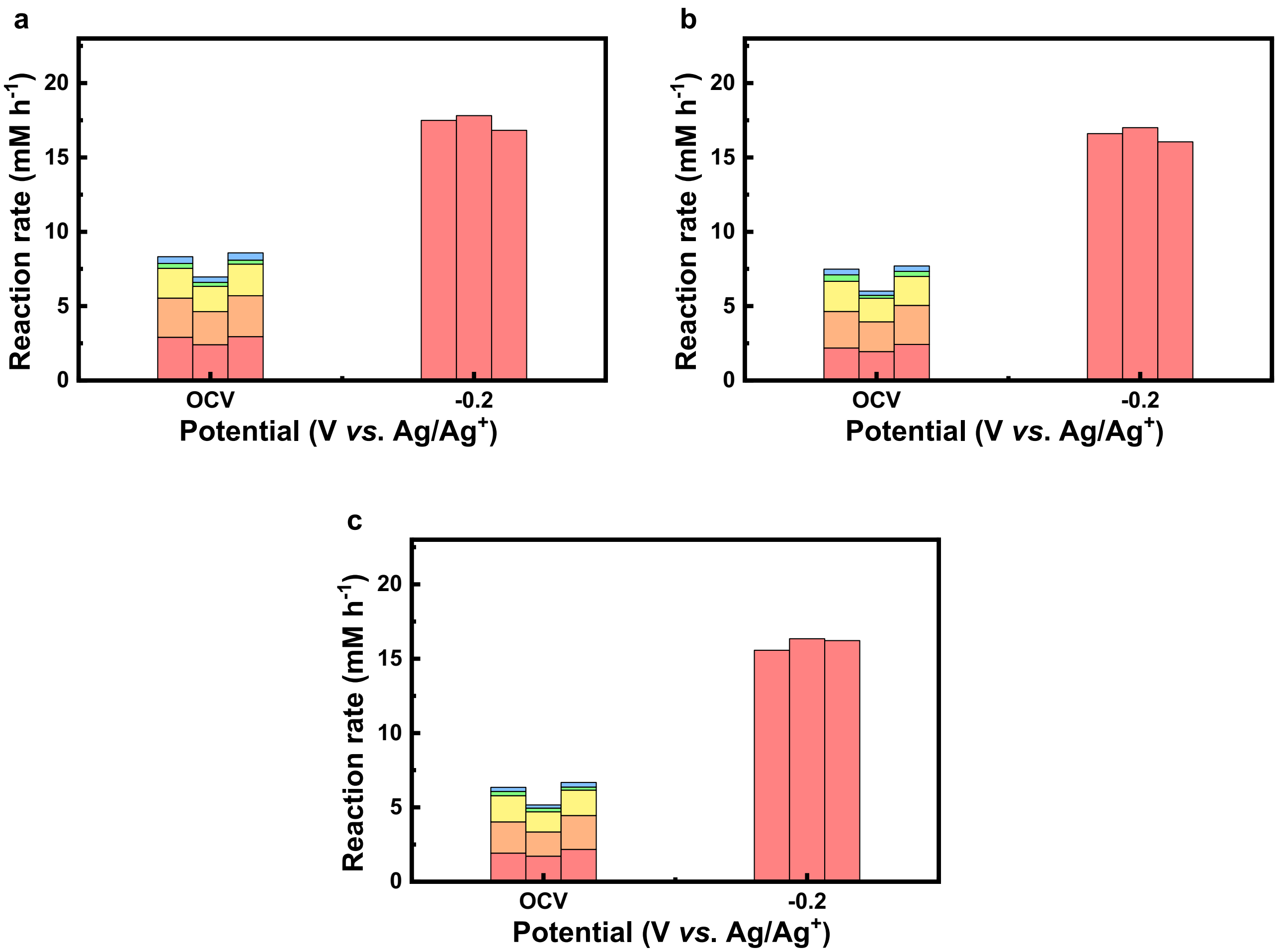

Figure S84. Reaction rate obtained in three independent replicate experiments of the 3-NS hydrogenation with varied $TBAPF_6$ concentration of (a) 50 mM, (b) 100 mM, and (c) 150 mM.

References

[1] A. Pélisson-Schecker, H. J. Hug, J. Patscheider, "Charge referencing issues in XPS of insulators as evidenced in the case of Al-Si-N thin films" *Surface and Interface Analysis* **44**, (2011): 29-36.

[2] M. J. Frisch, G. W. Trucks, H. B. Schlegel, G. E. Scuseria, M. A. Robb, J. R. Cheeseman, G. Scalmani, V. Barone, B. Mennucci, G. A. Petersson, H. Nakatsuji, M. Caricato, X. Li, H. P. Hratchian, A. F. Izmaylov, J. Bloino, G. Zheng, J. L. Sonnenberg, M. Hada, M. Ehara, K. Toyota, R. Fukuda, J. Hasegawa, M. Ishida, T. Nakajima, Y. Honda, O. Kitao, H. Nakai, T. Vreven, J. J. A. Montgomery, J. E. Peralta, F. Ogliaro, M. Bearpark, J. J. Heyd, E. Brothers, K. N. Kudin, V. N. Staroverov, T. Keith, R. Kobayashi, J. Normand, K. Raghavachari, A. Rendell, J. C. Burant, S. S. Iyengar, J. Tomasi, M. Cossi, N. Rega, J. M. Millam, M. Klene, J. E. Knox, J. B. Cross, V. Bakken, C. Adamo, J. Jaramillo, R. Gomperts, R. E. Stratmann, O. Yazyev, A. J. Austin, R. Cammi, C. Pomelli, J. W. Ochterski, R. L. Martin, K. Morokuma, V. G. Zakrzewski, G. A. Voth, P. Salvador, J. J. Dannenberg, S. Dapprich, A. D. Daniels, O. Farkas, J. B. Foresman, J. V. Ortiz, J. Cioslowski, D. J. Fox, "Gaussian 09, Revision C.01" *Gaussian, Inc., Wallingford CT* (2010).

[3] A. D. Becke, "Density-functional thermochemistry. III. The role of exact exchange" *The Journal of Chemical Physics* **98**, (1993): 5648-5652.

[4] C. Lee, W. Yang, R. G. Parr, "Development of the Colle-Salvetti correlation-energy formula into a functional of the electron density" *Physical Review B* **37**, (1988): 785-789.

[5] B. Miehlich, A. Savin, H. Stoll, H. Preuss, "Results obtained with the correlation energy density functionals of becke and Lee, Yang and Parr" *Chemical Physics Letters* **157**, (1989): 200-206.

[6] Y. Jiao, L. Mao, S. Liu, T. Tan, D. Wang, D. Cao, B. Mi, Z. Gao, W. Huang, "Effects of meta or para connected organic dyes for dye-sensitized solar cell" *Dyes and Pigments* **158**, (2018): 165-174.

[7] Y. Jiao, S. Liu, Z. Shen, L. Mao, Y. Ding, D. Ren, F. T. Eickemeyer, L. Pfeifer, D. Cao, W. Xu, J. Song, B. Mi, Z. Gao, S. M. Zakeeruddin, W. Huang, M. Grätzel, "Carbazol-phenyl-phenothiazine-based sensitizers for dye-sensitized solar cells" *Journal of Materials Chemistry A* **9**, (2021): 26311-26322.

[8] *White paper pH of non-aqueous samples measurement in organic solvents*, Mettler-Toledo GmbH, (2020).

[9] L. G. Gagliardi, C. B. Castells, C. Ràfols, M. Rosés, E. Bosch, "δ conversion parameter between pH scales (${}^{S}_{W}$pH and ${}^{S}_{S}$pH) in acetonitrile/water mixtures at various compositions and temperatures" *Analytical Chemistry* **79**, (2007): 3180-3187.

[10] D. Himmel, S. K. Goll, I. Leito, I. Krossing, "A unified pH scale for all phases" *Angewandte Chemie International Edition* **49**, (2010): 6885-6888.

[11] L. Deleebeeck, A. Snedden, D. Nagy, Z. Szilágyi Nagyné, M. Roziková, M. Vičarová, A. Heering, F. Bastkowski, I. Leito, R. Quendera, V. Cabral, D. Stoica, "Unified pH measurements of ethanol, methanol, and acetonitrile, and their mixtures with water" *Sensors* **21**, (2021): 3935.

[12] S. Espinosa, E. Bosch, M. Rosés, "Acid–base constants of neutral bases in acetonitrile–water mixtures" *Analytica Chimica Acta* **454**, (2002): 157-166.

[13] F. Haber, "Gradual electrolytic reduction of nitrobenzene with limited cathode potential" *Zeitschrift für Elektrochemie und Angewandte Physikalische Chemie* **22**, (1898): 506-514.

[14] P. Serna, A. Corma, "Transforming nano metal nonselective particulates into chemoselective catalysts for hydrogenation of substituted nitrobenzenes" *ACS Catalysis* **5**, (2015): 7114-7121.

[15] C. Berguerand, A. Yarulin, F. Cárdenas-Lizana, J. Wärnå, E. Sulman, D. Y. Murzin, L. Kiwi-Minsker, "Chemoselective liquid phase hydrogenation of 3-nitrostyrene over Pt nanoparticles: Synergy with ZnO support" *Industrial & Engineering Chemistry Research* **54**, (2015): 8659-8669.

[16] J. Song, Z.-F. Huang, L. Pan, K. Li, X. Zhang, L. Wang, J.-J. Zou, "Review on selective hydrogenation of nitroarene by catalytic, photocatalytic and electrocatalytic reactions" *Applied Catalysis B: Environmental* **227**, (2018): 386-408.
[17] Z.-N. Hu, J. Liang, K. Ding, Y. Ai, Q. Liang, H.-b. Sun, "Insight into the selectivity of nano-catalytic nitroarenes reduction over other active groups by exploring hydrogen sources and metal components" *Applied Catalysis A: General* **626**, (2021): 118339.
[18] J. H. Tyler, S. H. Nazari, R. H. Patterson, V. Udumula, S. J. Smith, D. J. Michaelis, "Synthesis of N-aryl and N-heteroaryl hydroxylamines via partial reduction of nitroarenes with soluble nanoparticle catalysts" *Tetrahedron Letters* **58**, (2017): 82-86.
[19] P. Prakash, D. De Masi, V. Geertsen, F. Miserque, H. Li, I. N. N. Namboothiri, E. Gravel, E. Doris, "Selective conversion of nitroarenes to N-aryl hydroxylamines catalysed by carbon-nanotube-supported nickel(II) hydroxide" *ChemistrySelect* **2**, (2017): 5891-5894.
[20] L. Zhang, M. Zhou, A. Wang, T. Zhang, "Selective hydrogenation over supported metal catalysts: From nanoparticles to single atoms" *Chemical Reviews* **120**, (2020): 683-733.
[21] C. Yao, W. Li, X. Ge, Y. Shi, Y. Cao, D. Chen, X. Zhou, X. Duan, "Mechanism and kinetics guided design of catalysts for functionalized nitroarenes hydrogenation" *ChemCatChem* **16**, (2024): e202400027.
[22] M. Macino, A. J. Barnes, S. M. Althahban, R. Qu, E. K. Gibson, D. J. Morgan, S. J. Freakley, N. Dimitratos, C. J. Kiely, X. Gao, A. M. Beale, D. Bethell, Q. He, M. Sankar, G. J. Hutchings, "Tuning of catalytic sites in Pt/TiO_2 catalysts for the chemoselective hydrogenation of 3-nitrostyrene" *Nature Catalysis* **2**, (2019): 873-881.
[23] Q. Wu, B. Zhang, C. Zhang, X. Meng, X. Su, S. Jiang, R. Shi, Y. Li, W. Lin, M. Arai, H. Cheng, F. Zhao, "Significance of surface oxygen-containing groups and heteroatom P species in switching the selectivity of Pt/C catalyst in hydrogenation of 3-nitrostyrene" *Journal of Catalysis* **364**, (2018): 297-307.
[24] A. Corma, P. Serna, P. Concepción, J. J. Calvino, "Transforming nonselective into chemoselective metal catalysts for the hydrogenation of substituted nitroaromatics" *Journal of the American Chemical Society* **130**, (2008): 8748-8753.
[25] A. Yarulin, C. Berguerand, I. Yuranov, F. Cárdenas-Lizana, I. Prokopyeva, L. Kiwi-Minsker, "Pt–Zn nanoparticles supported on porous polymeric matrix for selective 3-nitrostyrene hydrogenation" *Journal of Catalysis* **321**, (2015): 7-12.
[26] M. Lan, B. Zhang, H. Cheng, X. Li, Q. Wu, Z. Ying, Y. Zhu, Y. Li, X. Meng, F. Zhao, "Chemoselective hydrogenation of 3-nitrostyrene to 3-aminostyrene over $Pt-Bi/TiO_2$ catalysts" *Molecular Catalysis* **432**, (2017): 23-30.
[27] M. J. Beier, J.-M. Andanson, A. Baiker, "Tuning the chemoselective hydrogenation of nitrostyrenes catalyzed by ionic liquid-supported platinum nanoparticles" *ACS Catalysis* **2**, (2012): 2587-2595.
[28] H. Wei, X. Liu, A. Wang, L. Zhang, B. Qiao, X. Yang, Y. Huang, S. Miao, J. Liu, T. Zhang, "FeO_x-supported platinum single-atom and pseudo-single-atom catalysts for chemoselective hydrogenation of functionalized nitroarenes" *Nature Communications* **5**, (2014): 5634.
[29] J. F. Moulder, W. F. Stickle, P. E. Sobol, K. D. Bomben, *Handbook of X ray photoelectron spectroscopy*, Perkin-Elmer Corporation, United States of America, (1992).
[30] P. A. Rock, "The standard oxidation potential of the ferrocyanide-ferricyanide electrode at 25° and the entropy of ferrocyanide ion" *The Journal of Physical Chemistry* **70**, (1966): 576-580.
[31] T.-C. Chang Chien, M. F. Delley, "Disentangling chemical interaction and electric fields at electrochemical interfaces" *The Journal of Physical Chemistry C* **129**, (2025): 999-1012.
[32] J. M. Delgado, J. M. Orts, J. M. Pérez, A. Rodes, "Sputtered thin-film gold electrodes for in situ ATR-SEIRAS and SERS studies" *Journal of Electroanalytical Chemistry* **617**, (2008): 130-140.

[33] Yan, Q.-X. Li, S.-J. Huo, M. Ma, W.-B. Cai, M. Osawa, "Ubiquitous strategy for probing ATR surface-enhanced infrared absorption at platinum group metal−electrolyte interfaces" *The Journal of Physical Chemistry B* **109**, (2005): 7900-7906.
[34] J. S. Gordon, D. C. Johnson, "Application of an electrochemical quartz crystal microbalance to a study of water adsorption at gold surfaces in acidic media" *Journal of Electroanalytical Chemistry* **365**, (1994): 267-274.
[35] K. A. Friedrich, F. Henglein, U. Stimming, W. Unkauf, "Investigation of Pt particles on gold substrates by IR spectroscopy particle structure and catalytic activity" *Colloids and Surfaces A: Physicochemical and Engineering Aspects* **134**, (1998): 193-206.
[36] S. Zou, M. J. Weaver, "Surface-enhanced Raman scattering on uniform transition-metal films: Toward a versatile adsorbate vibrational strategy for solid-nonvacuum interfaces?" *Analytical Chemistry* **70**, (1998): 2387-2395.
[37] K. Kunimatsu, H. Uchida, M. Osawa, M. Watanabe, "In situ infrared spectroscopic and electrochemical study of hydrogen electro-oxidation on Pt electrode in sulfuric acid" *Journal of Electroanalytical Chemistry* **587**, (2006): 299-307.
[38] S. Zhu, X. Qin, Y. Yao, M. Shao, "pH-Dependent hydrogen and water binding energies on platinum surfaces as directly probed through surface-enhanced infrared absorption spectroscopy" *Journal of the American Chemical Society* **142**, (2020): 8748-8754.
[39] A. Peremans, A. Tadjeddine, "Vibrational spectroscopy of electrochemically deposited hydrogen on platinum" *Physical Review Letters* **73**, (1994): 3010-3013.
[40] X. Yang, J. Nash, N. Oliveira, Y. Yan, B. Xu, "Understanding the pH dependence of underpotential deposited hydrogen on platinum" *Angewandte Chemie International Edition* **58**, (2019): 17718-17723.
[41] H. Yoshida, K. Kato, J. Wang, X. Meng, S. Narisawa, S.-i. Fujita, Z. Wu, F. Zhao, M. Arai, "Hydrogenation of nitrostyrene with a Pt/TiO_2 catalyst in CO_2-dissolved expanded polar and nonpolar organic liquids: Their macroscopic and microscopic features" *The Journal of Physical Chemistry C* **115**, (2011): 2257-2267.
[42] M. Boronat, P. Concepción, A. Corma, S. González, F. Illas, P. Serna, "A molecular mechanism for the chemoselective hydrogenation of substituted nitroaromatics with nanoparticles of gold on TiO_2 catalysts: A cooperative effect between gold and the support" *Journal of the American Chemical Society* **129**, (2007): 16230-16237.
[43] G. Richner, J. A. van Bokhoven, Y.-M. Neuhold, M. Makosch, K. Hungerbühler, "In situ infrared monitoring of the solid/liquid catalyst interface during the three-phase hydrogenation of nitrobenzene over nanosized Au on TiO_2" *Physical Chemistry Chemical Physics* **13**, (2011): 12463-12471.
[44] R. Parellada, J. Perez-Peña, J. F. Arenas, "Infrared spectrum of acetonitrile-^{15}N" *Journal of Molecular Structure* **51**, (1979): 1-7.
[45] M. G. Rachid, W. R. M. Rocha, H. Linnartz, "Infrared spectra of complex organic molecules in astronomically relevant ice mixtures" *Astronomy & Astrophysics* **665**, (2022): A89.
[46] J. N'Diaye, A. Atifi, "Electrochemically mediated carbon capture on surface-confined quinone: Unveiling interfacial pathways via surface-enhanced spectroscopy" *ACS Electrochemistry* **1**, (2025): 1840-1851.
[47] P. H. Reinsberg, H. Baltruschat, "Potential- and cation-dependent adsorption of acetonitrile on gold investigated via surface enhanced infrared absorption spectroscopy" *Electrochimica Acta* **334**, (2020): 135609.
[48] I. Hamada, Y. Morikawa, "Density-functional analysis of hydrogen on Pt(111): Electric field, solvent, and coverage effects" *The Journal of Physical Chemistry C* **112**, (2008): 10889-10898.

[49] R. E. Clavijo, R. Araya-Maturana, B. K. Cassels, B. Weiss-López, "Infrared spectra of nitrostyrene derivatives" *Spectrochimica Acta Part A: Molecular Spectroscopy* **50**, (1994): 2105-2115.
[50] R. Kas, O. Ayemoba, N. J. Firet, J. Middelkoop, W. A. Smith, A. Cuesta, "In-situ infrared spectroscopy applied to the study of the electrocatalytic reduction of CO_2: Theory, practice and challenges" *ChemPhysChem* **20**, (2019): 2904-2925.
[51] M. Osawa, "Dynamic processes in electrochemical reactions studied by surface-enhanced infrared absorption spectroscopy (SEIRAS)" *Bulletin of the Chemical Society of Japan* **70**, (1997): 2861-2880.
[52] A. M. McCullagh, E. K. Gibson, S. F. Parker, K. Refson, D. Lennon, "The adsorption of nitrobenzene over an alumina-supported palladium catalyst: An infrared spectroscopic study" *Physical Chemistry Chemical Physics* **25**, (2023): 25993-26005.
[53] X.-K. Wan, T.-T. Liu, N.-L. Li, Q. Dai, J. Wei, Q.-M. Wang, "Tailoring atomically precise gold nanoclusters for boosting selective hydrogenation of nitrostyrene with H_2" *ACS Nano* **19**, (2025): 11371-11380.
[54] L. Zhang, X.-M. Cao, P. Hu, "Insight into chemoselectivity of nitroarene hydrogenation: A DFT-D3 study of nitroarene adsorption on metal surfaces under the realistic reaction conditions" *Applied Surface Science* **392**, (2017): 456-471.
[55] L. Zhang, Z.-J. Shao, X.-M. Cao, P. Hu, "Insights into different products of nitrosobenzene and nitrobenzene hydrogenation on Pd(111) under realistic reaction conditions" *The Journal of Physical Chemistry C* **122**, (2018): 20337-20350.
[56] R. A. v. Santen, *Modern heterogeneous catalysis: An introduction*, Wiley-VCH, Germany, (2017).
[57] F. Mittendorfer, C. Thomazeau, P. Raybaud, H. Toulhoat, "Adsorption of unsaturated hydrocarbons on Pd(111) and Pt(111): A DFT study" *The Journal of Physical Chemistry B* **107**, (2003): 12287-12295.
[58] F. Gao, Y. Wang, W. T. Tysoe, "The surface chemistry of propylene, 1-iodopropane, and 1,3-diiodopropane on MoAl alloy thin films formed on dehydroxylated alumina" *The Journal of Physical Chemistry B* **110**, (2006): 12555-12571.
[59] U. Sanyal, S. F. Yuk, K. Koh, M. S. Lee, K. Stoerzinger, D. Zhang, L. C. Meyer, J. A. Lopez-Ruiz, A. Karkamkar, J. D. Holladay, D. M. Camaioni, M. T. Nguyen, V. A. Glezakou, R. Rousseau, O. Y. Gutierrez, J. A. Lercher, "Hydrogen bonding enhances the electrochemical hydrogenation of benzaldehyde in the aqueous phase" *Angewandte Chemie International Edition* **60**, (2021): 290-296.
[60] S. Yang, L. Peng, E. Oveisi, S. Bulut, D. T. Sun, M. Asgari, O. Trukhina, W. L. Queen, "MOF-derived cobalt phosphide/carbon nanocubes for selective hydrogenation of nitroarenes to anilines" *Chemistry – A European Journal* **24**, (2018): 4234-4238.
[61] N. A. Arnosti, G. P. Wasser, Y.-C. Shen, M. F. Delley, "Competitive adsorption and modulated product binding using pyridines on cobalt phosphide enhance hydrogenation catalysis" *Journal of the American Chemical Society* **147**, (2025): 43487-43500.
[62] Z. Wei, S. Mao, F. Sun, J. Wang, B. Mei, Y. Chen, H. Li, Y. Wang, "The synergic effects at the molecular level in CoS_2 for selective hydrogenation of nitroarenes" *Green Chemistry* **20**, (2018): 671-679.
[63] E. Lam, J. J. Corral-Pérez, K. Larmier, G. Noh, P. Wolf, A. Comas-Vives, A. Urakawa, C. Copéret, "CO_2 hydrogenation on Cu/Al_2O_3: Role of the metal/support interface in driving activity and selectivity of a bifunctional catalyst" *Angewandte Chemie International Edition* **58**, (2019): 13989-13996.
